# Hydroquinone/Quinone Cycle for Reductive Photocatalytic Transformations

**DOI:** 10.1021/acs.orglett.5c04963

**Published:** 2026-01-08

**Authors:** Iida Alanko, Anna Lenarda, Xinyu Bao, Alice Genovese, Juan V. Alegre-Requena, Juho Helaja

**Affiliations:** † Department of Chemistry, 111628 University of Helsinki, 00014 Helsinki, Finland; ‡ Department of Inorganic Chemistry, Instituto de Síntesis Quimica ´ y Catalisis ´ Homogenea ´ (ISQCH), CSIC-Universidad de Zaragoza, C/Pedro Cerbuna 12, 50009 Zaragoza, Spain

## Abstract

Phenanthrenehydroquinones (H_2_PQs), generated *in situ* from phenanthrenequinones under visible light using alcohols as hydrogen sources, act as catalytic organic photoreductants. They deoxygenate efficiently diverse N-heterocyclic *N*-oxides, including substrates having more negative reduction potentials than the H_2_PQ excited-state reduction potential ( $E_{H_{2}{PQ}^{+\cdot }/H_{2}{PQ}^{*}}$ = −2.10 V vs SCE), suggesting hydrogen bonding assisted electron transfer. TD-DFT calculations indicate H_2_PQs act as both photoacids and photoreductants. This work demonstrates the harnessing of the hydroquinone/quinone cycle in visible-light-driven reductions.

## Graphical abstract

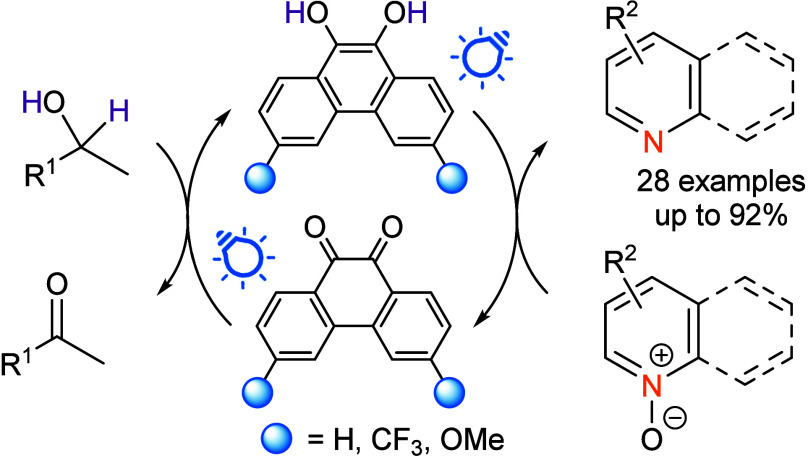

In quinone-catalyzed oxidative transformations, the hydroquinone/quinone redox cycle, typically mediated by oxidants such as NaNO_2_, *t*-BuONO, O_2_, or metal salts, is generally viewed as a simple regenerative step.^1^ A notable exception is the industrial anthraquinone process, where anthrahydroquinone reduces O_2_ to H_2_O_2_.^2^ Although the photoinduced reductive activity of *p*-hydroquinone is known,^3–5^ its synthetic applications remain scarce, with key reports including single-electron transfer (SET) from its singlet (S_1_) state to a cobalt complex,^3^ silver nanoparticle formation,^4^ and acetylacetone reduction.^5^

Inspired by this concept, we envisioned that phenanthrenehydroquinone (H_2_PQ), the reduced form of 9,10-phenanthrenequinone (PQ), could act as a photocatalyst for reductive transformations. Previous studies by Fukuzumi^6^ and our group^7^ have shown that PQ can be converted *in situ* to H_2_PQ under visible light using alcohols as hydrogen sources. While PQ has been widely employed as a photoactivated catalytic oxidant in numerous transformations,^8–10^ these methods require a stoichiometric oxidant to regenerate PQ from H_2_PQ. In contrast, we leveraged H_2_PQ oxidation to drive the photoinduced deoxygenation of N-heterocyclic *N*-oxides. Under blue light irradiation, both pyridine and quinoline *N*-oxides underwent efficient deoxygenation in the presence of PQ and alcohols. To the best of our knowledge, this constitutes the first visible-light-driven reductive application of *ortho*-hydroquinones in organic synthesis.

N-Heterocyclic *N*-oxides are key intermediates in the synthesis of selectively functionalized quinolines, common motives in many biologically active molecules.^11^ Their deoxygenation typically relies on phosphines,^12^ boranes,^13^ or metal-mediated methods.^14^ Recent advances have introduced photochemical alternatives: Cantat employed a Rh-based photocatalyst,^15^ Lee used Ru- or Ir-based photocatalysts with hydrazine hydrate^16^ or Hantzsch ester,^17^ and Jacobi von Wangelin reported a metal-free variant using Hantzsch ester (Scheme 1).^18^ More recently, Lee combined an oxidative organophotocatalyst with *i*-PrOH,^19^ and Guo showed that thioxanthone/TfOH can function as a photoactivated reductive system.^20^ Here, we disclose a mild, metal-free, visible-light-driven deoxygenation that exploits the hydroquinone/quinone redox cycle for photoactivated catalysis.

**Scheme 1:**
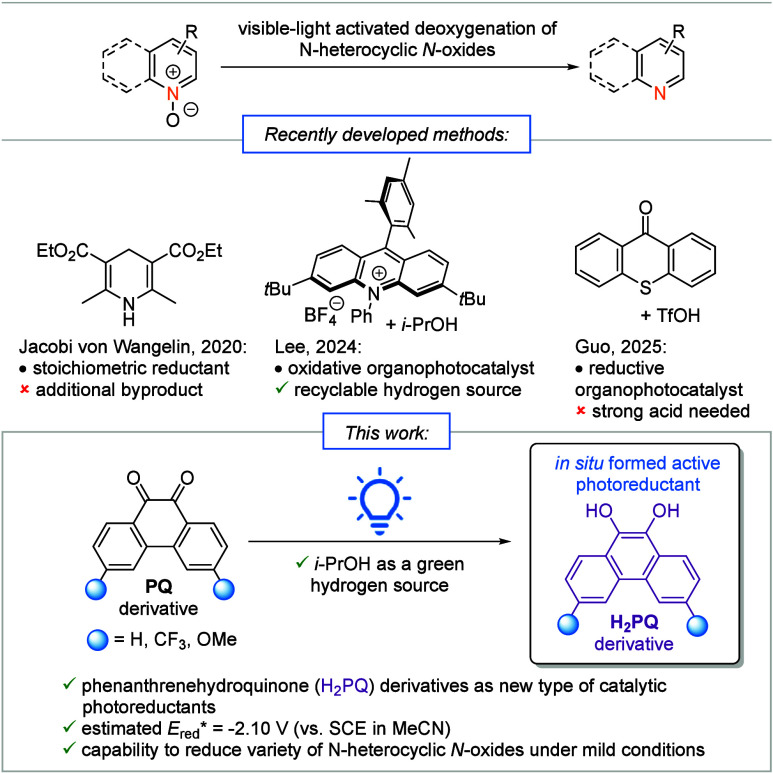
Recent Visible-Light-Activated Methods Based on Organic Photoreductions for the Deoxygenation of N-Heterocyclic *N*-Oxides

We initiated our study using 4-phenylpyridine *N*-oxide **1a** as a starting material, PQ as the photocatalyst, and 1-(4-methoxyphenyl)­ethan-1-ol **A** as a hydrogen source under blue LED (450 nm) irradiation (Table 1). Optimal performance was achieved in MeCN/0.1% H_2_O, whereas higher water content slowed the reaction significantly (). Alternative hydrogen donors, including dithiothreitol **B** ^21^ and 1-methyl-1,4-cyclohexadiene **C**,^22^ proved inferior: **C** promoted side reactions, while **B** gave incomplete conversions in slower reductions. We evaluated the application of different PQ derivatives, 3,6-bis­(trifluoromethyl)-9,10-phenanthrenequinone (PQ-CF_3_) and 3,6-dimethoxy-9,10-phenanthrenequinone (PQ-OMe), as photocatalysts. Although *in situ* formation of more electron-rich H_2_PQ-OMe could suggest stronger reductive power, electron-deficient H_2_PQ-CF_3_ unexpectedly showed the best performance. Our previous work demonstrated that the excited-state PQ-CF_3_ has superior ability to oxidize aliphatic alcohols,^7^ enabling the use of *i*-PrOH as a green and atom-economic hydrogen source. Control experiments confirmed that inert atmosphere is essential, as H_2_PQ species are rapidly reoxidized by O_2_.^23,24^

**Table 1:**
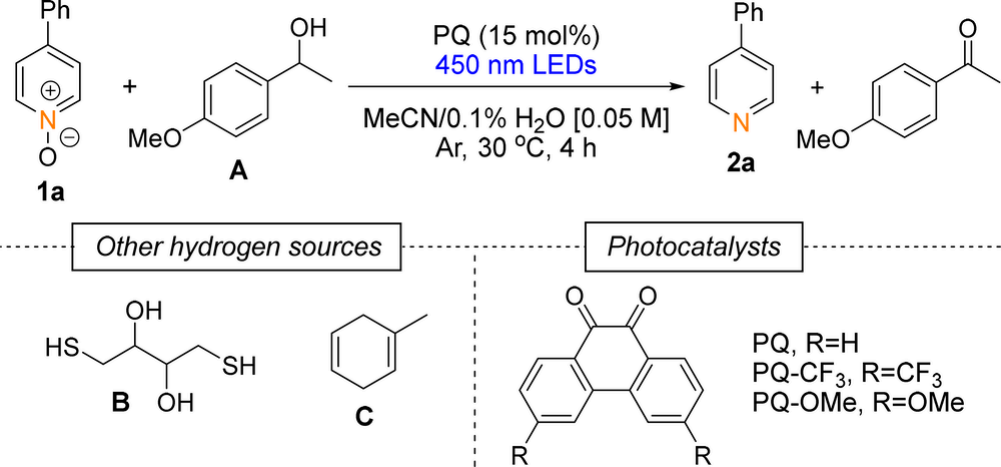
Optimization of the Reaction Conditions^a^

Entry	Variation	Product (%)^b^
1	no	93
2	**B** as hydrogen source	97
3	**C** as hydrogen source	84
4	PQ-OMe as catalyst	83
5	PQ-CF_3_ as catalyst	97
6	PQ-CF_3_, *i*-PrOH (3 equiv), 6 h	95 (86)^c^
7	under air^d^	71
8	no light^d^	0
9	no photocatalyst	0
10	no hydrogen source^d^	7

a Reaction conditions: 0.1 mmol of **1a**, 1.5 equiv of alcohol **A**, 15 mol % of PQ, MeCN/0.1% H_2_O [0.05 M], 450 nm LEDs, argon atmosphere, 30 °C, 4 h. Full optimization in .

b NMR yields. 1,3,5-Trimethoxybenzene was used as an internal standard.

c Reaction was performed in 0.2 mmol scale (isolated yield).

d PQ-CF_3_ was used as the catalyst.

With the optimized reaction conditions in hand, we extended the developed method to the deoxygenation of substituted pyridine *N*-oxides (Scheme 2A). The yields obtained in 16 h varied from fair to very good (47–87%). Substrates containing both electron-withdrawing and electron-donating groups reacted smoothly, and even more sterically hindered substrates (**1j** and **1k**) reacted in good yields, 56% and 69%, respectively. Gratifyingly, the developed method was demonstrated to work on a 1.0 mmol scale with a similar efficiency, delivering 92% of deoxygenated product **2a** in 24 h.

**Scheme 2:**
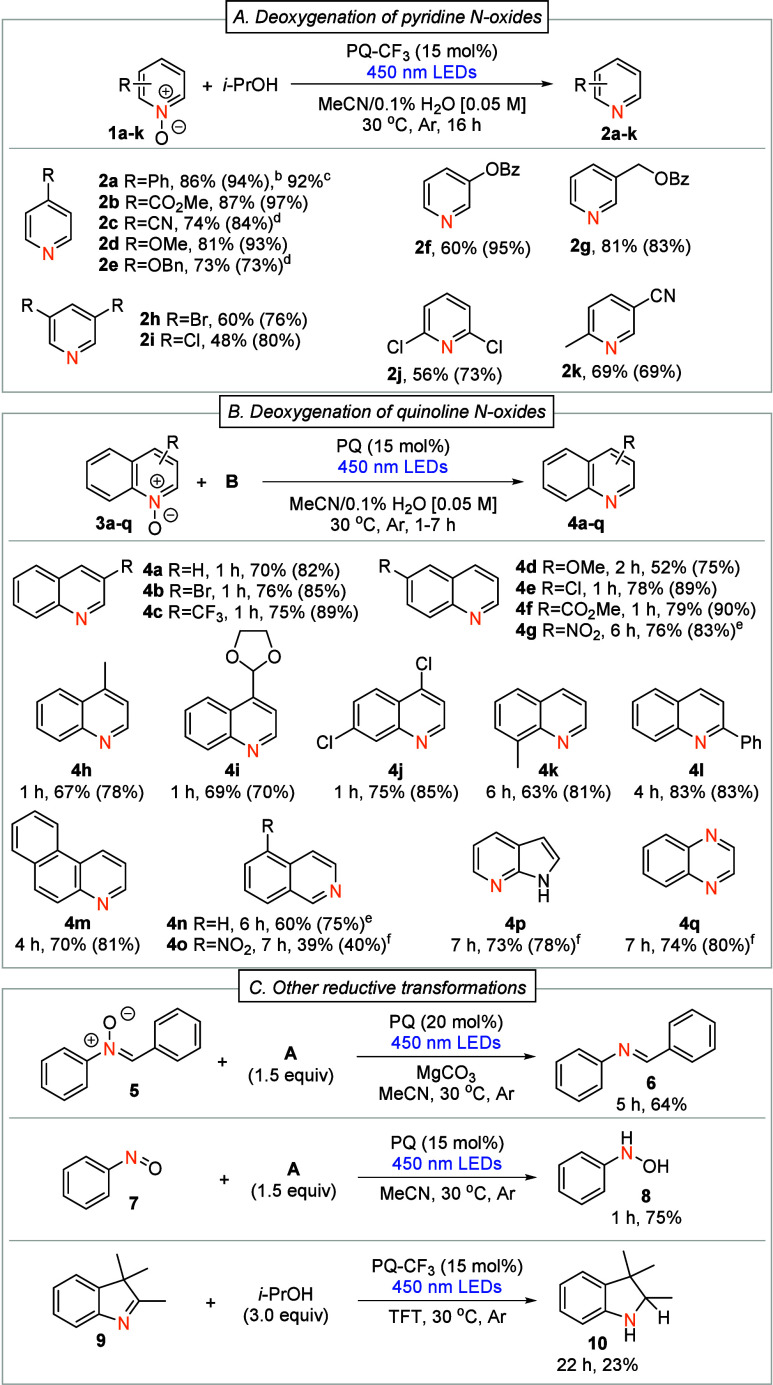
Reaction Scope Study^a^

a All reactions were performed in 0.2 mmol scale. NMR yields are in parentheses, and the yields were determined using 1,3,5-trimethoxybenzene as an internal standard.
b Reaction time 16 h.
c Reaction was performed in 1.0 mmol scale with reaction time of 24 h.
d PQ-CF_3_ (20 mol %) was used.
e PQ (20 mol %) was used.
f PQ (25 mol %) was used.

To expand the scope, we applied the developed method to the deoxygenation of quinoline *N*-oxides (Scheme 2B). Their smooth reactivity allowed the use of PQ as the photocatalyst and **B** as the hydrogen source. Compared to pyridine *N*-oxides, the reactions reached completion notably faster (1–6 h), with yields varying from fair to very good (52–83%). Even more sterically hindered substrates (**3k** and **3l**) were deoxygenated with good yields, 63% and 83%, respectively. Nitro-substituted quinoline **3g** and isoquinolines **3n** and **3o** showed slower reactivity, indicating that less negative reduction potential does not alone favor the reactivity.

The developed method was successfully applied to other reductive transformations (Scheme 2C): deoxygenation of nitrone compound **5** to imine **6** was carried out with a good yield (64%), and nitrosobenzene **7** was hydrogenated quickly to hydroxylamine **8** with a good yield (75%). Additionally, we could hydrogenate cyclic imine **9** achieving amine **10** with 23% yield in 22 h.

Next, we studied the photochemical properties of H_2_PQs with UV–vis and fluorescence spectroscopy. The absorption maxima of H_2_PQs were remarkably blue-shifted compared to PQs (Figures S5–S6). To estimate the reduction ability of the excited-state H_2_PQs, their excited-state reduction potentials ( $E_{H_{2}{PQ}^{+\cdot }/H_{2}{PQ}^{*}}$ ) were estimated by combining ground-state oxidation potentials ( $E_{H_{2}PQ/H_{2}{PQ}^{+\cdot }}$ ) obtained by cyclic voltammetry (Figures S3–S4) with emission measurements.^25^ As H_2_PQs are fluorescent compounds, their fluorescence emission was utilized to determine the excitation energy (*E*_0,0_) (Figure 1A). Unexpectedly, the same value of the $E_{H_{2}{PQ}^{+\cdot }/H_{2}{PQ}^{*}}$ , −2.10 V (vs SCE) was estimated for both H_2_PQ and H_2_PQ-CF_3_ (Figure 1B).

**Figure 1:**
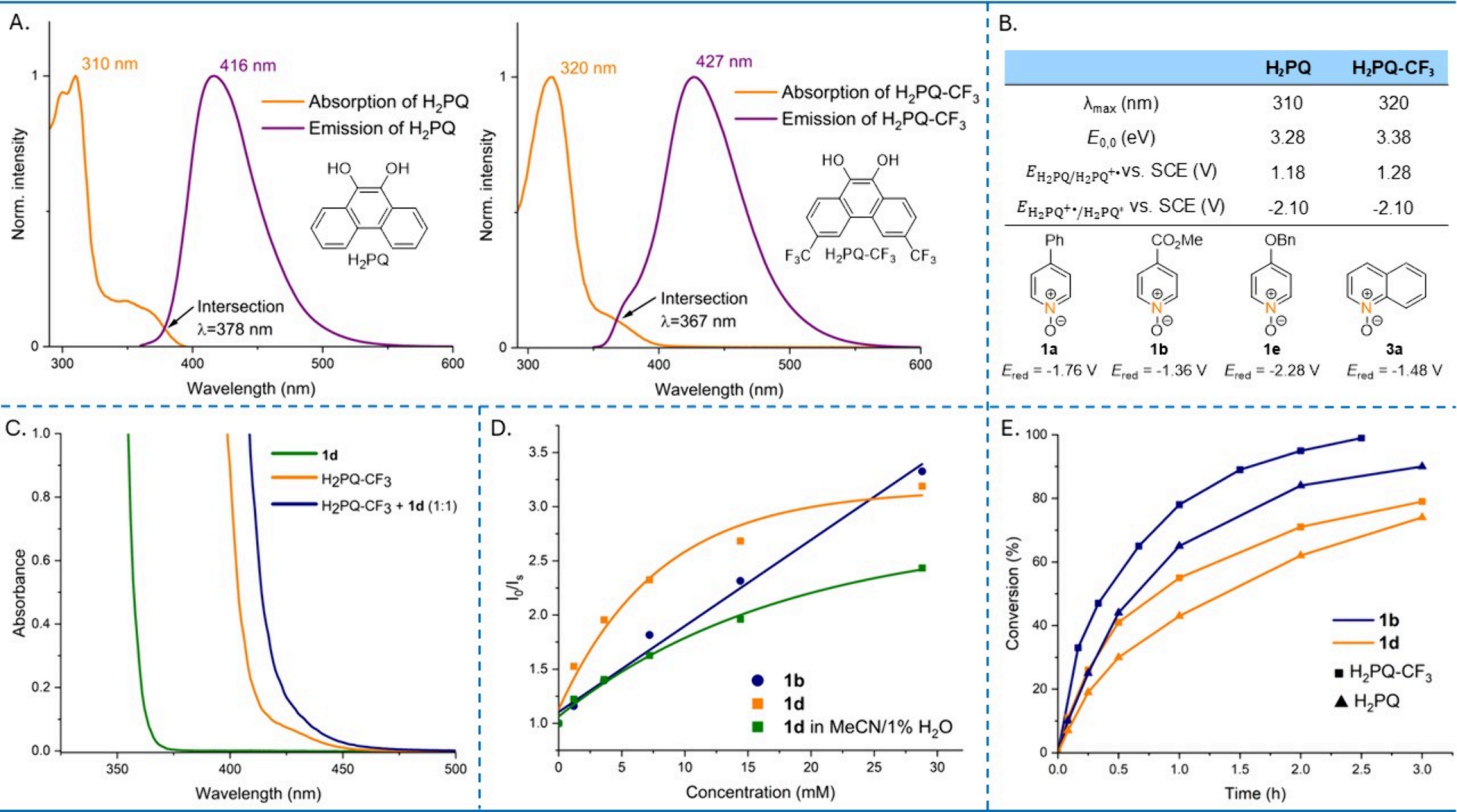
Mechanistic studies. A) Normalized absorption and emission spectra of H_2_PQ and H_2_PQ-CF_3_ in MeCN [0.1 mM]. *E*_0,0_ was determined using the intersection of normalized spectra. B) Estimation of the excited-state reduction potentials of H_2_PQs, and experimental reduction potentials (*E*_red_) of selected substrates measured by cyclic voltammetry. All potentials vs SCE in MeCN. C) UV–vis spectra of substrate **1d**, H_2_PQ-CF_3_, and H_2_PQ-CF_3_ + **1d** (1:1) in MeCN [0.01 M]. D) Stern–Volmer plots extracted from fluorescence quenching experiments of H_2_PQ-CF_3_ using substrates **1b** (in MeCN [0.12 mM]) and **1d** (in MeCN and in MeCN/1% H_2_O [0.12 mM]) as quenchers. E) Reaction kinetic profiles of substrates **1b** and **1d** run by H_2_PQ-CF_3_ (■) and H_2_PQ (▲) in CD_3_CN.

We also measured reduction potentials (*E*_red_) of selected substrates by cyclic voltammetry to evaluate whether the excited-state H_2_PQs were strong enough to reduce all of the substrates (Figure 1B). Comparing the $E_{H_{2}{PQ}^{+\cdot }/H_{2}{PQ}^{*}}$ with the *E*_red_ of the substrates revealed that reduction can occur even when the *E*_red_ of a substrate is clearly more negative than the $E_{H_{2}{PQ}^{+\cdot }/H_{2}{PQ}^{*}}$ , e.g., in the case of electron-rich substrate **1e** (*E*_red_ = −2.28 V vs SCE). This suggests that an additional mechanistic factor may be involved in the reduction event.

To shed light on the possible reaction mechanism, we performed UV–vis and ^1^H NMR experiments to reveal possible interactions between H_2_PQ-CF_3_ and substrates in the ground state. The UV–vis absorption spectrum revealed a clear red shift (∼11 nm) of the H_2_PQ-CF_3_ absorbance when mixed with electron-rich substrate **1d**, indicating the occurrence of a ground-state interaction between the compounds (Figure 1C). ^1^H NMR experiments suggested the interaction to be of a hydrogen-bonding nature since the O*H* signal of H_2_PQ-CF_3_ in the same conditions appears remarkably shifted (from 7.23 to 10.66 ppm, ). Furthermore, a light on/off experiment confirmed the importance of light activation of hydroquinones to induce *N*-oxides’ deoxygenation ().

Fluorescence quenching experiments were performed with two electronically distinct *N*-oxides, **1b** and **1d**, to evaluate their ability to quench the excited state of H_2_PQ-CF_3_ (Figure 1D). Substrate **1b** exhibits a linear Stern–Volmer plot, consistent with a single (collisional) quenching pathway.^26^ In contrast, **1d** shows a pronounced downward curvature, characteristic of weak ground-state association or other heterogeneity that creates subpopulations with reduced quenching efficiency.^26^ In our system, we attribute this behavior to strong hydrogen bonding between electron-rich **1d** and H_2_PQ-CF_3_, as supported by the UV–vis spectral changes (Figure 1C) and NMR data (). Upon addition of 1% H_2_O to MeCN, quenching by **1d** becomes less efficient and more linear, in line with the diminished hydrogen bonding interaction.

Next, we studied the reaction kinetics of H_2_PQ and H_2_PQ-CF_3_ with substrates **1b** and **1d**, using stoichiometric amounts of catalyst to exclude the effect resulting from its regeneration (Figure 1E). As expected from their reduction potentials, **1b** was deoxygenated faster than **1d** by both H_2_PQs. Notably, H_2_PQ-CF_3_ showed a higher reactivity than H_2_PQ, likely due to stronger hydrogen bonding with the substrates.

To gain theoretical insight into the mechanism, we performed computational studies using density-functional theory (DFT) and time-dependent DFT (TD-DFT) for excited states.^27,28^ The results show that the first excited S_1_ state is predominantly characterized by the HOMO–LUMO transition of H_2_PQ. This transition involves a π → π* excitation () that occurs upon absorption of light by H_2_PQ, as previously suggested.^29^ Upon excitation of the catalyst to the S_1_ state, the product forms through two highly exergonic hydrogen transfer steps (−43.9 and – 115.1 kcal·mol^–1^, Figure 2A). The results suggest that the first hydrogen transfer could proceed readily via consecutive proton and electron transfers, with two possible pathways depending on the order of these transfers. Both pathways show favorable reaction ΔG (−0.9 and – 1.6 kcal·mol^–1^, Figure 2B, top), and, in principle, the reaction could proceed through either. Kinetically, the deprotonation steps in both pathways are barrierless (). These findings may explain why Stern–Volmer plots exhibit substrate-dependent behavior, linear or nonlinear (Figure 1D), reflecting an interplay between competing mechanisms.

**Figure 2:**
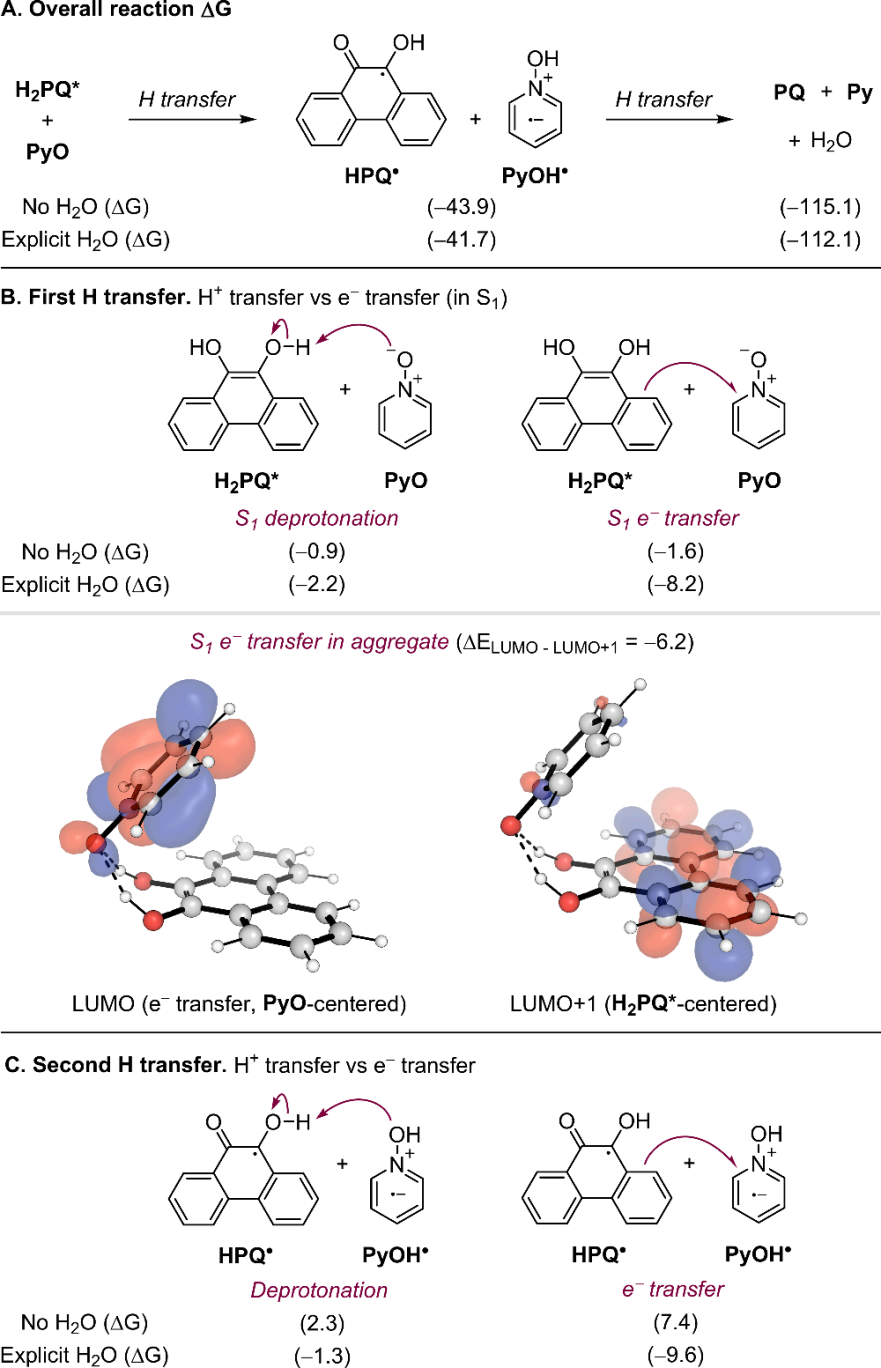
Computational mechanistic studies. A) Reaction Gibbs free energies (ΔG) for the two hydrogen transfer steps. B) Comparison of ΔG for proton vs electron transfer in the first reaction step, along with TD-DFT calculations of the two potential orbitals that might receive the electron after photoexcitation. C) Comparison of ΔG for proton vs electron transfer in the second reaction step. All energies in kcal·mol^–1^. DFT methods: M06–2X/def2-TZVPP//M06–2*X*/6–31+G­(d,p), including solvation effects with SMD in all calculations.

While the initial proton transfer can be rationalized by the known increase in acidity of phenolic compounds due to photoexcitation,^30^ an initial electron transfer seems less intuitive. To validate this possibility, we analyzed the system using TD-DFT and found that the π* orbital of PyO is more stabilized than that of H_2_PQ in the PyO···H_2_PQ aggregate (−6.2 kcal·mol^–1^, Figure 2B, bottom). This result supports the DFT findings and suggests that an excited electron in the catalyst···substrate aggregate would preferentially transfer into the substrate’s π* system, in line with a favorable electron transfer.

The second hydrogen transfer step likely proceeds via an initial proton transfer as the ΔG of the competing electron transfer pathway is significantly higher in energy (2.3 vs 7.4 kcal·mol^–1^, Figure 2C). However, as noted above, the dominant pathway may vary depending on the combination of catalyst and substrate involved. Although we identified plausible stepwise mechanisms, other competing pathways such as concerted proton-coupled electron transfer (PCET) or direct hydrogen atom transfer (HAT) might also contribute. The proposed mechanism cycle is illustrated in .

The observed accelerating effect induced by small amounts of water in the reaction () was also investigated computationally by adding an explicit water molecule to all of the intermediate species. In terms of overall thermodynamics, the reaction energies after each hydrogen transfer remain similarly exergonic upon the addition of water (Figure 2A, – 41.7 and – 112.1 kcal·mol^–1^ for the first and second transfers, respectively). However, water exhibits a significant stabilizing effect on the ionic intermediates formed after the individual deprotonation steps and electron transfers. This effect is more pronounced in the electron transfer steps, where the reaction energies decrease from – 1.6 to – 8.2 kcal·mol^–1^ (Figure 2B) in the first transfer and from 7.4 to – 9.6 kcal·mol^–1^ (Figure 2C) in the second. These preliminary results suggest that water may accelerate the reaction by stabilizing the ionic intermediates generated following electron transfer and deprotonation.

In summary, we have demonstrated that phenanthrenehydroquinones (H_2_PQs) can act as effective novel photoactivated catalytic reductants for the deoxygenation of N-heterocyclic *N*-oxides. The active catalyst is generated *in situ* from phenanthrenequinone (PQ) derivatives via visible-light-activated alcohol oxidation. Mechanistic studies suggest that the photoacidity of H_2_PQs is the key driving force for the reductions. The demonstrated use of the hydroquinone/quinone cycle in the deoxygenations of *N*-oxides highlights its broader potential for reductive transformations.

## Supplementary Material





## Data Availability

The data underlying this study are available in the published article and its online .

## Notes

Text file of all computed molecule Cartesian coordinates in a format for convenient visualization (XYZ)
Experimental procedures, full reaction optimization, mechanistic studies, NMR data, and computational data (PDF)
