# Supplementary material for: Hydroquinone/Quinone Cycle for Reductive Photocatalytic Transformations
Source: Org Lett. 2026 Jan 8;28(3):971–6. doi: 10.1021/acs.orglett.5c04963 (PMC12836346; doi:10.1021/acs.orglett.5c04963)
Supplement: Supplementary file 2 [file ol5c04963_si_002.pdf]

Supporting Information

Hydroquinone/Quinone Cycle for Reductive Photocatalytic Transformations

Iida Alanko[1], Anna Lenarda[1], Xinyu Bao[1], Alice Genovese[2], Juan V. Alegre-Requena[2]*, and Juho Helaja[1]*

[1] Department of Chemistry, University of Helsinki, A.I. Virtasen Aukio 1, P.O. Box 55, 00014 Finland
[2] Department of Inorganic Chemistry, Instituto de Síntesis Química y Catálisis Homogénea (ISQCH), CSIC-Universidad de Zaragoza, C/ Pedro Cerbuna 12, 50009 Zaragoza, Spain

* Correspondence to: Juho Helaja, juho.helaja@helsinki.fi; Juan V. Alegre-Requena, jv.alegre@csic.es

Supporting Information

Contents

General information S3

Preparation of PQ-CF_3 S4

Preparation of substrates S5

General procedure A: Preparation of N-heterocyclic *N*-oxides S5

Optimization of reaction conditions S13

General screening procedure S13

Scope of the reaction S15

General procedure B: Photocatalytic deoxygenation of pyridines S15

General procedure C: Photocatalytic deoxygenation of quinolines S15

Scale-up experiment S24

Other substrates S25

Mechanistic studies S26

Cyclic voltammetry experiments S26

UV-Vis absorption experiments S27

Emission experiments S30

Emission quenching experiment S32

Determination of excited state reduction potentials of H_2PQs S34

NMR experiments S35

Reaction kinetic experiments S36

Light ON/OFF experiment S37

Computational details S38

The proposed mechanism S43

Copies of NMR spectra S44

References S103

General information

All commercially available chemicals and solvents (Alfa Aesar, BLDpharm, Fluorochem, Sigma-Aldrich, TCI, VWR) were used as received without further purification. Anhydrous solvents (DCM, dioxane) were dried over 4 Å molecular sieves prior to use. Anhydrous THF was taken from the VAC Solvent Purification System and used immediately. Glassware was stored in ambient conditions before use unless otherwise specified. NMR spectra were recorded at 25 ˚C using Bruker Avance Neo 400 MHz or 500 MHz spectrometers. $CDCl_3$ containing TMS and DMSO-d_6 were used as deuterated solvents with solvent signals (0.00 ppm (from TMS), 77.16 ppm, and 2.50 ppm and 39.52 ppm, respectively) as references for chemical shifts. The high-resolution mass spectra (HRMS) were obtained using Jeol MStation JMS-700 (EI) instrument with a quadrupole mass analyzer. Thin-layer chromatography (TLC) was conducted using silica gel F_{254} with fluorescence indicator (254 nm). Chromatographic separations were performed with VWR silica gel (40–63 µm). The photoreaction set-up is shown in Figure S1. The photoreactor consisted of an aluminum block with build-in water cooling, and the light source was placed on the bottom of the reactor. The light source was 3×3W ProLight Opto royal blue (450–460 nm) LEDs, whose distance from the reaction vial was 4 mm. The reactions were perforemd in 20 mL glass vials. Temperature was maintained at 30 ˚C.

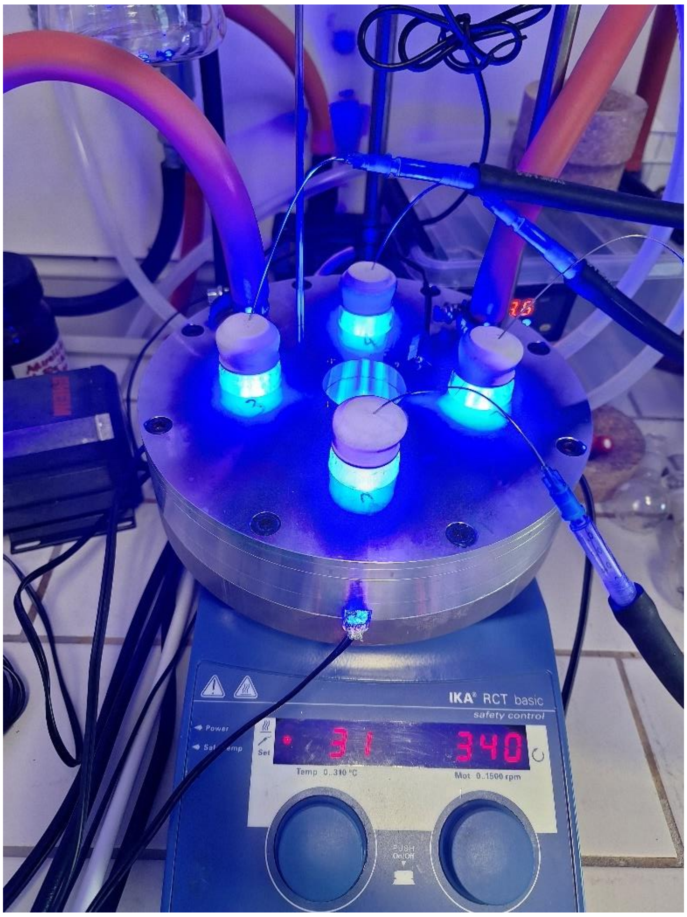

Figure S1: The photoreaction set-up.

Preparation of PQ-CF_3

PQ-CF_3 was synthesized according to a published literature procedure.[1]

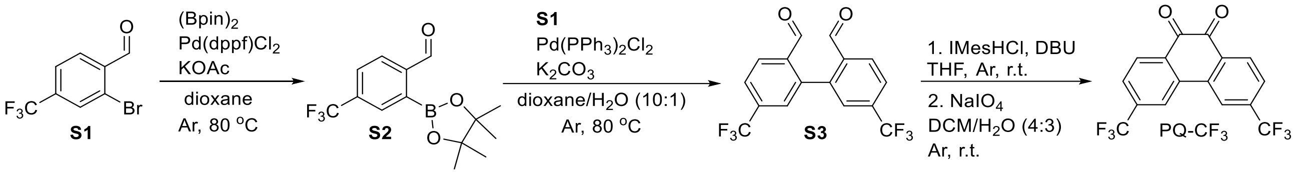

Synthesis of S3

An oven-dried two-neck flask was charged with $(Bpin)_2$ (2.53 g, 9.9 mmol, 1.5 equiv), KOAc (1.63 g, 16.6 mmol, 2.5 equiv), and Pd(dppf)Cl_2 (48.8 mg, 0.067 mmol, 1 mol%), and the flask was evacuated and backfilled with argon (3×). Anhydrous dioxane (27 mL), and **S1** (1.0 mL, 6.6 mmol, 1.0 equiv) were added to the flask under argon, and the reaction mixture was bubbled with argon for 30 min. The reaction mixture was stirred at 80 ˚C in an oil bath for 5 h, until the reaction was complete according to ^{1}H NMR. The reaction mixture was cooled down, filtered through Celite, and the pad of Celite was washed with EtOAc. The filtrate was washed with water (1×40 mL), and the aqueous phase was extracted with EtOAc (3×20 mL). The combined organic phases were washed with brine (1×50 mL) and dried over Na_2SO_4. The solution was concentrated on a rotary evaporator, and the residue was used directly in the next step.

A two-neck flask was charged with K_2CO_3 (1.83 g, 13.2 mmol, 2.0 equiv) and $Pd(PPh_3)_2Cl_2$ (185.7 mg, 0.27 mmol, 4 mol%), and the flask was evacuated and backfilled with argon (3×). The residue from the previous step was dissolved in dioxane (30 mL) and added to the flask. **S1** (1.0 mL, 6.6 mmol, 1.0 equiv) and water (3 mL) were added to the flask, and the reaction mixture was bubbled with argon for 30 min. The reaction mixture was stirred at 80 ˚C in an oil bath for 2 h, until the reaction was complete according to ^{1}H NMR. The reaction mixture was cooled down, filtered through Celite, and the pad of Celite was washed with EtOAc. The filtrate was washed with water (1×40 mL), and the aqueous phase was extracted with EtOAc (3×20 mL). The combined organic phases were washed with brine (1×50 mL) and dried over Na_2SO_4. The solution was concentrated on a rotary evaporator, and the residue was purified with flash chromatography (silica gel, *n*-Hex/EtOAc 10:1 → 5:1). The yield of the orange viscous oil over two steps was 91% (2.08 g).

^{1}H NMR (400 MHz, $CDCl_3$) δ 9.88 (s, 2H), 8.18 (d, *J* = 8.1 Hz, 2H), 7.92 (d, *J* = 8.1 Hz, 2H), 7.62 (s, 2H).
^{13}C NMR (100 MHz, $CDCl_3$) δ 189.6, 140.2, 136.8, 135.3 (q, *J* = 33.2 Hz), 130.7, 128.3 (q, *J* = 3.7 Hz), 126.4 (q, *J* = 3.6 Hz), 123.2 (q, *J* = 273.3 Hz).
The characterization data was in agreement with the previously published data.[2]

Synthesis of PQ-CF_3

An oven-dried two-neck flask was charged with IMesHCl (139.4 mg, 0.41 mmol, 10 mol%), and **S3** (1.42 g, 4.1 mmol, 1.0 equiv) was dissolved in anhydrous THF (20 mL), and the solution was added to the flask. Additional 20 mL of THF was added and the suspension was bubbled with argon for 30 min. DBU (0.67 mL, 4.5 mmol, 1.1 equiv), was added dropwise to the reaction mixture, and the reaction mixture was stirred at r.t. for 30 min, until the reaction was complete according to ^{1}H NMR. The reaction was quenched with 2 M HCl, and the solution was extracted with EtOAc (3×20 mL). The combined organic phases were washed with water (1×30 mL) and brine (1×30 mL), and the solution was concentrated on a rotary evaporator.

The residue from the previous step was dissolved in DCM (40 mL), and the resulting suspension was bubbled with argon for 30 min. The degassed solution was combined with degassed aqueous 0.15 M $NaIO_4$ solution

(964.1 mg, 4.5 mmol, 1.1 equiv in 30 mL), and the biphasic mixture was stirred vigorously for 5 min at r.t. The organic phase was separated, and the aqueous phase was extracted three times with DCM (3×15 mL). The combined organic phases were washed with water (1×50 mL), and the solvent was evaporated. The crude product was purified with flash chromatography (silica gel, *n*-Hex/EtOAc 5:1). The yield of the orange crystalline product over two steps was 77% (1.09 g).

^{1}H NMR (400 MHz, $CDCl_3$) δ 8.37 (d, *J* = 8.1 Hz, 2H), 8.30 (s, 2H), 7.82 (d, *J* = 8.4 Hz, 2H).
^{13}C NMR (101 MHz, $CDCl_3$) δ 178.7, 137.6 (q, *J* = 33.2 Hz), 135.3, 133.2, 131.6, 127.1 (q, *J* = 3.5 Hz), 123.2 (q, *J* = 273.7 Hz), 121.5 (q, *J* = 3.6 Hz).
The characterization data was in agreement with the previously published data.[1]

Preparation of substrates

General procedure A: Preparation of N-heterocyclic *N*-oxides

The heterocyclic *N*-oxides were synthesized according to a slightly modified literature procedure.[3] An example for synthesis of compound **1a**: 4-Phenylpyridine (778.0 mg, 5.0 mmol, 1.0 equiv) was dissolved to anhydrous DCM (5 mL) and the solution was placed under argon. *m*-CPBA (77 w%, 1.234 g, 5.5 mmol, 1.1. equiv) was dissolved to DCM (20 mL), and the mixture was added to the solution of *N*-oxide at 0 ˚C. The reaction mixture was stirred for 1 h at 0 ˚C after which it was allowed to warm slowly to r.t. and stirring was continued until the reaction was complete according to TLC. After completion of the reaction, 10 w% aqueous Na_2SO_3 solution (10 mL) and saturated $NaHCO_3$ solution (10 mL) were added, and the phases were separated. The aqueous phase was extracted with DCM (3×20 mL), and the combined organic phases were washed with saturated $NaHCO_3$ solution (1×40 mL) and brine (1×40 mL). The organic phase was dried over Na_2SO_4, filtered, and concentrated on a rotary evaporator. The crude product was purified with flash chromatography on silica gel.

Characterization of products

4-Phenylpyridine *N*-oxide **1a**

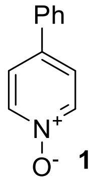
1a

The title compound was prepared from 4-phenylpyridine (778.0 mg, 5.0 mmol) according to the general procedure A. The product was purified with flash chromatography (silica gel, EtOAc/MeOH 10:1) and obtained as a white solid (792.0 mg, 92%).

^{1}H NMR (400 MHz, $CDCl_3$) δ 8.28–8.24 (m, 2H), 7.61–7.57 (m, 2H), 7.53–7.42 (m, 5H).
^{13}C NMR (101 MHz, $CDCl_3$) δ 139.5, 139.2, 136.2, 129.5, 129.4, 126.5, 123.9.
The characterization data was in agreement with the previously published data.[4]

4-Cyanopyridine *N*-oxide **1c**

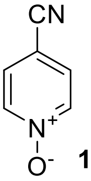
1c

The compound was prepared from 4-cyanopyridine (610.0 mg, 5.9 mmol) according to the general procedure A. The product was purified with flash chromatography (silica gel, DCM/MeOH 40:1) and obtained as white powder (450.0 mg, 64% yield).

^{1}H NMR (400 MHz, $CDCl_3$) δ 8.25–8.22 (m, 2H), 7.55–7.52 (m, 2H).
^{13}C NMR (101 MHz, $CDCl_3$) δ 140.4, 129.1, 115.9, 107.8.
The characterization data was in agreement with the previously published data.[5]

4-(Benzyloxy)pyridine *N*-oxide **1e**

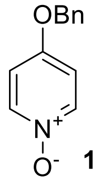

1e

The title compound was prepared from 4-(benzyloxy)pyridine (1.01 g, 5.4 mmol) according to the general procedure A. The product was purified with flash chromatography (silica gel, DCM/MeOH 10:1) and obtained as white powder (745.0 mg, 68%).

^{1}H NMR (400 MHz, $CDCl_3$) δ 8.14–8.10 (m, 2H), 7.45–7.35 (m, 5H), 6.89–6.84 (m, 2H), 5.10 (s, 2H).
^{13}C NMR (101 MHz, $CDCl_3$) δ 157.1, 140.3, 134.9, 129.0, 128.9, 127.7, 112.7, 71.1.
The characterization data was in agreement with the previously published data.[6]

3-(Benzoyloxy)pyridine *N*-oxide **1f**

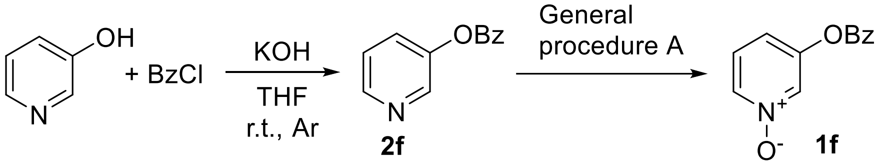

Compound **2f** was prepared according to a literature procedure[7] from 3-hydroxypyridine (503.3 mg, 5.3 mmol), benzoyl chloride (0.68 mL, 5.9 mmol), and KOH (325.3 mg, 5.8 mmol) in anhydrous THF (13 mL). The product was purified with flash chromatography (silica gel, *n*-Hex/EtOAc 3:1/0.5% TEA) and obtained as a white solid (890.0 mg, 84%).

The title compound was prepared from compound **2f** (404.1 mg, 2.0 mmol) according to the general procedure A. The product was purified with flash chromatography (silica gel, EtOAc/8% MeOH) and obtained as white powder (316.7 mg, 73%). mp: 119–121 ˚C

^{1}H NMR (400 MHz, $CDCl_3$) δ 8.27 (t, *J* = 1.9 Hz, 1H), 8.20 – 8.13 (m, 3H), 7.72–7.67 (m, 1H), 7.58–7.52 (m, 2H), 7.33 (dd, *J* = 8.6, 6.3 Hz, 1H), 7.28–7.24 (m, 1H).
^{13}C NMR (101 MHz, $CDCl_3$) δ 163.9, 149.7, 137.0, 134.6, 134.5, 130.5, 129.0, 128.1, 125.5, 120.1.
HRMS (EI) *m/z*: calculated for $C_{12}H_9NO_3$ [M^+]: 215.0582, found: 215.0578

3-((Benzoyloxy)methyl)pyridine *N*-oxide **1g**

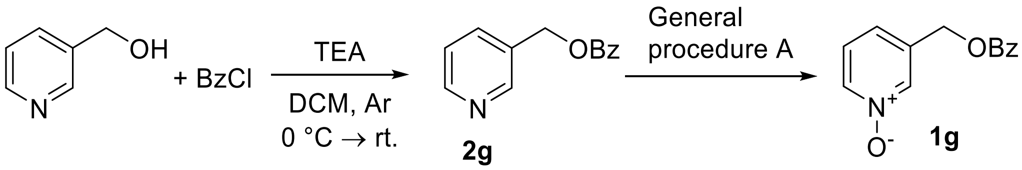

Compound **2g** was prepared according to a literature procedure[8] from 3-hydroxymethylpyridine (651.9 mg, 6.0 mmol), benzoyl chloride (0.84 mL, 7.2 mmol), and TEA (0.92 mL, 6.6 mmol) in anhydrous DCM (15 mL). The product was purified with flash chromatography (silica gel, 3:1 → 1:1 /0.5% TEA) and obtained as pale yellow oil (1.09 g, 86%).

The title compound was prepared from compound **2g** (651.5 mg, 3.1 mmol) according to the general procedure A. The product was purified with flash chromatography (silica gel, EtOAc/8% MeOH) as a white solid (632.4 mg, 90%). mp: 96–97 ˚C

^{1}H NMR (400 MHz, $CDCl_3$) δ 8.36 (s, 1H), 8.19 (dt, *J* = 6.0, 1.6 Hz, 1H), 8.07 (m, 2H), 7.64–7.58 (m, 1H), 7.50–7.44 (m, 2H), 7.35–7.28 (m, 2H), 5.34 (s, 2H).
^{13}C NMR (101 MHz, $CDCl_3$) δ 166.0, 138.9, 138.7, 136.0, 133.8, 129.9, 129.2, 128.7, 126.0, 125.2, 62.8.
HRMS (EI) *m/z*: calculated for $C_{13}H_{11}NO_3$ [M^+]: 229.0739, found: 229.0739

5-Cyano-2-methylpyridine *N*-oxide **1k**

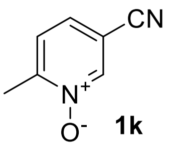

1k

The title compound was prepared from 5-cyano-2-methylpyridine (472.2 mg, 4.0 mmol) according to the general procedure A. The product was purified with flash chromatography (silica gel, EtOAc/MeOH 10:1) and obtained as a white solid (348.0 mg, 65%).

^{1}H NMR (400 MHz, $CDCl_3$) δ 8.49 (s, 1H), 7.40 (br s, 2H), 2.57 (s, 3H).
^{13}C NMR (101 MHz, $CDCl_3$) δ 154.4, 141.7, 127.1, 127.0, 114.4, 110.5, 18.3.
The characterization data was in agreement with the previously published data.[9]

3-Cyano-4-methylpyridine *N*-oxide **S4**

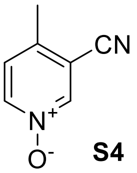

S4

The title compound was prepared from 3-cyano-4-methylpyridine (474.5 mg, 4.0 mmol) according to the general procedure A. The product was purified with flash chromatogrpahy (silica gel, EtOAc 100% → EtOAc/8% MeOH) and obtained as a white solid (251.1 mg, 47%).

^{1}H NMR (400 MHz, $CDCl_3$) δ 8.37 (d, *J* = 1.9 Hz, 1H), 8.24 (dd, *J* = 6.8, 1.8 Hz, 1H), 7.24 (d, *J* = 6.8 Hz, 1H), 2.55 (s, 3H).
^{13}C NMR (101 MHz, $CDCl_3$) δ 142.5, 141.2, 139.7, 127.9, 113.6, 113.5, 19.5.
HRMS (EI) *m/z*: calculated for $C_7H_6N_2O$ [M^+]: 134.0480, found: 134.0475

3-Bromo-5-methoxypyridine *N*-oxide **S5**

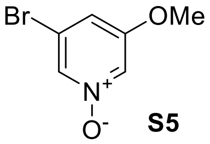

S5

The title compound was prepared from 3-bromo-5-methoxypyridine (530.0 mg, 2.8 mmol) according to the general procedure A. The product was purified with flash chromatography (silica gel, EtOAc/MeOH 10:1) and obtained as white needles (504.0 mg, 88%).

^{1}H NMR (400 MHz, $CDCl_3$) δ 8.02 (t, *J* = 1.5 Hz, 1H), 7.90 (dd, *J* = 2.1, 1.5 Hz, 1H), 7.03 (dd, *J* = 2.1, 1.4 Hz, 1H), 3.85 (s, 3H).
^{13}C NMR (101 MHz, $CDCl_3$) δ 157.8, 134.2, 127.1, 120.1, 116.1, 56.6.
The characterization data was in agreement with the previously published data.[10]

3-Bromo-5-(trifluoromethyl)pyridine *N*-oxide **S6**

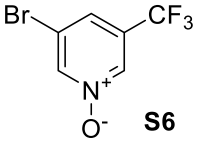

S6

The title compound was prepared from 3-bromo-5-(trifluoromethyl)pyridine (455.0 mg, 2.0 mmol) according to the general procedure A. The product was purified with flash chromatography (silica gel, *n*-Hex/EtOAc 1:1) and obtained as a white solid (198.0 mg, 41%). mp: 74–75 ˚C

^{1}H NMR (400 MHz, $CDCl_3$) δ 8.47 (s, 1H), 8.39 (s, 1H), 7.59 (s, 1H).
^{13}C NMR (101 MHz, $CDCl_3$) δ 143.5, 135.9, 130.8 (q, *J* = 35.4 Hz), 124.8 (q, *J* = 3.5 Hz), 121.23 (q, *J* = 273.9 Hz), 121.20.
^{19}F NMR (376 MHz, $CDCl_3$) δ -63.34.
HRMS (EI) *m/z*: calculated for $C_6H_3BrF_3NO$ [M^+]: 240.9350, found: 240.9350

3-(Benzyloxy)pyridine *N*-oxide **S8**

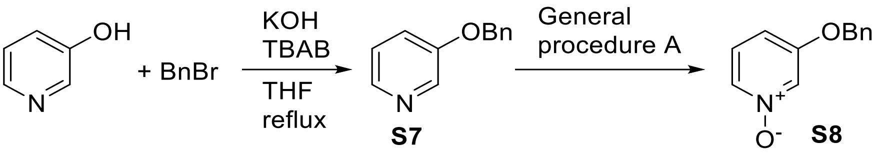

Compound **S7** was prepared according to a literature procedure[11] from 3-hydroxypyridine (703.3 mg, 7.4 mmol), benzyl bromide (1.3 mL, 10.9 mmol), KOH (825.0 mg, 14.7 mmol) and tetrabutylammonium bromide (119.3 mg, 0.37 mmol) by refluxing in anhydrous THF (20 mL) in an oil bath. The product was purified with flash chromatography (silica gel, *n*-Hex/EtOAc 3:1 → 1:1 /0.5% TEA) and obtained as a yellow oil (209.4 mg, 15%).

The title compound was prepared from **S7** (188.7 mg, 1.0 mmol) according to the general procedure A. The product was purified with flash chromatography (silica gel, EtOAc/8% MeOH) and obtained as a white solid (178.6 mg, 87%).

^{1}H NMR (400 MHz, DMSO-d_6) δ 8.11 (t, *J* = 2.0 Hz, 1H), 7.90–7.86 (m, 1H), 7.47–7.29 (m, 6H), 7.09 (dd, *J* = 8.7, 2.3 Hz, 1H), 5.18 (s, 2H).
^{13}C NMR (101 MHz, DMSO-d_6) δ 156.8, 135.8, 132.2, 128.5, 128.2, 128.0, 127.8, 126.1, 112.9, 70.2.
The characterization data was in agreement with the previously published data.[12]

Quinoline *N*-oxide **3a**

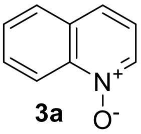

3a

The title compound was prepared from quinoline (0.48 mL, 4.1 mmol) according to the general procedure A. The product was purified with flash chromatography (silica gel, EtOAc/MeOH 10:1) and obtained as a white solid (535.4 mg, 91%).

^{1}H NMR (400 MHz, $CDCl_3$) δ 8.76 (d, *J* = 8.9 Hz, 1H), 8.54 (dd, *J* = 6.1, 1.1 Hz, 1H), 7.87 (d, *J* = 8.1 Hz, 1H), 7.79–7.72 (m, 2H), 7.65 (m, 1H), 7.30 (dd, *J* = 8.5, 6.1 Hz, 1H).
^{13}C NMR (101 MHz, $CDCl_3$) δ 141.7, 135.6, 130.6, 130.5, 128.8, 128.2, 125.9, 121.0, 119.8.
The characterization data was in agreement with the previously published data.[5]

3-Bromoquinoline *N*-oxide **3b**

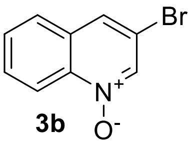

3b

The title compound was prepared from 3-bromoquinoline (0.80 mL, 5.9 mmol) according to the general procedure A. The product was purified with flash chromatography (silica gel, *n*-Hex/EtOAc 1:1) and obtained as a white solid (1.208 g, 91%).

^{1}H NMR (400 MHz, $CDCl_3$) δ 8.67 (d, *J* = 8.8 Hz, 1H), 8.62 (d, *J* = 1.6 Hz, 1H), 7.89 (s, 1H), 7.81–7.74 (m, 2H), 7.67 (m, 1H).
^{13}C NMR (101 MHz, $CDCl_3$) δ 140.6, 137.1, 130.5, 130.3, 129.9, 127.5, 127.4, 119.9, 114.4.
The characterization data was in agreement with the previously published data.[5]

3-(Trifluoromethyl)quinoline *N*-oxide **3c**

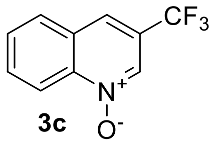

3c

The title compound was prepared from 3-(trifluoromethyl)quinoline (196.2 mg, 1.0 mmol) according to the general procedure A. The product was purified with flash chromatography (silica gel, *n*-Hex/EtOAc 1:1) and obtained as a white solid (189.6 mg, 89%). mp: 117–119 ˚C

^{1}H NMR (400 MHz, $CDCl_3$) δ 8.77 (d, *J* = 8.8 Hz, 1H), 8.71 (s, 1H), 8.02–7.96 (m, 2H), 7.92–7.87 (m, 1H), 7.80–7.75 (m, 1H).
^{13}C NMR (101 MHz, $CDCl_3$) δ 142.9, 132.6, 131.9, 130.2, 129.3, 129.1, 125.1 (q, *J* = 34.7 Hz), 122.6 (q, *J* = 4.3 Hz), 122.4 (q, *J* = 272.7 Hz), 120.1.
^{19}F NMR (376 MHz, $CDCl_3$) δ -62.64.
HRMS (EI) *m/z*: calculated for $C_{10}H_6F_3NO$ [M^+]: 213.0401, found: 213.0409

6-Methoxyquinoline *N*-oxide **3d**

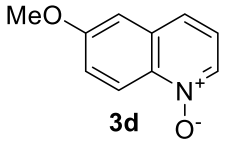

3d

The title compound was prepared from 6-methoxyquinoline (0.55 mL, 4.0 mmol) according to the general procedure A. The product was purified with flash chromatography (silica gel, EtOAc/MeOH 10:1) and obtained as a white solid (636.0 mg, 91%).

^{1}H NMR (400 MHz, $CDCl_3$) δ 8.66 (d, *J* = 9.5 Hz, 1H), 8.39 (dd, *J* = 6.0, 1.0 Hz, 1H), 7.62 (d, *J* = 8.5 Hz, 1H), 7.38 (dd, *J* = 9.6, 2.7 Hz, 1H), 7.24 (dd, *J* = 8.5, 6.0 Hz, 1H), 7.11 (d, *J* = 2.7 Hz, 1H), 3.94 (s, 3H).
^{13}C NMR (101 MHz, $CDCl_3$) δ 159.6, 137.3, 134.0, 132.1, 125.3, 123.0, 121.6, 121.5, 105.9, 55.8.
The characterization data was in agreement with the previously published data.[13]

6-Chloroquinoline *N*-oxide **3e**

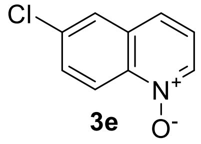

3e

The title compound was prepared from 6-chloroquinoline (670.6 mg, 4.1 mmol) according to the general procedure A. The product was purified with flash chromatography (silica gel, EtOAc/MeOH 10:1) and obtained as a white solid (651.0 mg 88%).

^{1}H NMR (400 MHz, $CDCl_3$) δ 8.71 (d, *J* = 9.3 Hz, 1H), 8.50 (dd, *J* = 6.1, 1.0 Hz, 1H), 7.86 (d, *J* = 2.2 Hz, 1H), 7.69 (dd, *J* = 9.3, 2.2 Hz, 1H), 7.65 (d, *J* = 8.5 Hz, 1H), 7.33 (dd, *J* = 8.5, 6.0 Hz, 1H).
^{13}C NMR (101 MHz, $CDCl_3$) δ 140.2, 135.7, 135.1, 131.3, 131.2, 126.9, 124.7, 122.4, 121.9.
The characterization data was in agreement with the previously published data.[4]

6-(Methoxycarbonyl)quinoline *N*-oxide **3f**

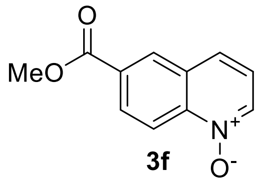

3f

The title compound was prepared according to the general procedure A. The product was purified with flash chromatography (silica gel, DCM/MeOH 20:1) and obtained as an off-white solid (650 mg, 60%).

^{1}H NMR (400 MHz, $CDCl_3$) δ 8.81 (d, *J* = 9.1 Hz, 1H), 8.63 (d, *J* = 1.8 Hz, 1H), 8.60 (dd, *J* = 6.1, 1.0 Hz, 1H), 8.34 (dd, *J* = 9.1, 1.8 Hz, 1H), 7.84 (d, *J* = 8.6 Hz, 1H), 7.38 (dd, *J* = 8.5, 6.1 Hz, 1H), 4.02 (s, 3H).
^{13}C NMR (101 MHz, $CDCl_3$) δ 165.9, 143.3, 137.3, 131.1, 130.6, 130.1, 130.0, 126.9, 122.0, 120.5, 52.8.
The characterization data was in agreement with the previously published data.[13]

6-Nitroquinoline *N*-oxide **3g**

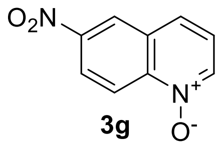

3g

The title compound was prepared from 6-nitroquinoline (584.4 mg, 3.4 mmol) according to the general procedure A. The product was purified with flash chromatography (silica gel, EtOAc/MeOH 10:1) and obtained as a yellow solid (540.0 mg, 84%).

^{1}H NMR (400 MHz, DMSO-d_6) δ 9.14 (d, *J* = 2.5 Hz, 1H), 8.77 (dd, *J* = 6.1, 1.0 Hz, 1H), 8.69 (d, *J* = 9.5 Hz, 1H), 8.46 (dd, *J* = 9.5, 2.5 Hz, 1H), 8.23 (d, *J* = 8.5 Hz, 1H), 7.66 (dd, *J* = 8.5, 6.1 Hz, 1H).
^{13}C NMR (101 MHz, DMSO-d_6) δ 146.7, 142.7, 138.0, 129.9, 126.6, 125.5, 124.1, 123.3, 121.4.

The characterization data was in agreement with the previously published data.[13]

4-Methylquinoline *N*-oxide **3h**

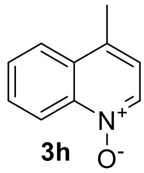
3h

The title compound was prepared from 4-methylquinoline (0.53 mL, 4.0 mmol) according to the general procedure A. The product was purified with flash chromatography (silica gel, EtOAc/MeOH 10:1) and obtained as a light brown solid (524.8 mg, 82%).

^{1}H NMR (400 MHz, $CDCl_3$) δ 8.82 (d, J = 8.8 Hz, 1H), 8.44 (d, J = 6.1 Hz, 1H), 7.98 (dd, J = 8.4, 1.8 Hz, 1H), 7.81–7.75 (m, 1H), 7.71–7.66 (m, 1H), 7.13 (d, J = 6.1 Hz, 1H), 2.67 (s, 3H).
^{13}C NMR (101 MHz, $CDCl_3$) δ 141.0, 135.2, 135.1, 130.3, 130.0, 128.6, 124.9, 121.5, 120.4, 18.5.
The characterization data was in agreement with the previously published data.[13]

4-(1,3-Dioxolan-2-yl)quinoline *N*-oxide **3i**

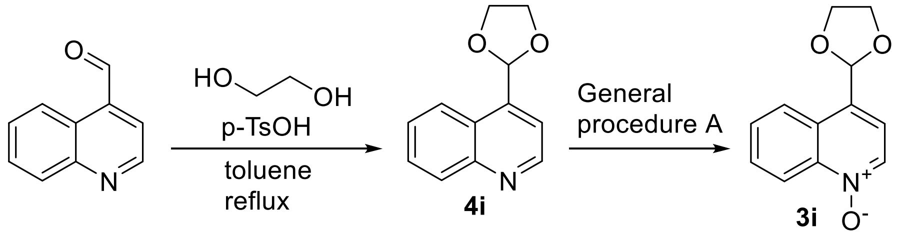

Compound **4i** was prepared according to a literature procedure[14] from quinoline-4-carboxaldehyde (628.6 mg, 4.0 mmol), ethylene glycol (0.88 mL, 15.8 mmol) and *p*-TsOH•H_2O (85.8 mg, 0.45 mmol) by refluxing in toluene (20 mL) in an oil bath. The crude product was used without purification in the next step.

The title compound was prepared according to the general procedure A from the crude product prepared in the previous step. The product was purified with flash chromatography (silica gel, EtOAc/8% MeOH) and obtained as a white solid (703.8 mg, 81% yield over two steps).

^{1}H NMR (400 MHz, $CDCl_3$) δ 8.80 (dd, J = 8.8, 1.4 Hz, 1H), 8.51 (d, J = 6.3 Hz, 1H), 8.23 (dd, J = 8.5, 0.7 Hz, 1H), 7.83–7.74 (m, 1H), 7.74–7.65 (m, 1H), 7.55 (d, J = 6.3 Hz, 1H), 6.37 (s, 1H), 4.17 (m, 4H).
^{13}C NMR (101 MHz, $CDCl_3$) δ 141.7, 135.0, 132.6, 130.3, 129.1, 128.3, 125.0, 120.4, 118.4, 100.4, 65.7.
The characterization data was in agreement with the previously published data.[15]

4,7-Dichloroquinoline *N*-oxide **3j**

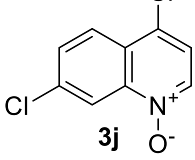
3j

The title compound was prepared from 4,7-dichloroquinoline (823.0 mg, 4.2 mmol) according to the general procedure A. The product was purified with flash chromatography (silica gel, EtOAc/MeOH 10:1) and obtained as a white solid (839.0 mg, 94%).

^{1}H NMR (400 MHz, $CDCl_3$) δ 8.80 (d, J = 2.1 Hz, 1H), 8.43 (d, J = 6.6 Hz, 1H), 8.16 (d, J = 9.0 Hz, 1H), 7.71 (dd, J = 9.0, 2.1 Hz, 1H), 7.37 (d, J = 6.6 Hz, 1H).
^{13}C NMR (101 MHz, $CDCl_3$) δ 142.6, 138.3, 136.0, 130.9, 129.8, 126.9, 126.7, 121.4, 120.1.
The characterization data was in agreement with the previously published data.[13]

8-Methylquinoline *N*-oxide **3k**

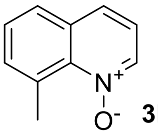

3k

The title compound was prepared from 8-methylquinoline (0.28 mL, 2.1 mmol) according to the general procedure A. The product was purified with flash chromatography (silica gel, EtOAc/MeOH 10:1) and obtained as a brown oil (195.0 mg, 58%).

^{1}H NMR (400 MHz, $CDCl_3$) δ 8.40 (d, *J* = 6.0 Hz, 1H), 7.64 (d, *J* = 9.6 Hz, 2H), 7.46–7.38 (m, 2H), 7.18 (dd, *J* = 8.5, 6.0 Hz, 1H), 3.19 (s, 3H).
^{13}C NMR (101 MHz, $CDCl_3$) δ 141.4, 137.4, 133.7, 133.5, 132.5, 128.2, 126.9, 126.8, 120.7, 25.0.
The characterization data was in agreement with the previously published data.[5]

2-Phenylquinoline *N*-oxide **3l**

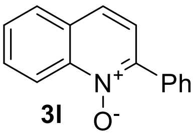

3l

The title compound was prepared from 2-phenylquinoline (411.2 mg, 2.0 mmol) according to the general procedure A. The product was purified with flash chromatography (silica gel, EtOAc/8% MeOH) and obtained as a light brown solid (261.5 mg, 59%).

^{1}H NMR (400 MHz, $CDCl_3$) δ 8.87 (d, *J* = 8.8 Hz, 1H), 8.00–7.95 (m, 2H), 7.87 (d, *J* = 8.1 Hz, 1H), 7.82–7.75 (m, 2H), 7.69–7.61 (m, 1H), 7.56–7.45 (m, 4H).
^{13}C NMR (101 MHz, $CDCl_3$) δ 145.1, 142.4, 133.6, 130.7, 129.7 (2C), 129.6, 128.5, 128.4, 128.1, 125.4, 123.4, 120.4.
The characterization data was in agreement with the previously published data.[4]

Benzo[*f*]quinoline *N*-oxide **3m**

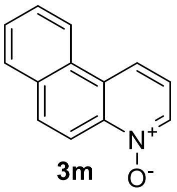

3m

The title compound was prepared from benzo[*f*]quinoline (717.4 mg, 4.0 mmol) according to the general procedure A. The product was purified with flash chromatography (silica gel, EtOAc/8% MeOH) and obtained as a white solid (695.5 mg, 89%).

^{1}H NMR (400 MHz, $CDCl_3$) δ 8.74 (d, *J* = 9.5 Hz, 1H), 8.64–8.59 (m, 2H), 8.53 (d, *J* = 8.9 Hz, 1H), 8.06 (d, *J* = 9.5 Hz, 1H), 8.00–7.97 (m, 1H), 7.79–7.71 (m, 2H), 7.48 (dd, *J* = 8.6, 6.2 Hz, 1H).
^{13}C NMR (101 MHz, $CDCl_3$) δ 141.2, 136.3, 132.3, 132.2, 129.2, 129.0, 128.7, 128.6, 128.1, 123.4, 121.5, 120.8, 117.3.
The characterization data was in agreement with the previously published data.[16]

Isoquinoline *N*-oxide **3n**

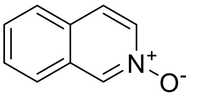

3n

The title compound was prepared from isoquinoline (0.70 mL, 5.9 mmol) according to the general procedure A. The product was purified with flash chromatography (silica gel, EtOAc/MeOH 10:1) and obtained as an off-white solid (657.0 mg, 77%).

^{1}H NMR (400 MHz, $CDCl_3$) δ 8.78 (s, 1H), 8.15 (dd, *J* = 7.1, 1.8 Hz, 1H), 7.82–7.78 (m, 1H), 7.76–7.72 (m, 1H), 7.68 (d, *J* = 7.1 Hz, 1H), 7.66–7.57 (m, 2H).
^{13}C NMR (101 MHz, $CDCl_3$) δ 136.8, 136.3, 129.7, 129.6, 129.2, 129.0, 126.8, 125.1, 124.4.
The characterization data was in agreement with the previously published data.[13]

5-Nitroisoquinoline **3o**

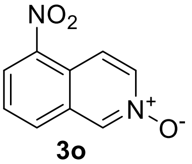

3o

The title compound was prepared from 5-nitroisoquinoline (697.4 mg, 4.0 mmol) according to the general procedure A. The product was purified with flash chromatography (silica gel, EtOAc/8% MeOH) and obtained as a light brown solid (114.2 mg, 15%).

^{1}H NMR (400 MHz, $CDCl_3$) δ 8.87 (s, 1H), 8.61 (d, *J* = 7.6 Hz, 1H), 8.42 (d, *J* = 7.9 Hz, 1H), 8.32 (d, *J* = 7.6 Hz, 1H), 8.02 (d, *J* = 8.3 Hz, 1H), 7.75 (t, *J* = 8.0 Hz, 1H).
^{13}C NMR (101 MHz, $CDCl_3$) δ 145.3, 140.0, 136.1, 131.5, 131.2, 128.7, 126.7, 121.0, 120.6.
HRMS (EI) *m/z*: calculated for $C_9H_6N_2O_3$ [M^+]: 190.0378, found: 190.0382

Quinoxaline 1,4-dioxide **3q**

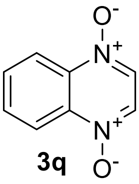

3q

The title compound was prepared according to the general procedure A from quinoxaline (587.4 mg, 4.5 mmol) using 2.5 equiv of *m*-CPBA. The product was purified with flash chromatography (EtOAc 100% → EtOAc/8% MeOH) and obtained as yellow crystals (310.4 mg, 42%).

^{1}H NMR (400 MHz, $CDCl_3$) δ 8.65–8.56 (m, 2H), 8.23 (s, 2H), 7.93–7.85 (m, 2H).
^{13}C NMR (101 MHz, $CDCl_3$) δ 138.7, 132.3, 130.6, 120.7.
The characterization data was in agreement with the previously published data.[17]

5-Nitroquinoline *N*-oxide **S9**

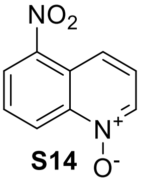

S14

The title compound was prepared from 5-nitroquinoline (896.5 mg, 5.1 mmol) according to the general procedure A. The product was purified with flash chromatography (silica gel, EtOAc/8% MeOH) and obtained as a yellow solid (919.6 mg, 94%).

^{1}H NMR (400 MHz, $CDCl_3$) δ 9.15 (d, *J* = 8.8 Hz, 1H), 8.61 (d, *J* = 6.0 Hz, 1H), 8.50 (d, *J* = 9.1 Hz, 1H), 8.45 (dd, *J* = 7.7, 1.3 Hz, 1H), 7.86 (dd, *J* = 8.8, 7.7 Hz, 1H), 7.53 (dd, *J* = 9.0, 6.1 Hz, 1H).
^{13}C NMR (101 MHz, $CDCl_3$) δ 146.2, 142.7, 136.4, 128.5, 126.8, 126.6, 124.2, 124.1, 121.0.
The characterization data was in agreement with the previously published data.[18]

Preparation of diphenylnitrone **5**

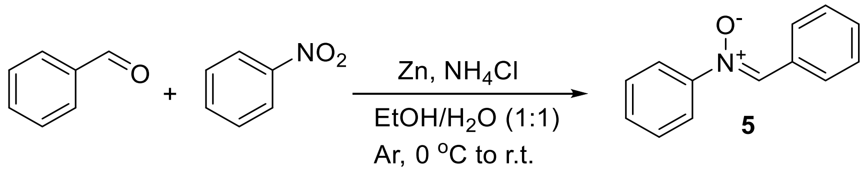

The title compound was prepared according to a literature procedure.[19] Benzaldehyde (1.0 mL, 9.8 mmol) was dissolved to EtOH/H_2O (1:1, 26 mL), and the solution was placed under argon. Nitrobenzene (1.0 mL, 9.7 mmol) and NH_4Cl (680.9 mg, 12.7 mmol) were added to the solution, and the mixture was cooled down to 0 ˚C. Zn (1.27 g, 19.4 mmol) was added in small portions, and the reaction mixture was stirred at 0 ˚C for 1 h after which it was allowed to warm to r.t. After 24 h, the reaction mixture was diluted with EtOAc and filtered through a pad of Celite, and the phases were separated. The aqueous phase was extracted with EtOAc (2×15 mL), the combined organic phases were washed with brine (1×50 mL) and dried over Na_2SO_4. The drying agent was filtered off, and the solution was concentrated on a rotary evaporator. The product was purified by crystallization from DCM/pentane and obtained as off-white needles (741 mg, 39 %).

^{1}H NMR (400 MHz, $CDCl_3$) δ 8.43–8.38 (m, 2H), 7.92 (s, 1H), 7.80–7.75 (m, 2H), 7.52–7.46 (m, 6H).
^{13}C NMR (101 MHz, $CDCl_3$) δ 149.3, 134.7, 131.1, 130.8, 130.1, 129.3, 129.2, 128.8, 121.9.
The characterization data was in agreement with the previously published data.[19]

Optimization of reaction conditions

General screening procedure

Alcohol, *N*-oxide **1a** (0.1 mmol), and PQ were weighted to a vial equipped with a stirring bar. Solvent was added, the vial was sealed with a septum, and the reaction mixture was bubbled with argon for 20 minutes. After this, the reaction mixture was stirred and irradiated with blue LEDs (450 nm) at 30 ˚C under argon atmosphere for 4 h. After LED irradiation, NMR yields were determined from ^{1}H spectra (in $CDCl_3$) using 1,3,5-trimethoxybenzene as an internal standard.

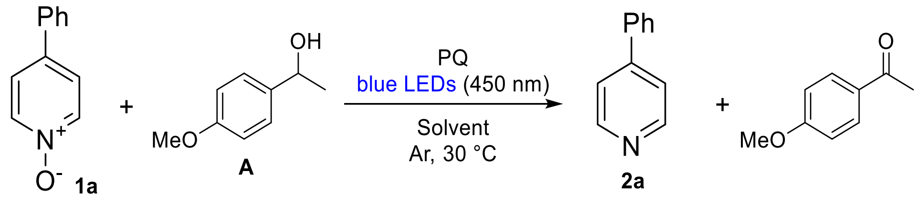

Table S1: Solvent screening. Conditions: 0.1 mmol of **1a**, 1.5 equiv of alcohol **A**, PQ (20 mol%), solvent specified (0.05 M), argon atmosphere, 450 nm LEDs, and 30 ˚C. Reaction time 4 h. [a] Reaction time 1 h.

Entry	Solvent	Unreacted SM (%)	Yield of product (%)
**1**	**MeCN/0.1% H_2O**	**4**	**94**
2	MeCN	3	93
3	MeCN (over 4Å sieves)	3	91
4	DCM	8	84
5	EtOAc	4	83
6	Toluene	29	66
7	DMSO	59	24
8	THF	58	20
9	Acetone [a]	6	88
10	DMC [a]	3	89

Table S2: Water content in MeCN. Conditions: 0.1 mmol of **1a**, 1.5 equiv of alcohol **A**, PQ (20 mol%), solvent specified (0.05 M), argon atmosphere, 450 nm LEDs, and 30 ˚C. Reaction time 4 h.

Entry	Solvent	Unreacted SM (%)	Yield of product (%)
1	MeCN (over 4Å sieves)	3	91
2	MeCN (ambient)	3	93
**3**	**MeCN/0.1% H_2O**	**4**	**94**
4	MeCN/0.5% H_2O	7	90
5	MeCN/1.0% H_2O	30	65

Table S3: Concentration screening. Conditions: 0.1 mmol of **1a**, 1.5 equiv of alcohol **A**, PQ (20 mol%), MeCN/0.1% H_2O (concentration specified), argon atmosphere, 450 nm LEDs, and 30 ˚C. Reaction time 4 h.

Entry	Concentration	Unreacted SM (%)	Yield of product (%)
1	0.1	3	89
2	0.067	3	91
**3**	**0.05**	**4**	**94**
4	0.04	3	89
5	0.033	3	89
6	0.025	3	91

Table S4: Catalyst and catalyst loading screening. Conditions: 0.1 mmol of **1a**, 1.5 equiv of alcohol **A**, catalyst specified, MeCN/0.1% H_2O (0.05M), argon atmosphere, 450 nm LEDs, and 30 ˚C. Reaction time 4 h. [a] *i*-PrOH (3.0 equiv) was used as the hydrogen source.

Entry	Catalyst (loading)	Unreacted SM (%)	Yield of product (%)
1	PQ (20 mol%)	4	94
2	PQ (15 mol%)	5	93
3	PQ-OMe (20 mol%)	9	89
4	PQ-OMe (15 mol%)	14	83
5	PQ-CF_3 (20 mol%)	0	98
6	PQ-CF_3 (15 mol%)	0	97
7	PQ-CF_3 (20 mol%) [a]	0	94
8	PQ-CF_3 (15 mol%) [a]	0	94
9	PQ-CF_3 (10 mol%) [a]	0	93
10	PQ-CF_3 (5 mol%) [a]	33	62

Table S5: Hydrogen source screening. Conditions: 0.1 mmol of **1a**, hydrogen source specified, PQ (15 mol%), MeCN/0.1% H_2O (0.05 M), argon atmosphere, 450 nm LEDs, and 30 ˚C. Reaction time 4 h. [a] PQ-CF_3 (15 mol%) used as the catalyst.

Entry	Hydrogen source	Unreacted SM (%)	Yield of product (%)
1	DTT (1.5 equiv)	1	97
2	*i*-PrOH (3.0 equiv)	17	78
3	1-Me-1,4-CHD (3.0 equiv)	10	84
4	DTT (1.5 equiv) [a]	0	97
5	*i*-PrOH (3.0 equiv) [a]	0	94
6	1-Me-1,4-CHD (3.0 equiv) [a]	0	93

Table S6: Control tests. Conditions: 0.1 mmol of **1a**, 1.5 equiv of alcohol **A**, PQ-CF_3 (15 mol%), MeCN/0.1% H_2O (0.05 M), argon atmosphere, 450 nm LEDs, and 30 ˚C. Reaction time 4 h.

Entry	Variation	Unreacted SM (%)	Yield of product (%)
1	under air	23	71
2	in dark	>99	0
3	no catalyst	>99	0
4	no hydrogen source	89	7
5	no hydrogen source, in dry MeCN	86	4
6	no hydrogen source, in dry CD_3CN	86	2
7	no hydrogen source, in CD_3CN/0.1% H_2O	84	2

Scope of the reaction

General procedure B: Photocatalytic deoxygenation of pyridines

Pyridine derivative *N*-oxide (0.20 mmol, 1.0 equiv) and PQ-CF_3 (15–20 mol%) were weighted to a vial equipped with a stirring bar. *i*-PrOH (46.0 µL, 0.60 mmol, 3.0 equiv) and MeCN/0.1% H_2O (4.0 mL, 0.05 M) were added, and the vial was sealed with a septum. The reaction mixture was bubbled with argon for 30 min. After this, the reaction mixture was stirred and irradiated with blue LEDs at 30 ˚C under argon for the reaction time specified. NMR yield was determined from ^{1}H spectrum (in $CDCl_3$) using 1,3,5-trimethoxybenzene as an internal standard. For isolated yield, the reaction mixture was concentrated on a rotary evaporator and the residue was purified with flash chromatography.

General procedure C: Photocatalytic deoxygenation of quinolines

Quinoline derivative *N*-oxide (0.20 mmol, 1.0 equiv), DTT (1.2–2.1 equiv) and PQ (15–25 mol%) were weighted to a vial equipped with a stirring bar. MeCN/0.1% H_2O (4.0 mL, 0.05 M) was added, the vial was sealed with a septum, and the reaction mixture was bubbled with argon for 30 min. After this, the reaction mixture was stirred and irradiated with blue LEDs at 30 ˚C under argon for the reaction time specified. NMR yield was determined from ^{1}H spectrum (in $CDCl_3$) using 1,3,5-trimethoxybenzene as an internal standard. For isolated yield, the reaction mixture was concentrated on a rotary evaporator and the residue was purified with flash chromatography.

Characterization of products

4-Phenylpyridine **2a**

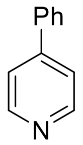

2a

The title compound was prepared according to the general procedure B from 4-phenylpyridine *N*-oxide **1a** (35.6 mg, 0.20 mmol) using *i*-PrOH (46.0 µL, 0.60 mmol) and PQ-CF_3 (10.4 mg, 0.030 mmol) with the reaction time of 6 h. The product was isolated with flash chromatography (silica gel, pentane/acetone 5:1 /0.5% TEA) and obtained as a white solid (26.6 mg, 86%).

^{1}H NMR (400 MHz, $CDCl_3$) δ 8.68–8.65 (m, 2H), 7.66–7.62 (m, 2H), 7.52–7.42 (m, 5H).
^{13}C NMR (101 MHz, $CDCl_3$) δ 150.4, 148.5, 138.3, 129.3, 129.2, 127.1, 121.8.
The characterization data was in agreement with the previously published data.[4]

Methyl isonicotinate **2b**

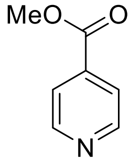

2b

The title compound was prepared according to the general procedure B from 4-(methoxycarbonyl)pyridine *N*-oxide (31.0 mg, 0.20 mmol) using *i*-PrOH (46.0 µL, 0.60 mmol) and PQ-CF_3 (10.4 mg, 0.030 mmol) with the reaction time of 16 h. The product was isolated with flash chromatography (silica gel, pentane/acetone 10:1 /0.5% TEA) and obtained as a pale yellow oil (24.1 mg, 87%).

^{1}H NMR (400 MHz, $CDCl_3$) δ 8.79 (d, *J* = 6.0 Hz, 2H), 7.85 (d, *J* = 6.0 Hz, 2H), 3.97 (s, 3H).
^{13}C NMR (101 MHz, $CDCl_3$) δ 165.7, 150.8, 137.4, 123.0, 52.8.
The characterization data was in agreement with the previously published data.[20]

4-Cyanopyridine **2c**

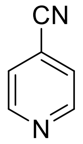

2c

The title compound was prepared according to the general procedure B from 4-cyanopyridine *N*-oxide **1c** (24.2 mg, 0.20 mmol) using *i*-PrOH (46.0 µL, 0.60 mmol) and PQ-CF_3 (13.7 mg, 0.040 mmol) with the reaction time of 16 h. The product was isolated with flash chromatography (silica gel, pentane/acetone 10:1 → 5:1 /0.5% TEA) and obtained as a white solid (15.5 mg, 74%).

^{1}H NMR (400 MHz, $CDCl_3$) δ 8.83 (d, *J* = 6.1 Hz, 1H), 7.54 (d, *J* = 6.0 Hz, 1H).
^{13}C NMR (101 MHz, $CDCl_3$) δ 150.9, 125.4, 120.6, 116.5.
The characterization data was in agreement with the previously published data.[21]

4-Methoxypyridine **2d**

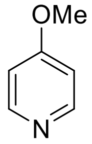

2d

The title compound was prepared according to the general procedure B from 4-methoxypyridine *N*-oxide hydrate (87 w%, 28.7 mg, 0.20 mmol) using *i*-PrOH (46.0 µL, 0.60 mmol) and PQ-CF_3 (10.4 mg, 0.030 mmol) with the reaction time of 16 h. The product was isolated with flash chromatography (silica gel, pentane/acetone 10:1 → 3:1 /0.5% TEA) and obtained as a yellow oil (16.9 mg, 81%).

^{1}H NMR (400 MHz, $CDCl_3$) δ 8.43 (dd, *J* = 4.8, 1.6 Hz, 2H), 6.82 (dd, *J* = 4.8, 1.6 Hz, 2H), 3.85 (s, 3H).
^{13}C NMR (101 MHz, $CDCl_3$) δ 165.8, 151.1, 110.0, 55.2.
The characterization data was in agreement with the previously published data.[22]

4-(Benzyloxy)pyridine **2e**

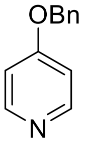

2e

The title compound was prepared according to the general procedure B from 4-(benzyloxy)pyridine *N*-oxide **1e** (40.3 mg, 0.20 mmol) using *i*-PrOH (46.0 µL, 0.60 mmol) and PQ-CF_3 (13.7 mg, 0.040 mmol) with the reaction time of 16 h. The product was isolated with flash chromatography (silica gel, *n*-Hex/EtOAc 3:1 → 1:1 /0.5% TEA) and obtained as a yellow oil (27.2 mg, 73%).

^{1}H NMR (400 MHz, $CDCl_3$) δ 8.46–8.41 (m, 2H), 7.44–7.33 (m, 5H), 6.90–6.85 (m, 2H), 5.11 (s, 2H).
^{13}C NMR (101 MHz, $CDCl_3$) δ 164.8, 151.3, 135.8, 128.9, 128.5, 127.7, 110.7, 69.9.
The characterization data was in agreement with the previously published data.[23]

3-(Benzoyloxy)pyridine **2f**

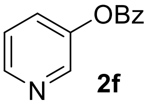

2f

The title compound was prepared according to the general procedure B from 4-(benzoyloxy)pyridine *N*-oxide **1f** (43.1 mg, 0.20 mmol) using *i*-PrOH (46.0 µL, 0.60 mmol) and PQ-CF_3 (10.4 mg, 0.030 mmol) with the reaction time of 16 h. The product was isolated with flash chromatography (silica gel, *n*-Hex/EtOAc 10:1 → 5:1 /0.5% TEA) and obtained as a white solid (23.8 mg, 60%).

^{1}H NMR (400 MHz, $CDCl_3$) δ 8.57 (d, *J* = 2.7 Hz, 1H), 8.54 (dd, *J* = 4.7, 1.5 Hz, 1H), 8.24–8.19 (m, 2H), 7.70–7.64 (m, 1H), 7.62 (ddd, *J* = 8.4, 2.8, 1.4 Hz, 1H), 7.58–7.50 (m, 2H), 7.39 (dd, *J* = 8.4, 4.8 Hz, 1H).
^{13}C NMR (101 MHz, $CDCl_3$) δ 164.8, 147.8, 147.2, 143.8, 134.2, 130.4, 129.5, 128.93, 128.86, 124.1.
The characterization data was in agreement with the previously published data.[24]

3-((Benzoyloxy)methyl)pyridine **2g**

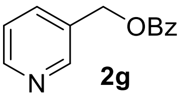
2g

The title compound was prepared according to the general procedure B from 3-((benzoyloxy)methyl)pyridine *N*-oxide **1g** (46.8 mg, 0.20 mmol) using *i*-PrOH (46.0 µL, 0.60 mmol) and PQ-CF_3 (13.7 mg, 0.040 mmol) with the reaction time of 16 h. The product was isolated with flash chromatography (silica gel, *n*-Hex/EtOAc 3:1/0.5% TEA) and obtained as a yellow oil (35.2 mg, 81%).

^{1}H NMR (400 MHz, $CDCl_3$) δ 8.73 (d, *J* = 1.5 Hz, 1H), 8.60 (dd, *J* = 4.8, 1.7 Hz, 1H), 8.09–8.03 (m, 2H), 7.83–7.75 (m, 2H), 7.61–7.55 (m, 2H), 7.48–7.42 (m, 2H), 7.33 (ddd, *J* = 7.9, 4.9, 1.0 Hz, 2H), 5.39 (s, 2H).
^{13}C NMR (101 MHz, $CDCl_3$) δ 166.4, 149.9, 136.1, 133.4, 131.8, 129.8, 128.6, 123.6, 64.3.
The characterization data was in agreement with the previously published data.[25]

3,5-Dibromopyridine **2h**

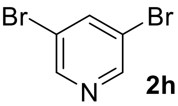
2h

The title compound was prepared according to the general procedure B from 3,5-dibromopyridine *N*-oxide (50.5 mg, 0.20 mmol) using *i*-PrOH (46.0 µL, 0.60 mmol) and PQ-CF_3 (10.4 mg, 0.030 mmol) with the reaction time of 16 h. The product was isolated with flash chromatography (silica gel, *n*-Hex/acetone 80:1/0.5% TEA) and obtained as a white solid (28.4 mg, 60%).

^{1}H NMR (400 MHz, $CDCl_3$) δ 8.61 (d, *J* = 2.0 Hz, 2H), 8.01 (t, *J* = 2.0 Hz, 1H).
^{13}C NMR (101 MHz, $CDCl_3$) δ 149.3, 141.1, 121.0.
The characterization data was in agreement with the previously published data.[26]

3,5-Dichloropyridine **2i**

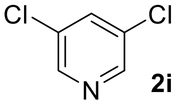
2i

The title compound was prepared according to the general procedure B from 3,5-dichloropyridine *N*-oxide (32.4 mg, 0.20 mmol) using *i*-PrOH (46.0 µL, 0.60 mmol) and PQ-CF_3 (10.4 mg, 0.030 mmol) with the reaction time of 16 h. The product was isolated with flash chromatography (silica gel, pentane/Et_2O 60:1 → 40:1 /0.5% TEA) and obtained as white needles (14.0 mg, 48%).

^{1}H NMR (400 MHz, $CDCl_3$) δ 8.48 (d, *J* = 2.1 Hz, 2H), 7.71 (t, *J* = 2.1 Hz, 1H).
^{13}C NMR (101 MHz, $CDCl_3$) δ 146.9, 135.7, 132.4.
The characterization data was in agreement with the previously published data.[27]

2,6-Dichloropyridine **2j**

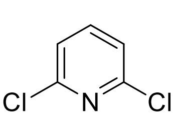
2j

The title compound was prepared according to the general procedure B from 2,6-dichloropyridine *N*-oxide (33.8 mg, 0.20 mmol) using *i*-PrOH (46.0 µL, 0.60 mmol) and PQ-CF_3 (10.4 mg, 0.030 mmol) with the reaction time of 16 h. The product was isolated with flash chromatography (silica gel, pentane/Et_2O 80:1 → 40:1 /0.5% TEA) and obtained as white needles (16.6 mg, 56%).

^{1}H NMR (400 MHz, $CDCl_3$) δ 7.62 (t, *J* = 7.8 Hz, 1H), 7.28 (d, *J* = 7.8 Hz, 2H).
^{13}C NMR (101 MHz, $CDCl_3$) δ 150.8, 140.8, 123.0.
The characterization data was in agreement with the previously published data.[28]

5-Cyano-2-methylpyridine **2k**

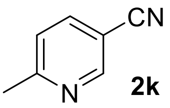

2k

The title compound was prepared according to the general procedure B from 5-cyano-2-methylpyridine *N*-oxide **1k** (26.4 mg, 0.20 mmol) using *i*-PrOH (46.0 µL, 0.60 mmol) and PQ-CF_3 (10.4 mg, 0.030 mmol) with the reaction time of 16 h. The product was isolated with flash chromatography (silica gel, pentane/acetone 10:1 → 5:1 /0.5% TEA) and obtained as a yellow oil (16.1 mg, 69%).

^{1}H NMR (400 MHz, $CDCl_3$) δ 8.78 (d, *J* = 2.4 Hz, 1H), 7.84 (dd, *J* = 8.1, 2.2 Hz, 1H), 7.29 (d, *J* = 8.0 Hz, 1H), 2.65 (s, 3H).
^{13}C NMR (101 MHz, $CDCl_3$) δ 163.3, 152.2, 139.3, 123.5, 117.1, 107.2, 25.1.
HRMS (EI) *m/z*: calculated for $C_7H_6N_2$ [M^+]: 118.0531, found: 118.0534
The characterization data was in agreement with the previously published data.[29]

Quinoline **4a**

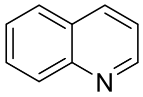

4a

The title compound was prepared according to the general procedure C from quinoline *N*-oxide **3a** (28.8 mg, 0.20 mmol) using DTT (37.0 mg, 0.24 mmol) and PQ (6.2 mg, 0.030 mmol) with the reaction time of 1 h. The product was isolated with flash chromatography (silica gel, *n*-Hex/EtOAc 10:1/0.5% TEA) and obtained as a yellow oil (18.1 mg, 70%).

^{1}H NMR (400 MHz, $CDCl_3$) δ 8.93 (dd, *J* = 4.2, 1.8 Hz, 1H), 8.16 (d, *J* = 8.4 Hz, 1H), 8.12 (d, *J* = 8.5 Hz, 1H), 7.82 (d, *J* = 8.1 Hz, 1H), 7.75–7.70 (m, 1H), 7.57–7.52 (m, 1H), 7.40 (dd, *J* = 8.3, 4.2 Hz, 1H).
^{13}C NMR (101 MHz, $CDCl_3$) δ 150.6, 148.4, 136.2, 129.60, 129.58, 128.4, 127.9, 126.7, 121.2.
The characterization data was in agreement with the previously published data.[4]

3-Bromoquinoline **4b**

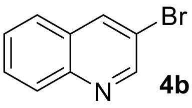

4b

The title compound was prepared according to the general procedure C from 3-bromoquinoline *N*-oxide **3b** (45.7 mg, 0.20 mmol) using DTT (37.0 mg, 0.24 mmol) and PQ (6.2 mg, 0.030 mmol) with the reaction time of 1 h. The product was isolated with flash chromatography (silica gel, *n*-Hex/EtOAc 20:1 → 5:1 /0.5% TEA) and obtained as a clear oil (31.5 mg, 76%).

^{1}H NMR (400 MHz, $CDCl_3$) δ 8.91 (d, *J* = 2.3 Hz, 1H), 8.32 (d, *J* = 2.3 Hz, 1H), 8.09 (d, *J* = 8.4 Hz, 1H), 7.77–7.71 (m, 2H), 7.61–7.56 (m, 1H).
^{13}C NMR (101 MHz, $CDCl_3$) δ 151.5, 146.5, 137.4, 129.9, 129.7, 129.2, 127.8, 127.1, 117.3.
The characterization data was in agreement with the previously published data.[30]

3-(Trifluoromethyl)quinoline **4c**

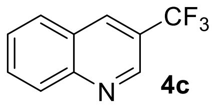

4c

The title compound was prepared according to the general procedure C from 3-(trifluoromethyl)quinoline *N*-oxide **3c** (42.9 mg, 0.20 mmol) using DTT (37.0 mg, 0.24 mmol) and PQ (6.2 mg, 0.030 mmol) with the reaction time of 1 h. The product was isolated with flash chromatography (silica gel, *n*-Hex/EtOAc 20:1 → 5:1 /0.5% TEA) and obtained as a white solid (29.5 mg, 75%).

^{1}H NMR (400 MHz, $CDCl_3$) δ 9.11 (d, *J* = 2.3 Hz, 1H), 8.46 (s, 1H), 8.20 (d, *J* = 8.5 Hz, 1H), 7.94 (d, *J* = 8.1 Hz, 1H), 7.90–7.84 (m, 1H), 7.71–7.63 (m, 1H).
^{13}C NMR (101 MHz, $CDCl_3$) δ 149.5, 146.2 (q, *J* = 3.3 Hz,), 134.1 (q, J = 4.0 Hz), 131.9, 129.8, 128.8, 128.2, 126.4, 123.83 (q, J = 272.3 Hz), 123.76 (q, *J* = 32.9 Hz).
^{19}F NMR (376 MHz, $CDCl_3$) δ -61.80.
The characterization data was in agreement with the previously published data.[31]

6-Methoxyquinoline **4d**

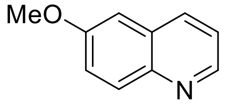

4d

The title compound was prepared according to the general procedure C from 6-methoxyquinoline *N*-oxide **3d** (35.1 mg, 0.20 mmol) using DTT (37.0 mg, 0.24 mmol) and PQ (6.2 mg, 0.030 mmol) with the reaction time of 2 h. The product was isolated with flash chromatography (silica gel, *n*-Hex/EtOAc 20:1 → 5:1 /0.5% TEA) and obtained as a yellow oil (16.4 mg, 52%).

^{1}H NMR (400 MHz, $CDCl_3$) δ 8.77 (dd, *J* = 4.3, 1.7 Hz, 1H), 8.05 (dd, *J* = 8.4, 0.9 Hz, 1H), 8.00 (d, *J* = 9.3 Hz, 1H), 7.40–7.33 (m, 2H), 7.07 (d, *J* = 2.8 Hz, 1H), 3.94 (s, 3H).
^{13}C NMR (101 MHz, $CDCl_3$) δ 157.9, 148.1, 144.6, 134.9, 131.0, 129.4, 122.4, 121.5, 105.3, 55.7.
The characterization data was in agreement with the previously published data.[13]

6-Chloroquinoline **4e**

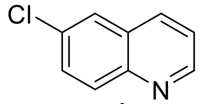

4e

The title compound was prepared according to the general procedure C from 6-chloroquinoline *N*-oxide **3e** (36.1 mg, 0.20 mmol) using DTT (37.0 mg, 0.24 mmol) and PQ (6.2 mg, 0.030 mmol) with the reaction time of 1 h. The product was isolated with flash chromatography (silica gel, *n*-Hex/EtOAc 20:1 → 5:1 /0.5% TEA) and obtained as a pale yellow solid (25.5 mg, 78%).

^{1}H NMR (400 MHz, $CDCl_3$) δ 8.91 (dd, *J* = 4.2, 1.7 Hz, 1H), 8.10–8.03 (m, 2H), 7.81 (d, *J* = 2.4 Hz, 1H), 7.66 (dd, *J* = 9.0, 2.3 Hz, 1H), 7.43 (dd, *J* = 8.3, 4.2 Hz, 1H).
^{13}C NMR (101 MHz, $CDCl_3$) δ 150.8, 146.8, 135.3, 132.4, 131.3, 130.6, 129.0, 126.6, 122.1.
The characterization data was in agreement with the previously published data.[4]

6-(Methoxycarbonyl)quinoline **4f**

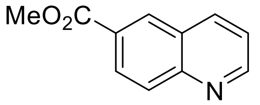

4f

The title compound was prepared according to the general procedure C from 6-(methoxycarbonyl)quinoline *N*-oxide **3f** (x40.6 mg, 0.20 mmol) using DTT (37.0 mg, 0.24 mmol) and PQ (6.2 mg, 0.03 mmol) with the reaction time of 1 h. The product was isolated with flash chromatography (silica gel, *n*-Hex/EtOAc 20:1 → 10:1 /0.5% TEA) and obtained as a yellow solid (29.6 mg, 79%).

^{1}H NMR (400 MHz, $CDCl_3$) δ 9.02 (dd, *J* = 4.2, 1.8 Hz, 1H), 8.60 (d, *J* = 1.9 Hz, 1H), 8.31 (dd, *J* = 8.8, 1.9 Hz, 1H), 8.27 (dd, *J* = 8.4, 1.0 Hz, 1H), 8.15 (d, *J* = 8.9 Hz, 1H), 7.48 (dd, *J* = 8.3, 4.2 Hz, 1H), 4.00 (s, 3H).
^{13}C NMR (101 MHz, $CDCl_3$) δ 166.8, 152.7, 150.2, 137.5, 131.2, 130.0, 129.1, 128.3, 127.6, 122.0, 52.6.
The characterization data was in agreement with the previously published data.[32]

6-Nitroquinoline **4g**

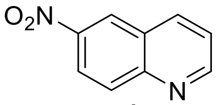

4g

The title compound was prepared according to the general procedure C from 6-nitroquinoline *N*-oxide **3g** (38.2 mg, 0.20 mmol) using DTT (46.3 mg, 0.30 mmol) and PQ (8.3 mg, 0.040 mmol) with the reaction time of 6 h. The product was isolated with flash chromatography (silica gel, *n*-Hex/EtOAc 5:1 → 1:1 /0.5% TEA) and obtained as a pale yellow fluffy solid (26.4 mg, 76%).

^{1}H NMR (400 MHz, $CDCl_3$) δ 9.11 (dd, *J* = 4.2, 1.8 Hz, 1H), 8.81 (d, *J* = 2.6 Hz, 1H), 8.49 (dd, *J* = 9.2, 2.5 Hz, 1H), 8.37 (dd, *J* = 8.4, 1.9 Hz, 1H), 8.25 (d, *J* = 9.2 Hz, 1H), 7.59 (dd, *J* = 8.4, 4.2 Hz, 1H).
^{13}C NMR (101 MHz, $CDCl_3$) δ 154.0, 150.4, 145.7, 138.0, 131.6, 127.2, 124.8, 123.1, 123.0.

The characterization data was in agreement with the previously published data.[13]

4-Methylquinoline **4h**

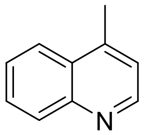
4h

The title compound was prepared according to the general procedure C from 4-methylquinoline *N*-oxide **3h** (32.0 mg, 0.2 mmol) using DTT (37.0 mg, 0.24 mmol) and PQ (6.2 mg, 0.030 mmol) with the reaction time of 1 h. The product was isolated with flash chromatography (silica gel, *n*-Hex/EtOAc 10:1 → 5:1 /0.5% TEA) and obtained as a yellow oil (19.2 mg, 67%).
^{1}H NMR (400 MHz, $CDCl_3$) δ 8.78 (d, *J* = 4.4 Hz, 1H), 8.11 (dd, *J* = 8.5, 0.7 Hz, 1H), 8.00 (dd, *J* = 8.4, 0.9 Hz, 1H), 7.74–7.68 (m, 1H), 7.59–7.54 (m, 1H), 7.23 (dd, *J* = 4.4, 1.0 Hz, 1H), 2.71 (s, 3H).
^{13}C NMR (101 MHz, $CDCl_3$) δ 150.3, 148.1, 144.4, 130.2, 129.2, 128.4, 126.4, 124.0, 122.0, 18.8.
The characterization data was in agreement with the previously published data.[4]

4-(1,3-Dioxolan-2-yl)quinoline **4i**

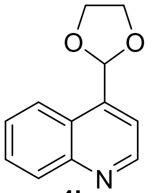
4i

The title compound was prepared according to the general procedure C from 4-(1,3-dioxolan-2-yl)quinoline *N*-oxide **3i** (43.3 mg, 0.20 mmol) using DTT (37.0 mg, 0.24 mmol) and PQ (6.2 mg, 0.030 mmol) with the reaction time of 1 h. The product was isolated with flash chromatography (silica gel, *n*-Hex/EtOAc 10:1 → 5:1 /0.5% TEA) and obtained as a yellow oil (27.8 mg, 69%).

^{1}H NMR (400 MHz, $CDCl_3$) δ 8.94 (d, *J* = 4.4 Hz, 1H), 8.20 (dd, *J* = 8.4, 0.8 Hz, 1H), 8.15 (d, *J* = 8.5 Hz, 1H), 7.75–7.70 (m, 1H), 7.64–7.57 (m, 2H), 6.47 (s, 1H), 4.16 (s, 4H).
^{13}C NMR (101 MHz, $CDCl_3$) δ 150.4, 148.6, 142.6, 130.2, 129.4, 127.0, 126.0, 124.3, 117.5, 100.8, 65.6.
The characterization data was in agreement with the previously published data.[33]

4,7-Dichloroquinoline **4j**

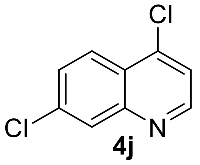
4j

The title compound was prepared according to the general procedure C from 4,7-dichloroquinoline *N*-oxide **3j** (42.6 mg, 0.20 mmol) using DTT (37.0 mg, 0.24 mmol) and PQ (6.2 mg, 0.030 mmol) with the reaction time of 1 h. The product was isolated with flash chromatography (silica gel, *n*-Hex/EtOAc 20:1/0.5% TEA) and obtained as a white solid (29.4 mg, 75%).

^{1}H NMR (400 MHz, $CDCl_3$) δ 8.78 (d, *J* = 4.7 Hz, 1H), 8.17 (d, *J* = 9.0 Hz, 1H), 8.12 (d, *J* = 2.1 Hz, 1H), 7.59 (dd, *J* = 9.0, 2.1 Hz, 1H), 7.48 (d, *J* = 4.7 Hz, 1H).
^{13}C NMR (101 MHz, $CDCl_3$) δ 151.1, 149.6, 142.8, 136.7, 128.9, 128.8, 125.7, 125.1, 121.6.
The characterization data was in agreement with the previously published data.[34]

8-Methylquinoline **4k**

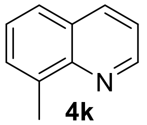
4k

The title compound was prepared according to the general procedure C from 8-methylquinoline *N*-oxide **3k** (31.9 mg, 0.20 mmol) using DTT (46.3 mg, 0.30 mmol) and PQ (6.2 mg, 0.030 mmol) with the reaction time of 6 h. The product was isolated with flash chromatography (silica gel, *n*-Hex/EtOAc 10:1/0.5% TEA) and obtained as a clear oil (18.0 mg, 63%).

^{1}H NMR (400 MHz, $CDCl_3$) δ 8.95 (dd, *J* = 4.2, 1.8 Hz, 1H), 8.12 (dd, *J* = 8.3, 1.8 Hz, 1H), 7.66 (d, *J* = 8.1 Hz, 1H), 7.56 (d, *J* = 6.5 Hz, 1H), 7.46–7.36 (m, 2H), 2.83 (s, 3H).
^{13}C NMR (101 MHz, $CDCl_3$) δ 149.4, 147.5, 137.2, 136.4, 129.7, 128.4, 126.4, 126.0, 121.0, 18.3.
The characterization data was in agreement with the previously published data.[35]

2-Phenylquinoline **4l**

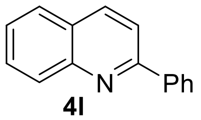

4l

The title compound was prepared according to the general procedure C from 2-phenylquinoline *N*-oxide **3l** (44.8 mg, 0.2 mmol) using DTT (46.3 mg, 0.30 mmol) and PQ (6.2 mg, 0.030 mmol) with the reaction time of 4 h. The product was isolated with flash chromatography (silica gel, *n*-Hex/EtOAc 20:1/0.5% TEA) and obtained as a white solid (34.6 mg, 83%).

^{1}H NMR (400 MHz, $CDCl_3$) δ 8.21 (d, *J* = 8.6 Hz, 1H), 8.19–8.14 (m, 3H), 7.87 (d, *J* = 8.5 Hz, 1H), 7.82 (dd, *J* = 8.1, 1.9 Hz, 1H), 7.78–7.68 (m, 1H), 7.55–7.50 (m, 3H), 7.49–7.43 (m, 1H).
^{13}C NMR (101 MHz, $CDCl_3$) δ 157.5, 148.4, 139.8, 136.9, 129.9, 129.8, 129.5, 129.0, 127.7, 127.6, 127.3, 126.4, 119.2.
The characterization data was in agreement with the previously published data.[32]

Benzo[*f*]quinoline **4m**

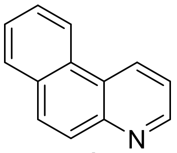

4m

The title compound was prepared according to the procedure C from benzo[*f*]quinoline *N*-oxide **3m** (39.3 mg, 0.20 mmol) using DTT (46.3 mg, 0.30 mmol) and PQ (6.2 mg, 0.030 mmol) with the reaction time of 4 h. The product was isolated with flash chromatography (silica gel, *n*-Hex/EtOAc 20:1 → 5:1 /0.5% TEA) and obtained as a white solid (25.1 mg, 70%).

^{1}H NMR (400 MHz, $CDCl_3$) δ 8.98–8.94 (m, 2H), 8.63 (d, *J* = 8.3 Hz, 1H), 7.99 (s, 2H), 7.96–7.93 (m, 1H), 7.73–7.63 (m, 2H), 7.56 (dd, *J* = 8.2, 4.5 Hz, 1H).
^{13}C NMR (101 MHz, $CDCl_3$) δ 149.9, 148.4, 131.8, 131.0, 130.8, 129.8, 128.9, 128.4, 127.5, 127.3, 125.6, 122.7, 121.5.
The characterization data was in agreement with the previously published data.[36]

Isoquinoline **4n**

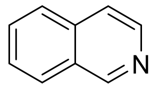

4n

The title compound was prepared according to the general procedure C from isoquinoline *N*-oxide **3n** (29.4 mg, 0.2 mmol) using DTT (46.3 mg, 0.30 mmol) and PQ (8.3 mg, 0.040 mmol) with the reaction time of 6 h. The product was isolated with flash chromatography (silica gel, *n*-Hex/EtOAc 10:1/0.5% TEA) and obtained as a yellow oil (15.5 mg, 60%).

^{1}H NMR (400 MHz, $CDCl_3$) δ 9.26 (s, 1H), 8.53 (d, *J* = 5.8 Hz, 1H), 7.98 (dd, *J* = 8.3, 1.1 Hz, 1H), 7.83 (dd, *J* = 8.3, 1.0 Hz, 1H), 7.74–7.66 (m, 1H), 7.65 (d, *J* = 5.8 Hz, 1H), 7.66–7.57 (m, 1H).
^{13}C NMR (101 MHz, $CDCl_3$) δ 152.7, 143.2, 135.9, 130.5, 128.8, 127.8, 127.4, 126.6, 120.6.
The characterization data was in agreement with the previously published data.[4]

5-Nitroisoquinoline **4o**

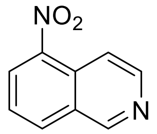

4o

The title compound was prepared according to the general procedure C from 5-nitroisoquinoline *N*-oxide **3q** (37.6 mg, 0.20 mmol) using DTT (46.3 mg, 0.30 mmol) and PQ (10.4 mg, 0.050 mmol) with the reaction time of 7 h. The product was isolated with flash chromatography (silica gel, *n*-Hex/EtOAc 5:1 → 1:1 /0.5% TEA) and obtained as an off-white solid (13.4 mg, 39%).

^{1}H NMR (400 MHz, $CDCl_3$) δ 9.40 (s, 1H), 8.78 (d, *J* = 6.3 Hz, 1H), 8.59 (dd, *J* = 7.7, 1.3 Hz, 1H), 8.52 (d, *J* = 6.3 Hz, 1H), 8.32 (d, *J* = 8.1 Hz, 1H), 7.75 (t, *J* = 7.9 Hz, 1H).
^{13}C NMR (101 MHz, $CDCl_3$) δ 153.2, 146.8, 144.7, 135.1, 129.3, 128.7, 128.5, 126.0, 116.0.
The characterization data was in agreement with the previously published data.[37]

7-Azaindole **4p**

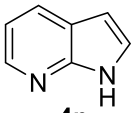

4p

The title compound was prepared according to the general procedure C from 7-azaindole *N*-oxide (26.9 mg, 0.2 mmol) using DTT (46.3 mg, 0.30 mmol) and PQ (10.4 mg, 0.050 mmol) with the reaction time of 7 h. The product was isolated with flash chromatography (silica gel, *n*-Hex/EtOAc 10:1 → 1:1 /0.5% TEA) and obtained as a yellow solid (17.2 mg, 73%).

^{1}H NMR (400 MHz, $CDCl_3$) δ 11.16 (br s, 1H), 8.35 (dd, *J* = 4.8, 1.6 Hz, 1H), 7.97 (dd, *J* = 7.8, 1.6 Hz, 1H), 7.39 (d, *J* = 3.6 Hz, 1H), 7.10 (dd, *J* = 7.8, 4.8 Hz, 1H), 6.52 (d, *J* = 3.5 Hz, 1H).
^{13}C NMR (101 MHz, $CDCl_3$) δ 148.9, 142.6, 129.2, 125.3, 120.6, 115.9, 100.8.
The characterization data was in agreement with the previously published data.[4]

Quinoxaline **4q**

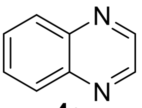

4q

The title compound was prepared according to the general procedure C from quinoxaline 1,4-dioxide **3p** (32.4 mg, 0.20 mmol) using DTT (65.0 mg, 0.42 mmol) and PQ (10.4 mg, 0.050 mmol) with the reaction time of 7 h. The product was isolated with flash chromatography (silica gel, *n*-Hex/EtOAc 5:1 → 3:1 /0.5% TEA) and obtained as a yellow oil (19.3 mg, 74%).

^{1}H NMR (400 MHz, $CDCl_3$) δ 8.86 (s, 2H), 8.16–8.11 (m, 2H), 7.83–7.77 (m, 2H).
^{13}C NMR (101 MHz, $CDCl_3$) δ 145.1, 143.2, 130.2, 129.7.
The characterization data was in agreement with the previously published data.[38]

Following substrates were also tested in the catalysis but the products were not isolated due to difficult purification. The given yields are NMR yields from the reaction mixture determined using 1,3,5-trimethoxybenzene as the internal standard.

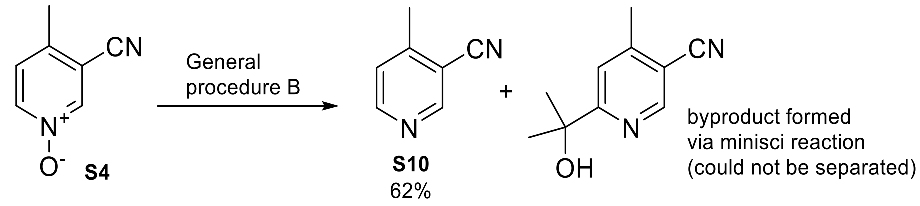

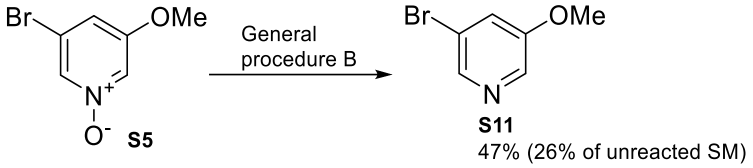

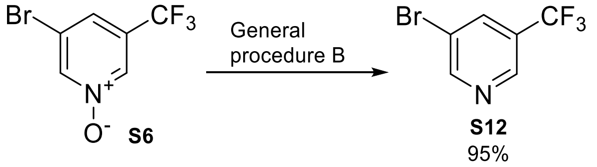

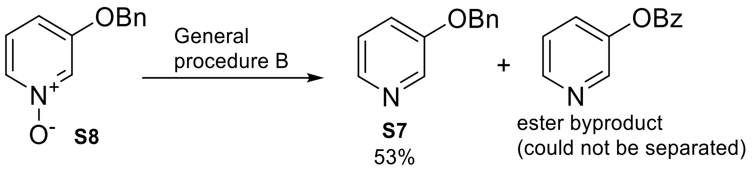

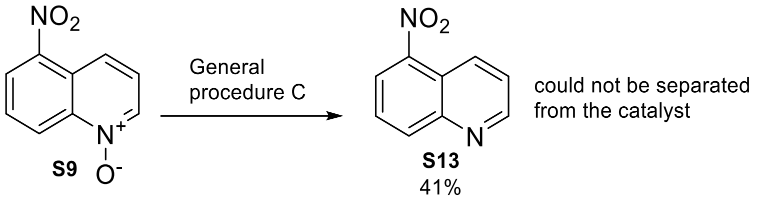

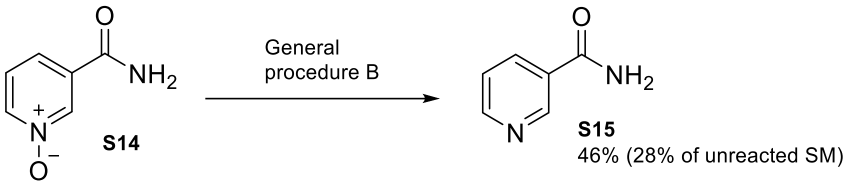

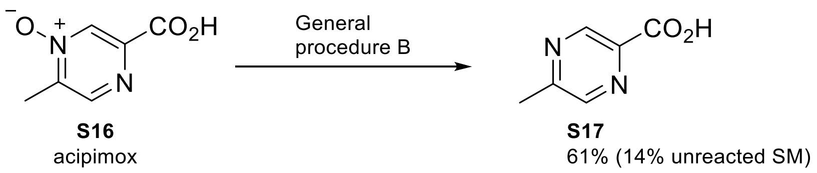

Scale-up experiment

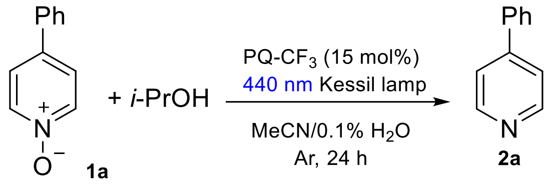

Compound **1a** (172.0 mg, 1.0 mmol, 1.0 equiv) and PQ-CF_3 (51.6 mg, 0.15 mmol, 15 mol%) were weighted to a two-neck flask equipped with a stirring bar. *i*-PrOH (230.0 µL, 3.0 mmol, 3.0 equiv) and MeCN/0.1% H_2O (20 mL, 0.05 M) were added to the flask, and the reaction mixture was bubbled with argon for 30 min. The reaction mixture was stirred and irradiated with blue light (440 nm) under argon for 24 h. The reaction mixture was concentrated, and the product **2a** was purified with flash chromatography (silica gel, pentane/acetone 5:1 /0.5% TEA) and obtained as a light yellow solid (142.6 mg, 92%).

The set-up for the scale-up experiment is shown in Figure S2. The reaction flask was irradiated with Kessil PR160L lamp (440 nm, 50% intensity used) with 4 cm distance between the reaction flask and the lamp. Temperature varied 35–37 ˚C due to heat produced by the lamp.

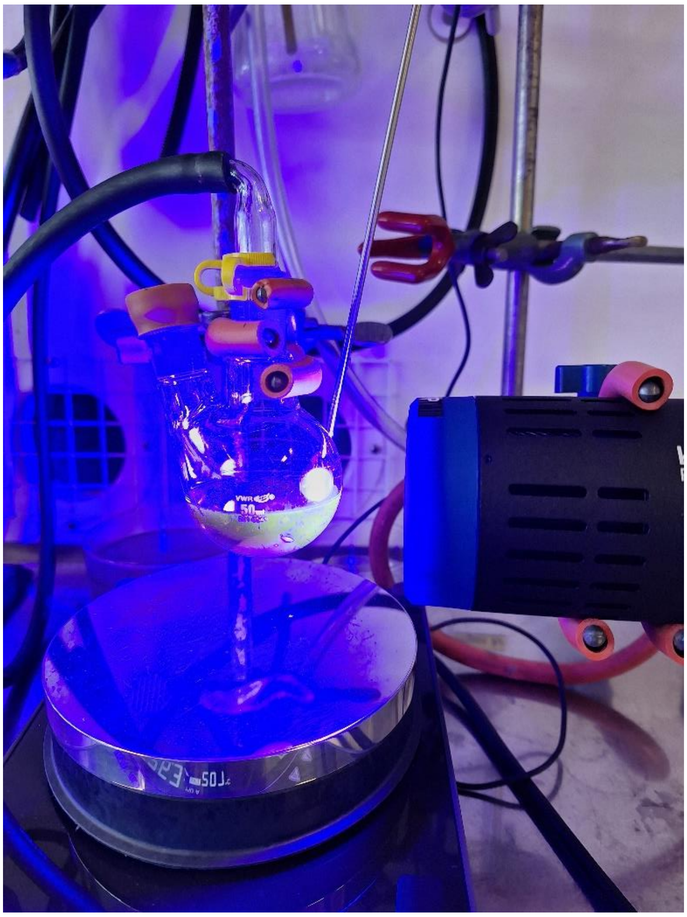

Figure S2: The set-up for the scale-up experiment.

Other substrates

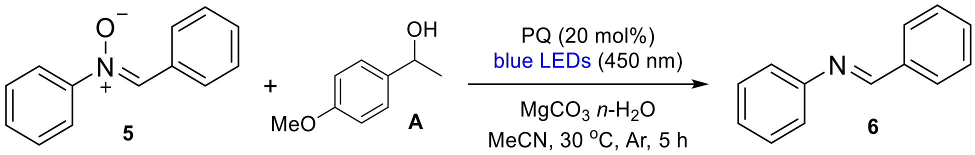

Alcohol **A** (46.1 mg, 0.3 mmol, 1.5 equiv), the compound **5** (39.9 mg, 0.2 mmol, 1 equiv), PQ (8.5 mg, 0.04 mmol, 20 mol%) and $MgCO_3$ *n*-H_2O (78.9 mg, 20 mg/mL) were weighted to a vial. MeCN (4.0 mL, 0.05 M) was added, and the vial was sealed with a septum. The reaction mixture was bubbled with argon for 30 min. The reaction mixture was irradiated with blue LEDs at 30 ˚C under argon for 5 h. NMR yield was determined from ^{1}H NMR spectrum (in $CDCl_3$) using 1,3,5-trimethoxybenzene as an internal standard.

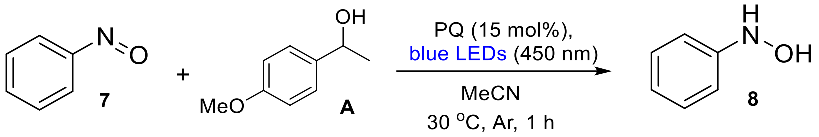

Alcohol **A** (45.3 mg, 0.3 mmol, 1.5 equiv), the compound **7** (21.2 mg, 0.2 mmol, 1.0 equiv) and PQ (6.2 mg, 0.03 mmol, 15 mol%) were weighted to a vial. MeCN (4.0 mL, 0.05 M) was added, and the vial was sealed with a septum. The reaction mixture was bubbled with argon for 30 min. The reaction mixture was irradiated with blue LEDs at 30 ˚C under argon for 1 h. NMR yield was determined from ^{1}H NMR spectrum (in $CDCl_3$) using 1,3,5-trimethoxybenzene as an internal standard.

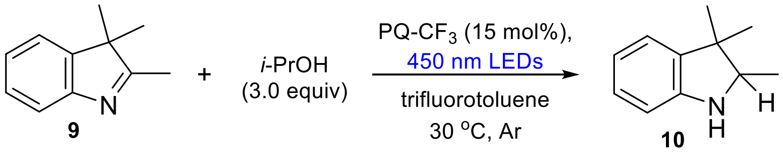

The compound **9** (32.1 mg, 0.2 mmol, 1.0 equiv) and PQ-CF_3 (10.2 mg, 0.03 mmol, 15 mol%) were weighted to a vial. *i*-PrOH (46.0 µL, 0.6 mmol, 3 equiv) and trifluorotoluene (4.0 mL, 0.05 M) were added, and the vial was sealed with a septum. The reaction mixture was bubbled with argon for 30 min. The reaction mixture was irradiated with blue LEDs at 30 ˚C under argon for 22 h. NMR yield was determined from ^{1}H NMR spectrum (in $CDCl_3$) using 1,3,5-trimethoxybenzene as an internal standard.

Mechanistic studies

Cyclic voltammetry experiments

Cyclic voltammetries were performed on a Gamry electrochemical workstation. The cell set-up consisted in a three-electrode system using a platinum coil as the counter electrode, platinum wire as the reference electrode, in the presence of ferrocene as an internal standard, and a glassy carbon (GC) electrode (disk, 3mm diameter, geometric surface area ~ 0.071 cm^2) as the working electrode. The GC electrode was polished using 1 µm alumina slurry followed by distilled water and acetone washing. All experiments were performed at room temperature under argon atmosphere in a 0.10 M NBu_4PF_6 solution in MeCN. Starting potential 0 V, oxidative scan to 2 V (first switching potential), backward (reductive) scan to -1 V (second switching potential).

Hydroquinones were prepared *in situ* for cyclic voltammetry measurements as following:

H_2PQ-CF_3: A degassed solution of PQ-CF_3 (34.5 mg, 0.1 mmol), *i*-PrOH (23.0 µL, 0.3 mmol) and MeCN (2.0 mL) was irradiated with blue LEDs under argon until the solution was colorless (took 15 min). Then 1.0 mL of the solution was transferred with a syringe to the cell for CV measurements.

H_2PQ: A degassed solution of PQ (6.3 mg, 0.03 mmol), 1,4-cyclohexadiene (5.02 mg, 0.063 mmol) and MeCN (1.2 mL) was irradiated with blue LEDs under argon until the solution was nearly colorless (took 30 min). Then the solution was transferred with a syringe to the cell for CV measurements.

The ground state redox potentials of H_2PQs have been determined from the half peak potential $E_{p/2}$ of the anodic wave, as it has been reported to be a reasonable estimation of $E^{(0)}$ for irreversible redox couples at high scan rates (>500 mV/s).[39]

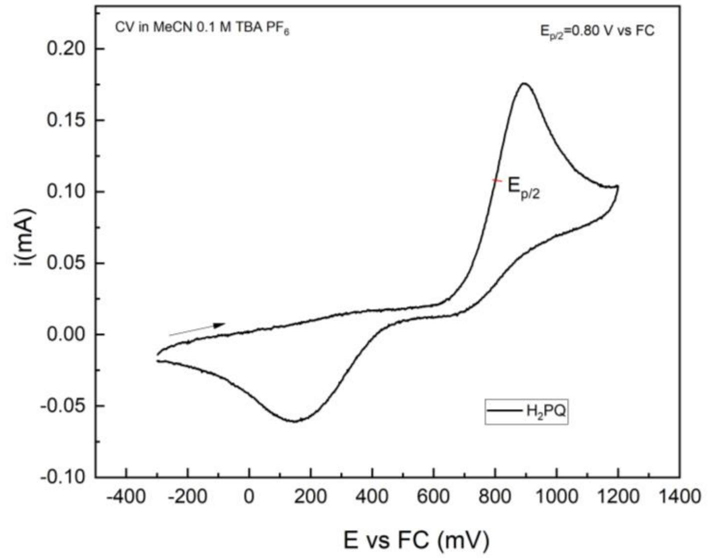

Figure S3: Cyclic voltammetry of H_2PQ, obtained on GCE in a 0.10 M NBu_4PF_6 solution in MeCN under Ar with scan rate of 1 V/s. IUPAC plotting convention: starting potential 0 V, first oxidative scan to 2 V (first switching potential), backward (reductive) scan to -1 V (second switching potential).

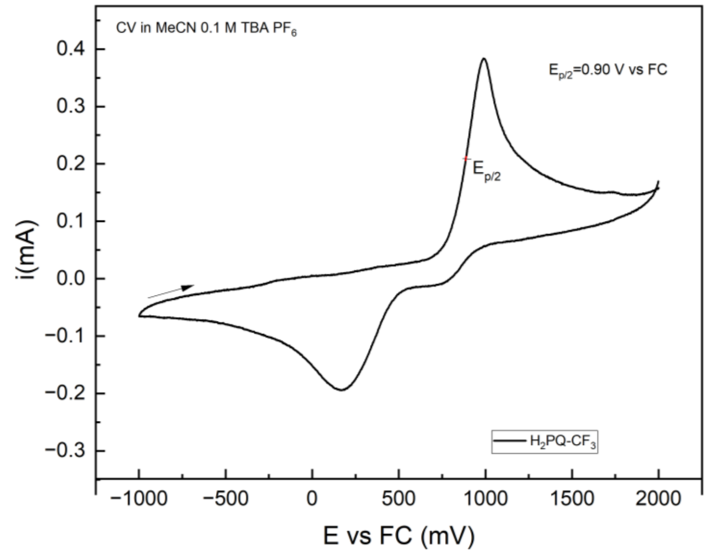

Figure S4: Cyclic voltammetry of H_2PQ-CF_3, obtained on GCE in a 0.10 M NBu_4PF_6 solution in MeCN under Ar with scan rate of 1 V/s. IUPAC plotting convention: starting potential 0 V, first oxidative scan to 2 V (first switching potential), backward (reductive) scan to -1 V (second switching potential.

UV-Vis absorption experiments

UV-Vis spectra were recorded with a Varian Cary 50 UV-Visible spectrophotometer using standard 10 mm UV-Vis quartz cuvettes. H_2PQs were prepared *in situ* in cuvettes under argon.

For the measurements, 0.2 mM solutions of PQ and 1-methyl-1,4-cyclohexadiene (3 equiv), and $PQ-CF_3$ and *i*-PrOH (3 equiv) were prepared in MeCN. The solution was placed in a cuvette, the cuvette was sealed with a septum and parafilm, and the sample was bubbled with argon for 10 min. To form H_2PQs *in situ*, the sample was irradiated with blue LEDs for 5 min or until the sample was colorless, and a UV-vis spectrum was recorded immediately.

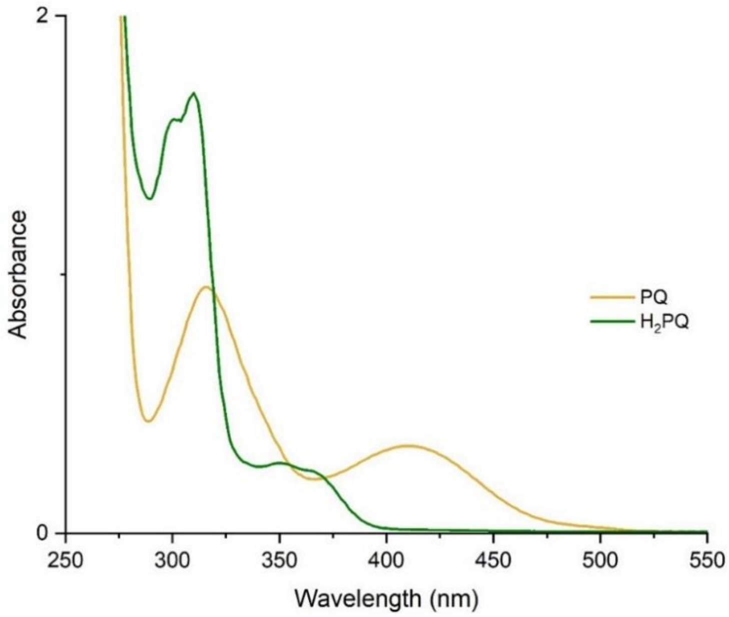

Figure S5: UV-Vis spectra of PQ (yellow line) and H_2PQ (green line) in a 0.2 mM solution in MeCN.

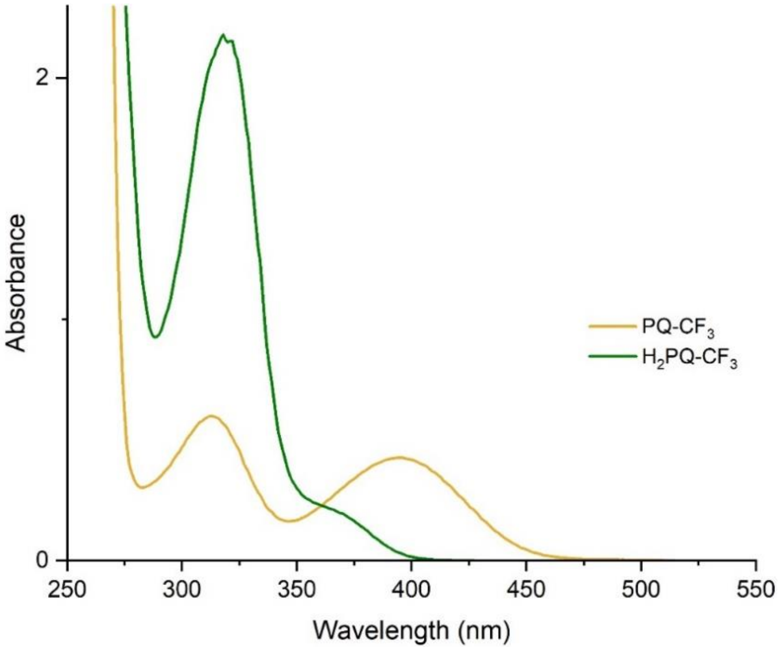

Figure S6: UV-Vis spectra of PQ-CF_3 (yellow line) and H_2PQ-CF_3 (green line) in a 0.2 mM solution in MeCN.

To record the UV-Vis spectra of PQH_2-CF_3 combined with substrates, PQH_2-CF_3 was prepared as above (the solution with a specified concentration was placed in a cuvette, bubbled with argon, and irradiated with blue LEDs until the solution was colorless). Then, an argon purged 0.1 M solution of substrate **1b**, **1d** or **3a** in MeCN was added to the cuvette, and a UV-Vis spectrum was recorded after each addition.

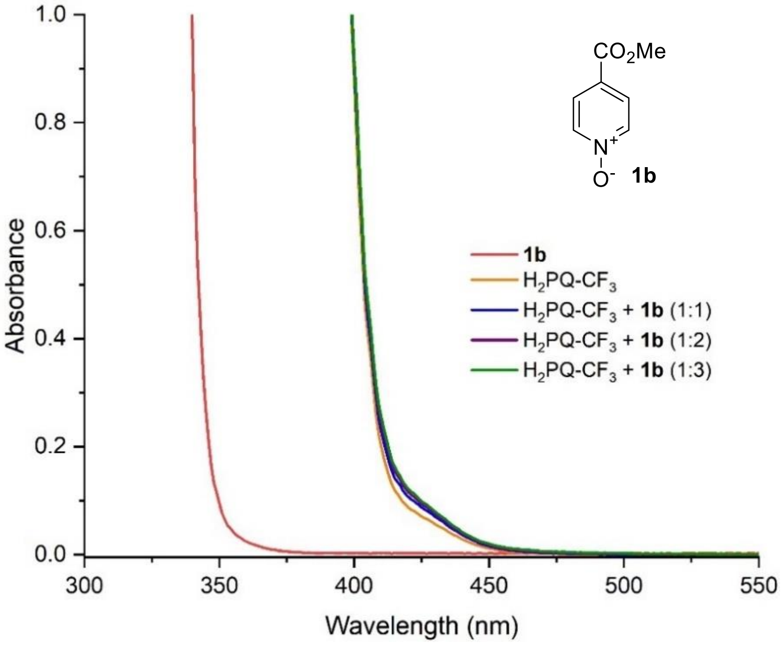

Figure S7: UV-Vis spectra of substrate **1b**, H_2PQ-CF_3, and H_2PQ-CF_3 with 1, 2 and 3 equivalents of **1b** in a 0.010 M solution in MeCN. No remarkable shift of H_2PQ-CF_3 was observed.

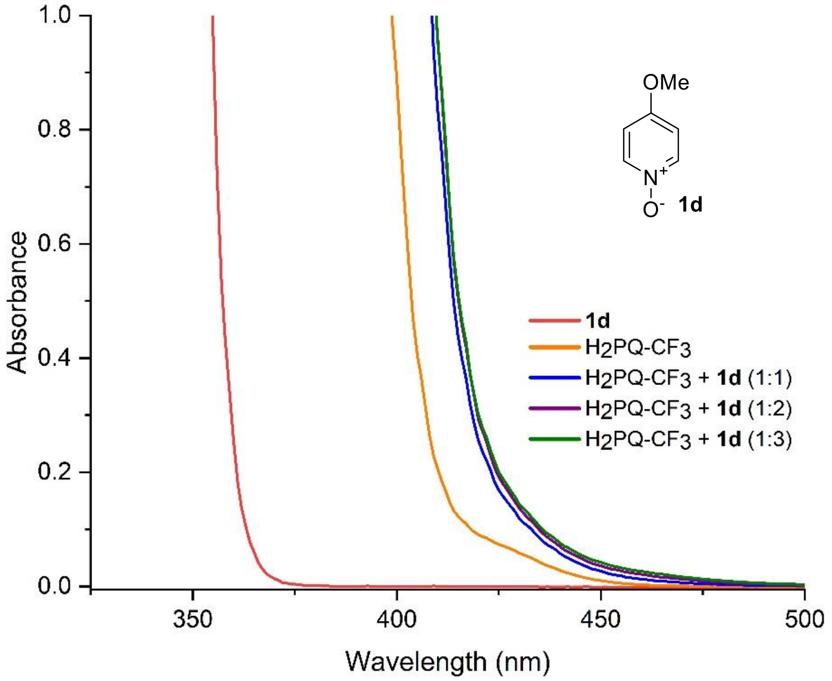

Figure S8: UV-Vis spectra of substrate **1d**, H_2PQ-CF_3, and H_2PQ-CF_3 with 1, 2 and 3 equivalents of **1d** in a 0.010 M solution in MeCN. A clear bathochromic shift of H_2PQ-CF_3 was observed.

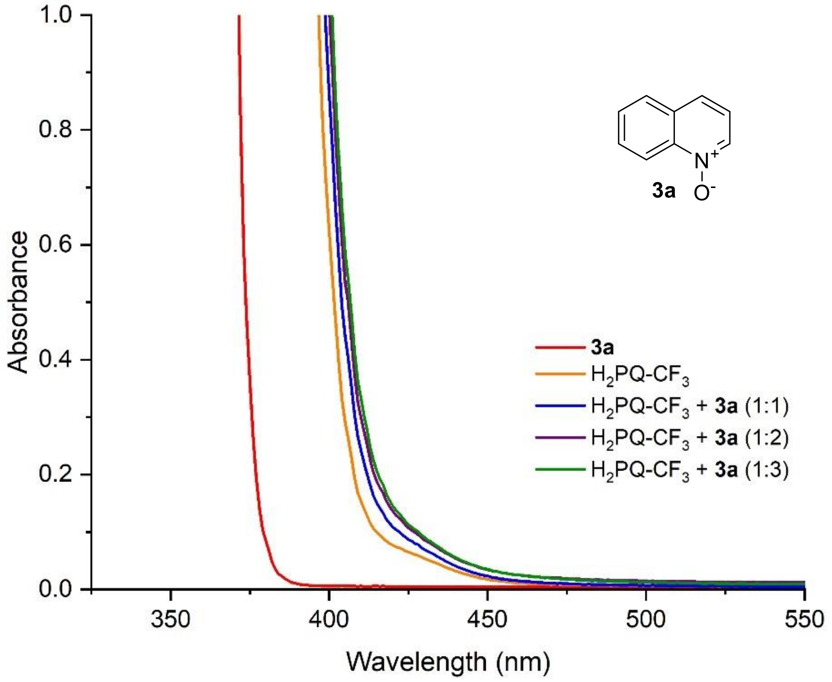

Figure S9: UV-Vis spectra of substrate **3a**, H_2PQ-CF_3, and H_2PQ-CF_3 with 1, 2 and 3 equivalents of **3a** in a 0.0075 M solution in MeCN. A small bathochromic shift of H_2PQ-CF_3 can be observed.

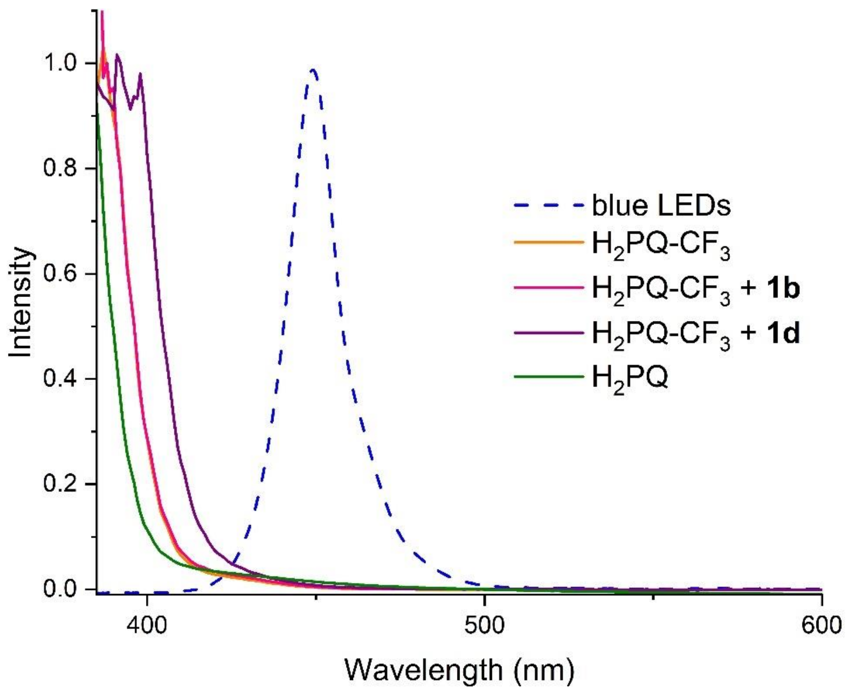

Figure S10: Normalized absorption spectra of H_2PQ-CF_3 with both substrates **1b** and **1d** (in MeCN [0.0075 M], and H_2PQ (in MeCN [0.01 M])), and emission of blue LEDs (450 m) used in the catalysis.

Emission experiments

Emission spectra were recorded with JASCO FP-8550 Spectrofluorometer equipped with Xe lamp, and the measurements were done at r.t. Samples were prepared in standard 10 mm fluorescence quartz cuvettes. H_2PQs were prepared *in situ* in cuvettes.

For the measurements, 0.1 mM solutions of PQ and 1-methyl-1,4-cyclohexadiene (3 equiv), and PQ-CF_3 and *i*-PrOH (3 equiv) were prepared in MeCN. The solution was placed in a cuvette, the cuvette was sealed with a septum and parafilm, and the sample was bubbled with argon for 10 min. To form H_2PQs *in situ*, the sample was irradiated with blue LEDs for 5 min or until the sample was colorless, and the absorption and emission spectra were recorded immediately.

For the emission measurement of H_2PQ, 350 nm excitation (bandwidth = 5 nm for both excitation and emission) was used, and the emission spectrum was recorded from 360 nm to 650 nm. For the emission measurement of H_2PQ-CF_3, 320 nm excitation (bandwidth = 5 nm for both excitation and emission) was used, and the emission spectrum was recorded from 350 nm to 600 nm.

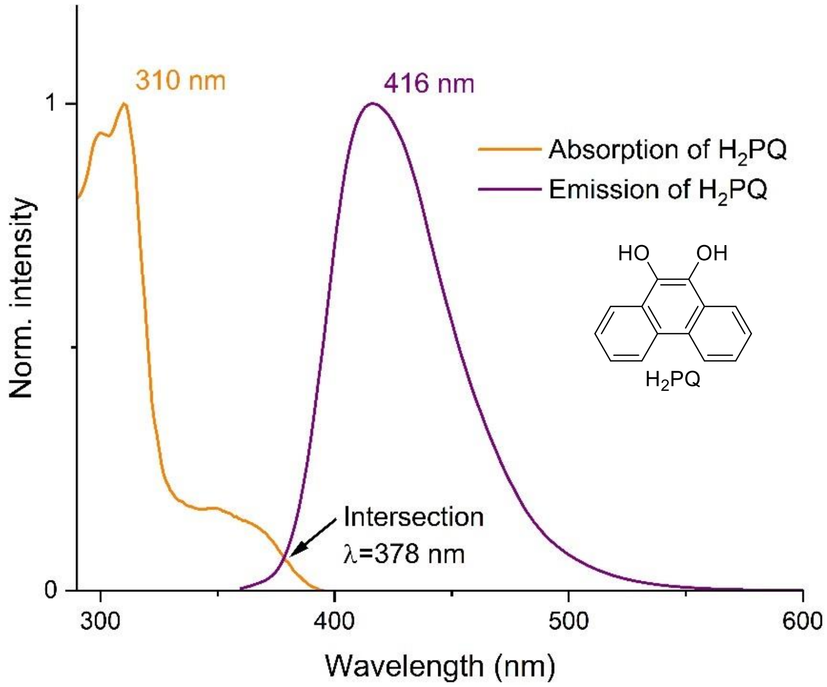

Figure S11: Normalized absorption spectrum (orange line) and emission spectrum (purple line) of H_2PQ in MeCN [0.1 mM]. The intersection of absorption and emission spectra (λ=378 nm) was used to calculate $E_{0,0}$.

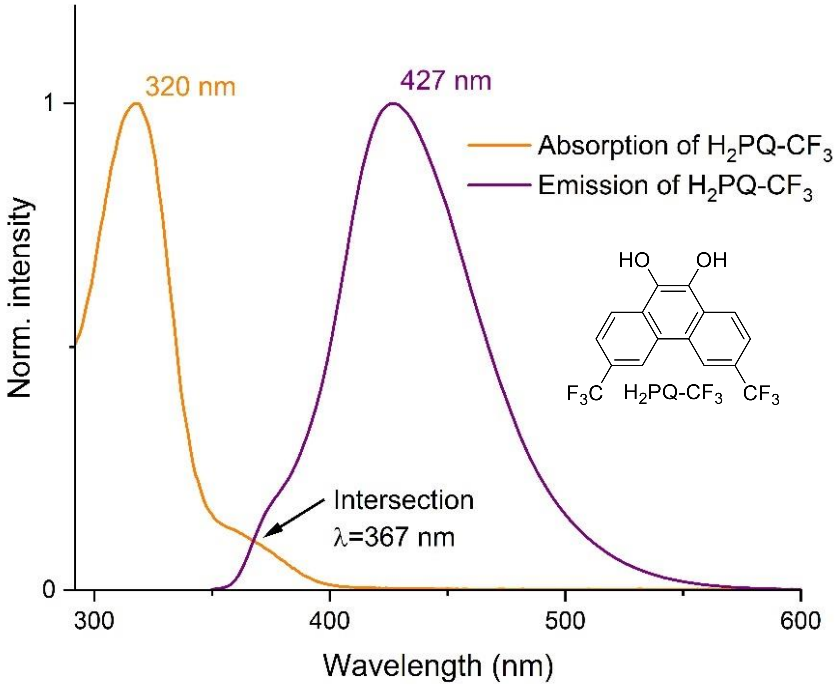

Figure S12: Normalized absorption spectrum (orange line) and emission spectrum (purple line) of $H_2PQ\text{-}CF_3$ in MeCN [0.1 mM]. The intersection of absorption and emission spectra (λ=367 nm) was used to calculate $E_{0,0}$.

Emission quenching experiment

For the emission quenching experiment, 2.0 mL of a degassed 0.12 mM solution of PQ-CF_3 and *i*-PrOH (3 equiv) in MeCN was placed in a screw cap fluorescence cuvette under argon atmosphere. H_2PQ-CF_3 was prepared *in situ* by irradiating the sample with blue LEDs. The emission of H_2PQ-CF_3 was measured, and a degassed 0.24 M solution of substrate in MeCN was added in a such way that the specified concentration was achieved. After each addition, the sample was stirred, and the emission was measured again. The measurement parameters were selected so that the inner filter effect was avoided: when substrate **1b** was used as a quencher, the excitation wavelength was 350 nm and emission was recorded from 370 nm to 550 nm; when **1d** was used as a quencher, excitation wavelength was 380 nm and emission was recorded from 390 nm to 550 nm (ex and em bandwidth were 2.5 nm). The Stern-Volmer plots were extracted from the results. The Stern-Volmer plot of substrate **1b** follows a linear relationship: $I_0/I = 1 + K_{SV}[Q]$, where Q=**1b**. The Stern-Volmer plot of substrate **1d** shows a non-linear relationship.

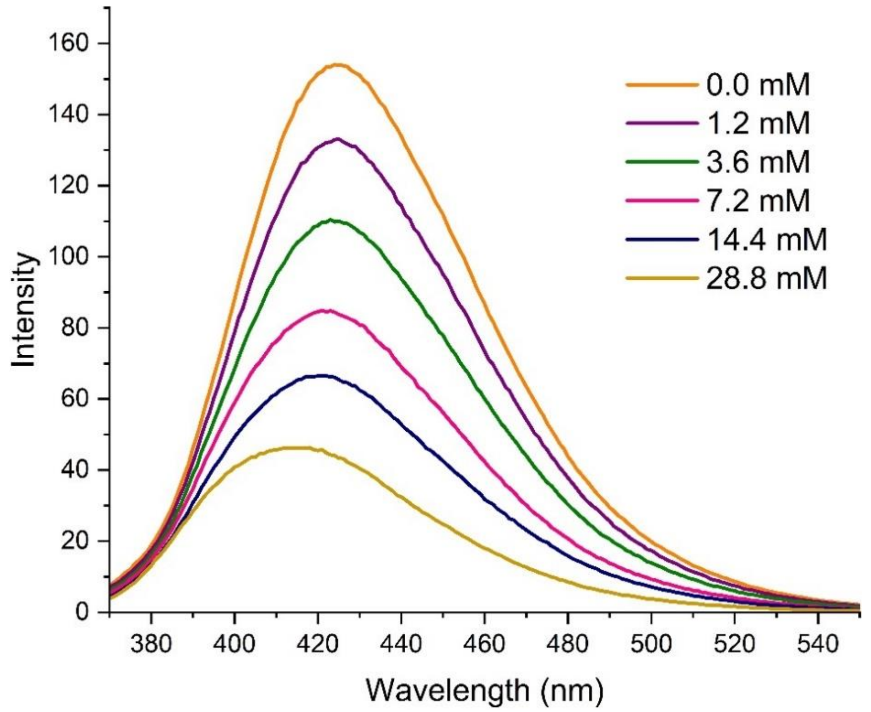

Figure S13: Emission quenching of H_2PQ-CF_3 using **1b** as a quencher in MeCN [0.12 mM].

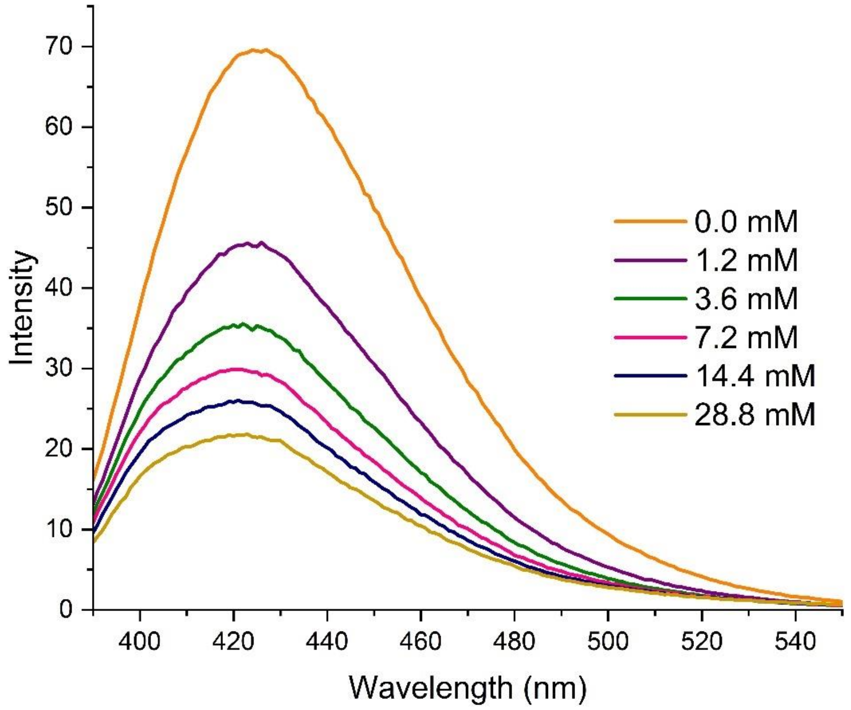

Figure S14: Emission quenching of H_2PQ-CF_3 using **1d** as a quencher in MeCN [0.12 mM].

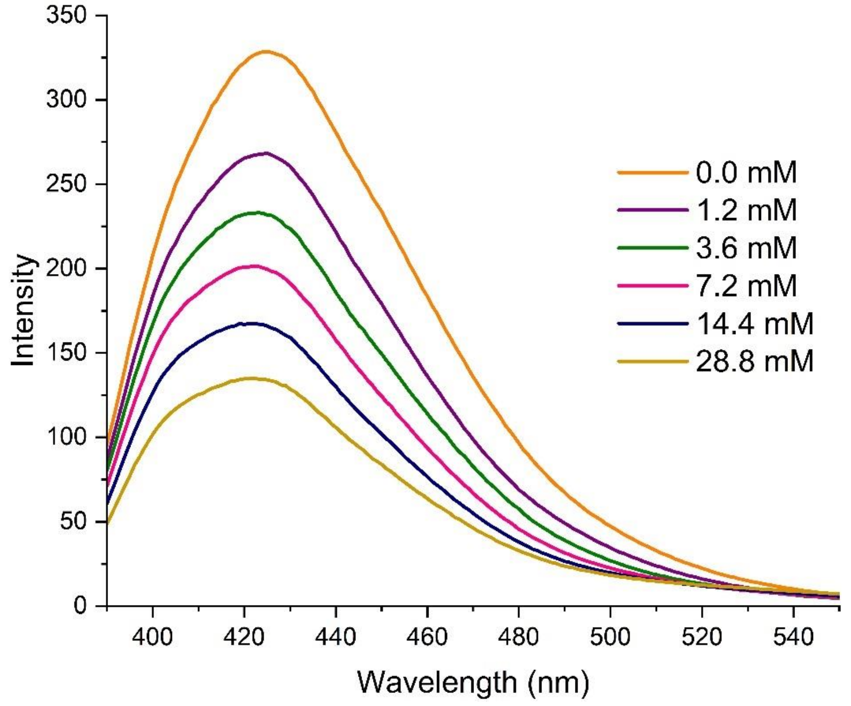

Figure S15: Emission quenching of H_2PQ-CF_3 using **1d** as a quencher in MeCN/1% H_2O [0.12 mM].

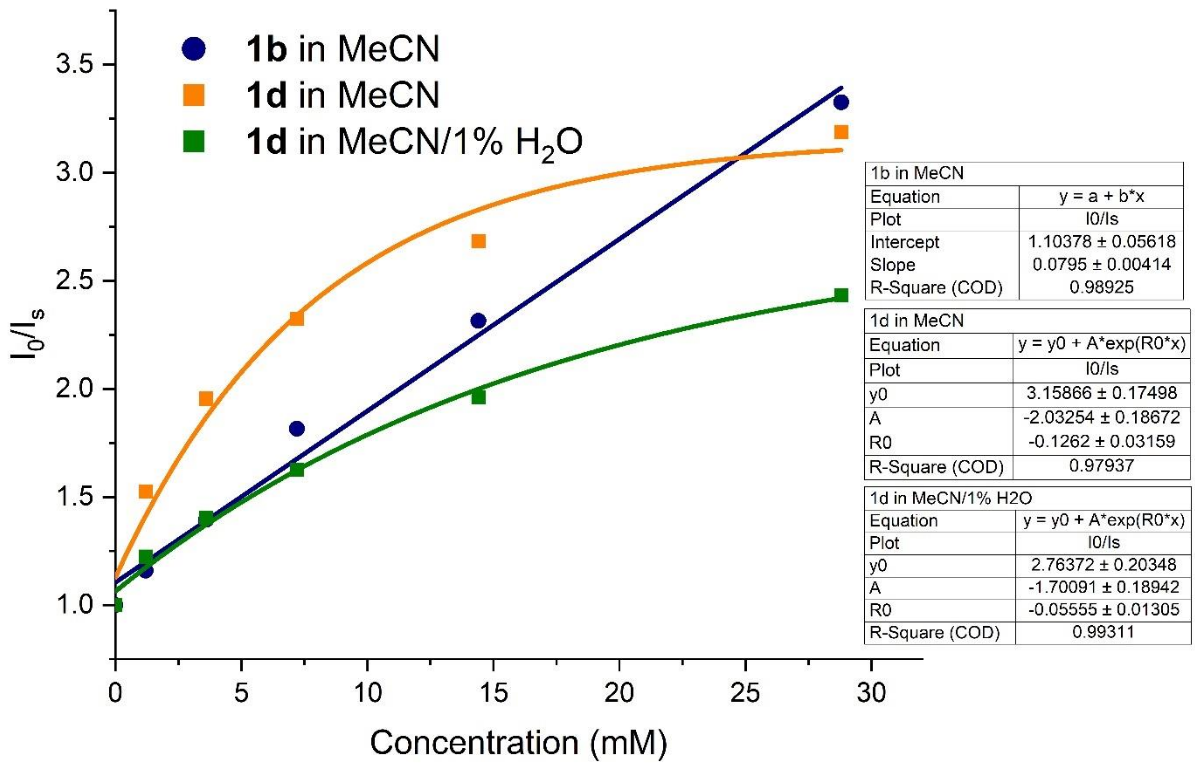

Figure S16: Stern-Volmer plot constructed from emission quenching experiment data of H_2PQ-CF_3 using **1b** (in MeCN) and **1d** (in MeCN and MeCN/1% H_2O) as quenchers.

Determination of excited state reduction potentials of H_2PQs

The excited state reduction potentials were determined as following:[40]

$$E_{\mathrm{H_2PQ^{+\bullet}/H_2PQ^*}} = E_{\mathrm{H_2PQ/H_2PQ^{+\bullet}}} - E_{0,0}$$

where ground state oxidation potential of H_2PQs ($E_{\mathrm{H_2PQ/H_2PQ^{+\bullet}}}$) determined by cyclic voltammetries was combined with exication energy ($E_{0,0}$) determined by emission measurements.

For H_2PQ:

$E_{\mathrm{H_2PQ/H_2PQ^{+\bullet}}} = 1.18\ \mathrm{V}$

$E_{0,0} = 3.28\ \mathrm{V}$

→ $E_{\mathrm{H_2PQ^{+\bullet}/H_2PQ^*}} = -2.10\ \mathrm{V}$

For H_2PQ-CF_3:

$E_{\mathrm{H_2PQ/H_2PQ^{+\bullet}}} = 1.28\ \mathrm{V}$

$E_{0,0} = 3.38\ \mathrm{V}$

→ $E_{\mathrm{H_2PQ^{+\bullet}/H_2PQ^*}} = -2.10\ \mathrm{V}$

NMR experiments

NMR experiments were conducted to reveal possible ground state interactions between H_2PQ-CF_3 and substrates. PQ-CF_3 (3.3 mg, 0.01 mmol) and *i*-PrOH (2.3 µL, 0.03 mmol) were dissolved to CD_3CN (1.0 mL), and the solution was degassed (freeze-pump-thaw cycle ×4) and placed under argon in an NMR tube. H_2PQ-CF_3 was prepared *in situ* by irradiating the solution with blue LEDs until it was colorless. Then the substrate was added, the sample was mixed, and ^{1}H NMR spectrum was recorded.

H_2PQ-CF_3 was combined with electronically different substrates **1b** (Figure S15) and **1d** (Figure S16). When H_2PQ-CF_3 was combined with **1b** (1 equiv, 2 equiv and 3 equiv were added and ^{1}H NMR spectrum was recorded after each addition), no clear shifts except broadening of O*H* signal of H_2PQ-CF_3 (marked with * in the spectra) were observed. When H_2PQ-CF_3 was combined with **1d**, shifts of both H_2PQ-CF_3 and **1d** signals were observed. Also, O*H* signal of H_2PQ-CF_3 almost disappeared because of broadening when H_2PQ-CF_3 was combined with **1d**. When the spectrum was recorded at lower temperature (-22 ˚C), O*H* signal appeared at 10.66 ppm (O*H* appears at 7.23 ppm of H_2PQ-CF_3 at room temperature). The remarkable shift of O*H* signal indicates hydrogen bonding between the compounds.

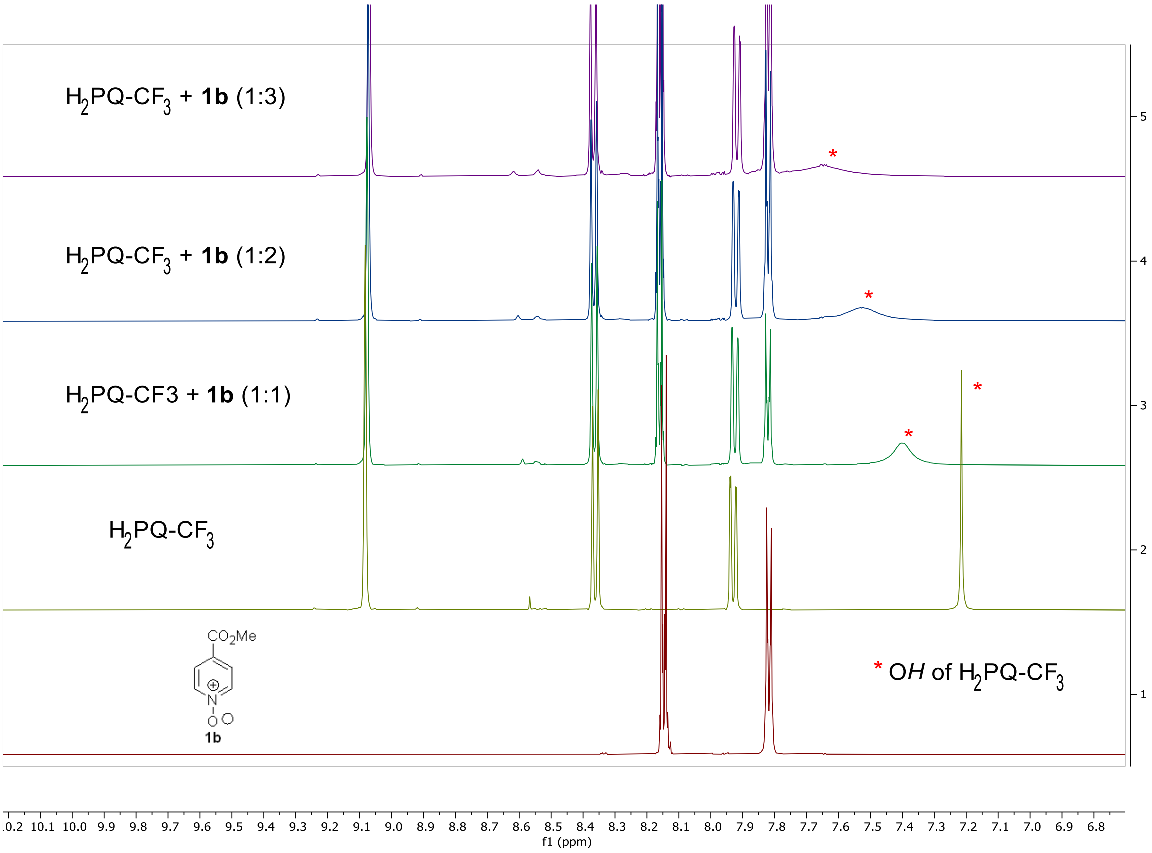

Figure S17: Stacked ^{1}H NMR spectra of substrate **1b** (spectrum 1), H_2PQ-CF_3 (2), H_2PQ-CF_3 + **1b** 1:1 (3), H_2PQ-CF_3 + **1b** 1:2 (4), H_2PQ-CF_3 + **1b** 1:3 (5).

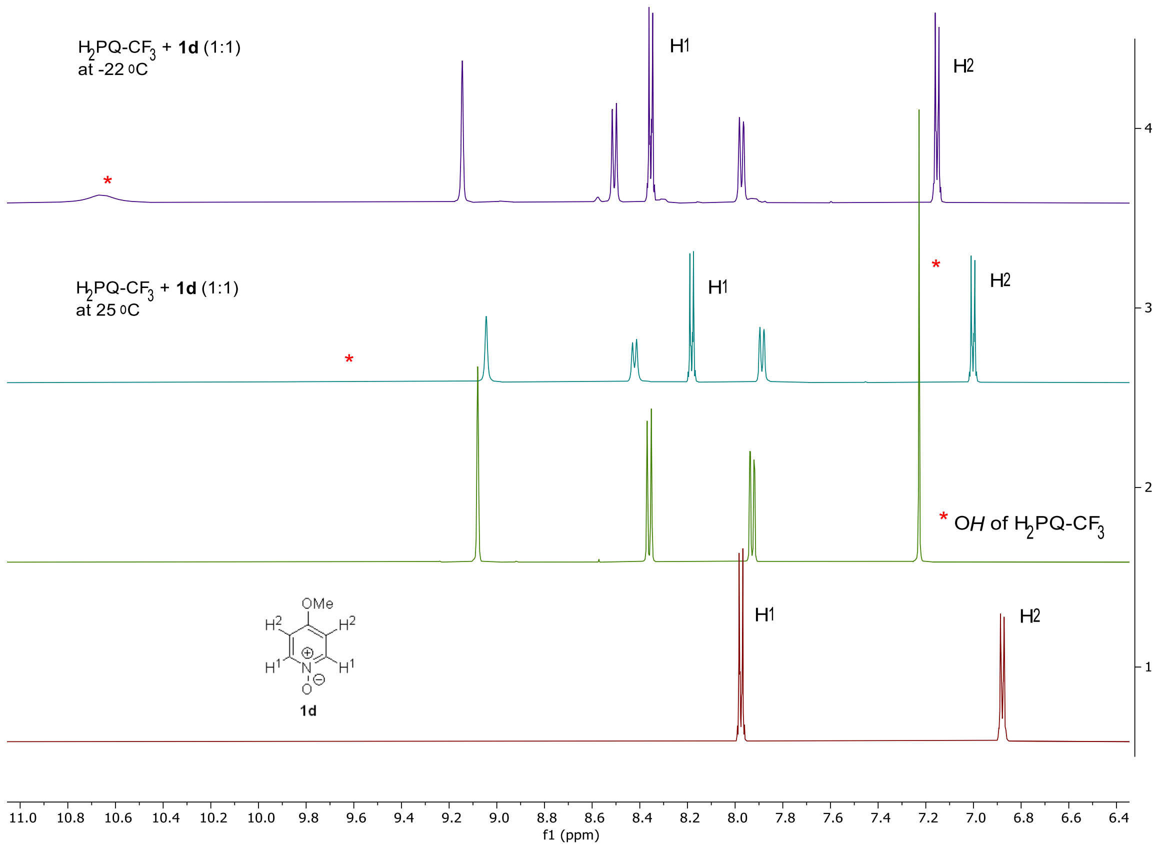

Figure S18: Stacked ^{1}H NMR spectra of substrate **1d** (spectrum 1), H_2PQ-CF_3 (2), H_2PQ-CF_3 + **1d** (1:1) at 25 ˚C (3), and H_2PQ-CF_3 + **1d** (1:1) at -22 ˚C (4).

Reaction kinetic experiments

Stoichiometric kinetics (see Figure 1E in mainbody) were performed to study the reaction kinetics of different H_2PQs and substrates. H_2PQs were prepared *in situ* in an NMR tube as in NMR experiments (0.01 M solution of H_2PQ/H_2PQ-CF_3 in CD_3CN), and then 1 equivalent of substrate **1b** or **1d** was added. The NMR tube was irradiated with blue LEDs and product formation was monitored with ^{1}H NMR.

Light ON/OFF experiment

Light ON/OFF experiment was conducted to demonstrate that H_2PQs need to be light-activated to induce the deoxygenation. H_2PQ-CF_3 was formed *in situ* in an NMR tube by irradiating a degassed (freeze-pump-thaw cycle ×4, and then placed under argon) solution of PQ-CF_3 (3.2 mg, 0.009 mmol, 1.0 equiv) and *i*-PrOH (1.15 µL, 0.015 mmol, 1.7 equiv) in CD_3CN (1.0 mL) with blue LEDs until the solution was colorless. Substrate **1b** (1.4 mg, 0.009 mmol, 1.0 equiv) was added to the tube, and it was kept in the dark for 30 min. Then the tube was irradiated with blue LEDs in 10-minute intervals, and a ^1H NMR spectrum was recorded after each interval, and conversion was monitored. Product formation was observed only under blue LED irradiation.

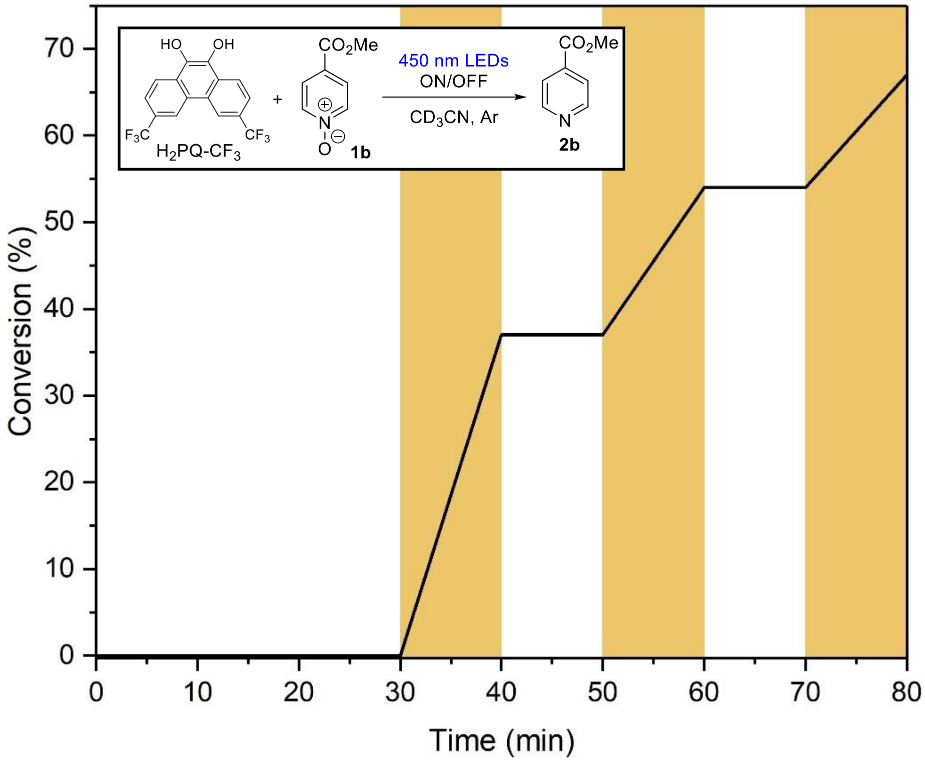

Figure S19: Light ON/OFF experiment. Yellow: light on, white: light off.

Computational details

General information

The range-separated dispersion-corrected M06-2X density functional[41] was used in combination with the 6-31+G(d,p)[42–47] basis set to optimize the geometries of all stationary points. We performed vibrational frequency calculations to verify that stationary points were minima on the potential energy surface, and to calculate thermal corrections to Gibbs free energies at 298.15 K (25˚C, see the *Thermochemical data* section).

Electronic energies were refined with single point energy calculations at the M06-2X/def2-TZVPP[48,49] level. QHA corrections to G calculated at the M06-2X/6-31+G(d,p) level of theory were included to these single point energies using the *GoodVibes*[50] program (see the *Thermochemical data* section).

Solvent effects were considered in all calculations using the integral equation formalism variant of the polarizable continuum model (IEF-PCM) with the SMD solvation model (solvent = acetonitrile).[51–56]

In all the reaction steps, a manual conformational search was performed. In this process, the different OH groups of the catalyst were rotated, and different interactions were generated in the substrate···catalyst, substrate···H_2O and catalyst···H_2O aggregates. The *GoodVibes* software was used to calculate the Boltzmann-weighted relative energy of each reaction step.

Gaussian[57] was employed for all density functional theory (DFT) and time-dependent (TD) DFT calculations. *PyMol*[58] was used to create molecular graphics with the display settings created by Prof. Robert Paton.[59]

AQME[60] jobs

1. Ensuring that there were no errors in the *Gaussian* output files with the following command line:
python -m aqme --qcorr --files "*.log"

2. Generating inputs of single-point energy calculations using their optimization outputs:

For ground state (S_0) systems: python -m aqme --qprep --files "*.log" --qm_input "m062x def2tzvpp scrf=(smd,solvent=acetonitrile)" --suffix SPC --program gaussian --mem 16GB --nprocs 8)

For excited state (S_1) systems: python -m aqme --qprep --files "*.log" --qm_input "td=(nstates=1) m062x def2tzvpp scrf=(smd,solvent=acetonitrile)" --suffix SPC --program gaussian --mem 16GB --nprocs 8)

Thermochemical data

Using the *GoodVibes* program, quasi-harmonic (QHA) corrections were introduced to the computed vibrational entropies using a frequency cut-off value of 100.0 cm^{-1}, following the model proposed by Grimme[61] at 298.15K. Also, a correction for the change in standard state from gas phase at 1 atm to a 1 M solution was introduced (option "-c 1" in *GoodVibes*). Entropy corrections due to entropy of symmetry (option "--ssym") was also included.[62–64] Relative G values (ΔG) were also calculated with *GoodVibes* (option "--pes").

All the thermochemical data including absolute energies, zero-point energies (ZPE) and T·S, among other parameters, at the M06-2X/6-31+G(d,p) level, as well as the absolute and relative G including the M06-2X/def2-TZVPP single point energy corrections (option "--spc"), were generated in an automated way using *GoodVibes* and tabulated below. This process for creating G profiles in an automated manner provides a

useful method to avoid errors related to human manipulation of the data. The keyword input line used in *GoodVibes* was “python -m goodvibes --ssym --pes 0_PES.yaml --xyz -c 1 --imag --spc SPC”.

GoodVibes information and input line:

GoodVibes v3.0.2 2025/11/21 22:10:49
Citation: Luchini, G.; Alegre-Requena, J. V.; Funes-Ardoiz, I.; Paton, R. S. F1000Research, 2020, 9, 291.
GoodVibes version 3.0.2 DOI: 10.12688/f1000research.22758.1

Requested: --ssym --pes 0_PES.yaml --xyz -c 1 --imag --spc SPC

Temperature = 298.15 Kelvin   Concentration = 1.0 mol/L
All energetic values below shown in Hartree unless otherwise specified.

Using vibrational scale factor 1.0: differing levels of theory detected.

Caution! Implicit solvation (SMD/CPCM) detected. Enthalpic and entropic terms cannot be safely separated. Use them at your own risk!

Entropic quasi-harmonic treatment: frequency cut-off value of 100.0 wavenumbers will be applied.
QS = Grimme: Using a mixture of RRHO and Free-rotor vibrational entropies.
REF: Grimme, S. Chem. Eur. J. 2012, 18, 9955-9964

Ssymm requested. Symmetry contribution to entropy to be calculated using S. Patchkovskii's open source software "Brute Force Symmetry Analyzer" available under GNU General Public License.
REF: (C) 1996, 2003 S. Patchkovskii, Serguei.Patchkovskii@sympatico.ca

Atomic radii used to calculate internal symmetry based on Cambridge Structural Database covalent radii.
REF: C. R. Groom, I. J. Bruno, M. P. Lightfoot and S. C. Ward, Acta Cryst. 2016, B72, 171-179
Cordero, B.; Gomez V.; Platero-Prats, A. E.; Reves, M.; Echeverria, J.; Cremades, E.; Barragan, F.; Alvarez, S. Dalton Trans. 2008, 2832-2838

Combining final single point energy with thermal corrections.

Structure	E_SPC	E	ZPE	H_SPC	T.S	T.qh-S	G(T)_SPC	qh-G(T)_SPC	im freq	Point Group
PyO-anionrad-H_2O_conf1	-399.968142	-399.82508	0.112324	-399.846	0.043177	0.041423	-399.889	-399.88725		C1
PyO-anionrad-H_2O_conf2	-399.968803	-399.825565	0.112811	-399.846	0.042442	0.040834	-399.889	-399.88703		C1
PyO-anionrad-H_2O_conf3	-399.968807	-399.825564	0.112875	-399.846	0.042036	0.040627	-399.888	-399.88681		C1
PyO-anionrad	-323.513242	-323.403052	0.087741	-323.418	0.035793	0.03465	-323.454	-323.45287		C2v
PyO-H_2O_conf1	-399.898847	-399.754593	0.117847	-399.772	0.041665	0.039782	-399.813	-399.81138		C1
PyO	-323.451247	-323.339884	0.093768	-323.352	0.030976	0.03098	-323.382	-323.38249		C2v
PyOH-anion-H_2O_conf1	-400.651164	-400.512456	0.124531	-400.516	0.044337	0.042611	-400.56	-400.55847		C1
PyOH-anion-H_2O_conf2	-400.651184	-400.512365	0.124513	-400.516	0.044157	0.042519	-400.56	-400.55844		C1
PyOH-anion	-324.183406	-324.080525	0.099659	-324.075	0.039532	0.038219	-324.114	-324.11318		Cs
PyOH-cation-H_2O_conf1	-400.337177	-400.191852	0.129242	-400.197	0.043899	0.042304	-400.241	-400.23938		C1
PyOH-cation-H_2O_conf2	-400.337141	-400.191856	0.128902	-400.197	0.045263	0.043186	-400.242	-400.24033		C1
PyOH-cation-H_2O_conf3	-400.348807	-400.203196	0.130195	-400.209	0.041344	0.039837	-400.25	-400.24881		C1
PyOH-cation	-323.896466	-323.782763	0.106198	-323.784	0.032683	0.032691	-323.816	-323.8164		Cs
PyOH-rad-H_2O_conf1	-400.453743	-400.308331	0.126815	-400.317	0.042755	0.041561	-400.359	-400.35812		C1
PyOH-rad-H_2O_conf4	-400.447524	-400.301544	0.125517	-400.311	0.04463	0.043279	-400.356	-400.3542		Cs
PyOH-rad	-324.00592	-323.892745	0.102378	-323.897	0.034165	0.034124	-323.931	-323.9307		Cs
$PyOH_2$-cationrad-H_2O_conf1	-400.913967	-400.768679	0.136097	-400.765	0.047815	0.046092	-400.813	-400.81156		C1
$PyOH_2$-cationrad-H_2O_conf2	-400.919306	-400.77449	0.137915	-400.77	0.045457	0.043833	-400.816	-400.81391		C1
$PyOH_2$-cationrad-H_2O_conf3	-400.913969	-400.768679	0.136111	-400.765	0.047786	0.046073	-400.813	-400.81154		C1
$PyOH_2$-cationrad-H_2O_conf4	-400.913327	-400.767784	0.135845	-400.765	0.048327	0.046448	-400.813	-400.81137		C1
$PyOH_2$-cationrad	-324.470544	-324.357495	0.112662	-324.349	0.038485	0.037874	-324.388	-324.38712		Cs
$PyOH_2$-H_2O_conf1	-401.169201	-401.024172	0.138098	-401.019	0.04916	0.045454	-401.068	-401.06466		C1
$PyOH_2$-H_2O_conf2	-401.168976	-401.023973	0.138128	-401.019	0.049047	0.045579	-401.068	-401.06459		C1
$PyOH_2$-H_2O_conf3	-401.168698	-401.023867	0.137925	-401.019	0.050434	0.046247	-401.069	-401.06502		C1
$PyOH_2$-H_2O_conf4	-401.169202	-401.024172	0.138089	-401.019	0.049262	0.045501	-401.068	-401.06471		C1
$PyOH_2$	-324.723367	-324.610412	0.113493	-324.601	0.039893	0.038045	-324.641	-324.63915		C1
S_0-H_2PQ-cationrad-H_2O_conf1	-766.229807	-765.965253	0.227831	-765.985	0.058355	0.056339	-766.043	-766.04126		C1
S_0-H_2PQ-cationrad-H_2O_conf2	-766.229642	-765.965967	0.227494	-765.985	0.059593	0.057293	-766.044	-766.04208		C1
S_0-H_2PQ-cationrad-H_2O_conf3	-766.228641	-765.964768	0.22839	-765.983	0.058206	0.05631	-766.042	-766.03962		C1

S_0-H_2PQ-cationrad-H_2O_conf4	-766.241709	-765.97851	0.228811	-765.997	0.054912	0.053591	-766.052	-766.05075		C1
S_0-H_2PQ-cationrad-H_2O_conf5	-766.241705	-765.978509	0.228542	-765.997	0.055543	0.053946	-766.053	-766.05125		C1
S_0-H_2PQ-cationrad-H_2O_conf6	-766.242026	-765.979049	0.228777	-765.997	0.057084	0.054644	-766.055	-766.05209		C1
S_0-H_2PQ-cationrad	-689.790119	-689.558222	0.204659	-689.573	0.048114	0.047561	-689.621	-689.62018		C1
S_0-HPQ-cation-H_2O_conf1	-765.614083	-765.350912	0.216343	-765.381	0.059401	0.05648	-765.44	-765.43726		C1
S_0-HPQ-cation-H_2O_conf2	-765.615385	-765.352318	0.21731	-765.382	0.057316	0.054817	-765.439	-765.43644		Cs
S_0-HPQ-cation-H_2O_conf3	-765.630435	-765.369064	0.21676	-765.399	0.052612	0.051465	-765.451	-765.45016		C1
S_0-HPQ-cation-H_2O_conf4	-765.61436	-765.351513	0.216742	-765.381	0.060787	0.056856	-765.442	-765.43758		Cs
S_0-HPQ-cation-H_2O_conf5	-765.615385	-765.352318	0.217312	-765.382	0.057304	0.054812	-765.439	-765.43644		Cs
S_0-HPQ-cation	-689.175359	-688.944456	0.193659	-688.969	0.046804	0.046229	-689.016	-689.01557		C1
S_0-HPQ-rad-H_2O_conf1	-765.807269	-765.545002	0.215577	-765.575	0.058047	0.05561	-765.633	-765.6307		C1
S_0-HPQ-rad-H_2O_conf2	-765.814719	-765.553022	0.216693	-765.583	0.054786	0.053314	-765.637	-765.63584		C1
S_0-HPQ-rad-H_2O_conf3	-765.806341	-765.544394	0.216126	-765.574	0.057917	0.055437	-765.632	-765.62928		Cs
S_0-HPQ-rad-H_2O_conf4	-765.810566	-765.548503	0.216933	-765.578	0.056415	0.054305	-765.634	-765.63203		Cs
S_0-HPQ-rad-H_2O_conf5	-765.807284	-765.544999	0.215884	-765.575	0.058026	0.055375	-765.633	-765.63038		C1
S_0-HPQ-rad-H_2O_conf6	-765.805759	-765.544176	0.215422	-765.573	0.060467	0.057205	-765.634	-765.63057		C1
S_0-HPQ-rad	-689.366772	-689.137031	0.192224	-689.162	0.049323	0.047726	-689.211	-689.20973		Cs
S_0-PQ-anionrad-H_2O_conf1	-765.34296	-765.08335	0.203847	-765.124	0.056156	0.053844	-765.18	-765.17741		Cs
S_0-PQ-anionrad-H_2O_conf2	-765.34745	-765.088424	0.204028	-765.128	0.05543	0.053344	-765.183	-765.18139		Cs
S_0-PQ-anionrad-H_2O_conf3	-765.342962	-765.083322	0.203797	-765.124	0.056341	0.053952	-765.18	-765.17756		C1
S_0-PQ-anionrad-H_2O_conf4	-765.347453	-765.088431	0.204048	-765.128	0.055304	0.053278	-765.183	-765.18132		Cs
S_0-PQ-anionrad	-688.895239	-688.668167	0.17921	-688.704	0.046506	0.045851	-688.75	-688.7497		C2
S_0-PQ-H_2O_conf2	-765.207707	-764.947276	0.205538	-764.986	0.055861	0.053635	-765.042	-765.04001		C1
S_0-PQ-H_2O_conf4	-765.204997	-764.944118	0.205488	-764.984	0.056512	0.053948	-765.04	-765.03753		Cs
S_0-PQ	-688.763025	-688.534664	0.181405	-688.569	0.046262	0.045357	-688.616	-688.61478		C2
S_1-H_2PQ-H_2O_conf1	-766.30087	-766.03863	0.223752	-766.06	0.056879	0.055106	-766.117	-766.11536		C1
S_1-H_2PQ-H_2O_conf2	-766.301459	-766.03932	0.224205	-766.061	0.056978	0.055017	-766.118	-766.1156		C1
S_1-H_2PQ-H_2O_conf3	-766.292207	-766.029147	0.223497	-766.051	0.059472	0.056838	-766.111	-766.10794		C1
S_1-H_2PQ-H_2O_conf4	-766.29427	-766.030996	0.222644	-766.054	0.060216	0.057462	-766.114	-766.11109		C1
S_1-H_2PQ-H_2O_conf6	-766.291245	-766.028599	0.222578	-766.05	0.062033	0.058781	-766.112	-766.10907		C1
S_1-H_2PQ-H_2O_conf7	-766.294821	-766.031349	0.223202	-766.054	0.058789	0.056532	-766.113	-766.11053		C1
S_1-H_2PQ	-689.852609	-689.621922	0.19961	-689.639	0.050267	0.048891	-689.689	-689.68803		C1
S_1-HPQ-anion-H_2O_conf1	-765.854594	-765.593968	0.212427	-765.626	0.056911	0.054612	-765.683	-765.68034		Cs
S_1-HPQ-anion-H_2O_conf2	-765.854741	-765.593944	0.21235	-765.626	0.057284	0.054744	-765.683	-765.68063		C1
S_1-HPQ-anion-H_2O_conf4	-765.856516	-765.595742	0.212331	-765.628	0.055348	0.053755	-765.683	-765.68178		C1
S_1-HPQ-anion-H_2O_conf5	-765.847407	-765.586936	0.210938	-765.619	0.059416	0.056939	-765.678	-765.67593		C1
S_1-HPQ-anion	-689.409199	-689.180865	0.187834	-689.208	0.047873	0.047233	-689.256	-689.25559		Cs

Gconf correction requested to be applied to below relative values using quasi-harmonic Boltzmann factors

RXN: H^+ + e, step 1 (kcal/mol)	DE_SPC	DE	DZPE	DH_SPC	T.DS	T.qh-DS	DG(T)_SPC	qh-DG(T)_SPC
S_1-H_2PQ + PyO	0.0	0.0	0.0	0.0	0.0	0.0	0.0	0.0
S_1-HPQ-anion + PyOH-cation	-1.1	-1.1	0.4	-0.9	-0.4	0.0	-0.5	-0.9
S_0-HPQ-rad + PyOH-rad	-43.2	-42.7	0.8	-42.6	1.4	1.2	-44.0	-43.9

RXN: e + H^+, step 1 (kcal/mol)	DE_SPC	DE	DZPE	DH_SPC	T.DS	T.qh-DS	DG(T)_SPC	qh-DG(T)_SPC
S_1-H_2PQ + PyO	0.0	0.0	0.0	0.0	0.0	0.0	0.0	0.0
S_0-H_2PQ-cationrad + PyO-anionrad	0.3	0.3	-0.6	-0.1	1.7	1.5	-1.8	-1.6
S_0-HPQ-rad + PyOH-rad	-43.2	-42.7	0.8	-42.6	1.4	1.2	-44.0	-43.9

RXN: H^+ + e, step 2 (kcal/mol)	DE_SPC	DE	DZPE	DH_SPC	T.DS	T.qh-DS	DG(T)_SPC	qh-DG(T)_SPC
S_1-H_2PQ + PyO	0.0	0.0	0.0	0.0	0.0	0.0	0.0	0.0
S_0-PQ-anionrad + $PyOH_2$-cationrad	-38.9	-40.1	-0.9	-39.2	2.4	2.4	-41.5	-41.6
S_0-PQ + $PyOH_2$	-114.5	-115	1.0	-112.9	3.1	2.2	-116.0	-115.1

RXN: e + H^+, step 2 (kcal/mol)	DE_SPC	DE	DZPE	DH_SPC	T.DS	T.qh-DS	DG(T)_SPC	qh-DG(T)_SPC
S_1-H_2PQ + PyO	0.0	0.0	0.0	0.0	0.0	0.0	0.0	0.0
S_0-HPQ-cation + PyOH-anion	-34.5	-39.6	0.0	-33.7	3.2	2.9	-36.9	-36.5
S_0-PQ + $PyOH_2$	-114.5	-115	1.0	-112.9	3.1	2.2	-116.0	-115.1

RXN: (H_2O) H^+ + e, step 1 (kcal/mol)	DE_SPC	DE	DZPE	DH_SPC	T.DS	T.qh-DS	DG(T)_SPC	qh-DG(T)_SPC
S_1-H_2PQ-H_2O + PyO-H_2O	0.0	0.0	0.0	0.0	0.0	0.0	0.0	0.0
S_1-HPQ-anion-H_2O + PyOH-cation-H_2O	-2.9	-3.0	0.4	-2.7	-0.7	-0.5	-1.9	-2.2
S_0-HPQ-rad-H_2O + PyOH-rad-H_2O	-42.8	-42.4	1.0	-41.9	-0.9	-0.2	-41.0	-41.7

RXN: (H_2O) e + H^+, step 1 (kcal/mol)	DE_SPC	DE	DZPE	DH_SPC	T.DS	T.qh-DS	DG(T)_SPC	qh-DG(T)_SPC
S_1-H_2PQ-H_2O + PyO-H_2O	0.0	0.0	0.0	0.0	0.0	0.0	0.0	0.0
S_0-H_2PQ-cationrad-H_2O + PyO-anionrad-H_2O	-6.5	-6.7	-0.3	-7.2	1.0	1.1	-8.2	-8.2
S_0-HPQ-rad-H_2O + PyOH-rad-H_2O	-42.8	-42.4	1.0	-41.9	-0.9	-0.2	-41.0	-41.7

RXN: (H_2O) H^+ + e, step 2 (kcal/mol)	DE_SPC	DE	DZPE	DH_SPC	T.DS	T.qh-DS	DG(T)_SPC	qh-DG(T)_SPC
S_1-H_2PQ-H_2O + PyO-H_2O	0.0	0.0	0.0	0.0	0.0	0.0	0.0	0.0
S_0-PQ-anionrad-H_2O + PyOH_2-cationrad-H_2O	-41.2	-42.8	-0.2	-40.9	2.1	2.2	-43.0	-43.0
S_0-PQ-H_2O + PyOH_2-H_2O	-110.8	-111.4	1.1	-108.7	4.8	3.4	-113.5	-112.1

RXN: (H_2O) e + H^+, step 2 (kcal/mol)	DE_SPC	DE	DZPE	DH_SPC	T.DS	T.qh-DS	DG(T)_SPC	qh-DG(T)_SPC
S_1-H_2PQ-H_2O + PyO-H_2O	0.0	0.0	0.0	0.0	0.0	0.0	0.0	0.0
S_0-HPQ-cation-H_2O + PyOH-anion-H_2O	-51.2	-55.2	-0.4	-51.8	-1.1	-0.5	-50.7	-51.3
S_0-PQ-H_2O + PyOH_2-H_2O	-110.8	-111.4	1.1	-108.7	4.8	3.4	-113.5	-112.1

Molecular orbital analysis in S_1

In all the geometries considered to calculate ΔG in the excited S_1 state, this state majorly corresponds to the HOMO–LUMO transition as observed in their TD-DFT outputs:

Optimized geometry in H_2PQ*:

Excited State 1: Singlet-A 3.5033 eV 353.91 nm f=0.0563 <S**2>=0.000

54 -> 57 0.17822
55 -> 56 0.67791 *(HOMO–LUMO)*

Optimized geometry in HPQ^-* (anion formed after proton transfer):

Excited State 1: Singlet-A 2.5488 eV 486.43 nm f=0.0628 <S**2>=0.000

55 -> 56 0.70272 *(HOMO–LUMO)*

The HOMO–LUMO transitions involve the following π → π* excitations:

Optimized geometry in H_2PQ*:

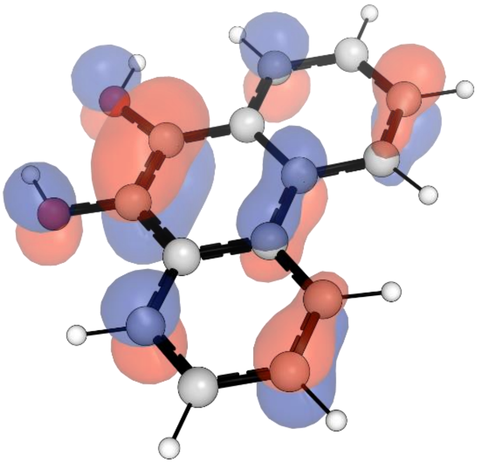

HOMO = π of H_2PQ

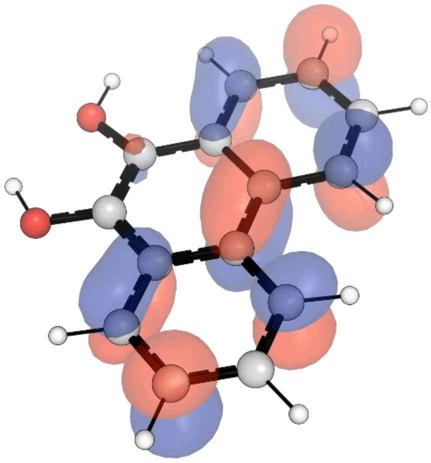

LUMO = π* of H_2PQ

Figure S20: HOMO and LUMO of geometry optimized H_2PQ.

Optimized geometry in HPQ⁻* (anion formed after proton transfer):

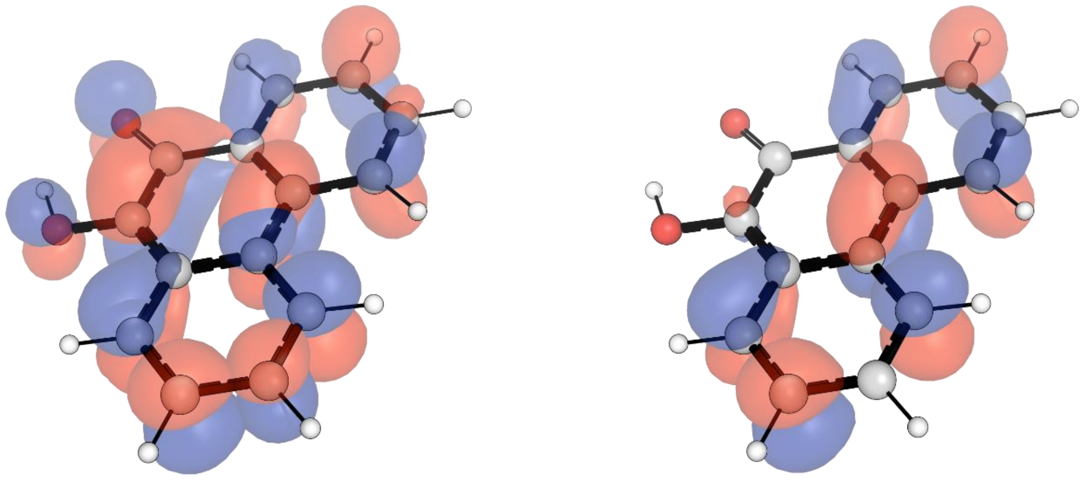

HOMO = π of HPQ⁻ LUMO = π* of HPQ⁻

Figure S21: HOMO and LUMO of geometry optimized HPQ⁻.

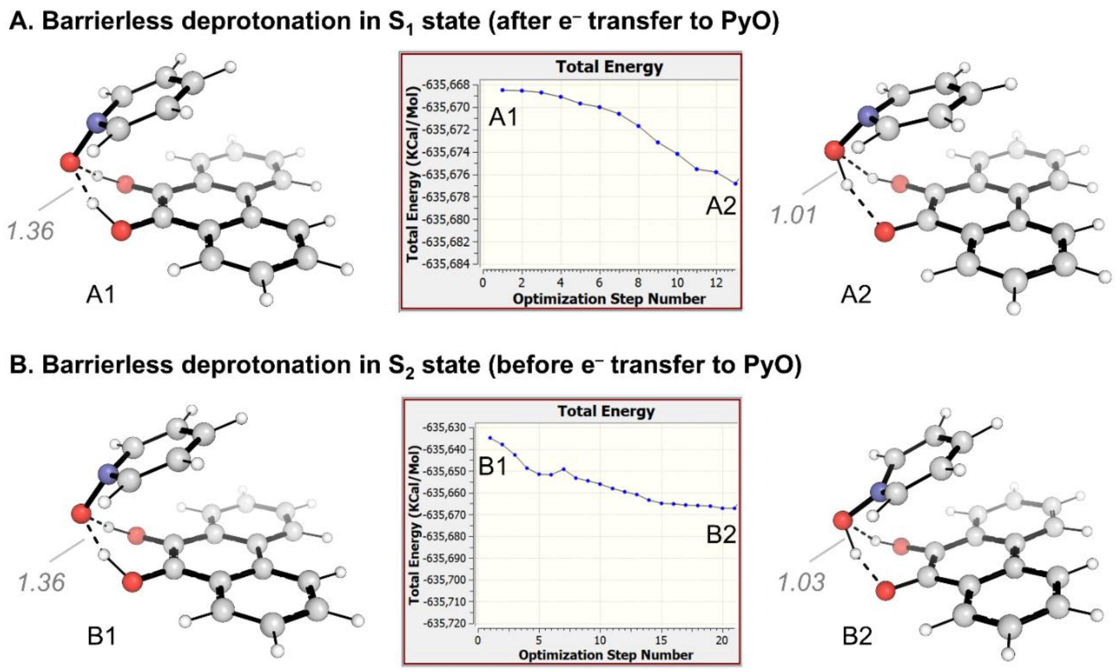

Figure S22: Computed barrierless deprotonation of H_2PQ in the first step A) after e⁻ transfer to PyO and B) before e⁻ transfer to PyO.

The proposed mechanism

The full catalytic cycle was constructed based on experimental and computational studies. The full proposed mechanictic cycle is illustrated in Figure S23.

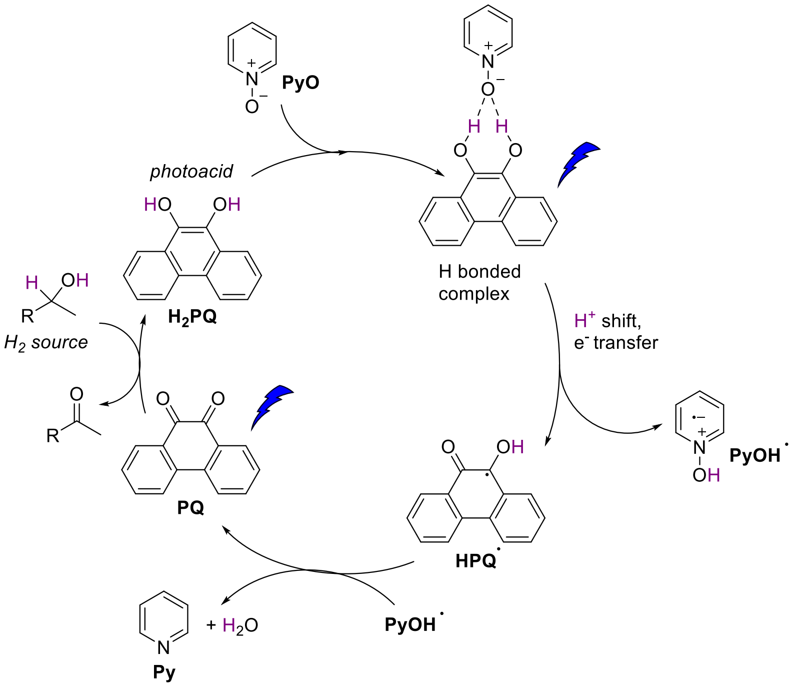

Figure S23: The proposed mechanism.

Copies of NMR spectra

^{1}H NMR (400 MHz, $CDCl_3$) of **S3**:

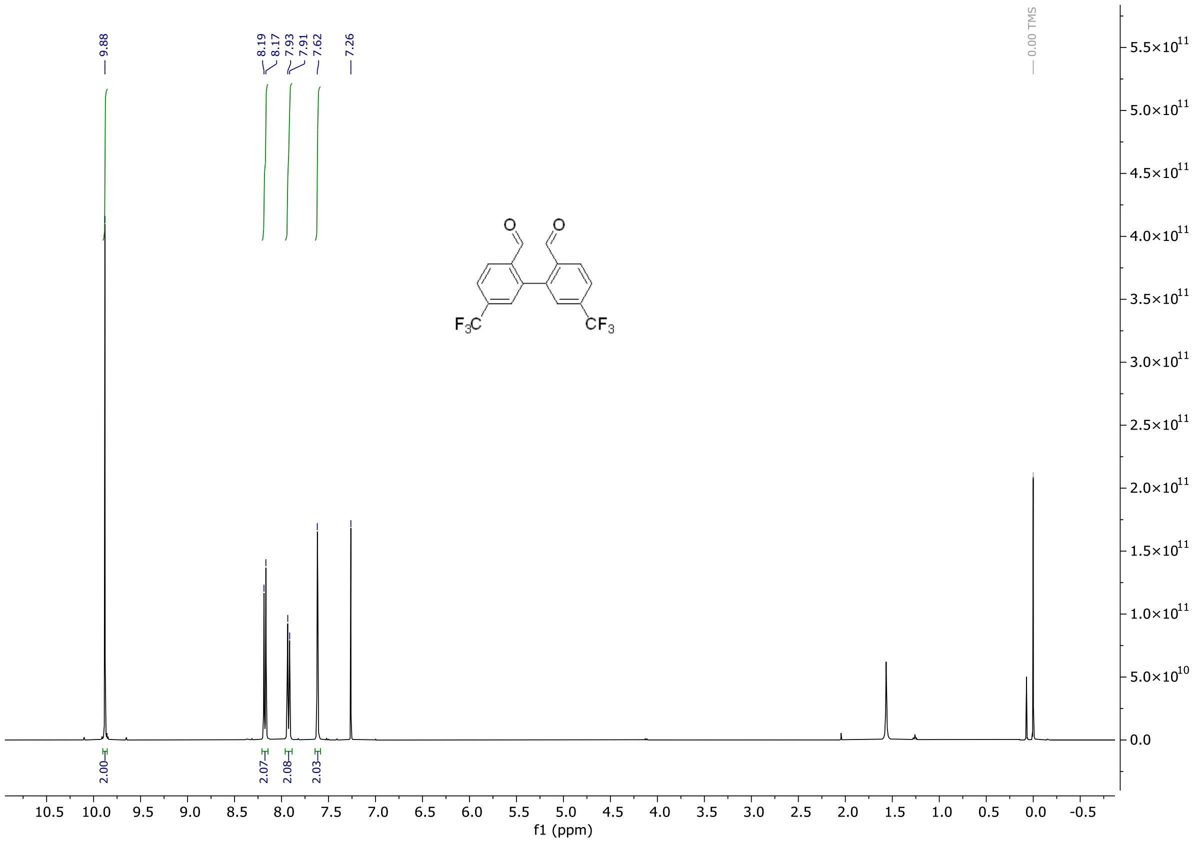

^{13}C NMR (101 MHz, $CDCl_3$) of **S3**:

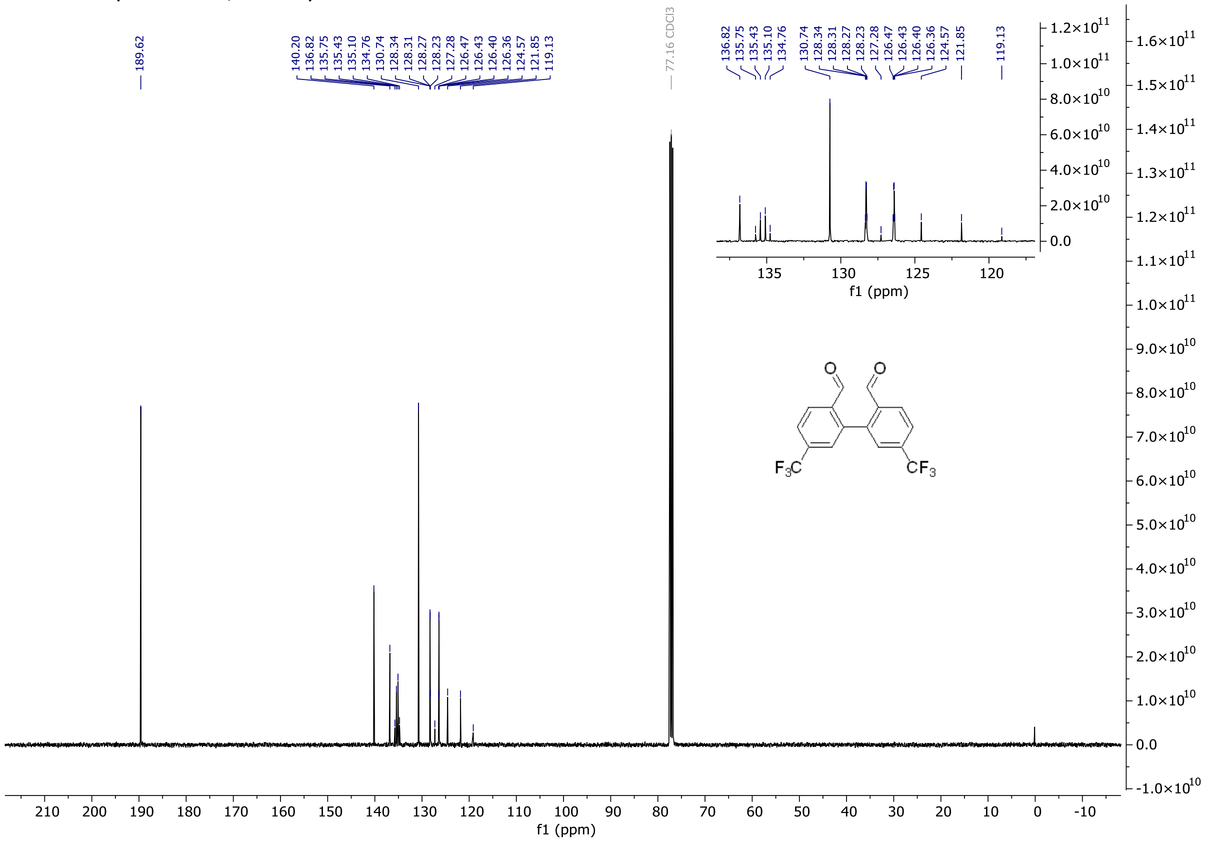

^{1}H NMR (400 MHz, $CDCl_3$) of PQ-CF_3:

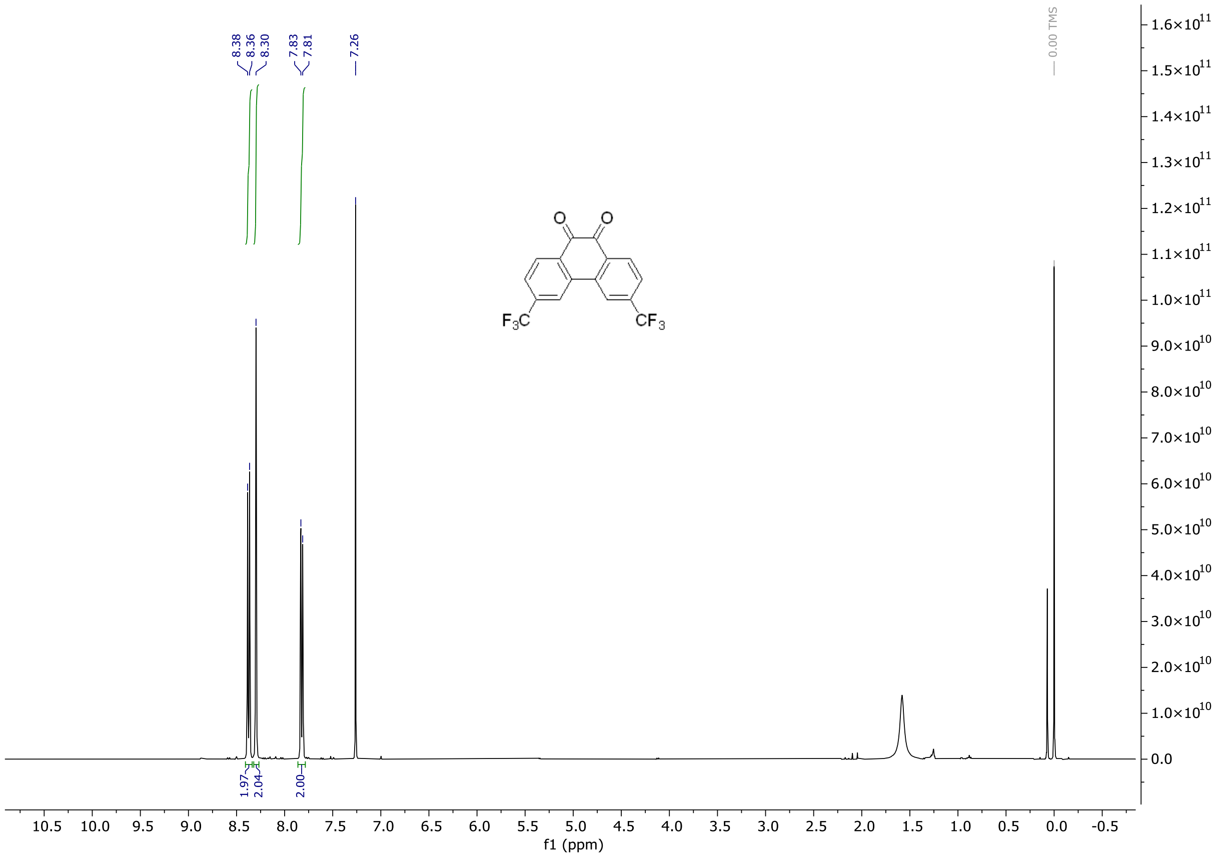

^{13}C NMR (101 MHz, $CDCl_3$) of PQ-CF_3:

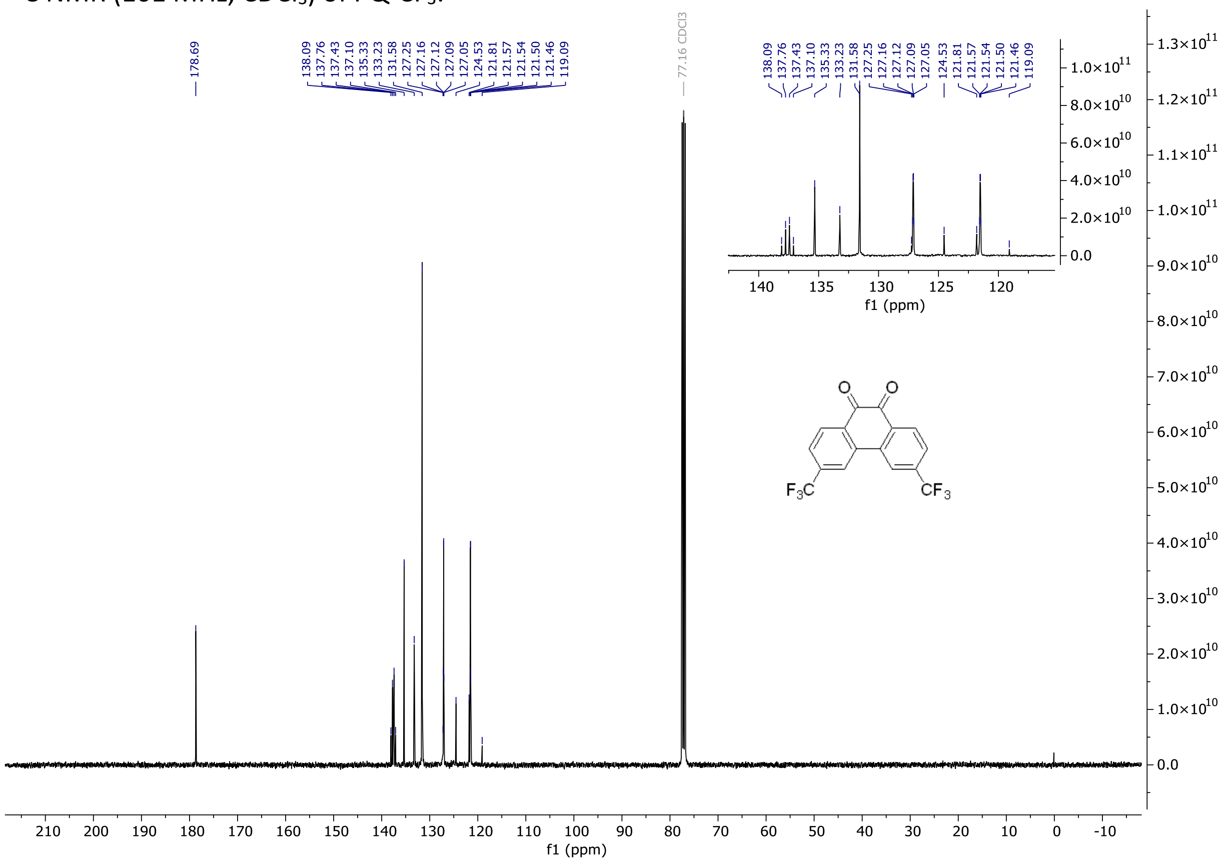

^{1}H NMR (400 MHz, $CDCl_3$) of **1a**:

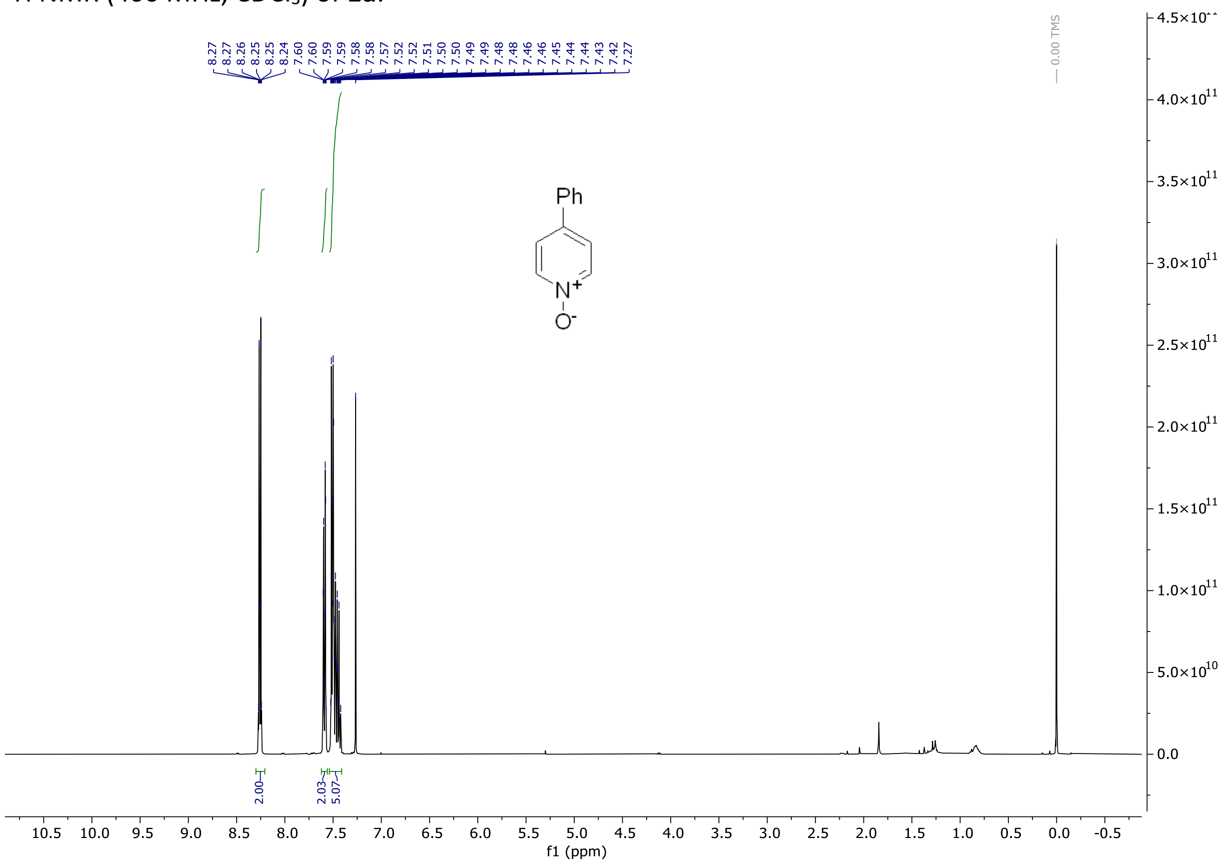

^{13}C NMR (101 MHz, $CDCl_3$) of **1a**:

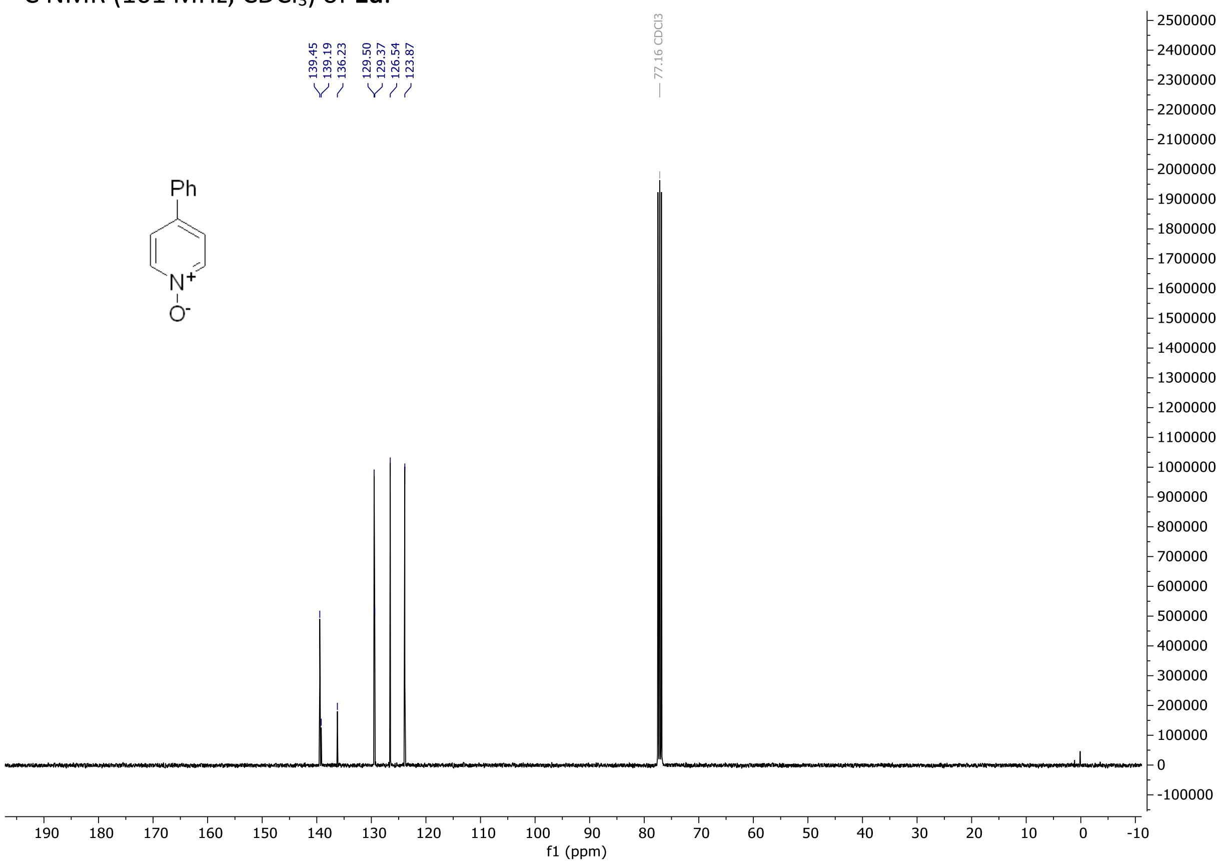

^{1}H NMR (400 MHz, $CDCl_3$) of **1c**:

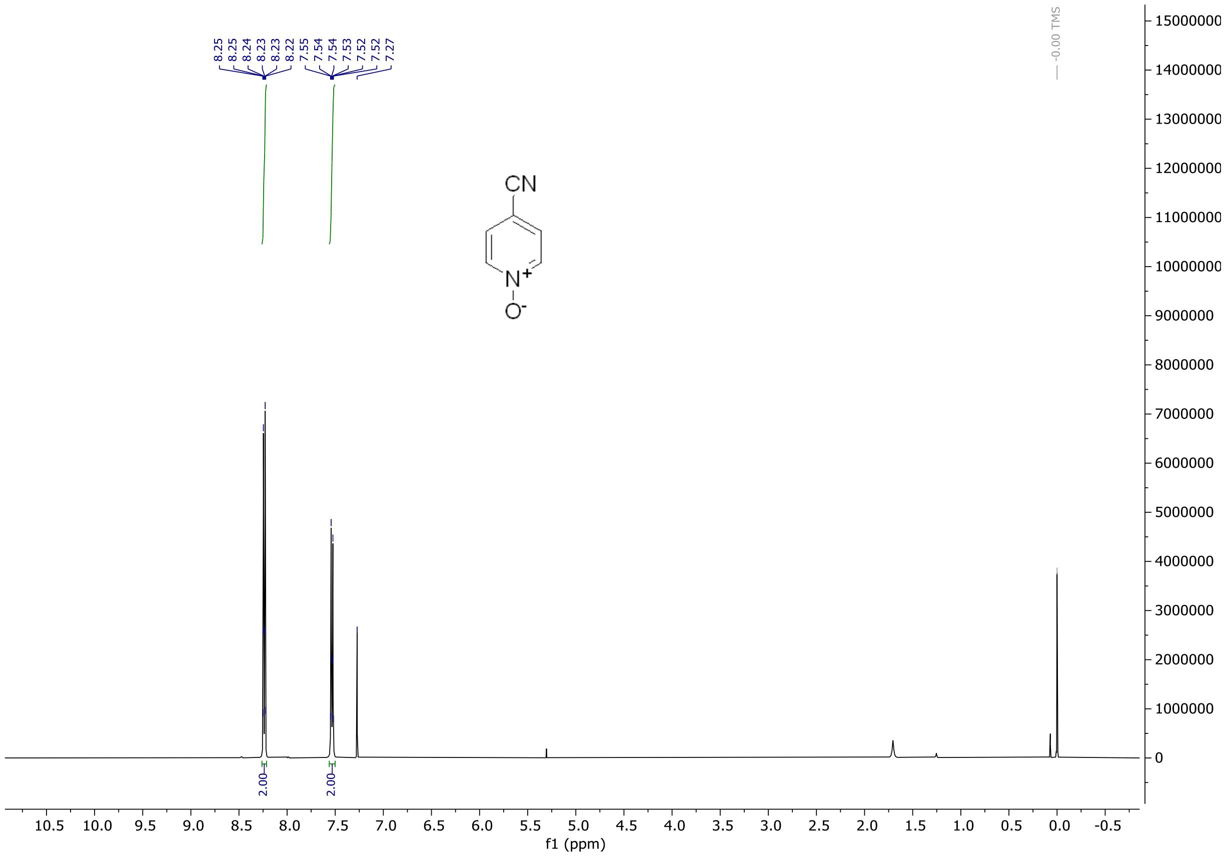

^{13}C NMR (101 MHz, $CDCl_3$) of **1c**:

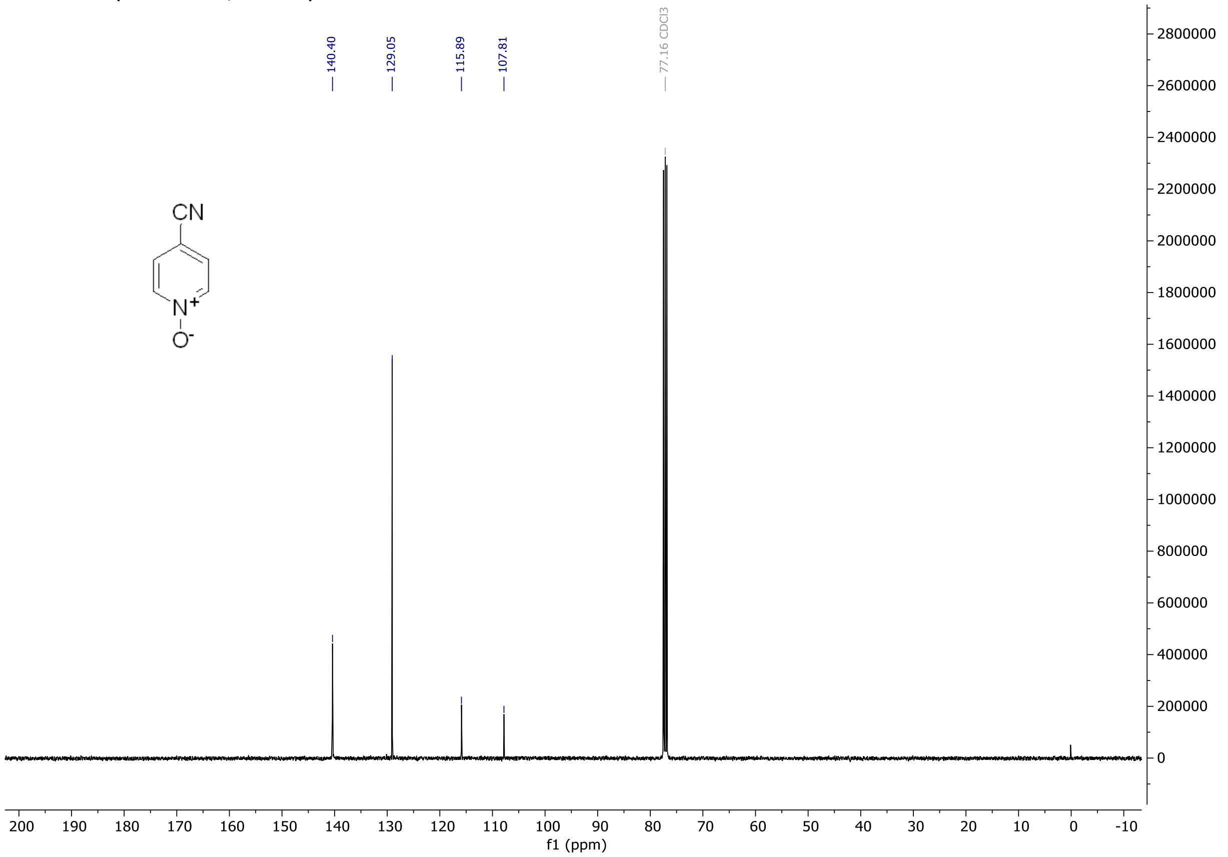

^1H NMR (400 MHz, $CDCl_3$) of **1e**:

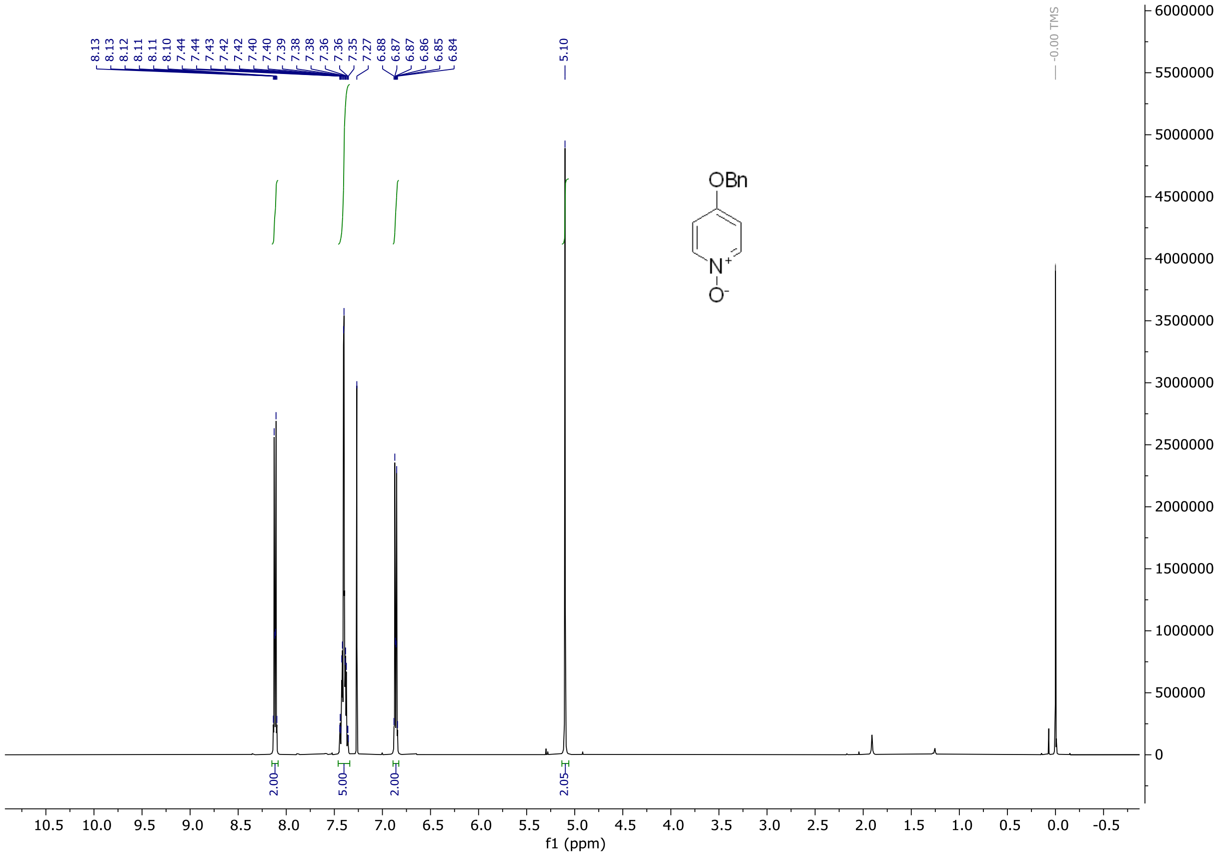

^{13}C NMR (101 MHz, $CDCl_3$) of **1e**:

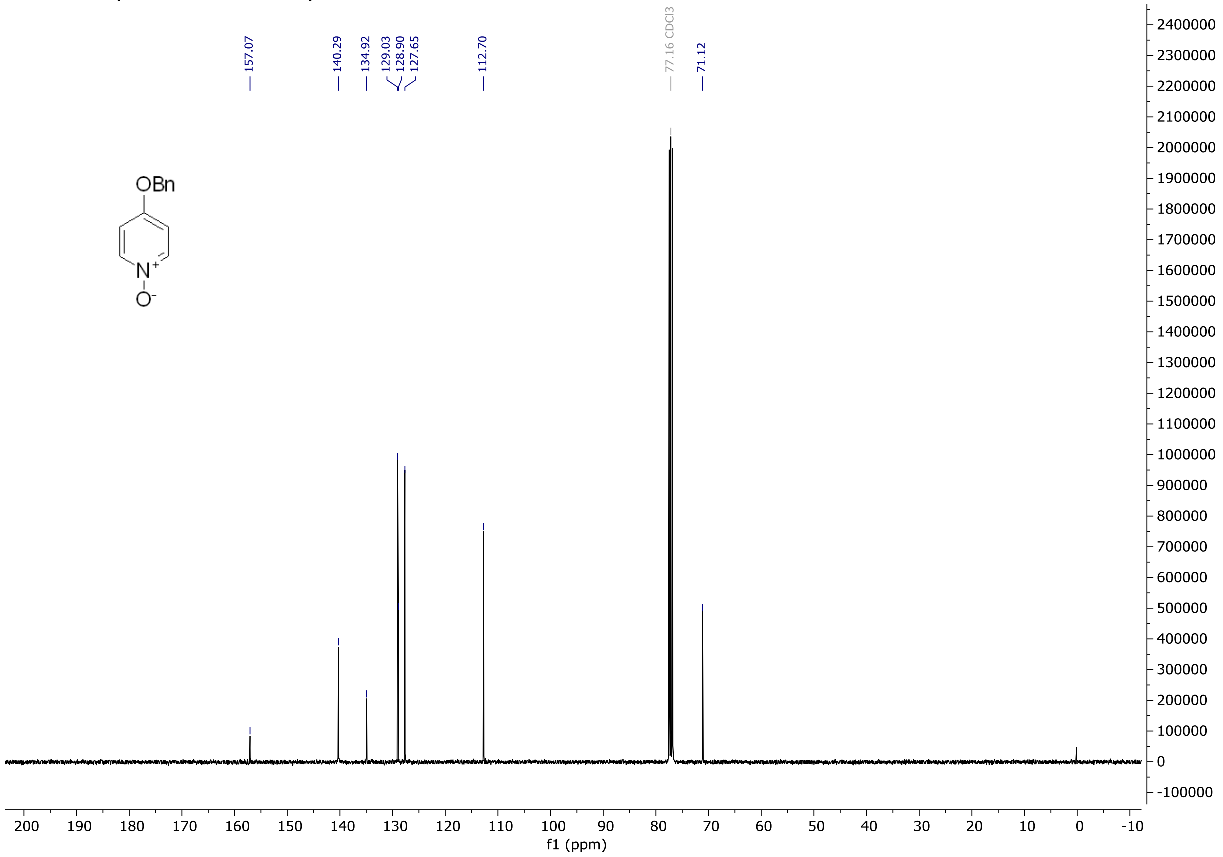

^{1}H NMR (400 MHz, $CDCl_3$) of **1f**:

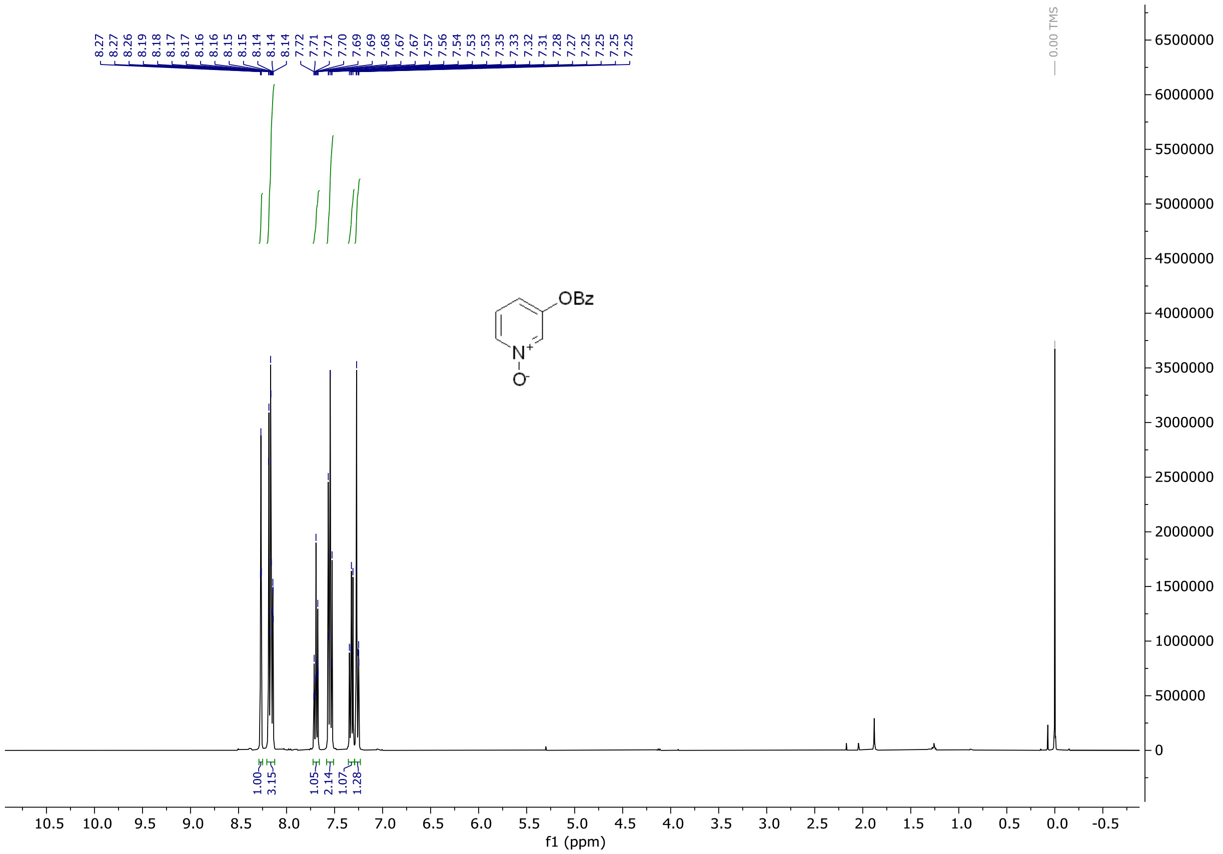

^{13}C NMR (101 MHz, $CDCl_3$) of **1f**:

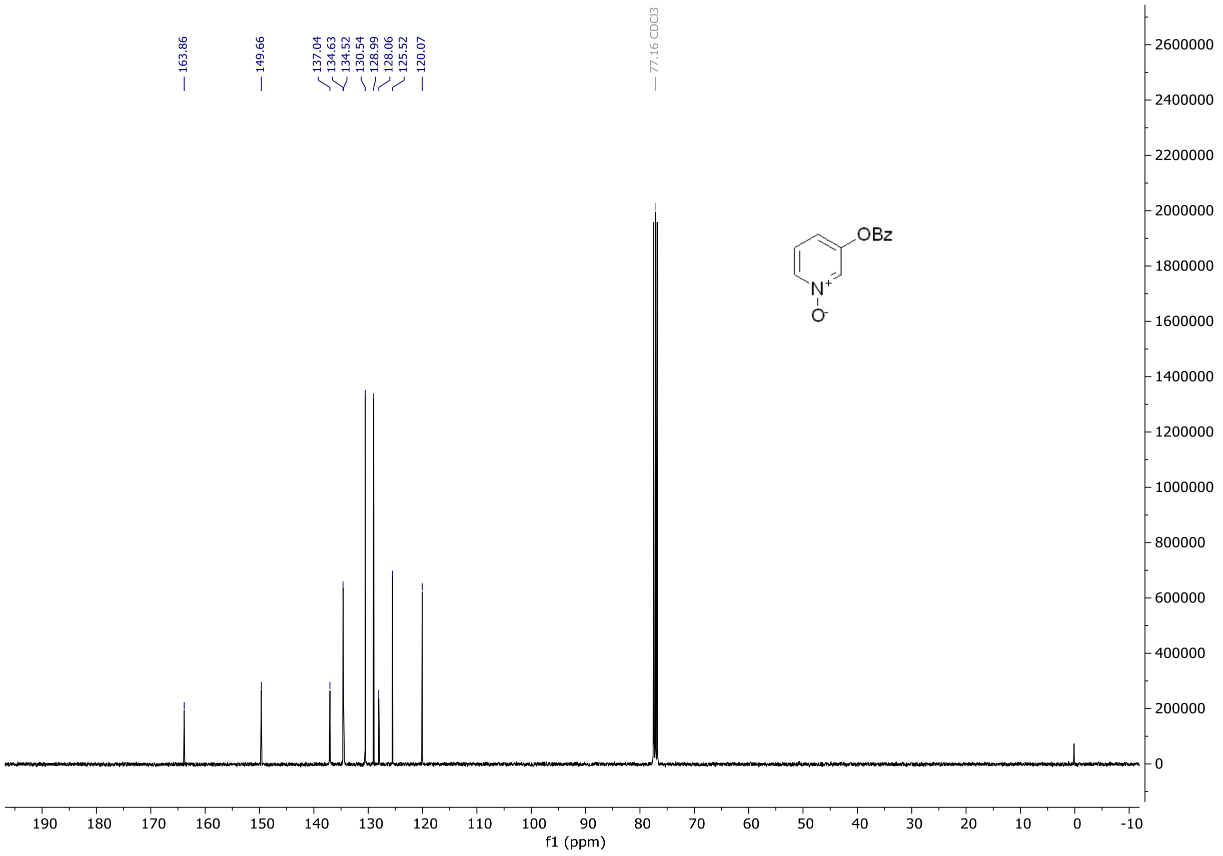

^{1}H NMR (400 MHz, $CDCl_3$) of **1g**:

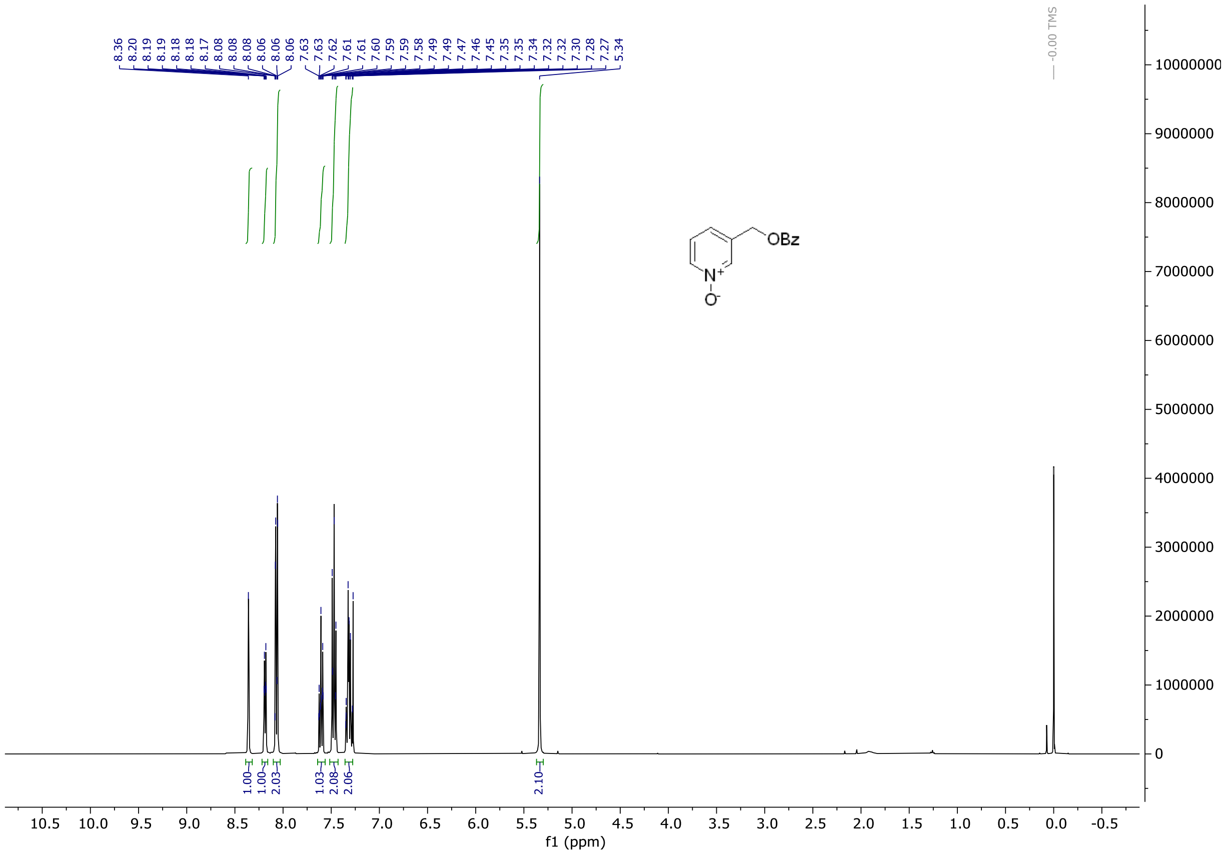

^{13}C NMR (101 MHz, $CDCl_3$) of **1g**:

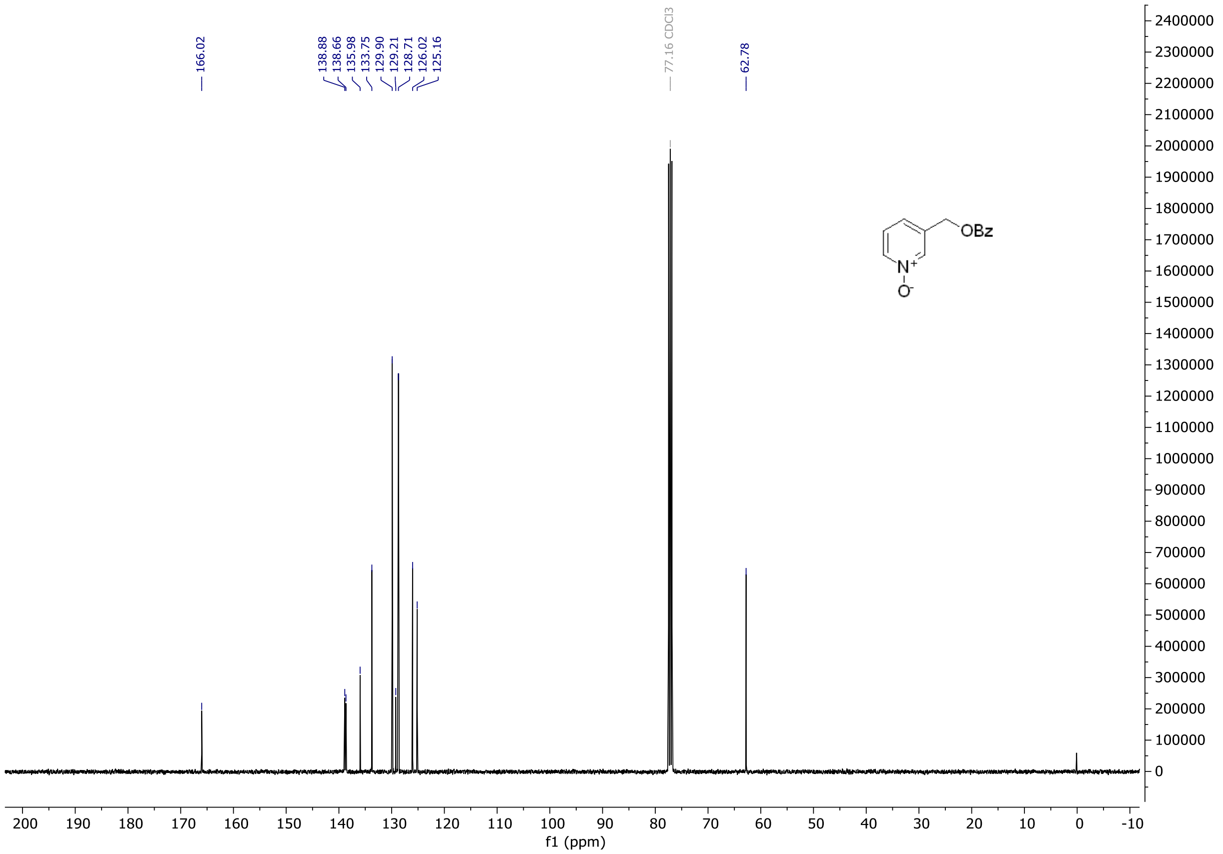

^{1}H NMR (400 MHz, $CDCl_3$) of **1k**:

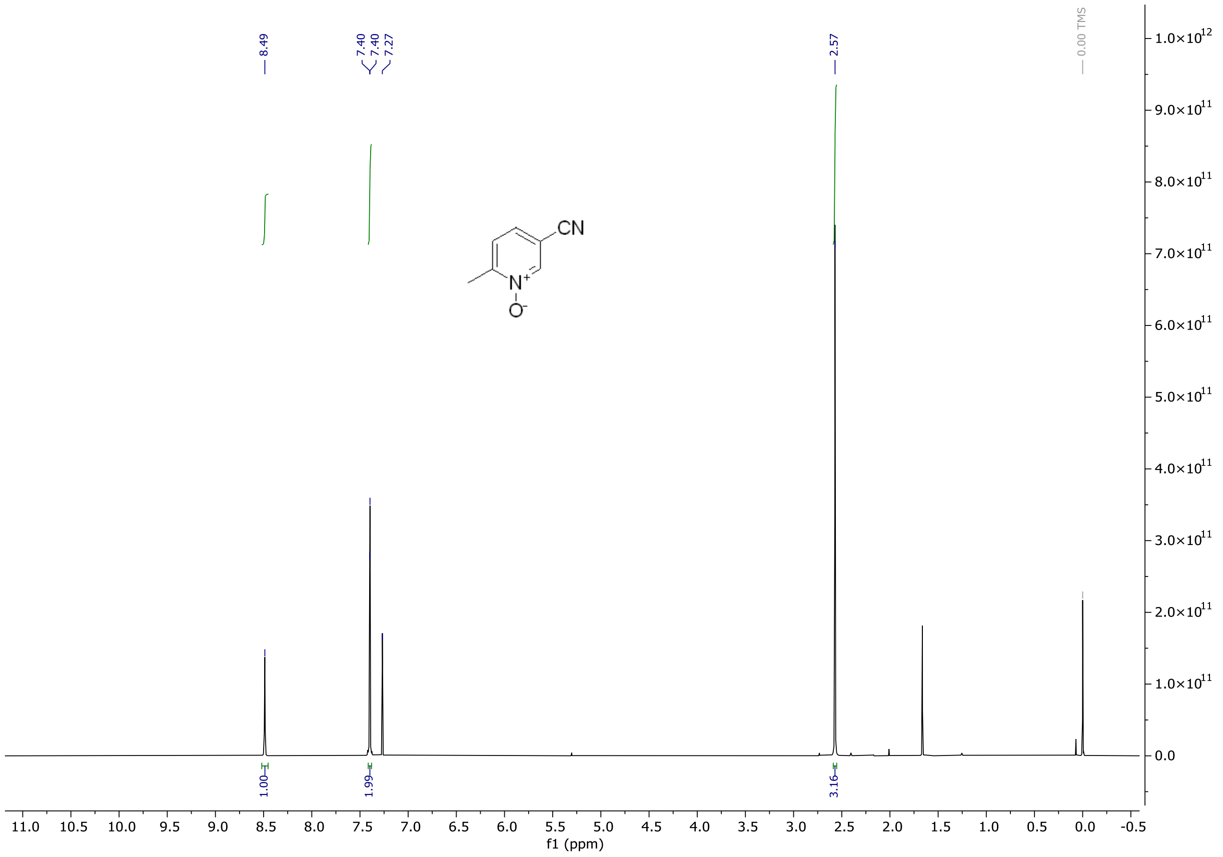

^{13}C NMR (101 MHz, $CDCl_3$) of **1k**:

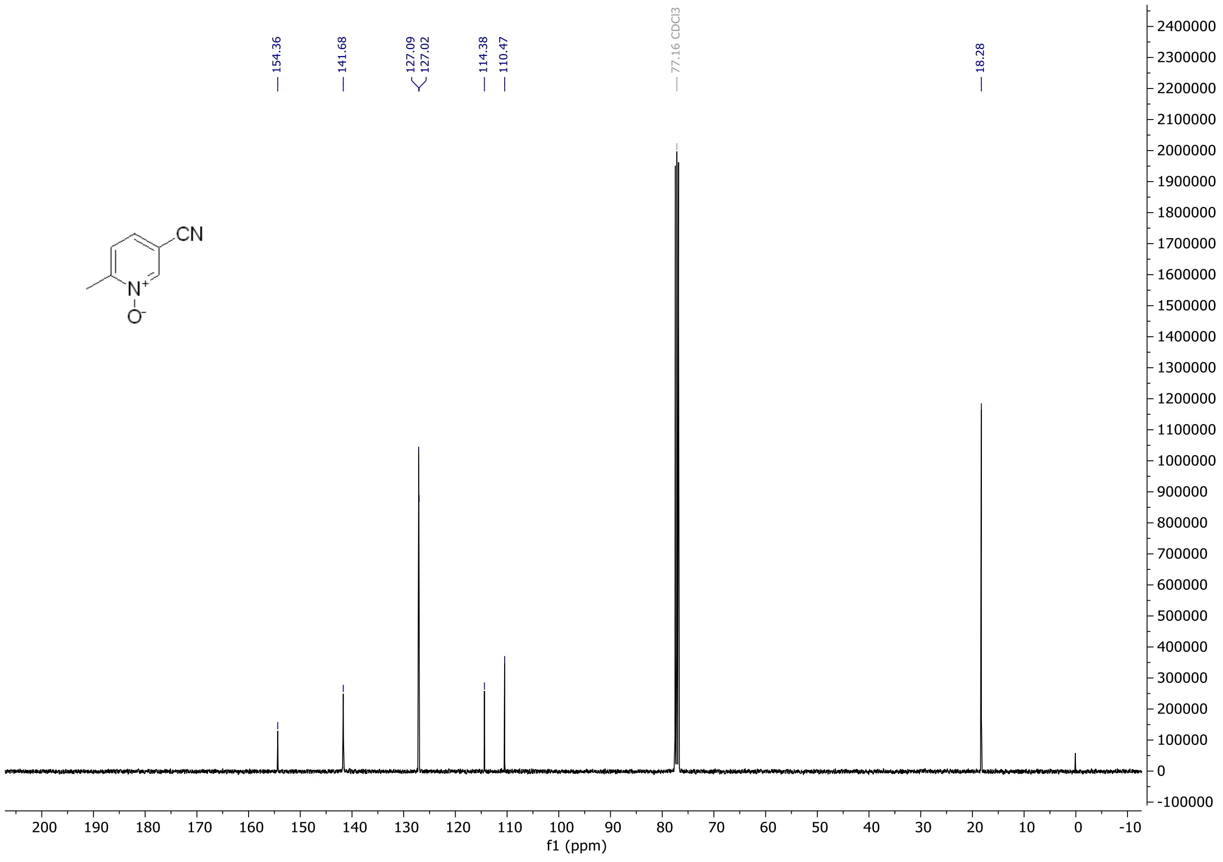

^{1}H NMR (400 MHz, $CDCl_3$) of **S4**:

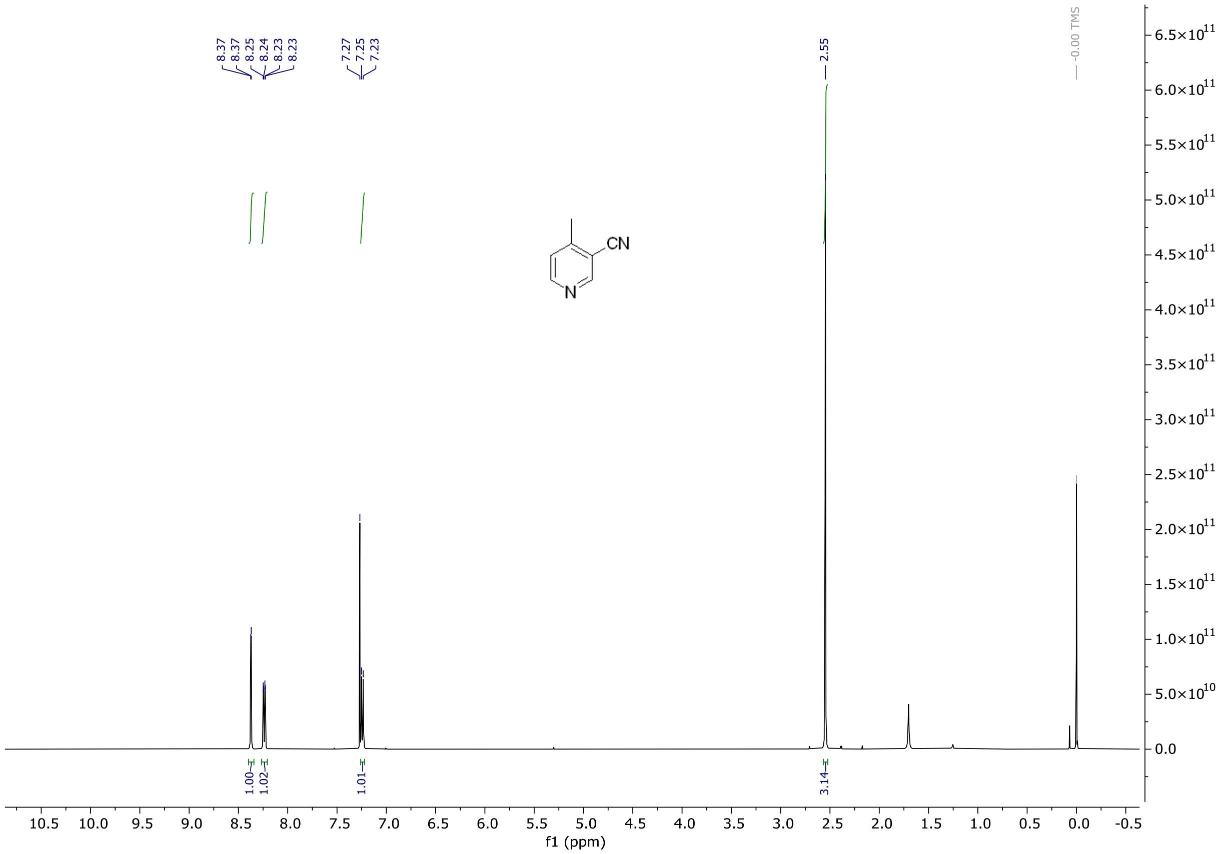

^{13}C NMR (101 MHz, $CDCl_3$) of **S4**:

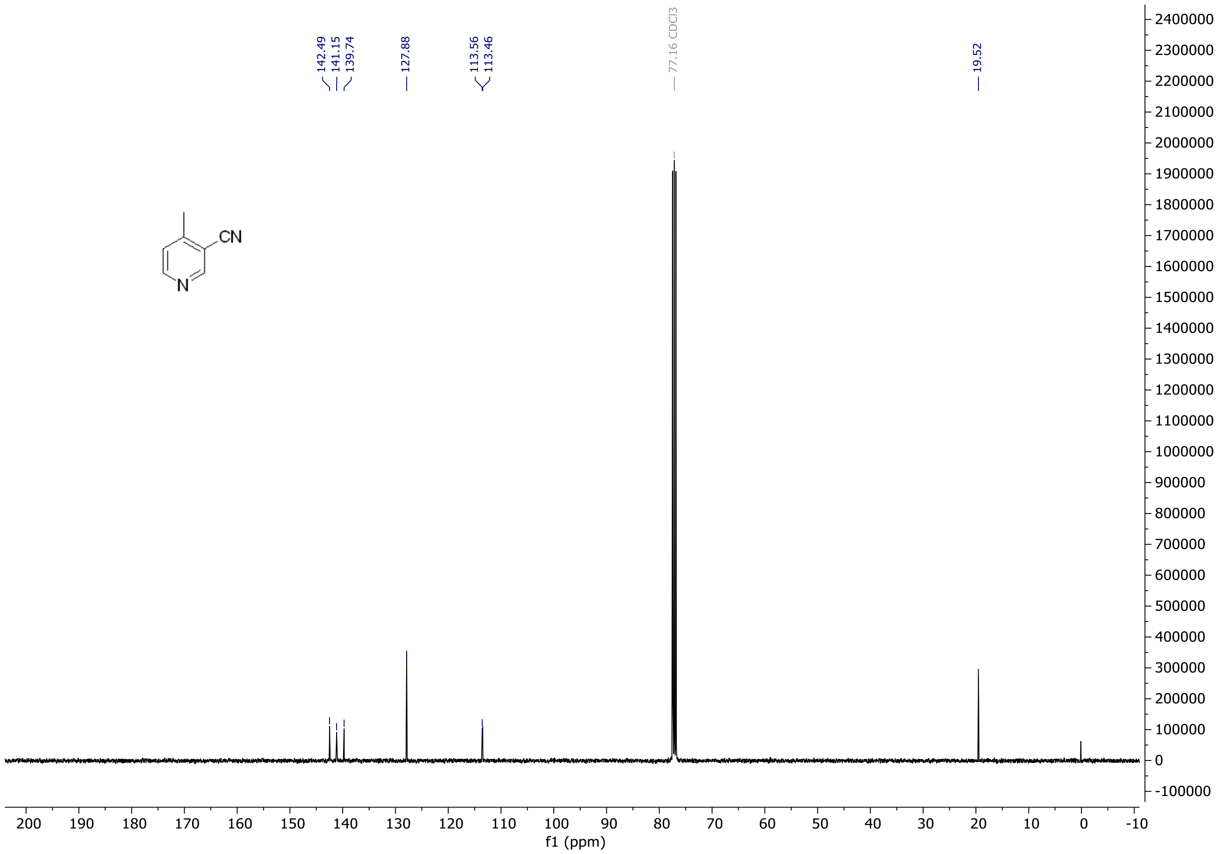

^{1}H NMR (400 MHz, $CDCl_3$) of **S5**:

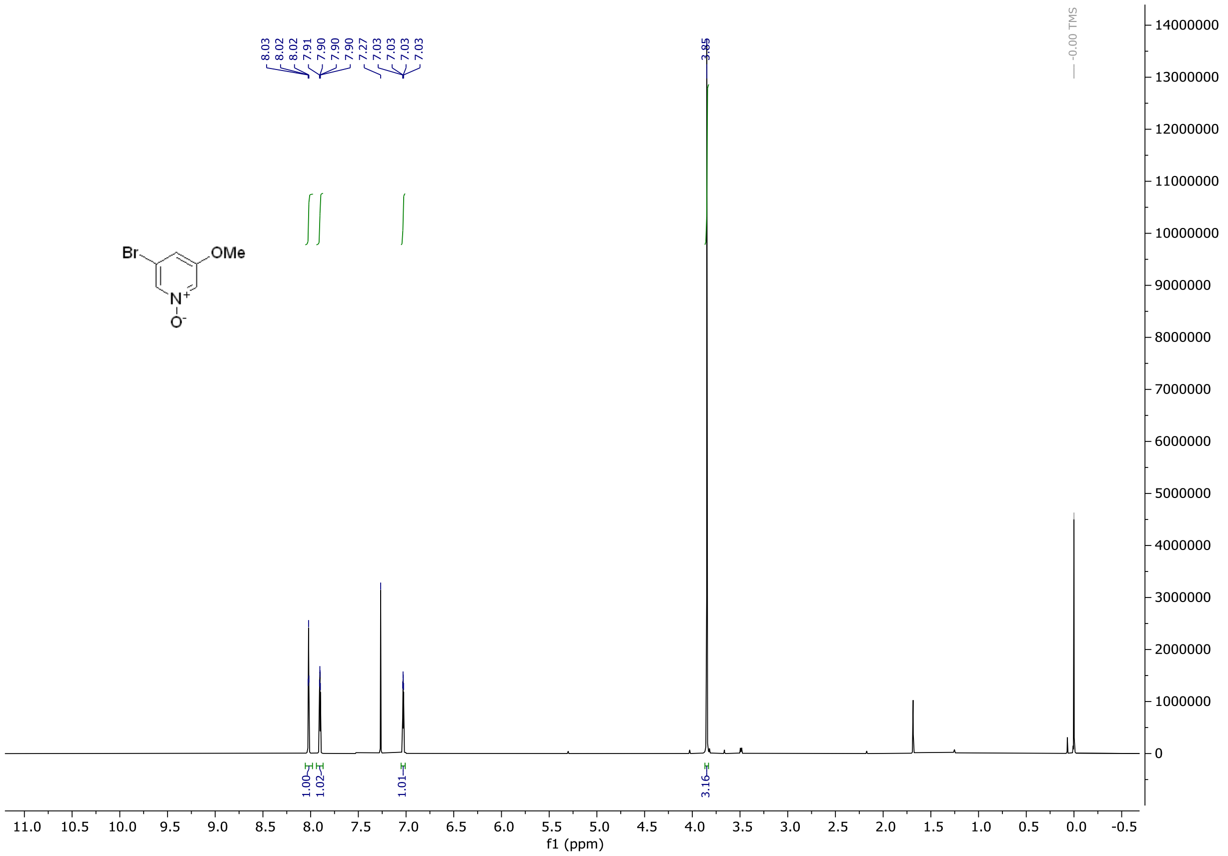

^{13}C NMR (101 MHz, $CDCl_3$) of **S5**:

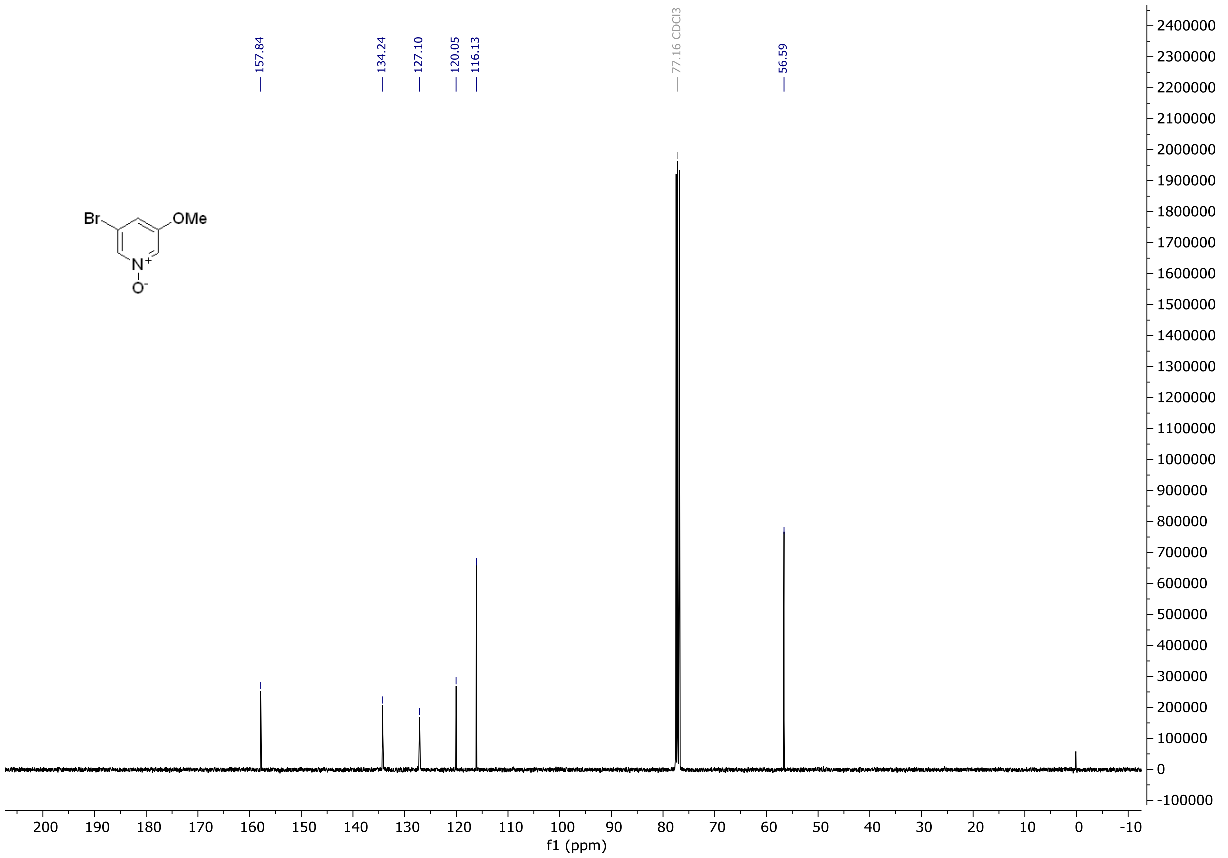

^{1}H NMR (400 MHz, $CDCl_3$) of **S6**:

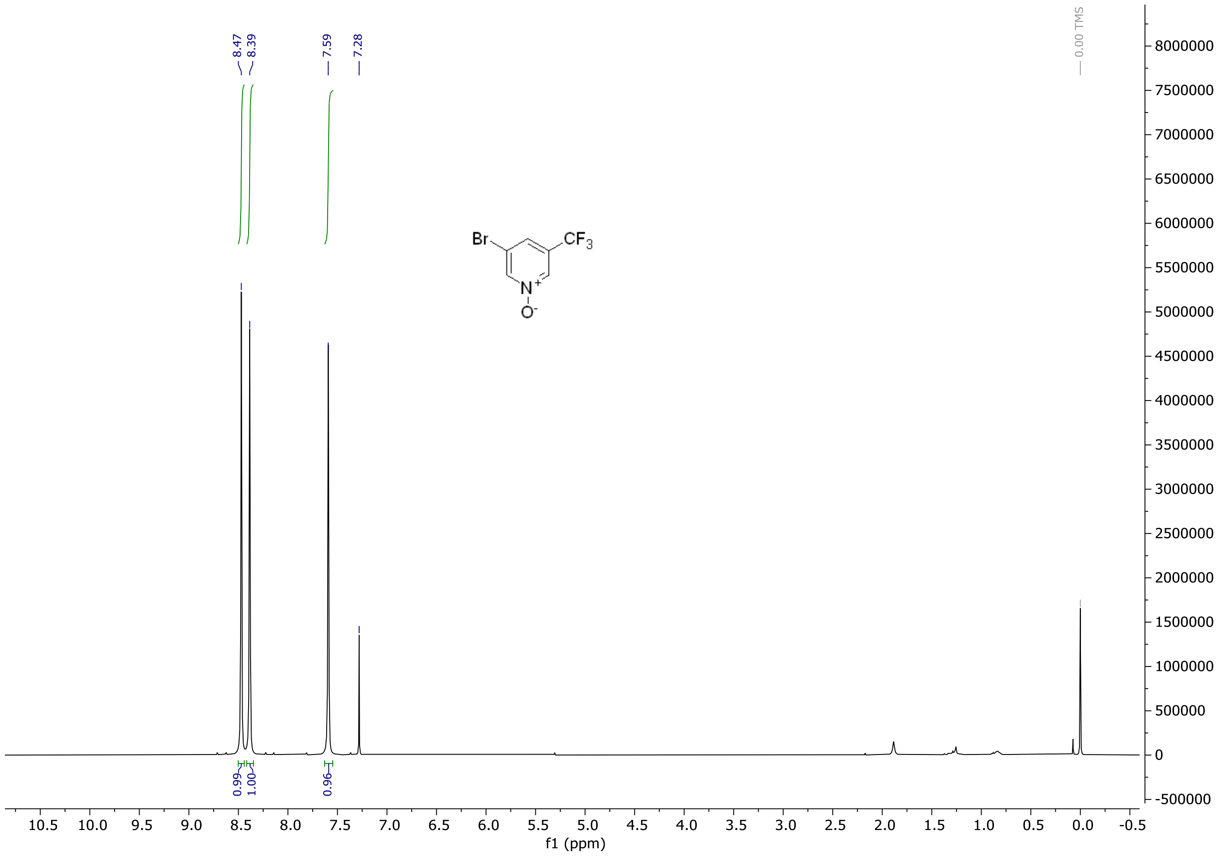

^{13}C NMR (101 MHz, $CDCl_3$) of **S6**:

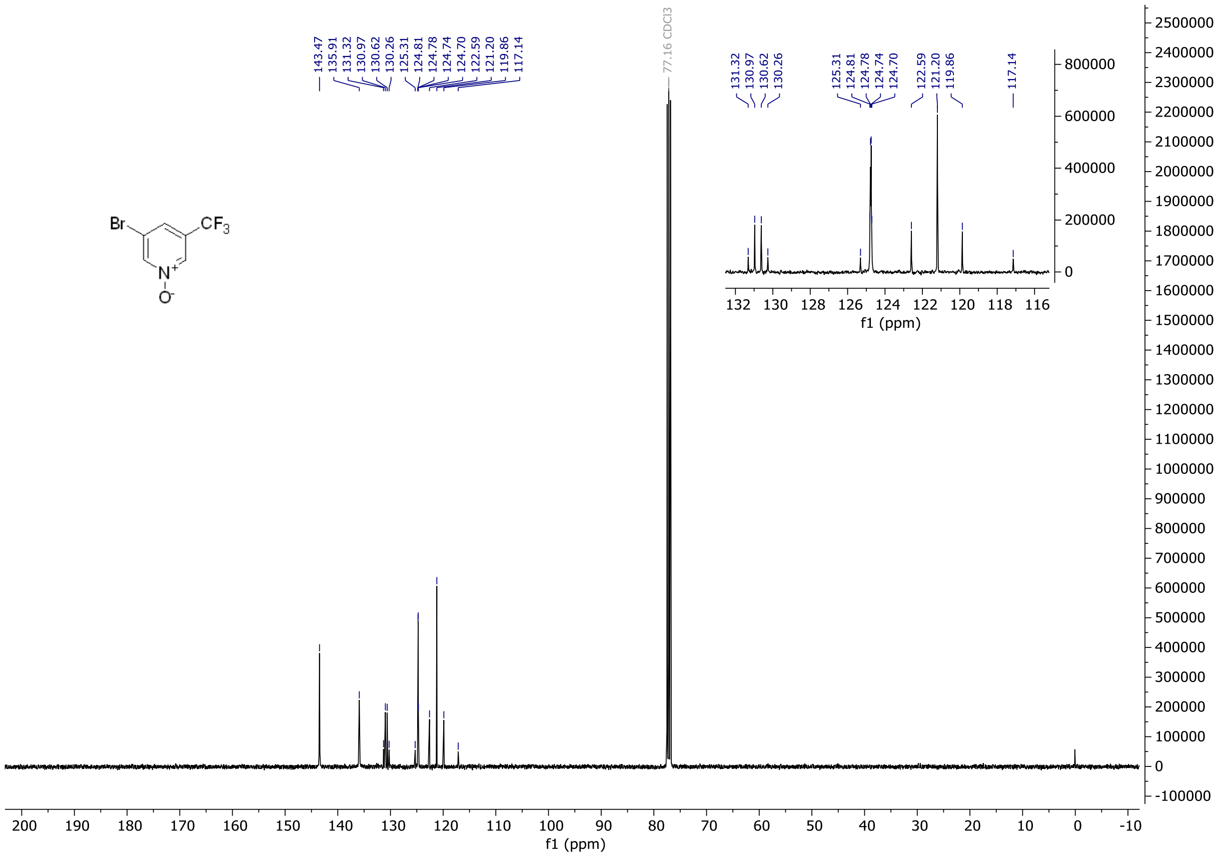

^{19}F NMR (376 MHz, $CDCl_3$) of **S6**:

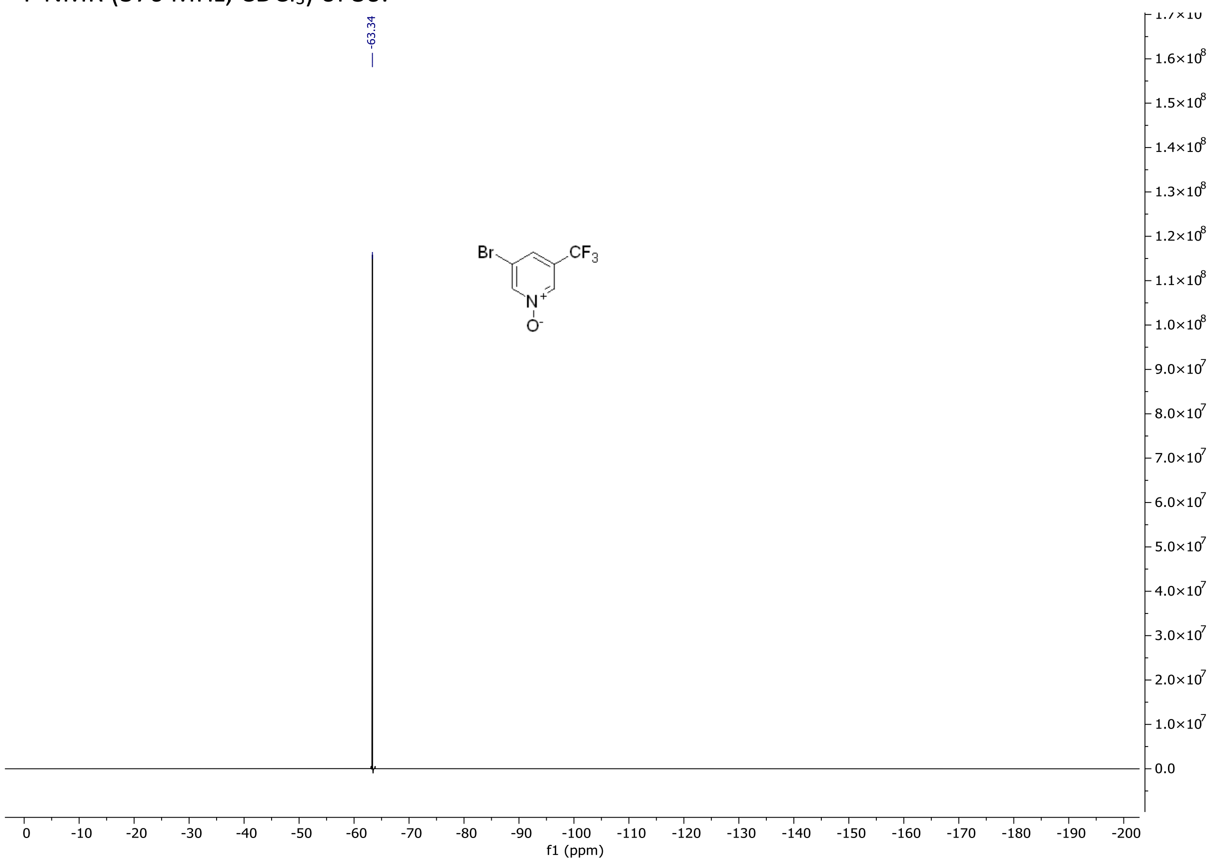

^{1}H NMR (400 MHz, DMSO-d_6) of **S8**:

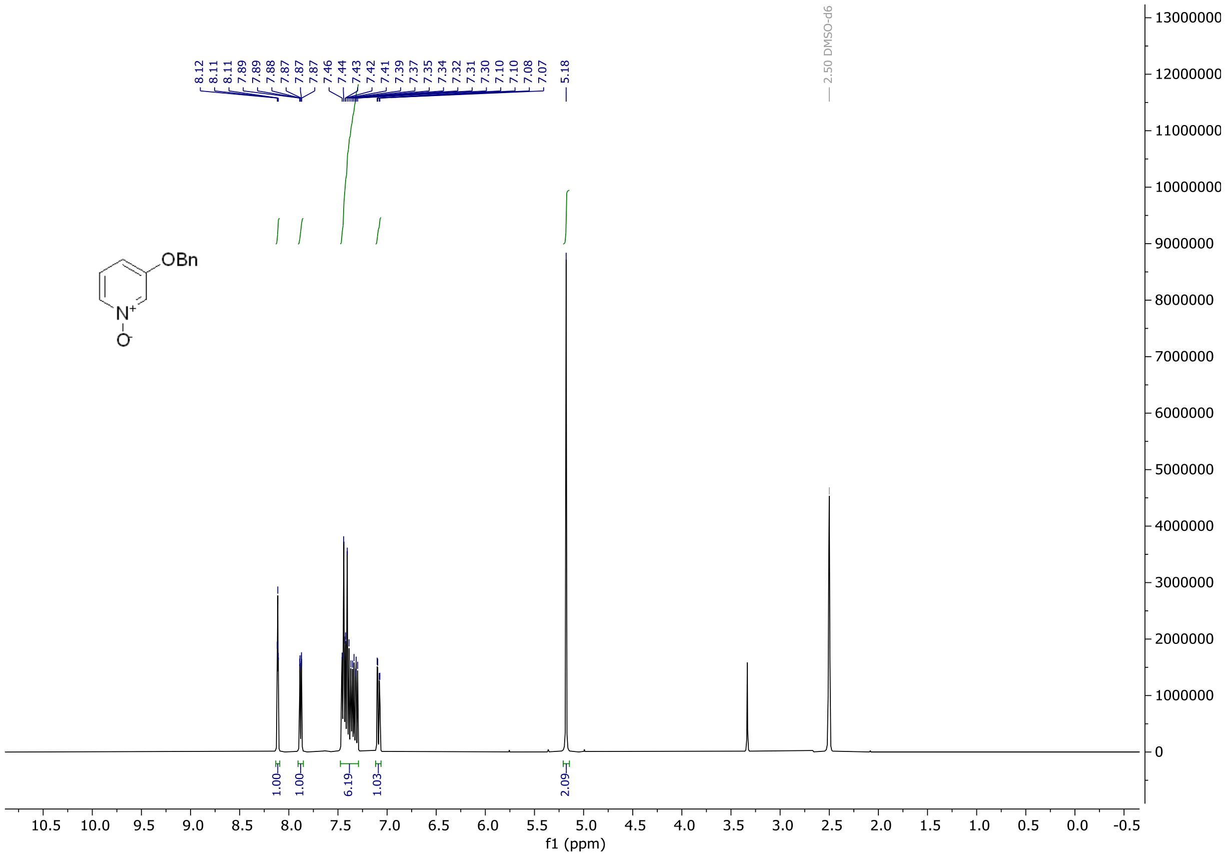

^{13}C NMR (101 MHz, DMSO-d_6) of **S8**:

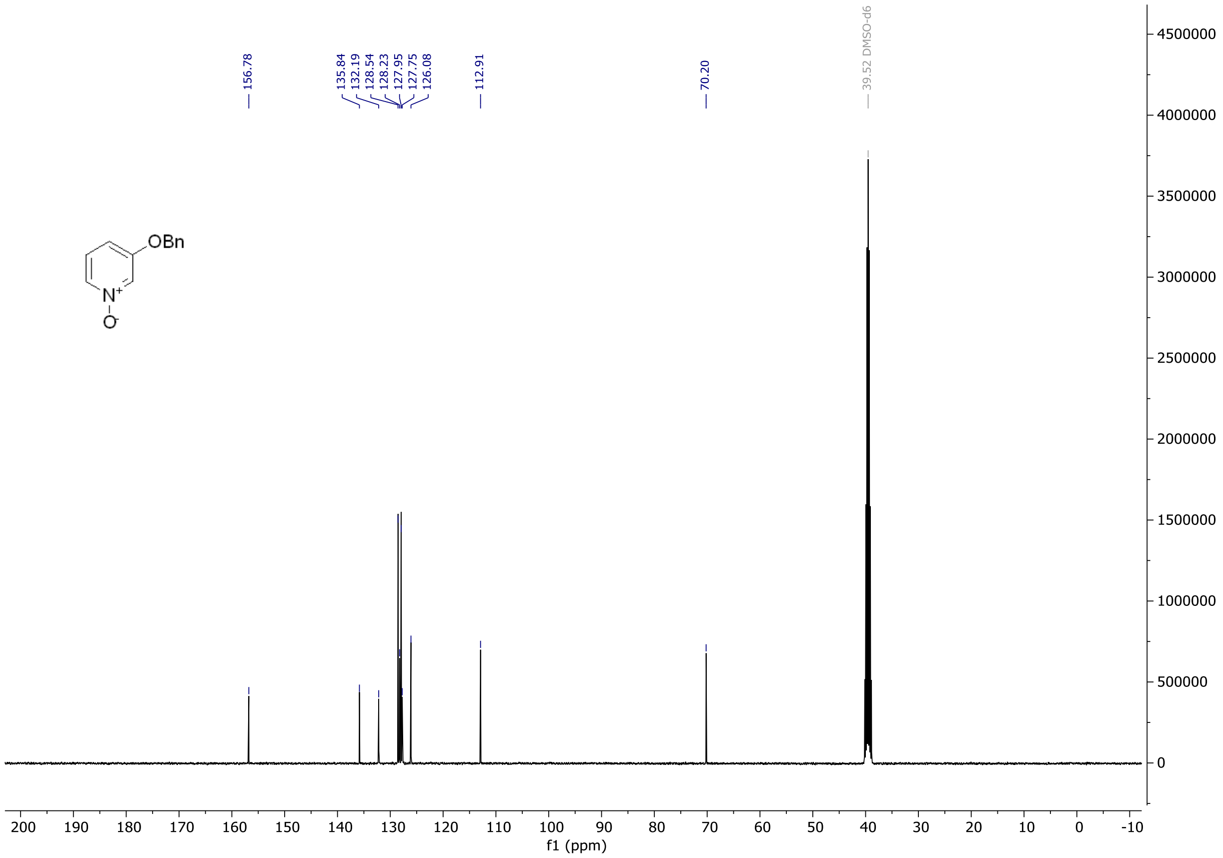

^{1}H NMR (400 MHz, $CDCl_3$) of **3a**:

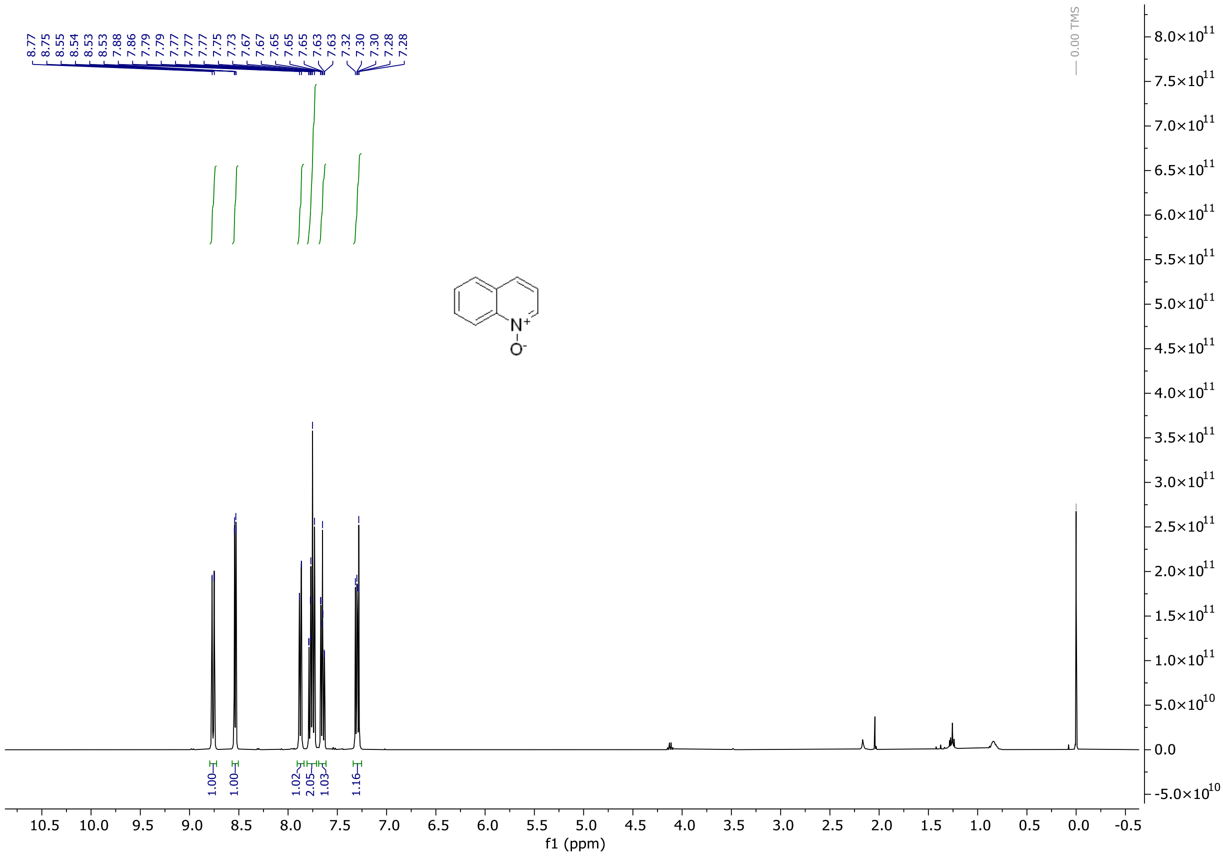

^{13}C NMR (101 MHz, $CDCl_3$) of **3a**:

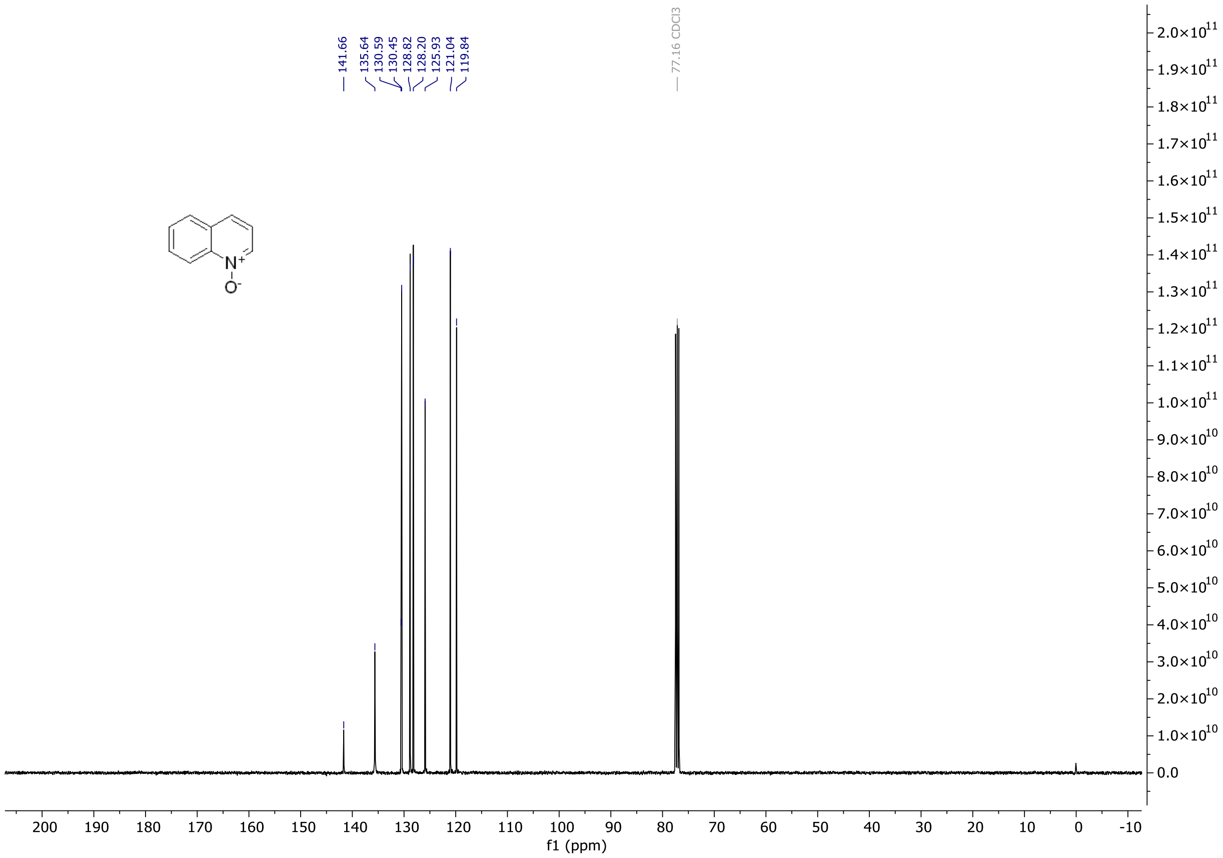

^{1}H NMR (400 MHz, $CDCl_3$) of **3b**:

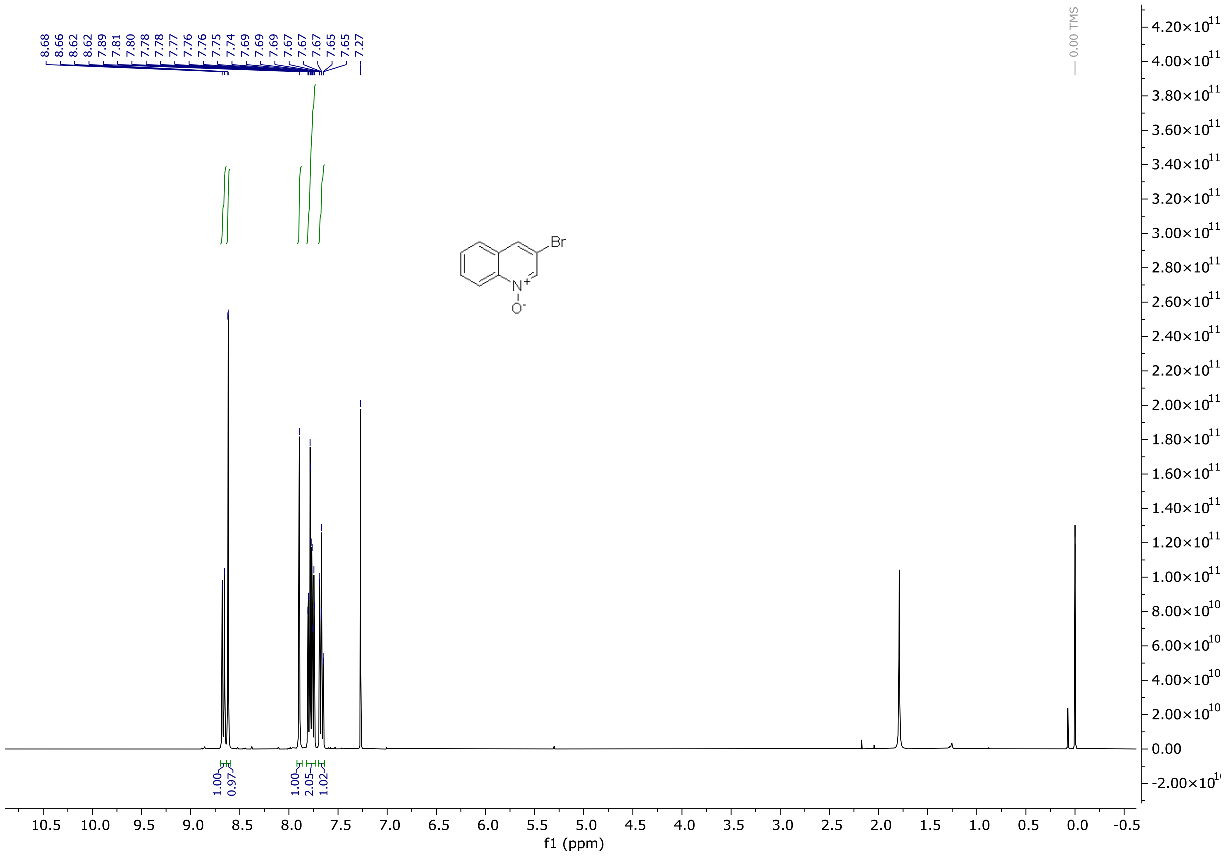

^{13}C NMR (101 MHz, $CDCl_3$) of **3b**:

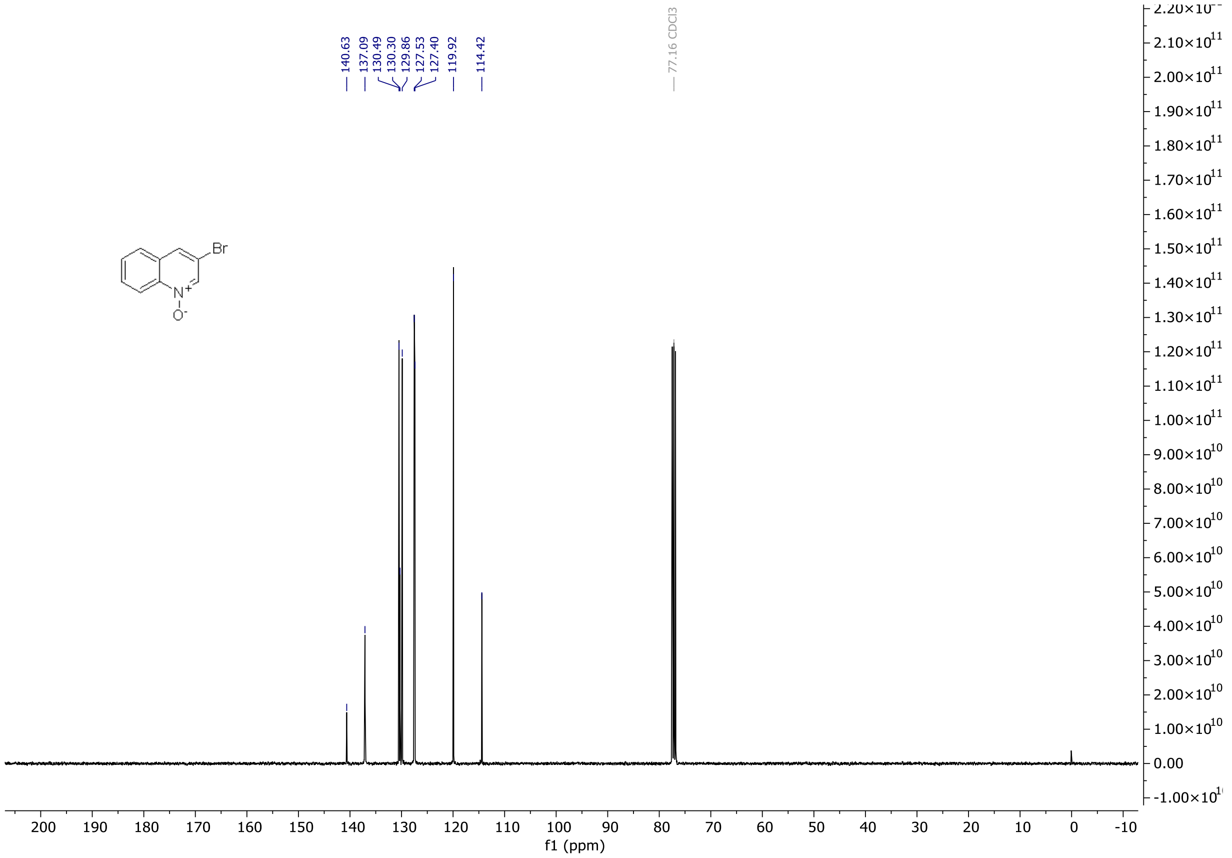

^{1}H NMR (400 MHz, $CDCl_3$) of **3c**:

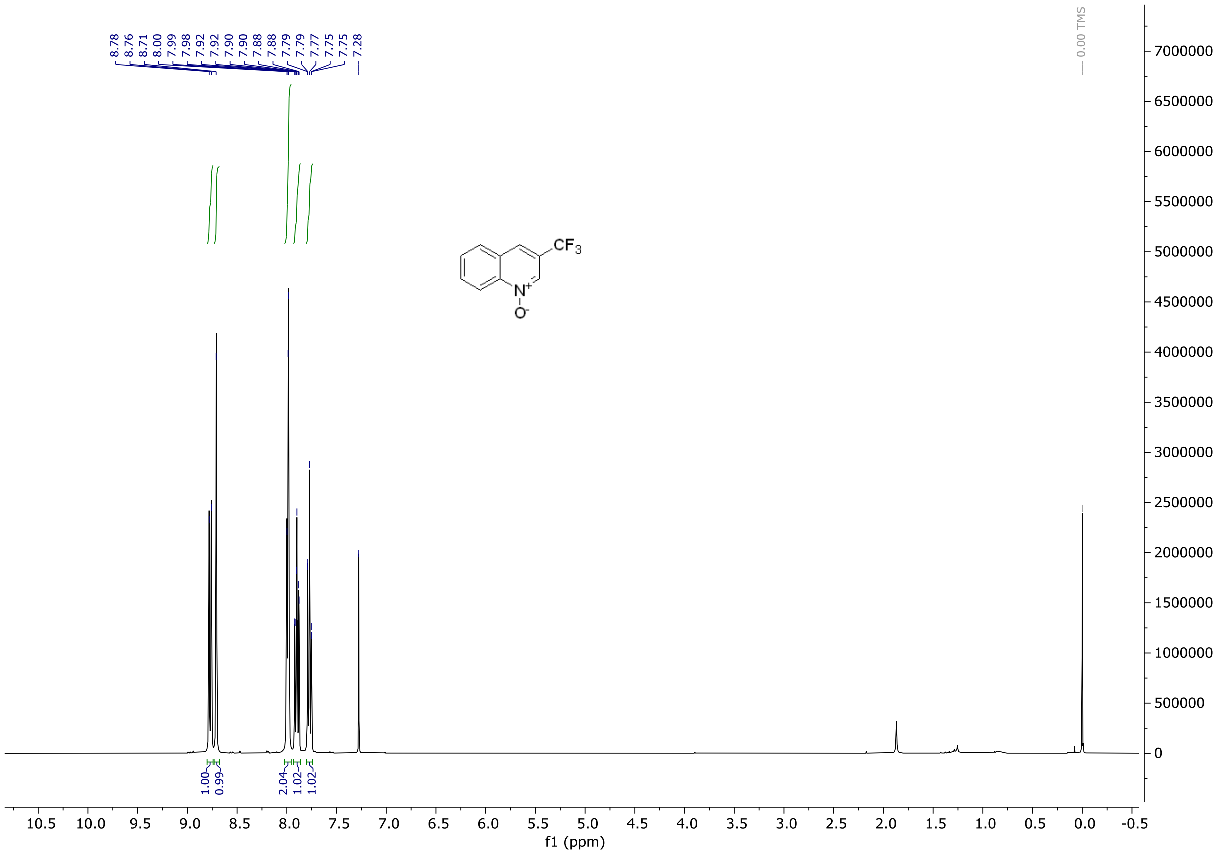

^{13}C NMR (101 MHz, $CDCl_3$) of **3c**:

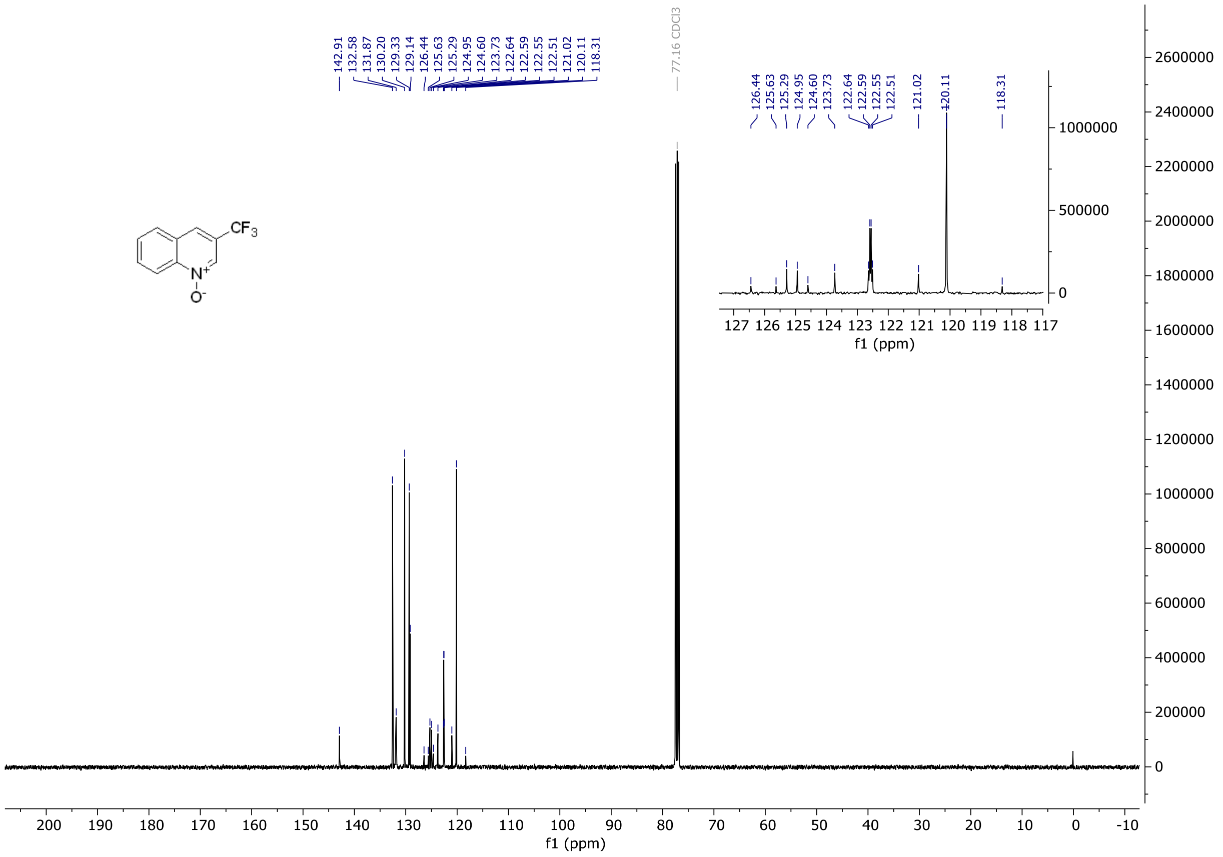

^{19}F NMR (376 MHz, $CDCl_3$) of **3c**:

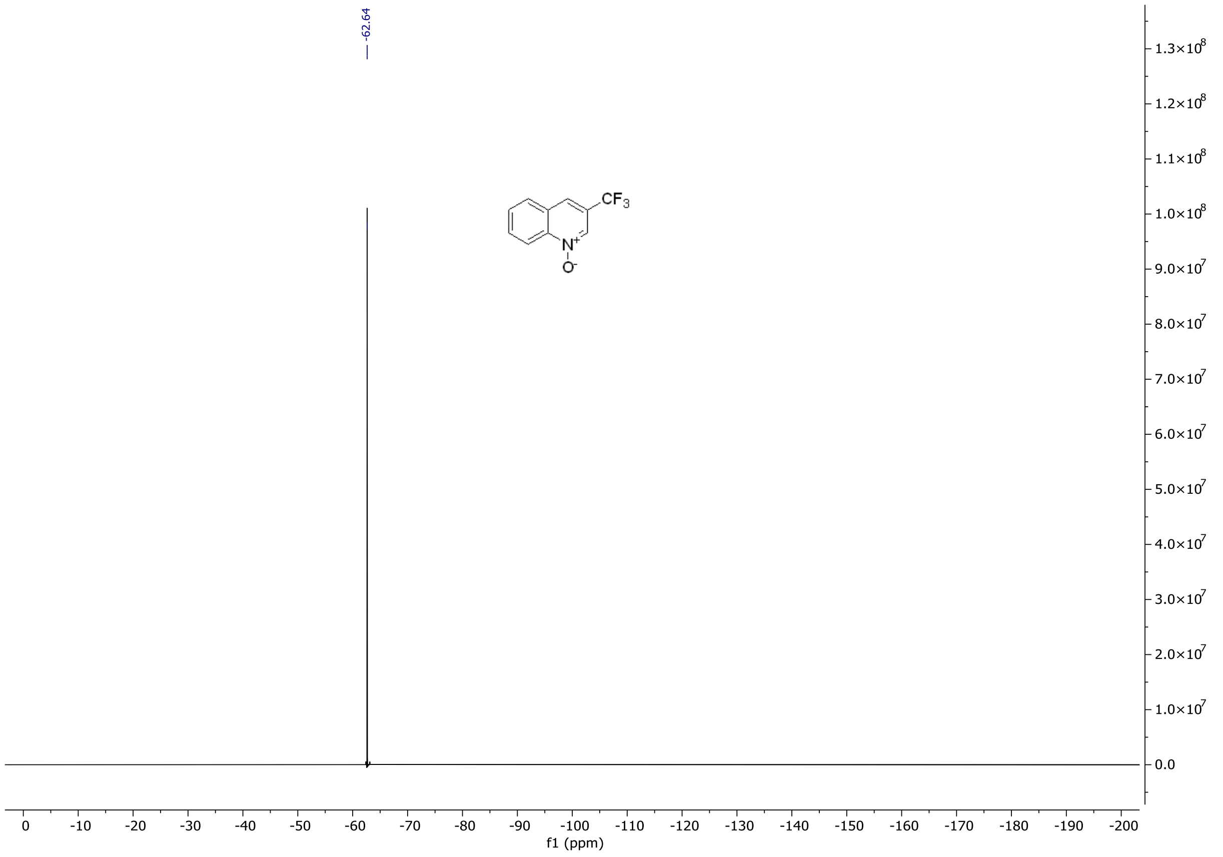

^{1}H NMR (400 MHz, $CDCl_3$) of **3d**:

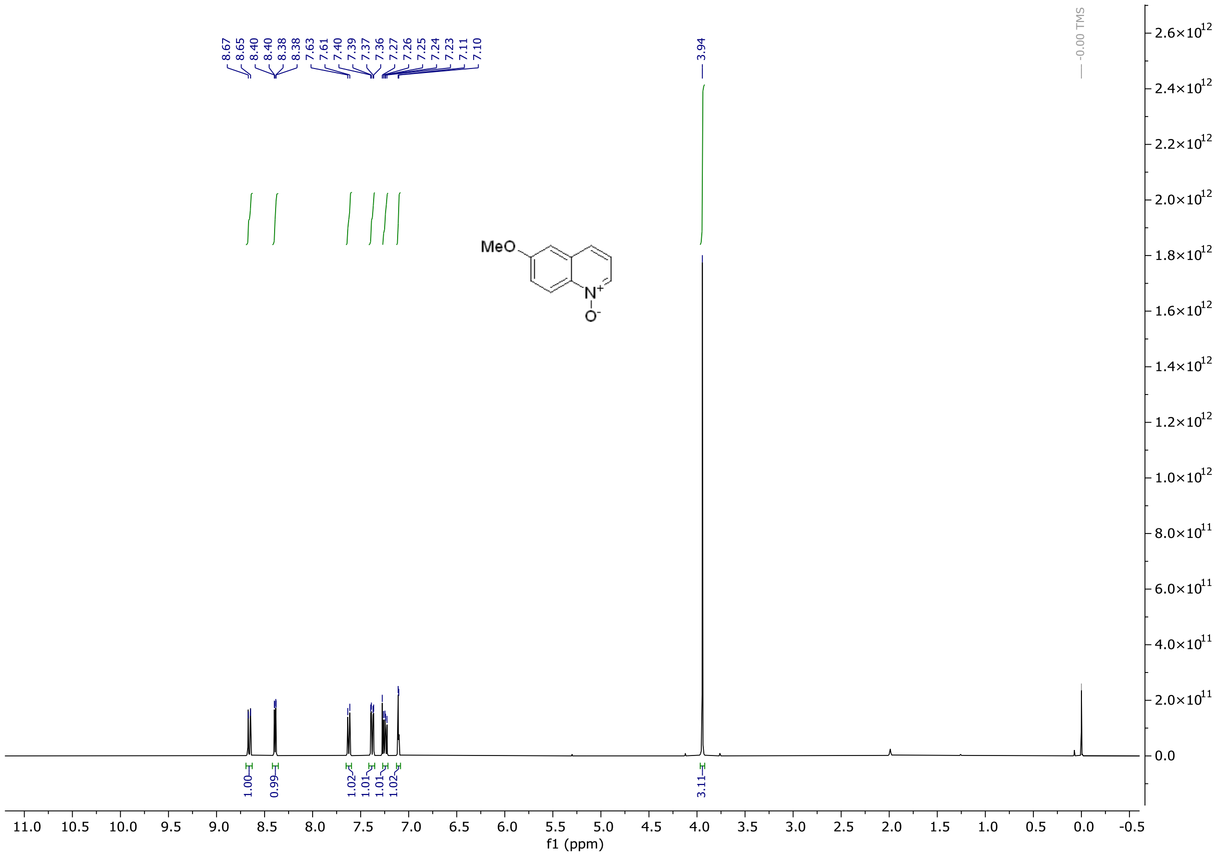

^{13}C NMR (101 MHz, $CDCl_3$) of **3d**:

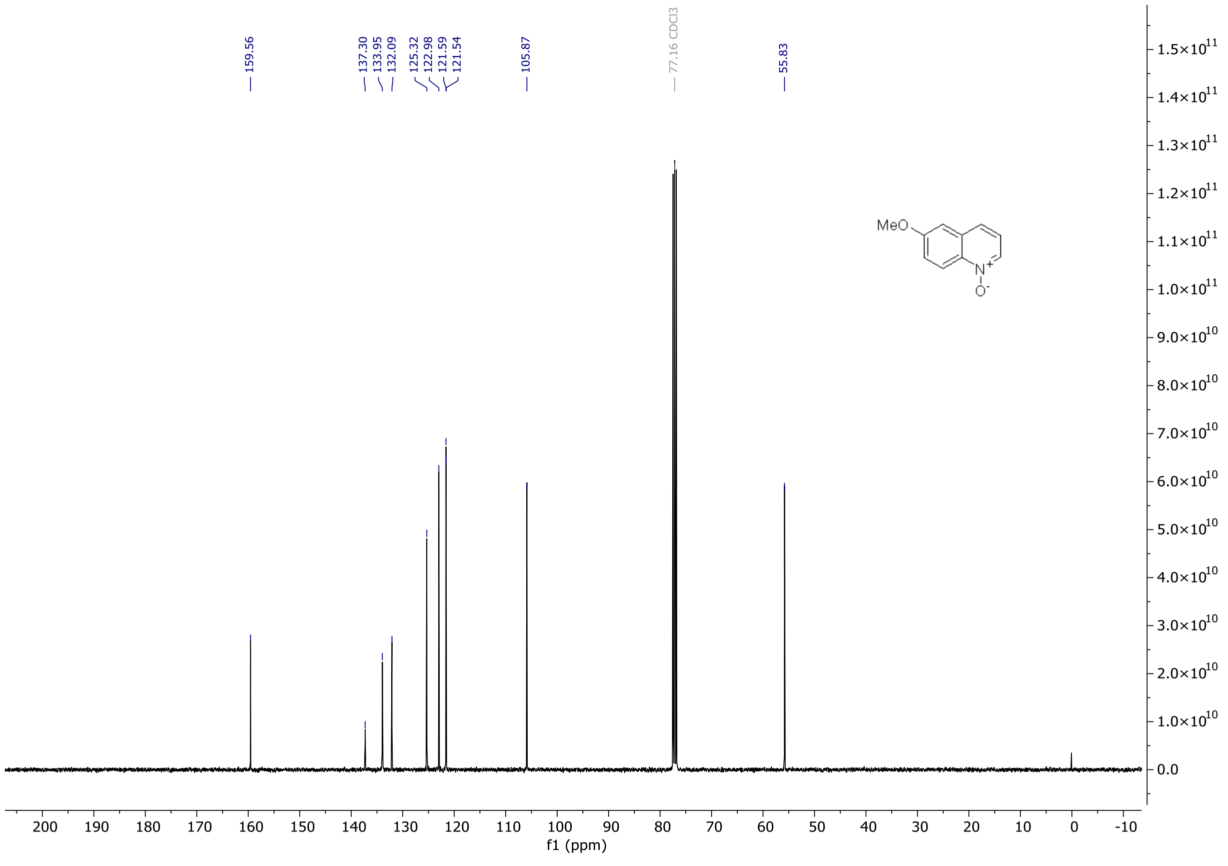

^{1}H NMR (400 MHz, $CDCl_3$) of **3e**:

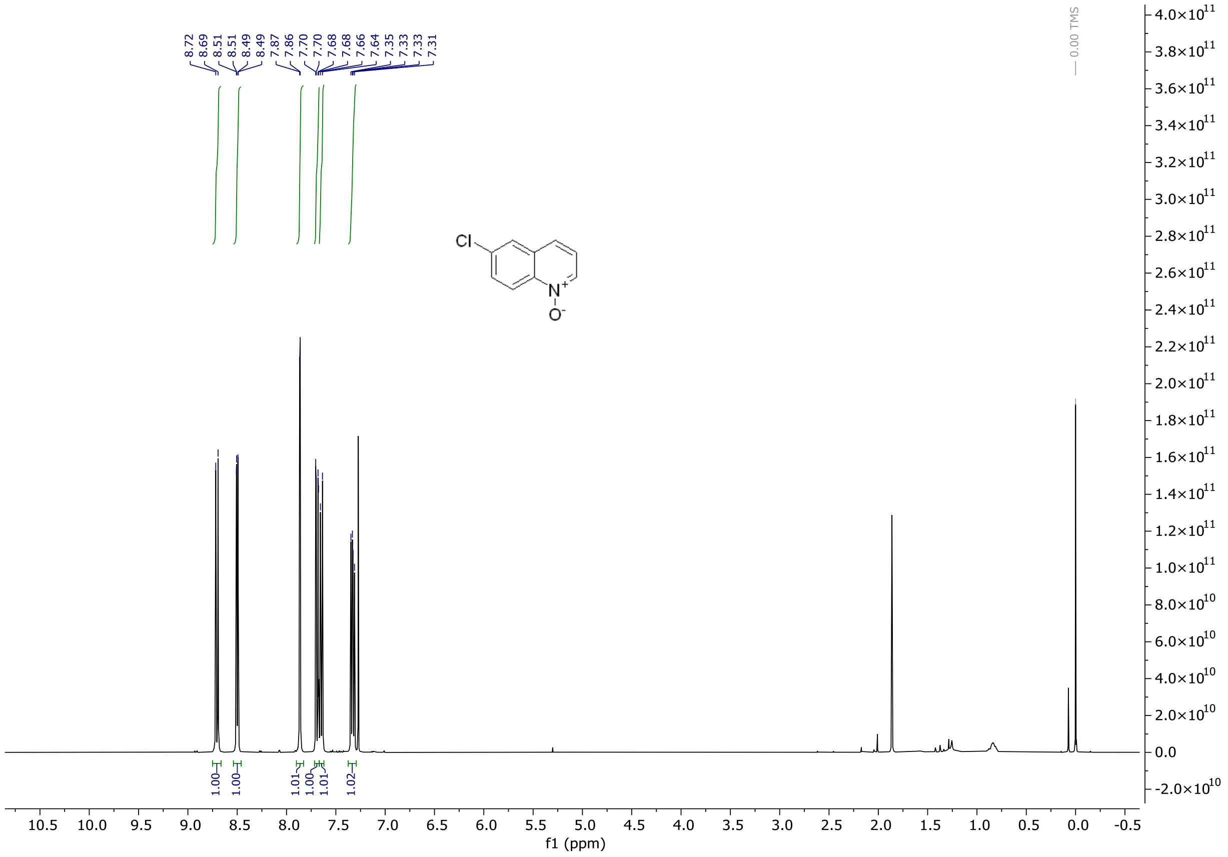

^{13}C NMR (101 MHz, $CDCl_3$) of **3e**:

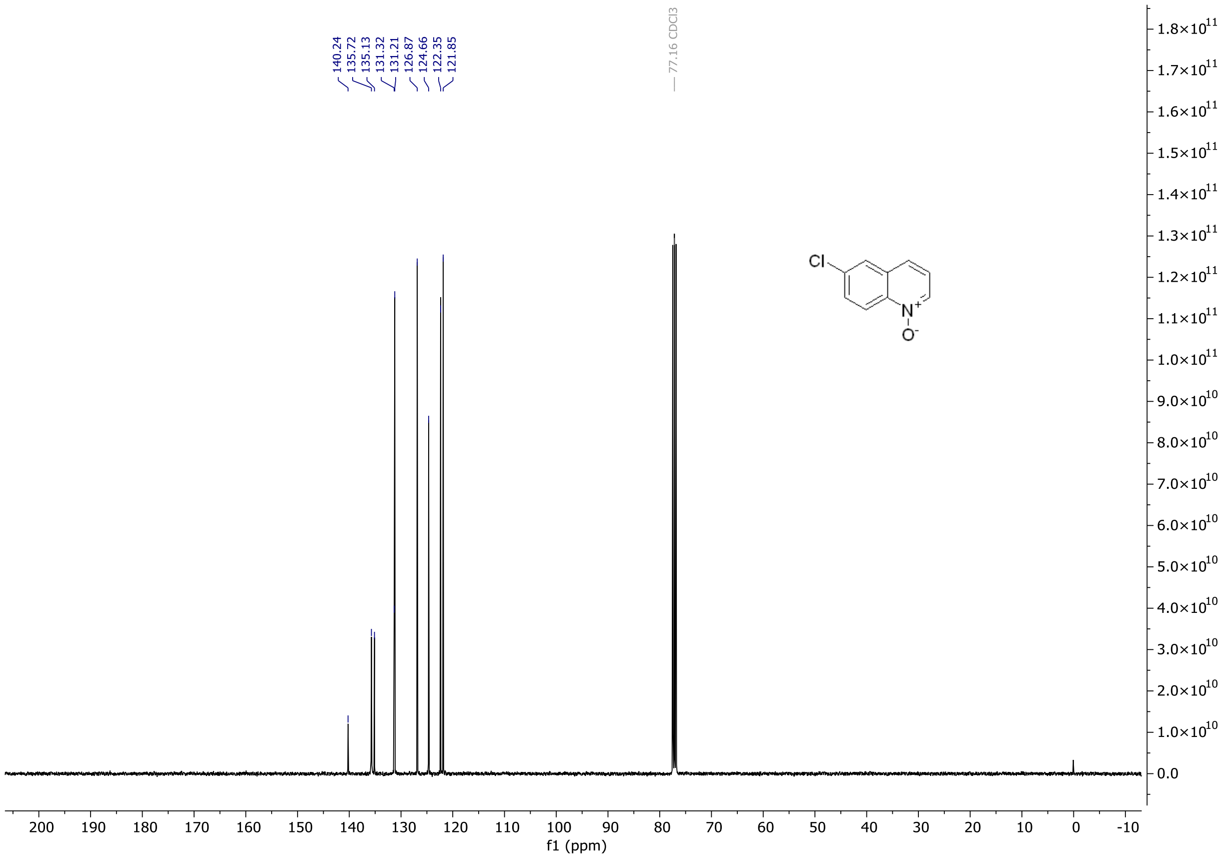

^{1}H NMR (400 MHz, $CDCl_3$) of **3f**:

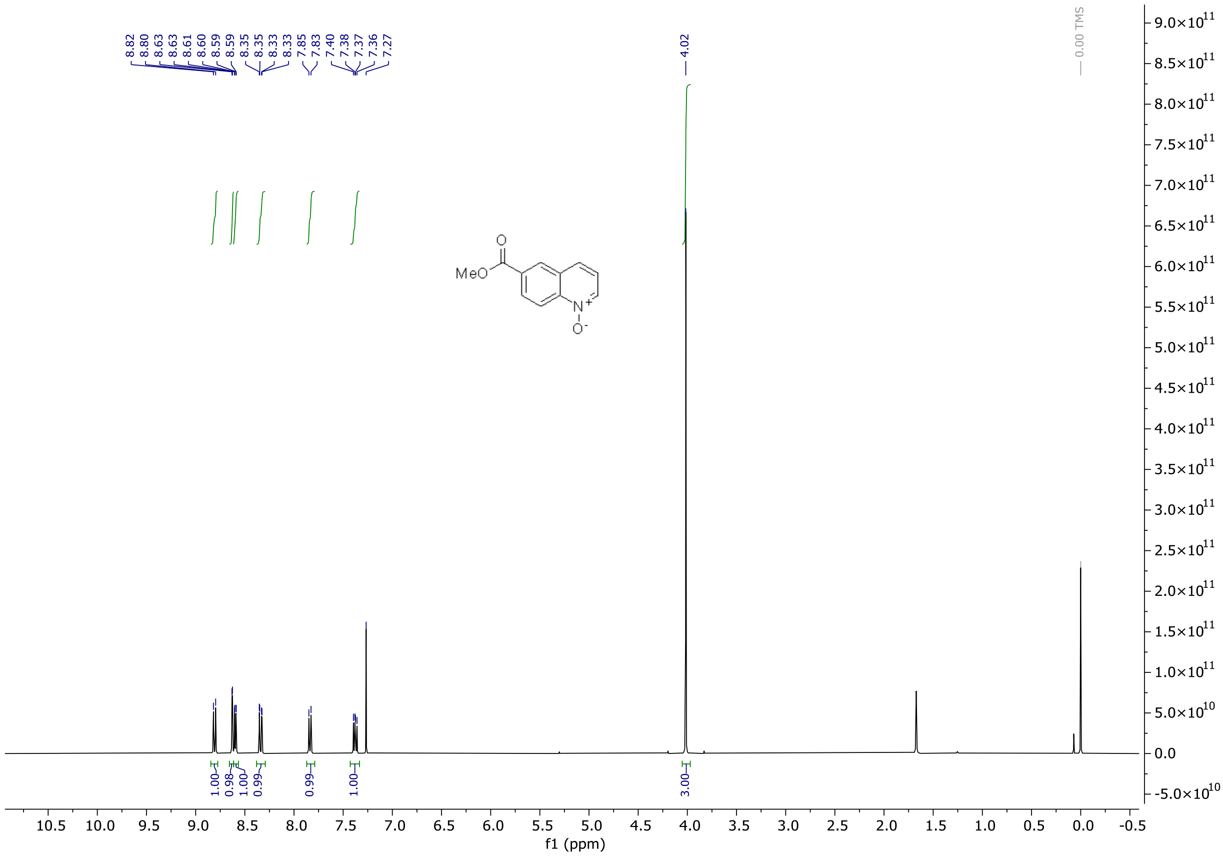

^{13}C NMR (101 MHz, $CDCl_3$) of **3f**:

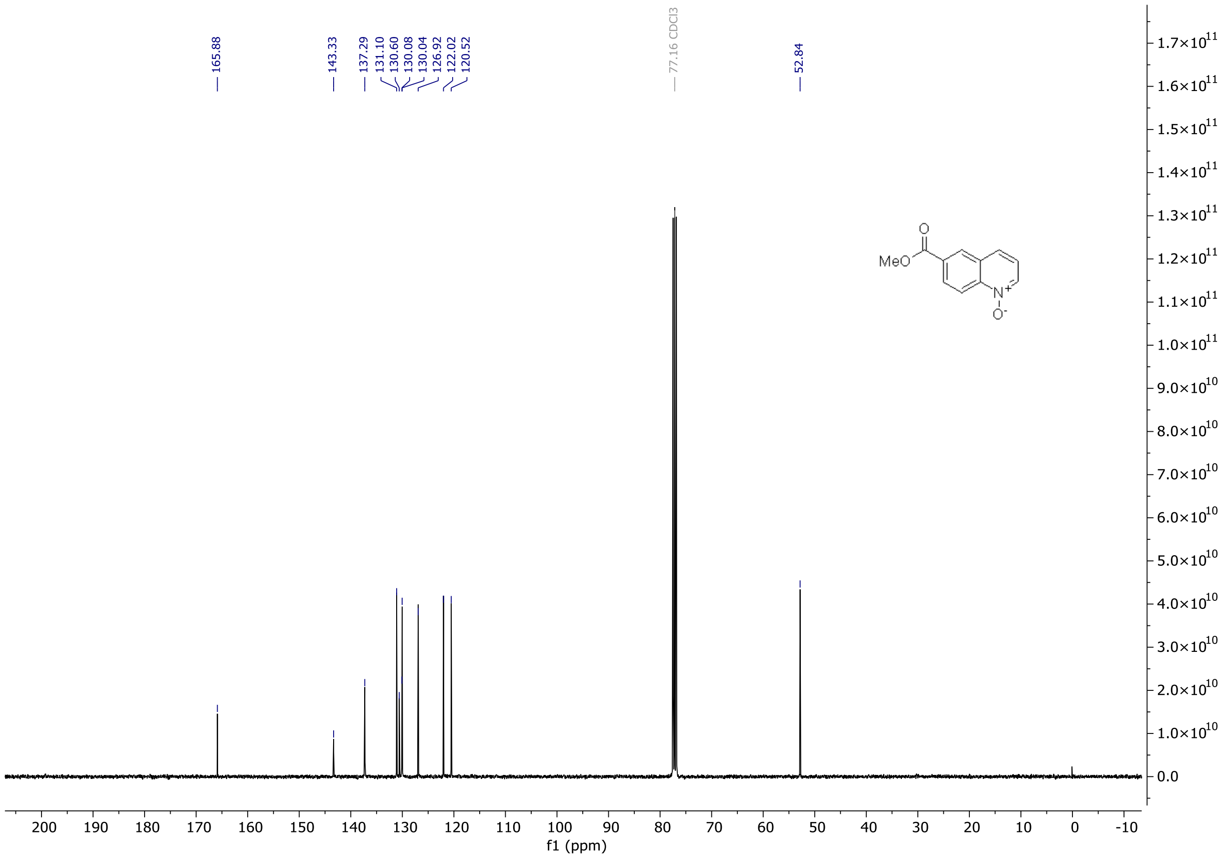

^{1}H NMR (400 MHz, DMSO-d_6) of **3g**:

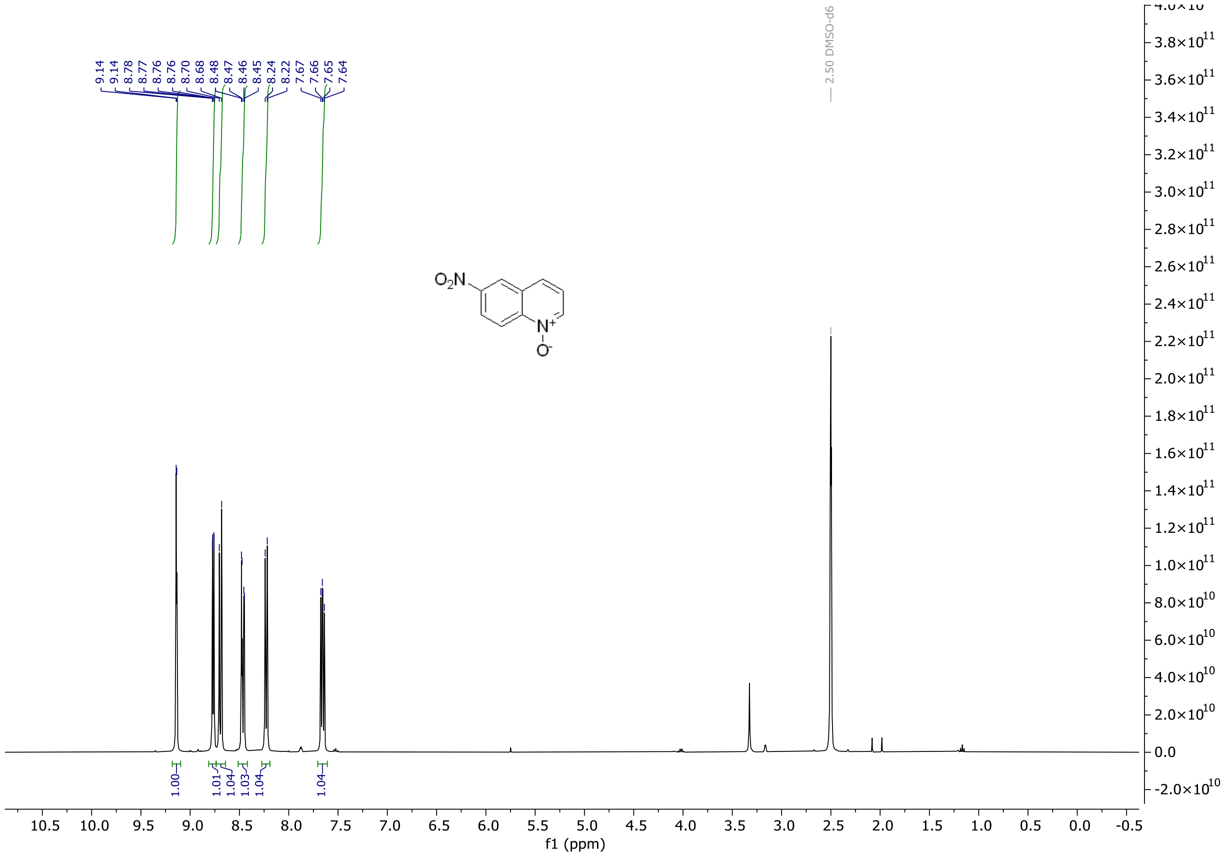

^{13}C NMR (101 MHz, DMSO-d_6) of **3g**:

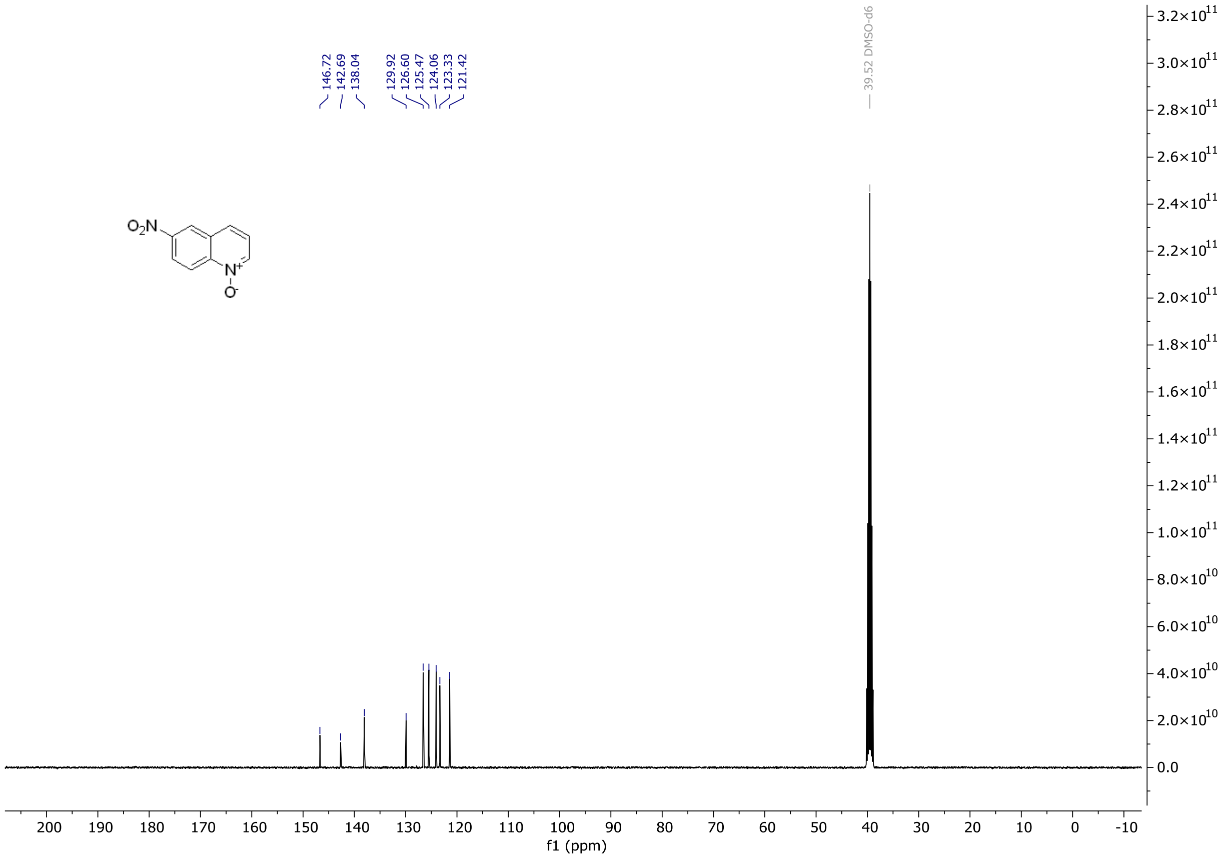

^{1}H NMR (400 MHz, $CDCl_3$) of **3h**:

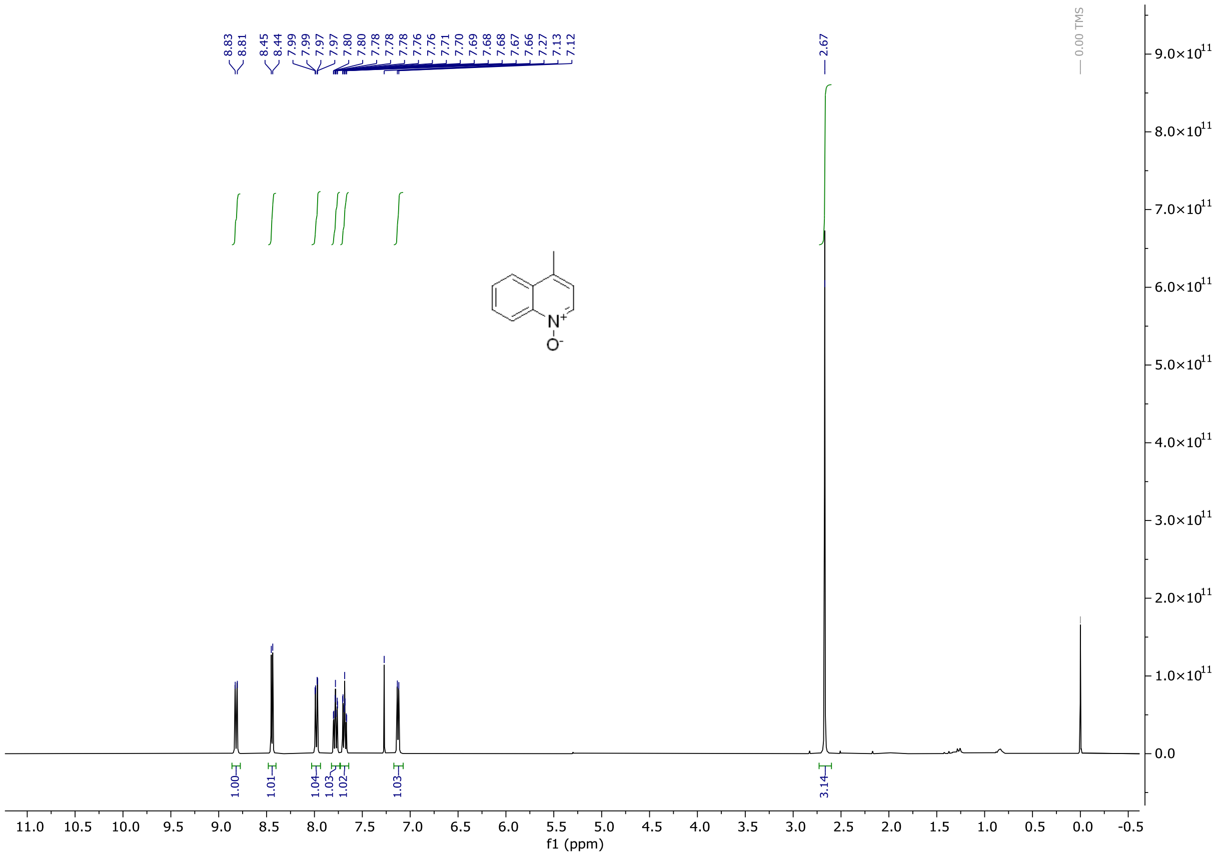

^{13}C NMR (101 MHz, $CDCl_3$) of **3h**:

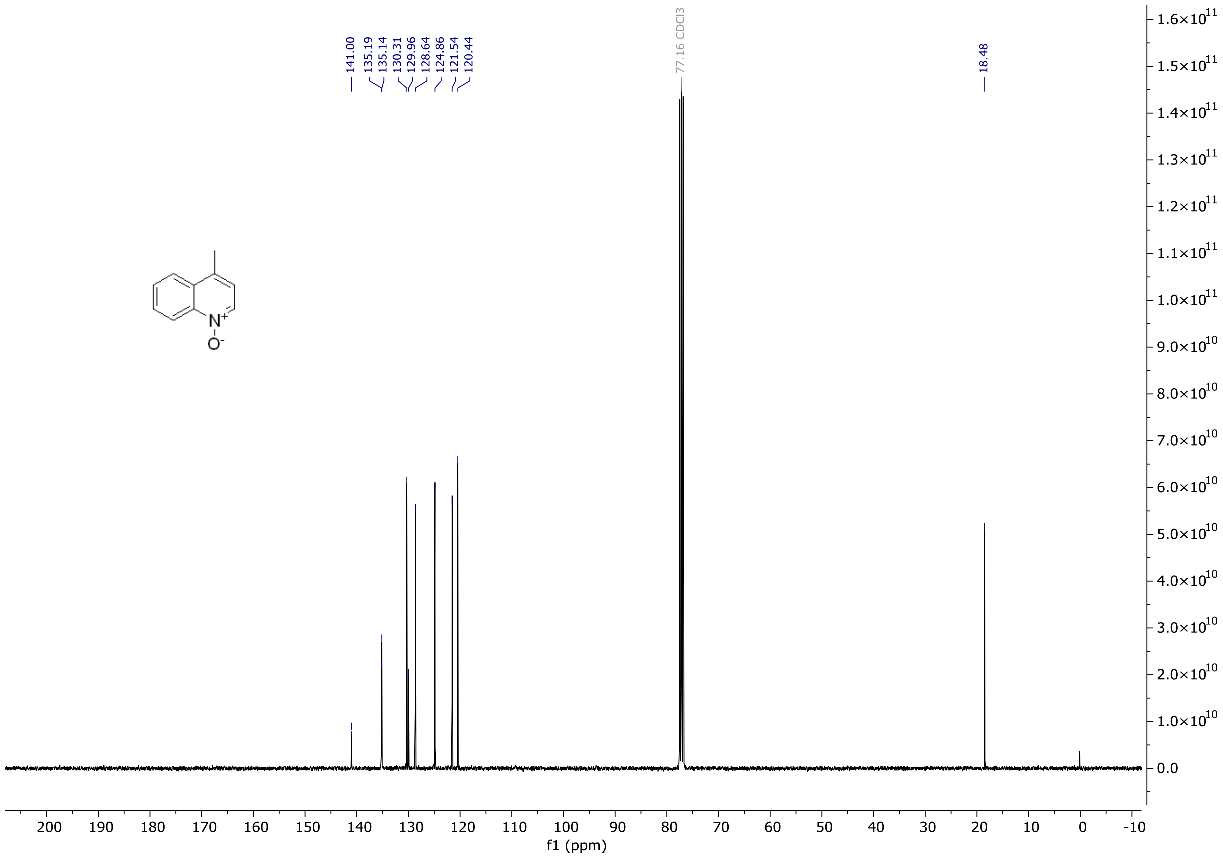

^{1}H NMR (400 MHz, $CDCl_3$) of **3i**:

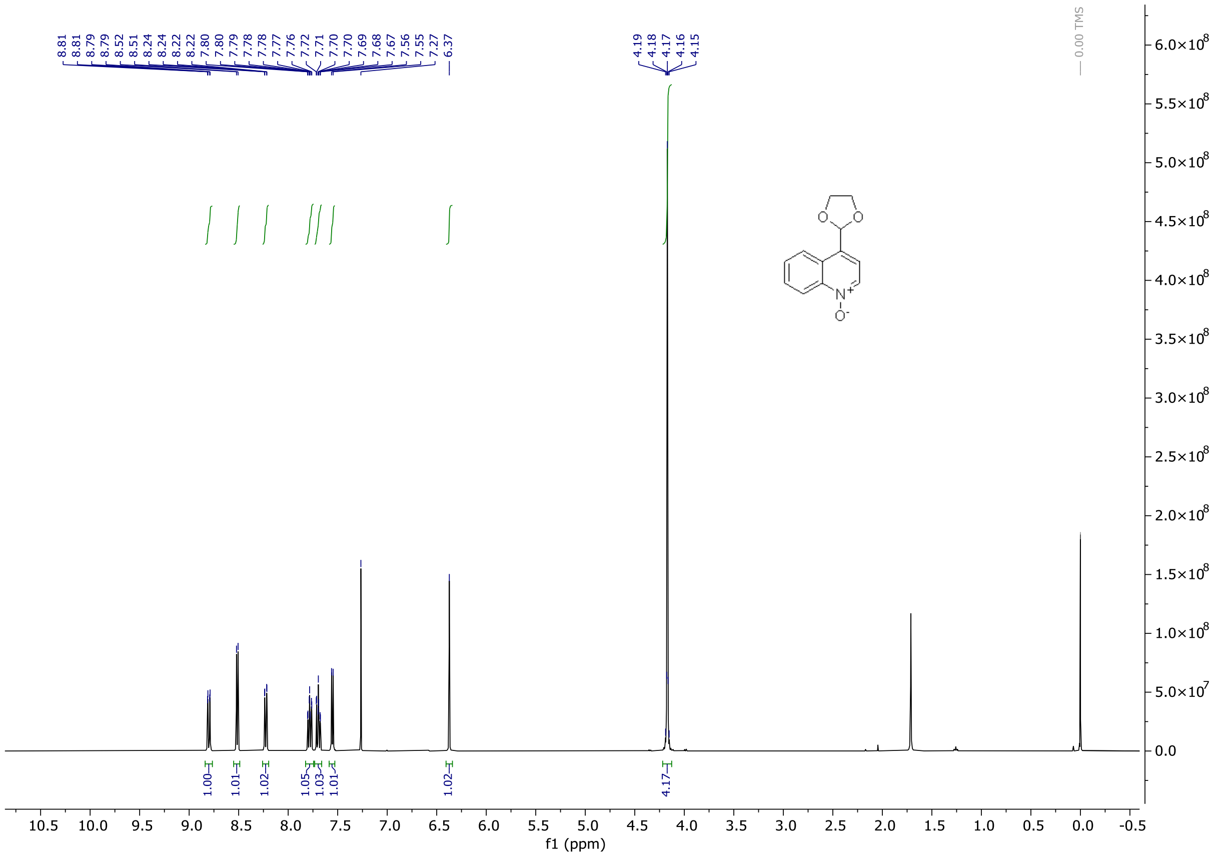

^{13}C NMR (101 MHz, $CDCl_3$) of **3i**:

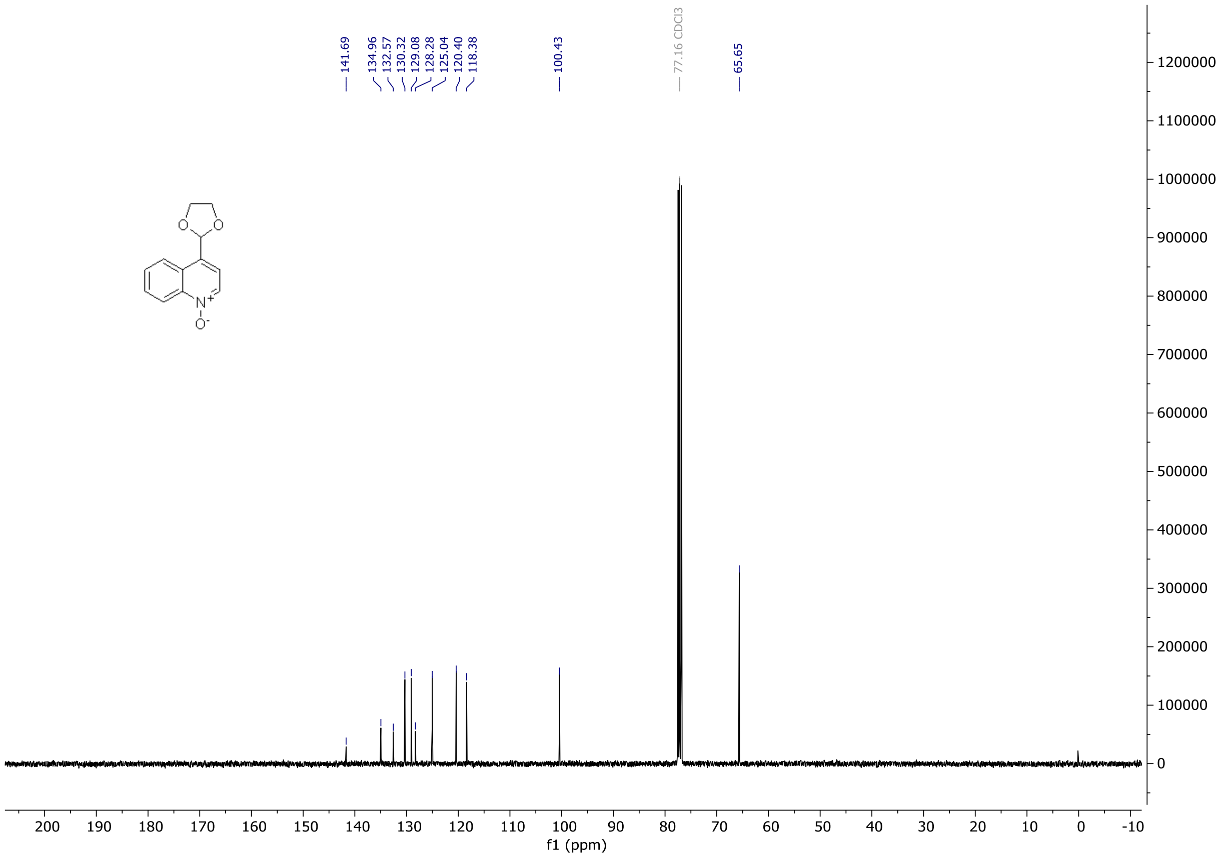

^{1}H NMR (400 MHz, $CDCl_3$) of **3j**:

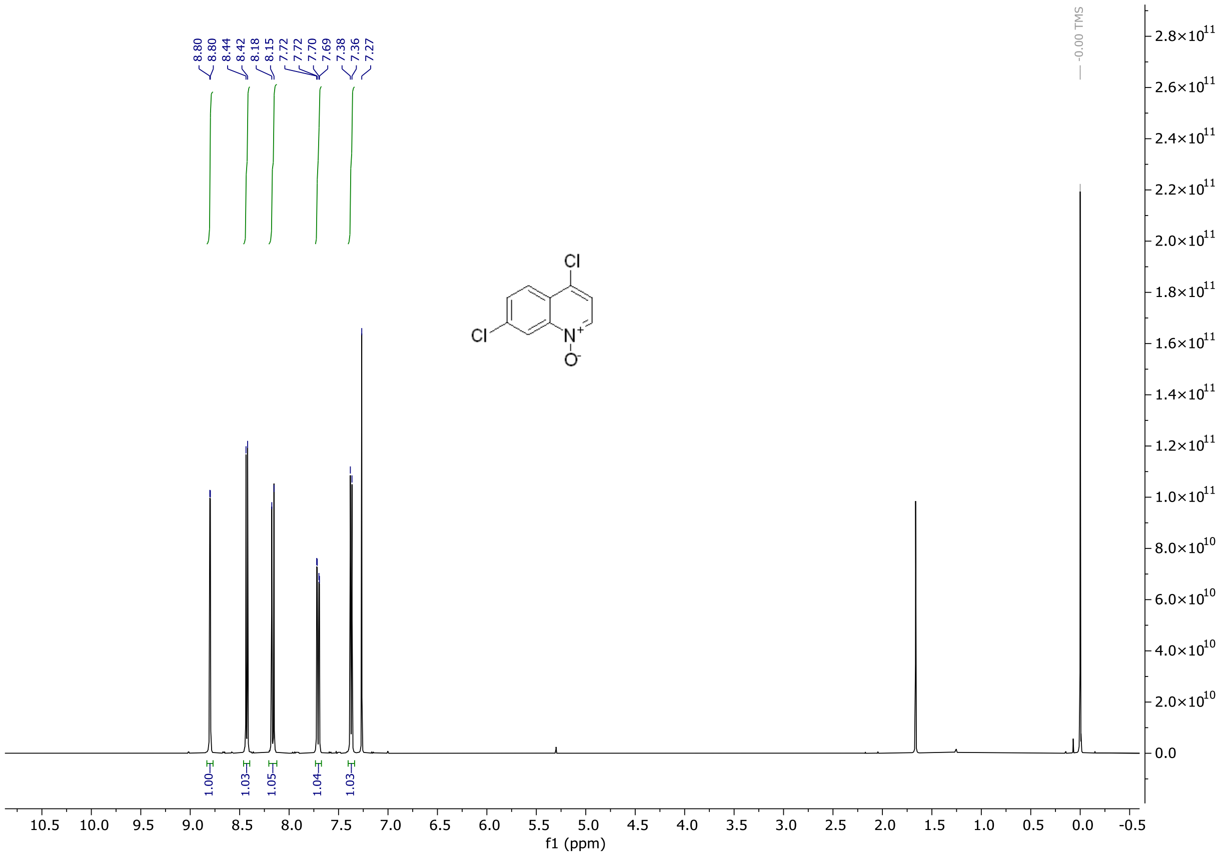

^{13}C NMR (101 MHz, $CDCl_3$) of **3j**:

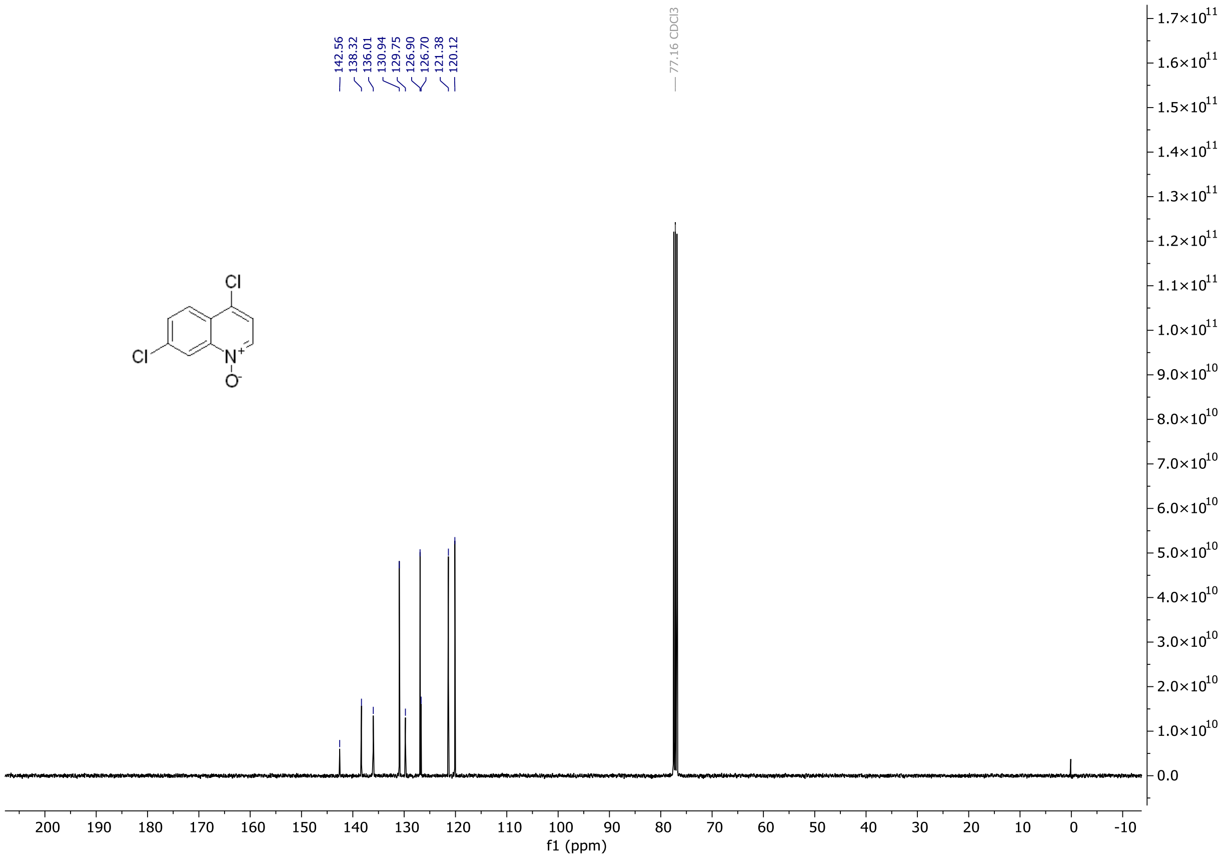

^{1}H NMR (400 MHz, $CDCl_3$) of **3k**:

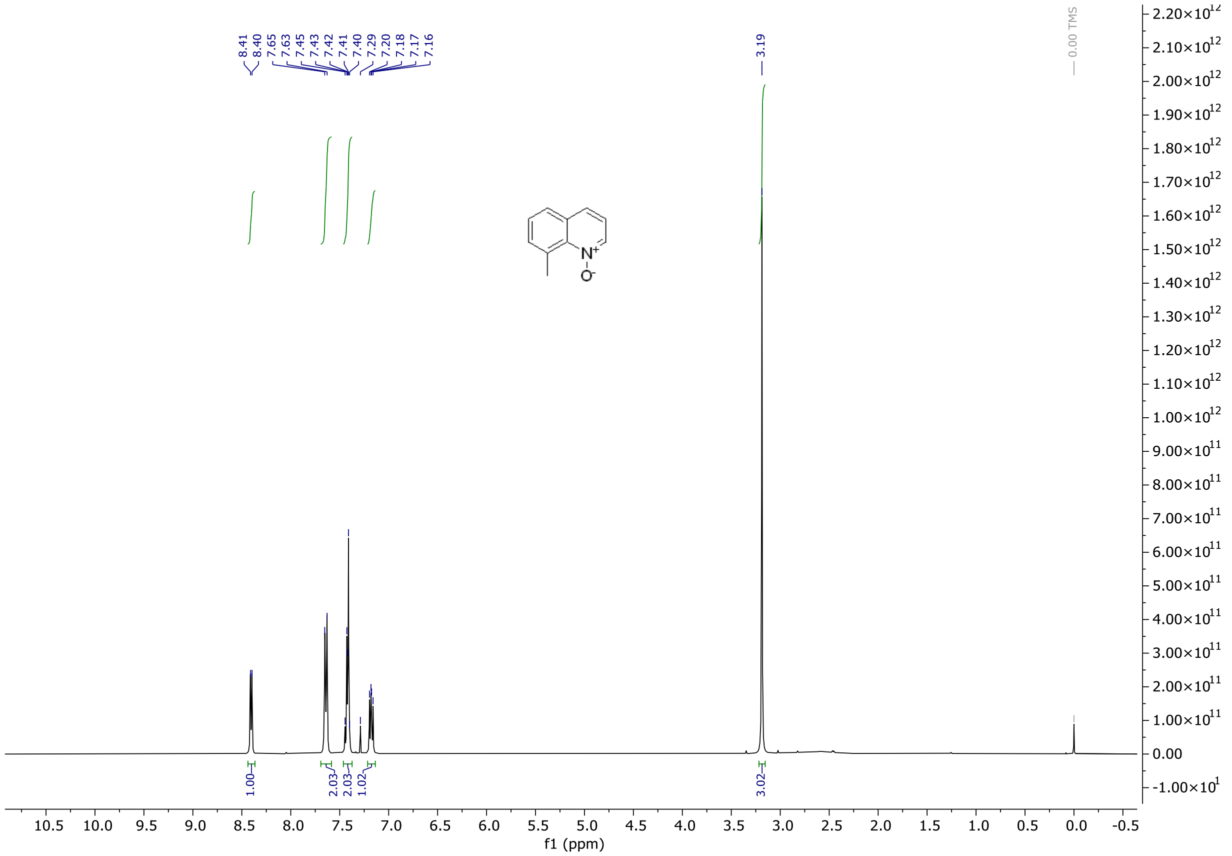

^{13}C NMR (101 MHz, $CDCl_3$) of **3k**:

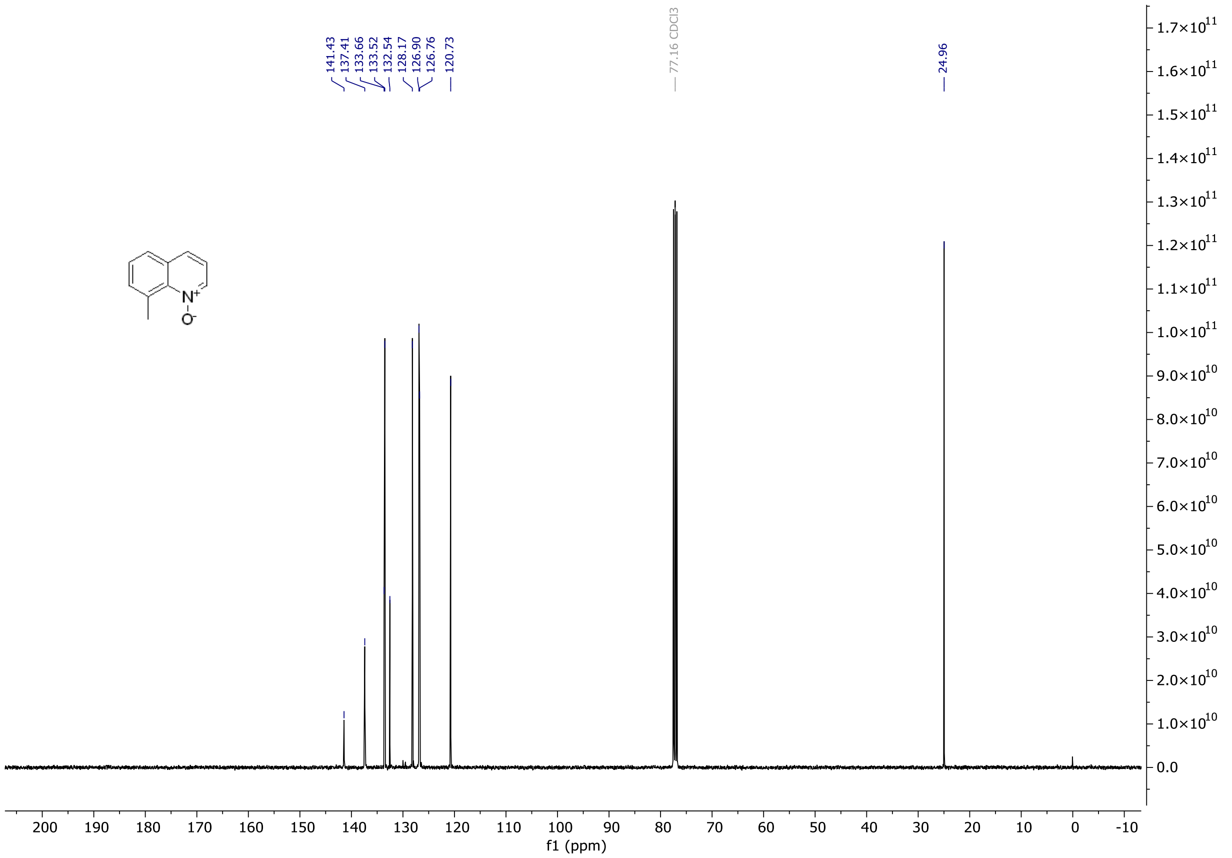

^1H NMR (400 MHz, $CDCl_3$) of **3l**:

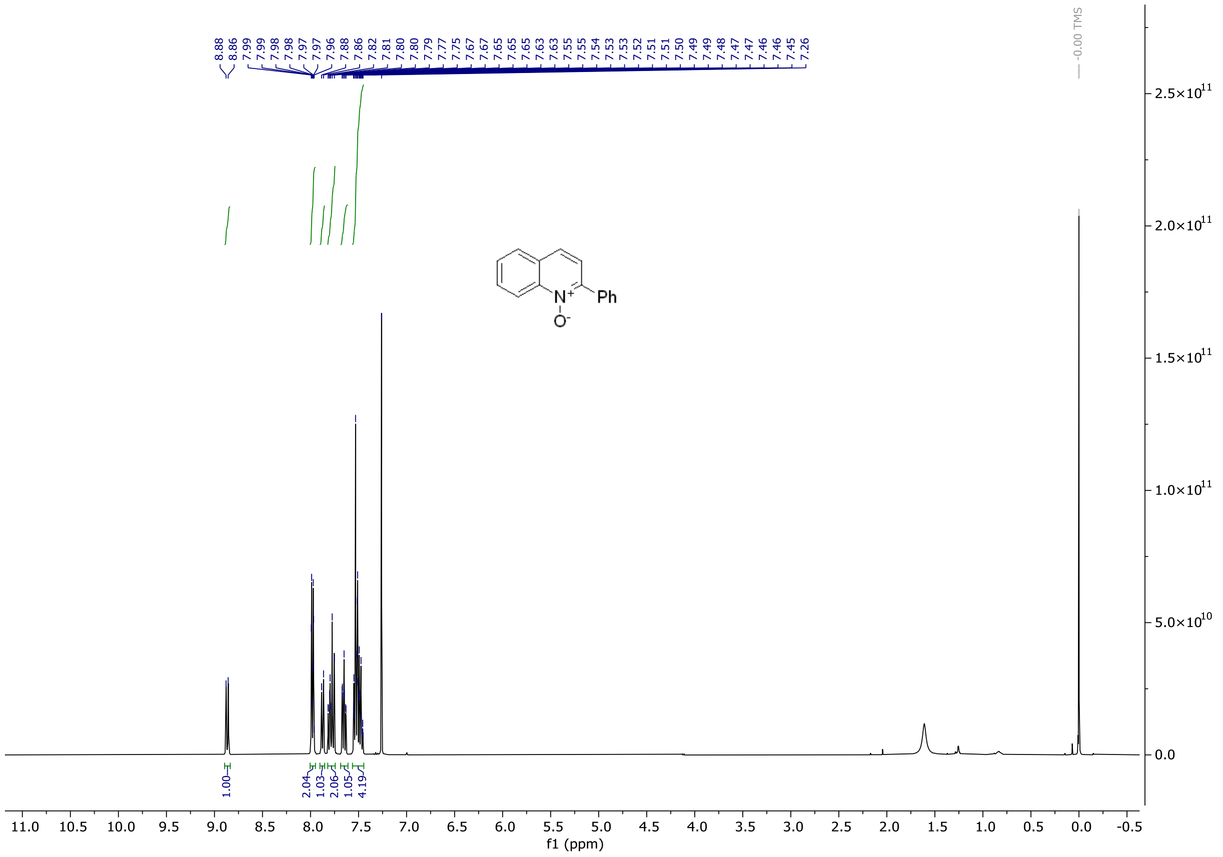

^{13}C NMR (101 MHz, $CDCl_3$) of **3l**:

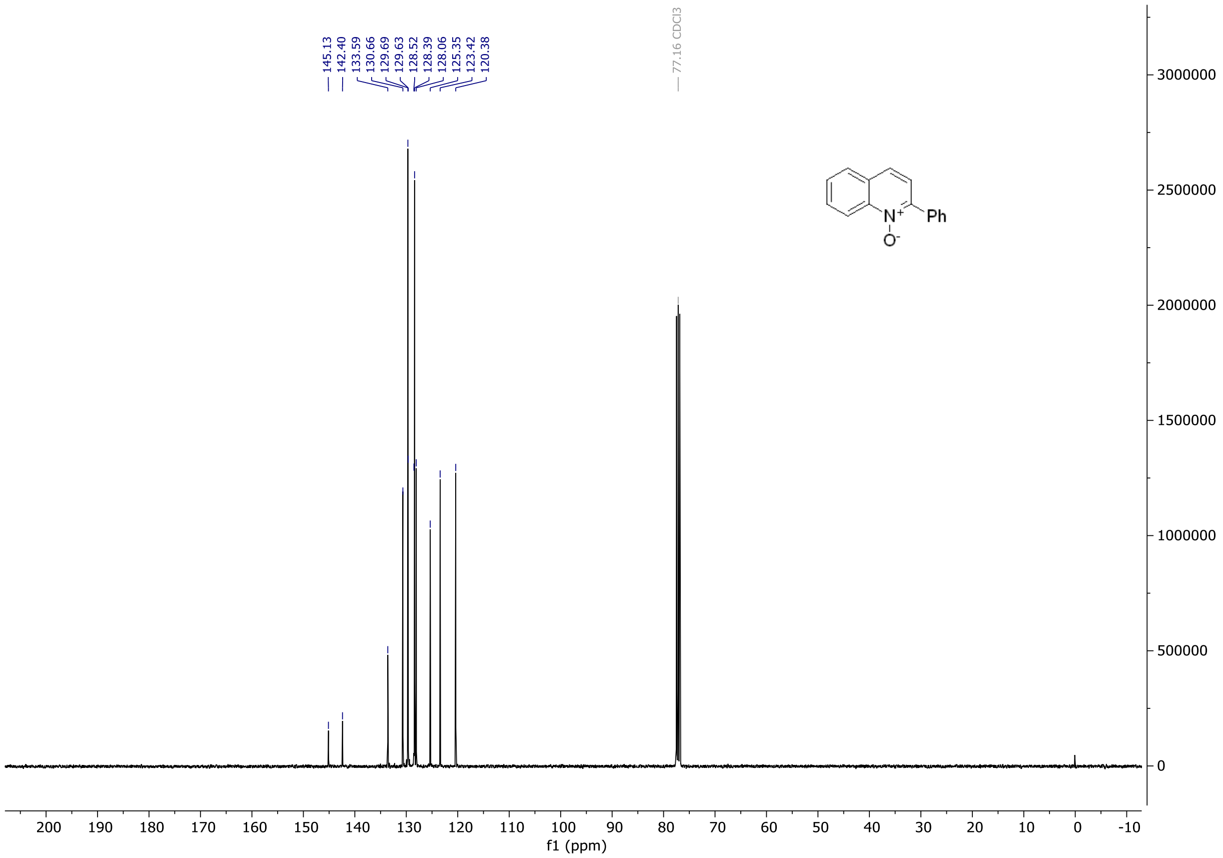

^{1}H NMR (400 MHz, $CDCl_3$) of **3m**:

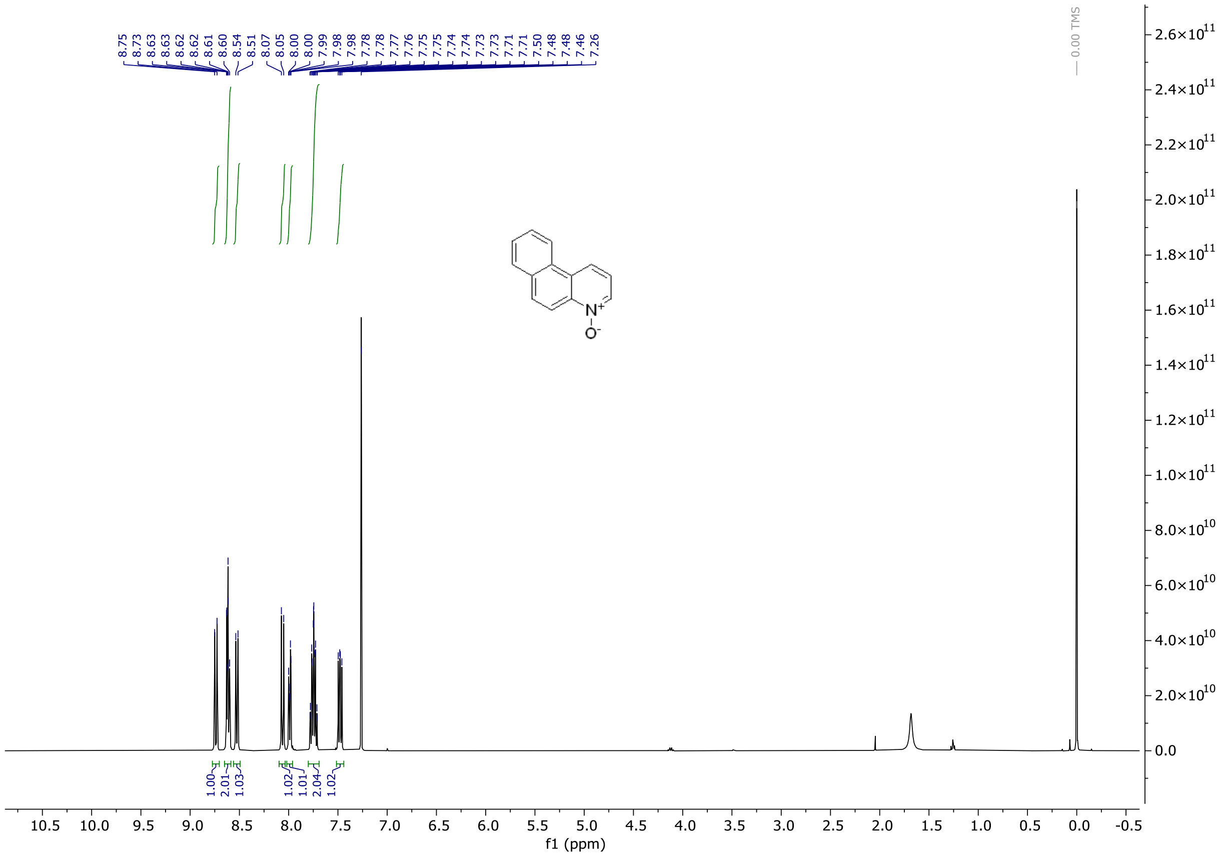

^{13}C NMR (101 MHz, $CDCl_3$) of **3m**:

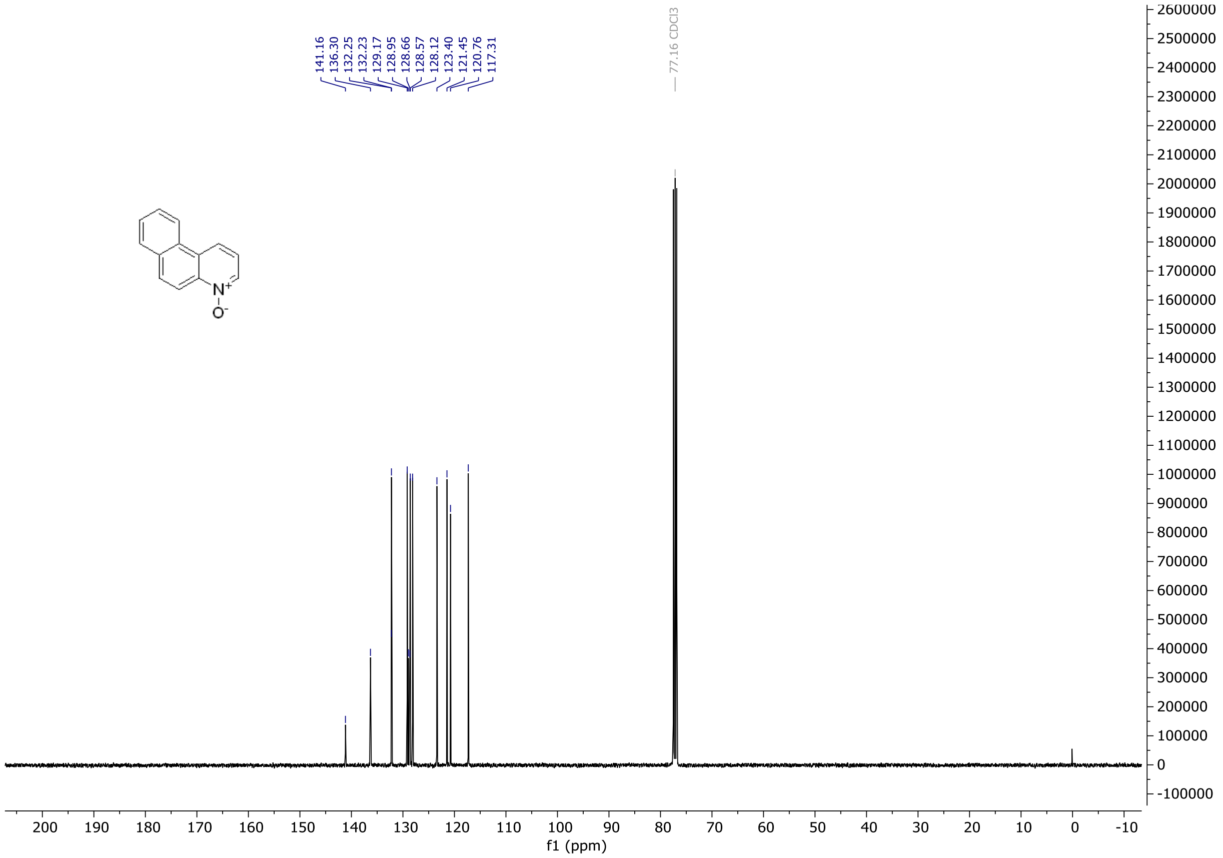

^{1}H NMR (400 MHz, $CDCl_3$) of **3n**:

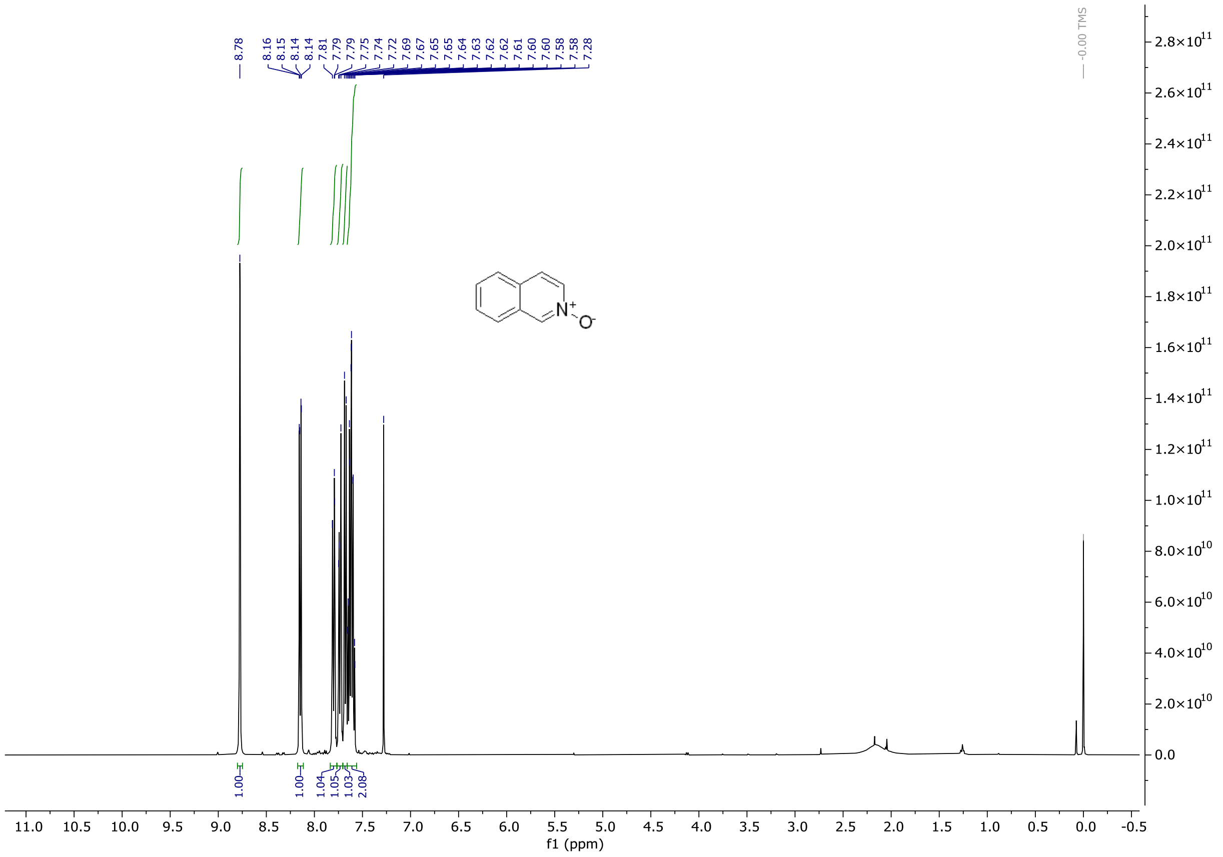

^{13}C NMR (101 MHz, $CDCl_3$) of **3n**:

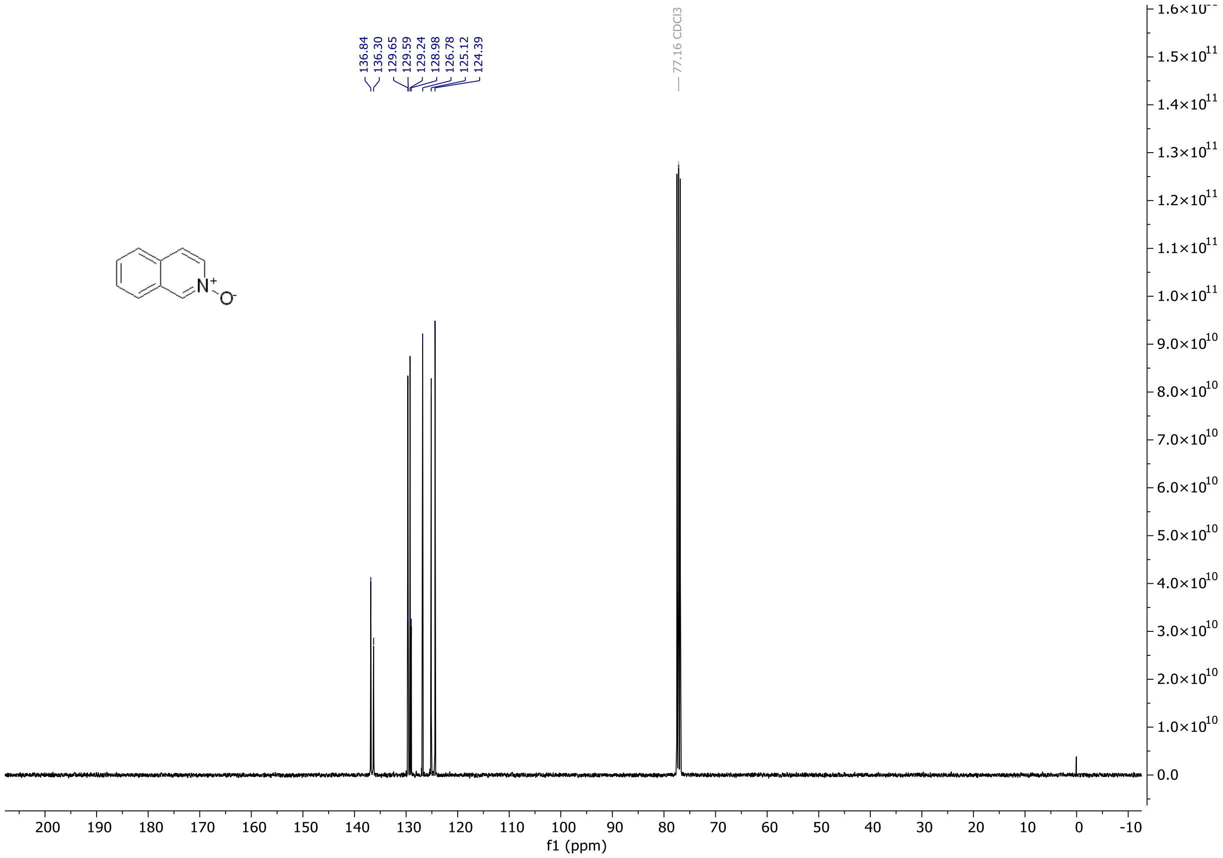

^{1}H NMR (400 MHz, $CDCl_3$) of **3o**:

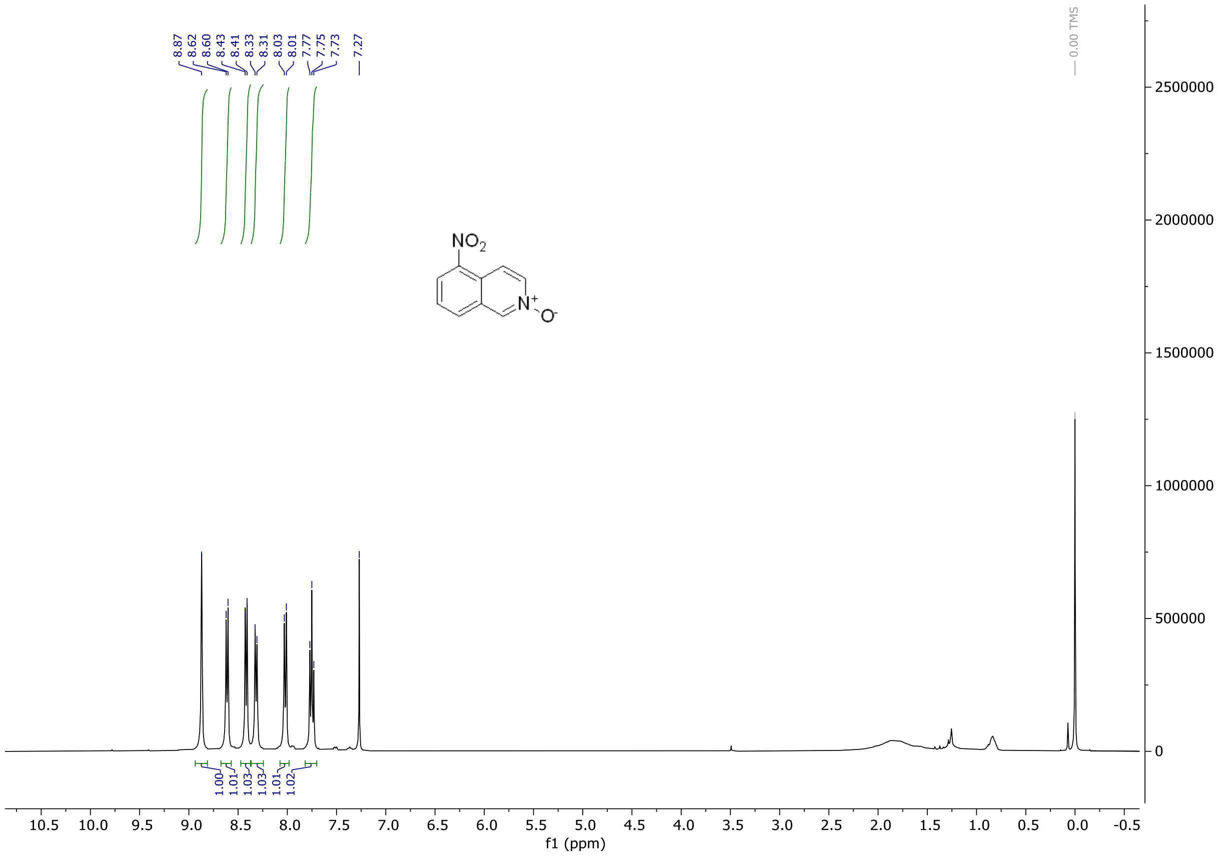

^{13}C NMR (101 MHz, $CDCl_3$) of **3o**:

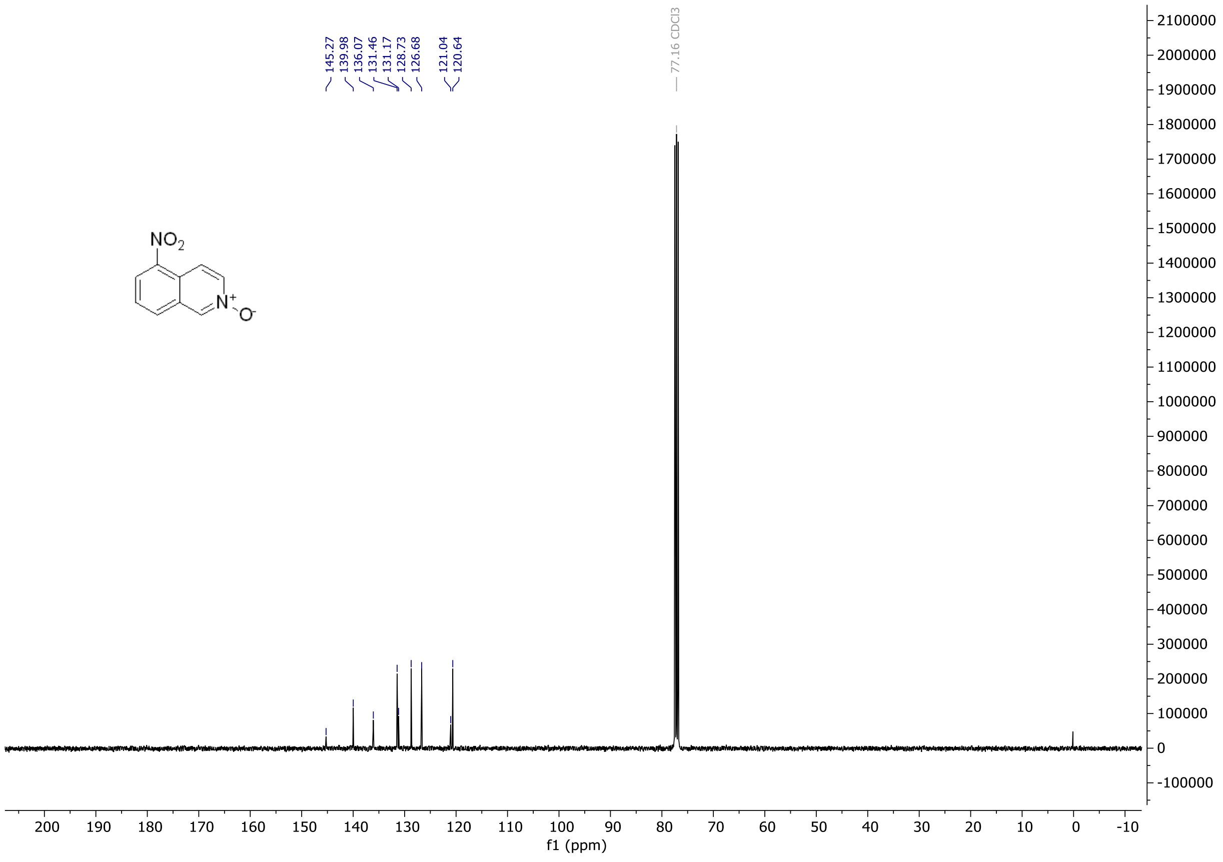

^{1}H NMR (400 MHz, $CDCl_3$) of **3q**:

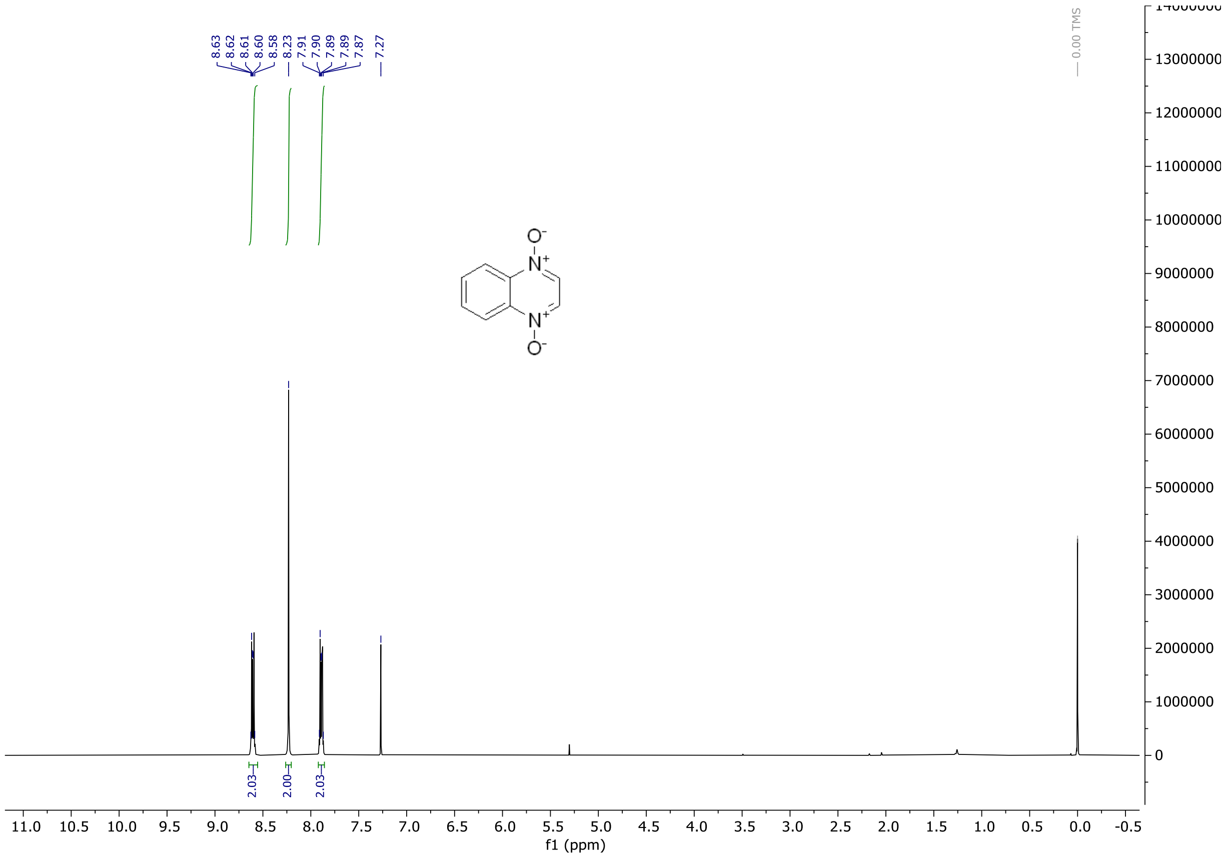

^{13}C NMR (101 MHz, $CDCl_3$) of **3q**:

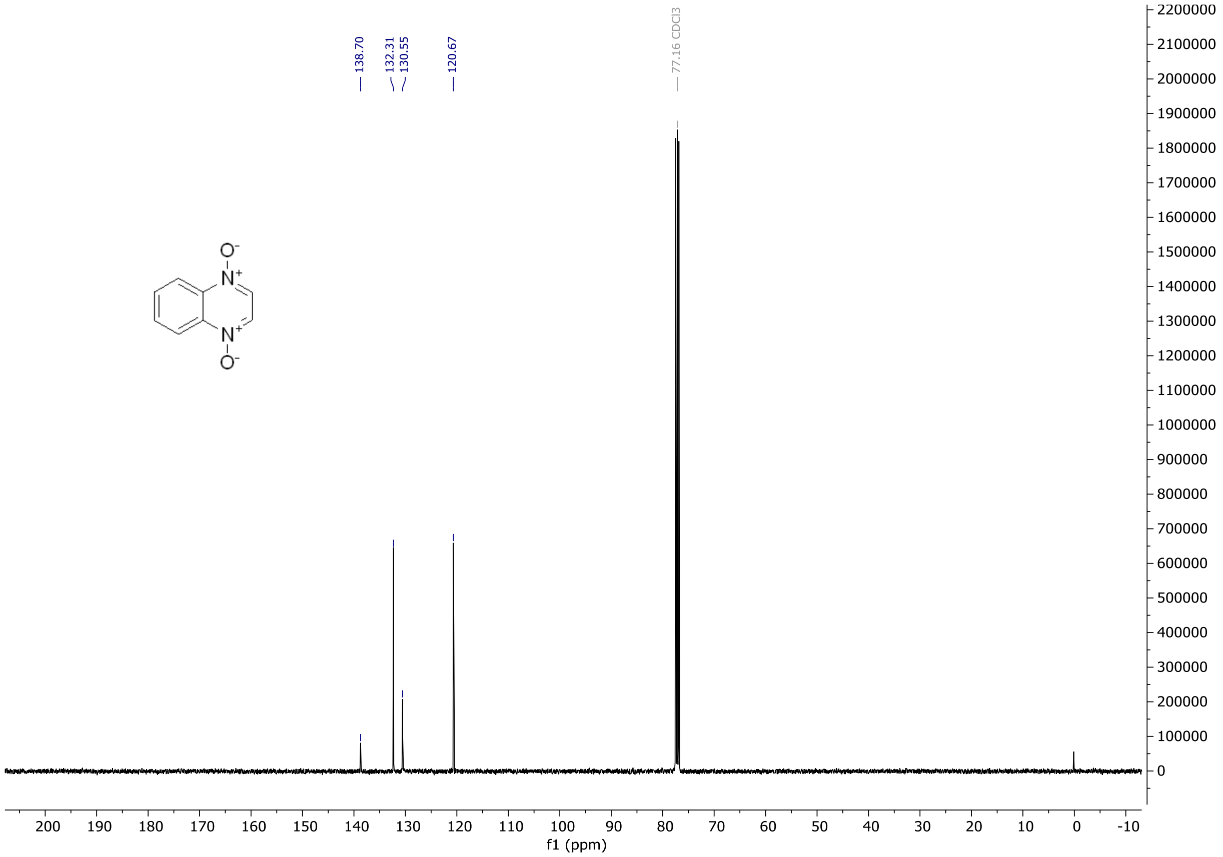

^{1}H NMR (400 MHz, $CDCl_3$) of **S9**:

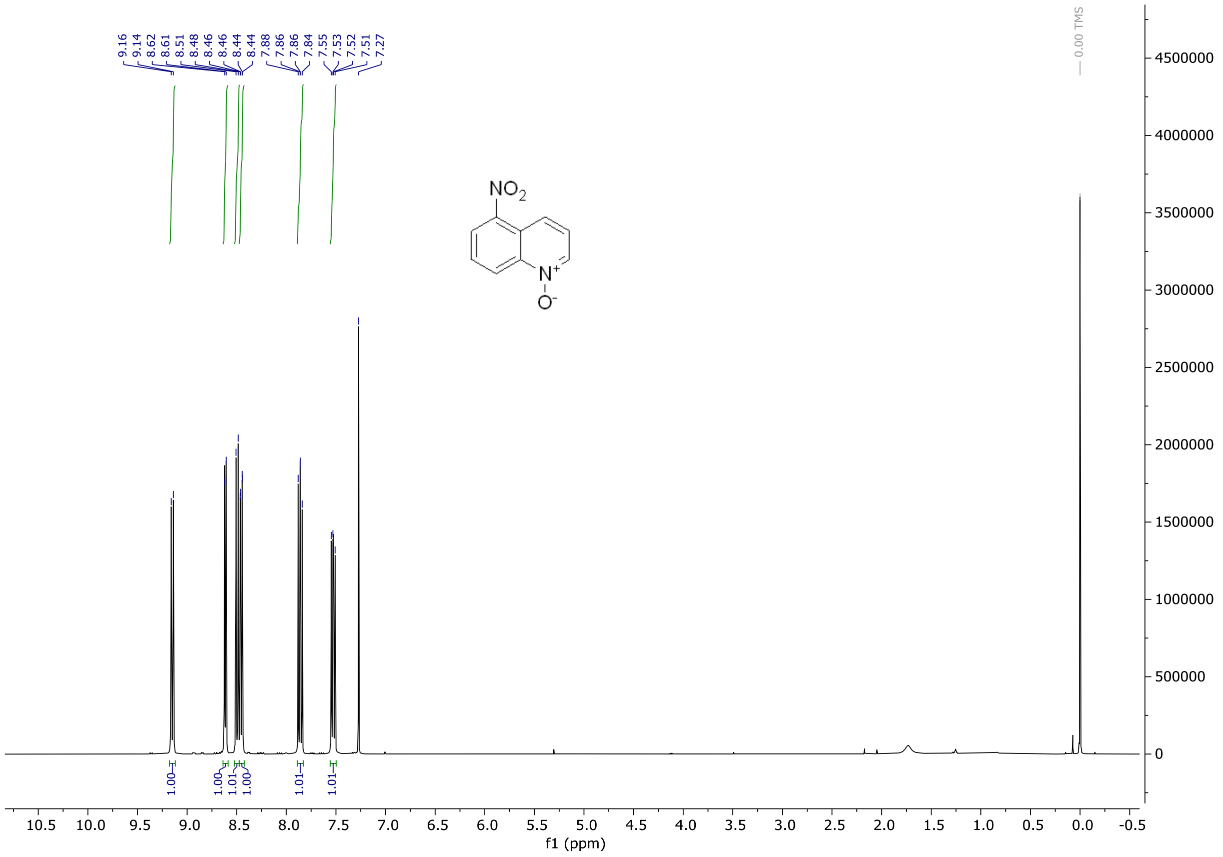

^{13}C NMR (101 MHz, $CDCl_3$) of **S9**:

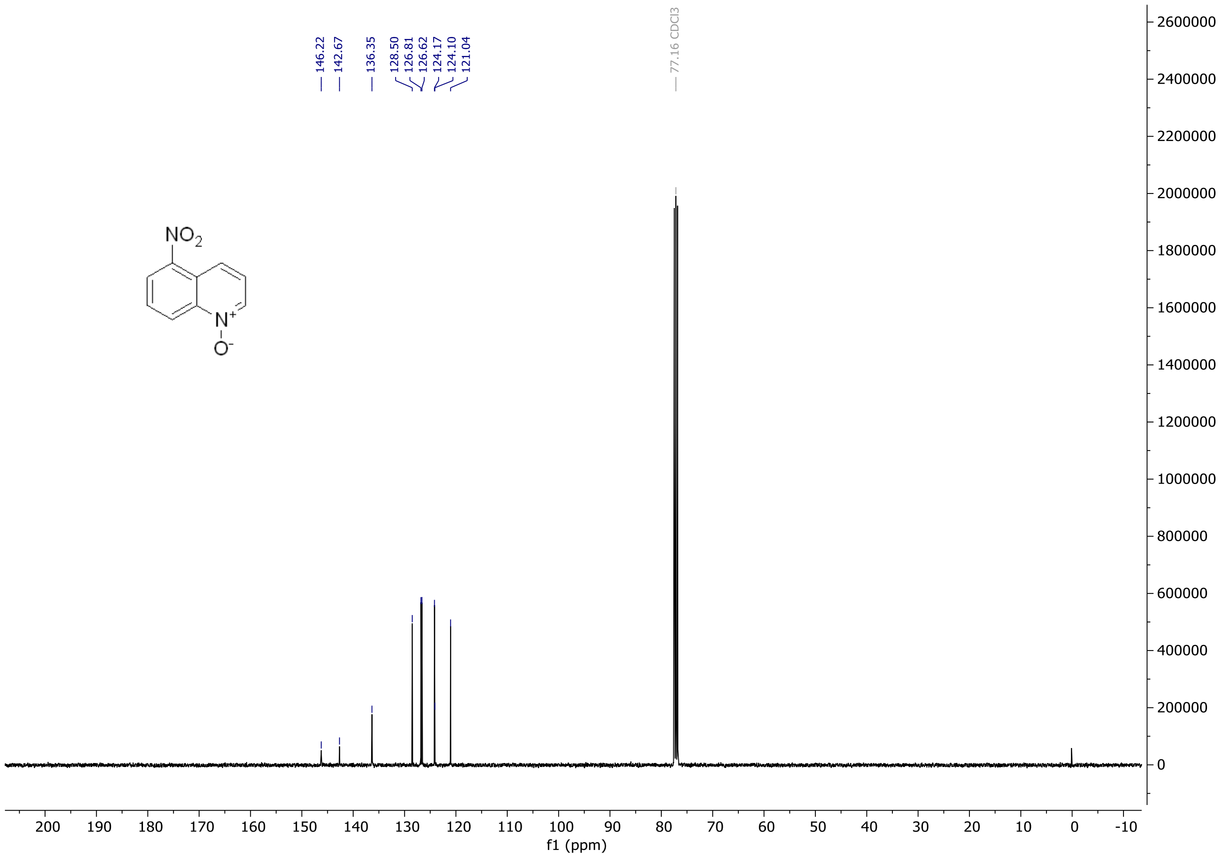

^{1}H NMR (400 MHz, $CDCl_3$) of **5**:

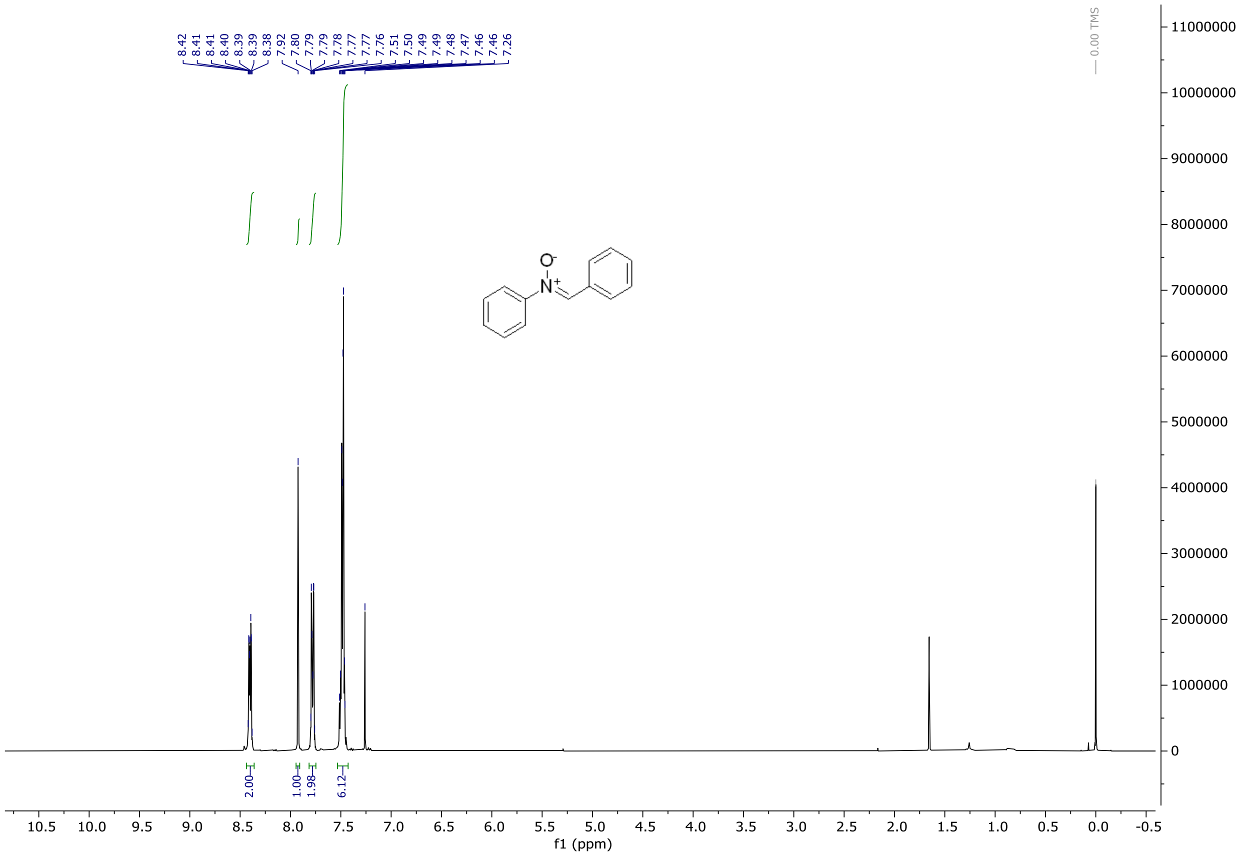

^{13}C NMR (101 MHz, $CDCl_3$) of **5**:

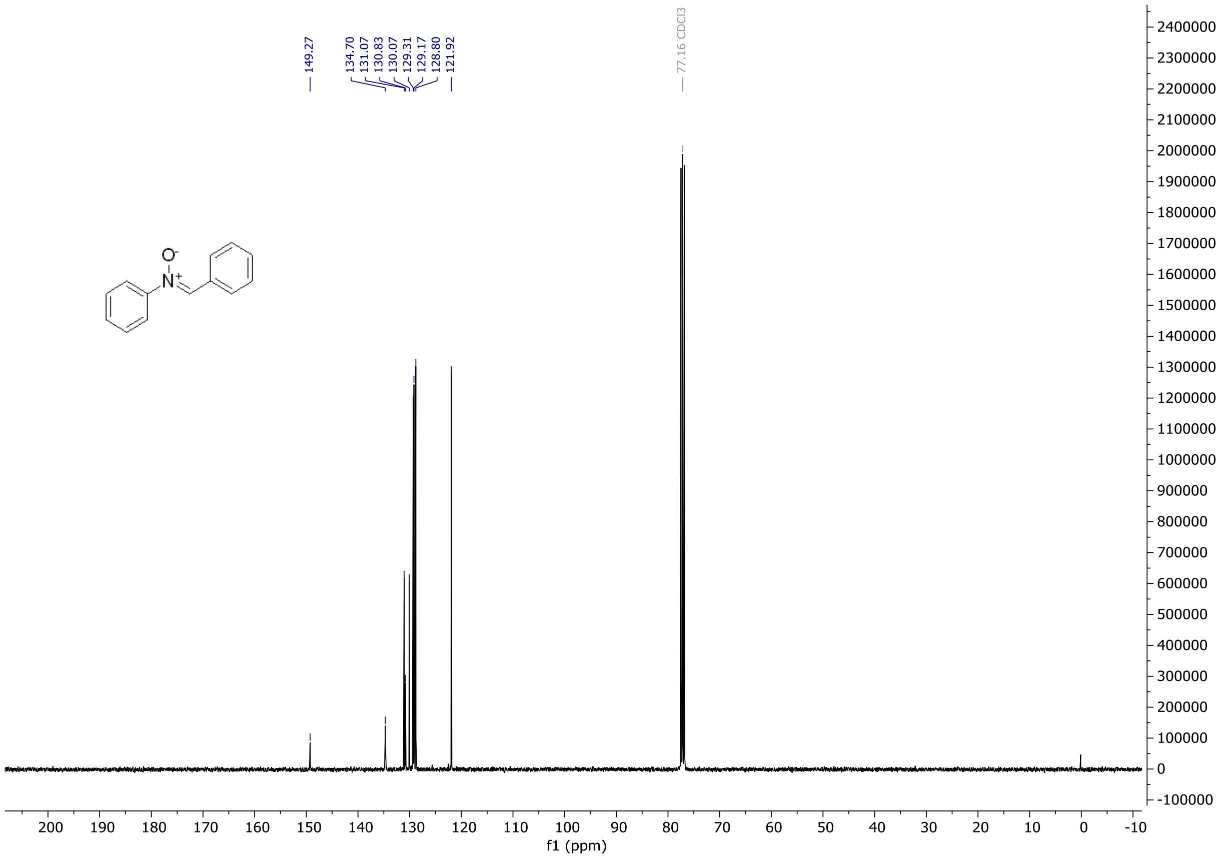

^{1}H NMR (400 MHz, $CDCl_3$) of **2a**:

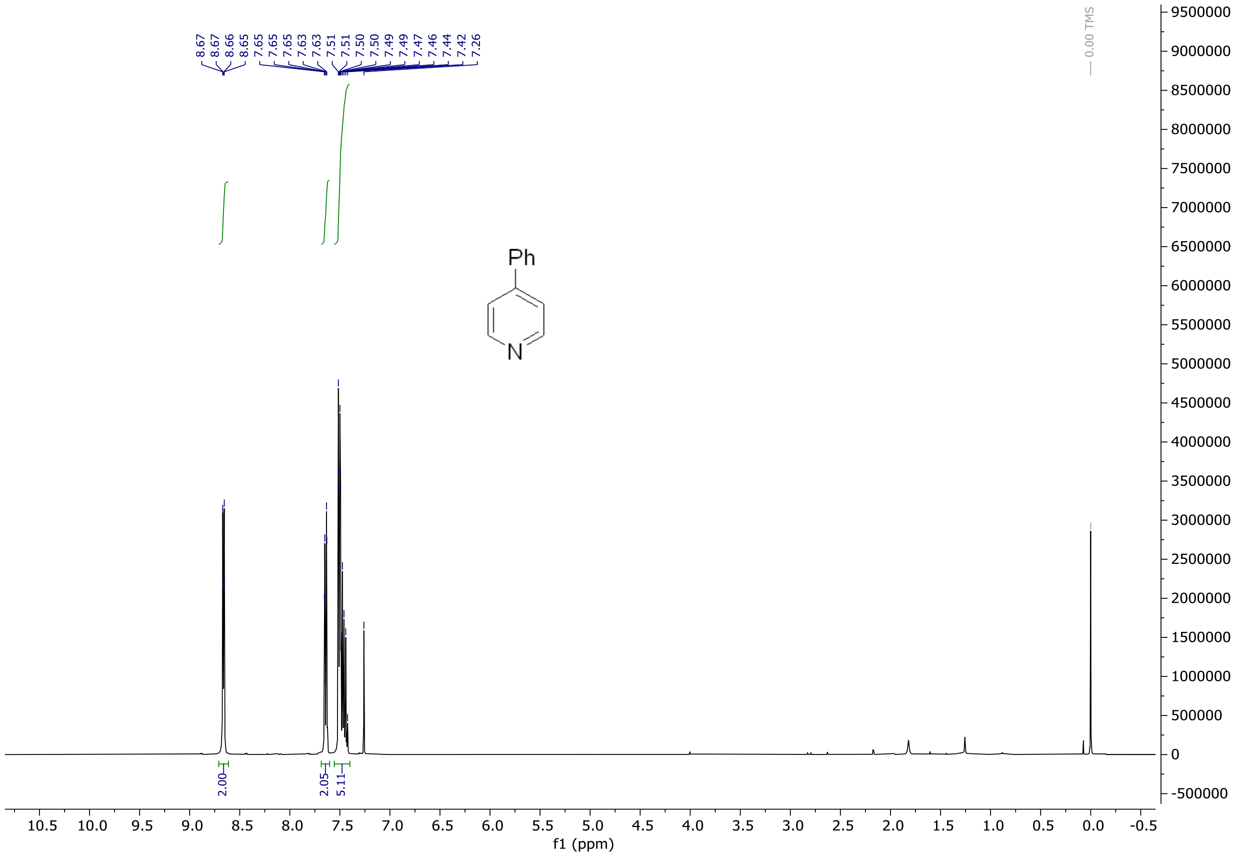

^{13}C NMR (101 MHz, $CDCl_3$) of **2a**:

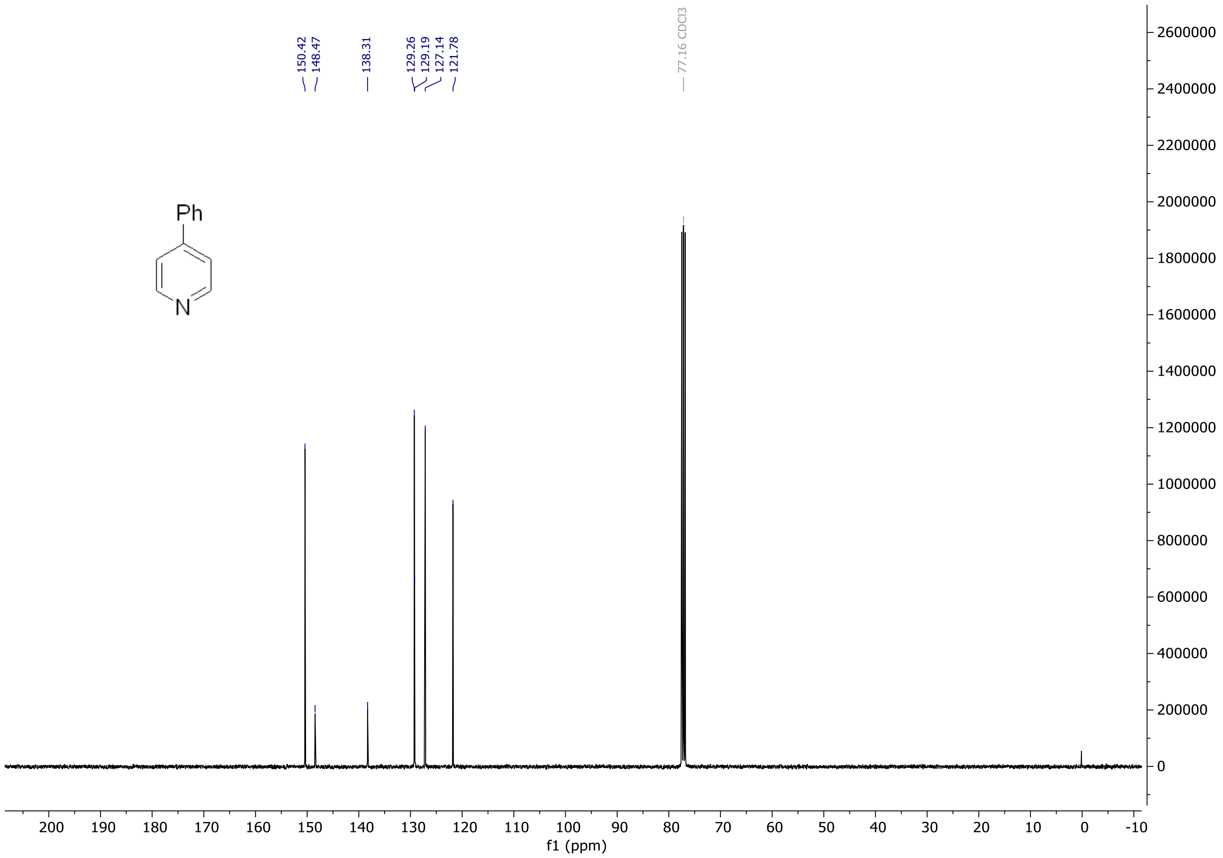

^{1}H NMR (400 MHz, $CDCl_3$) of **2b**:

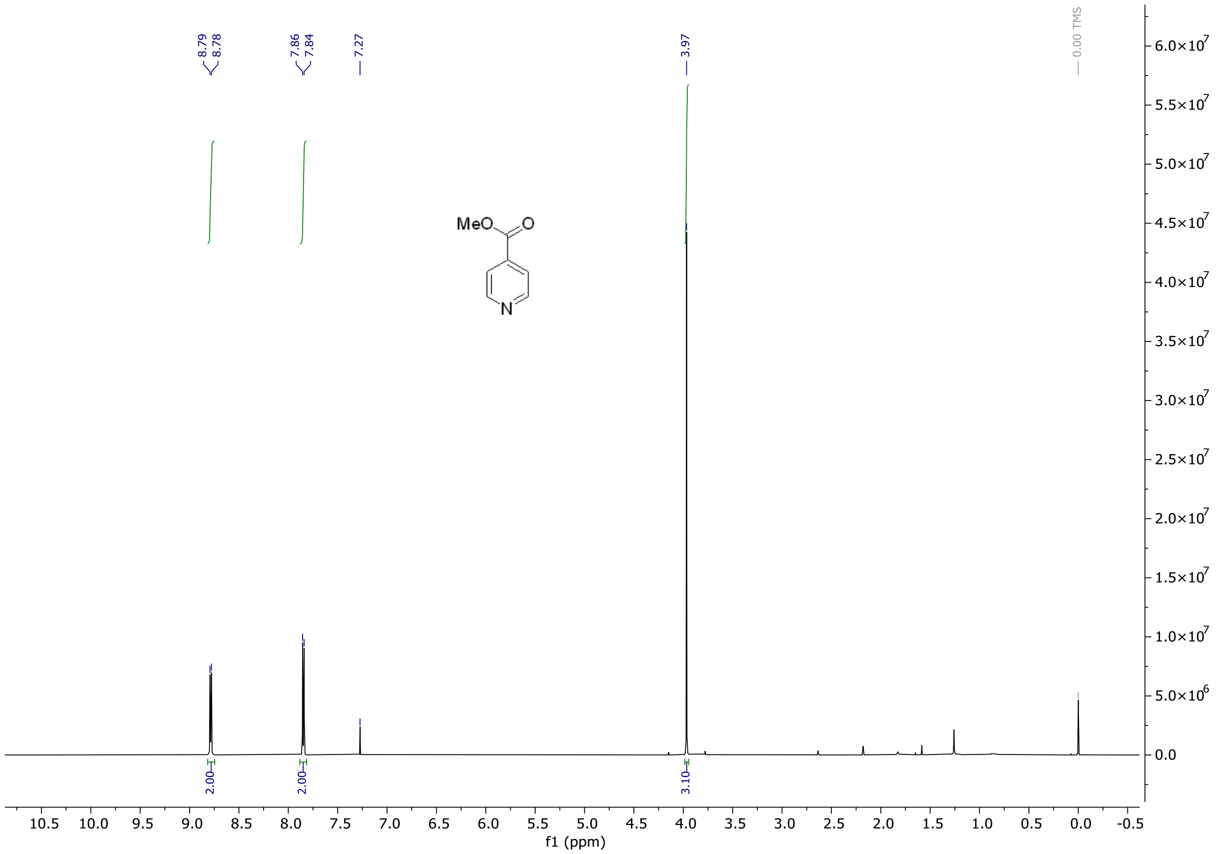

^{13}C NMR (101 MHz, $CDCl_3$) of **2b**:

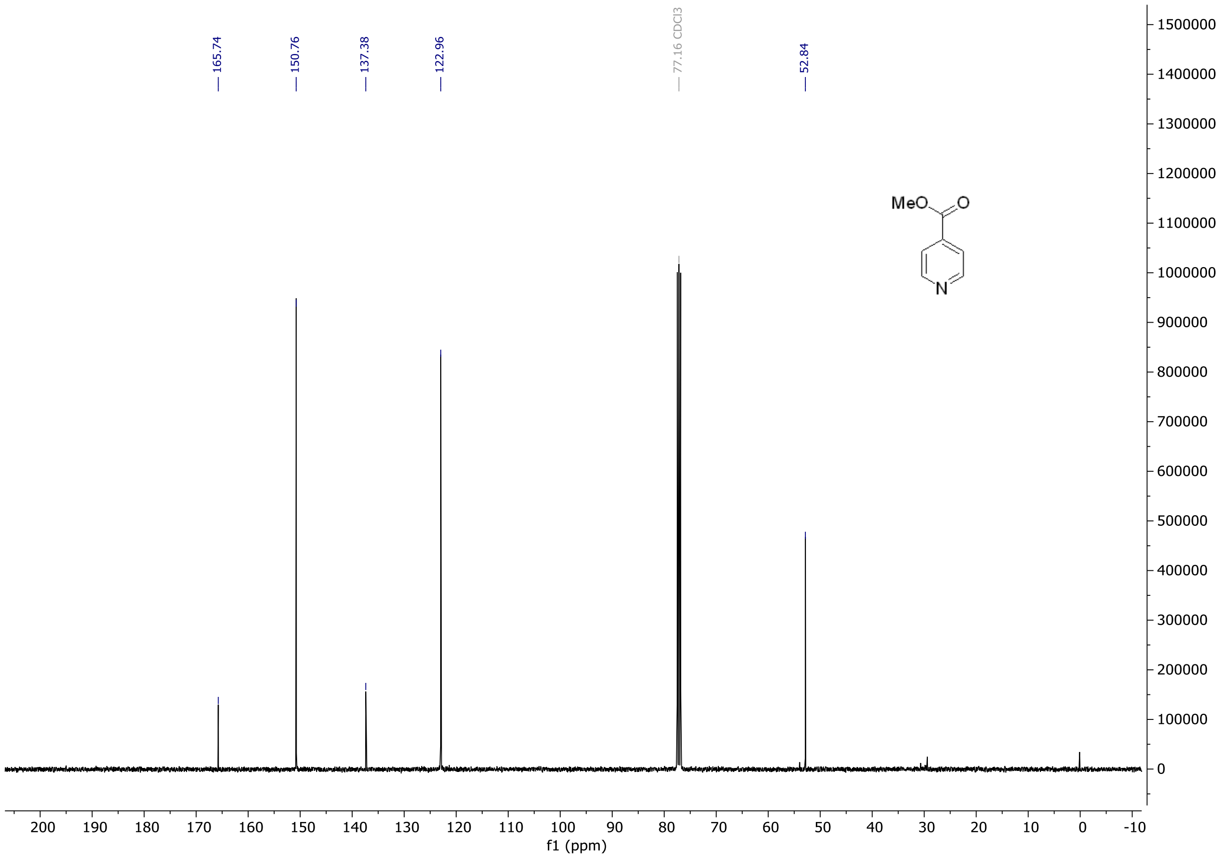

^{1}H NMR (400 MHz, $CDCl_3$) of **2c**:

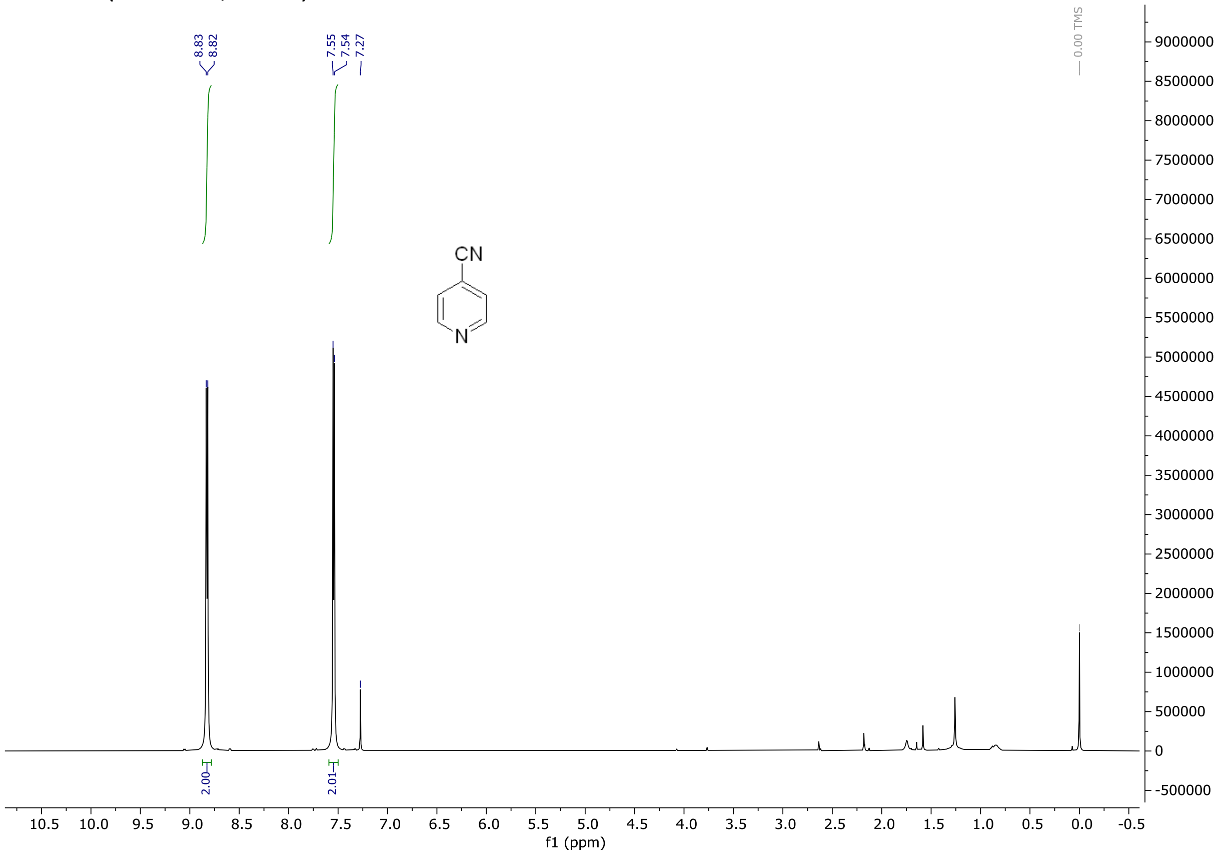

^{13}C NMR (101 MHz, $CDCl_3$) of **2c**:

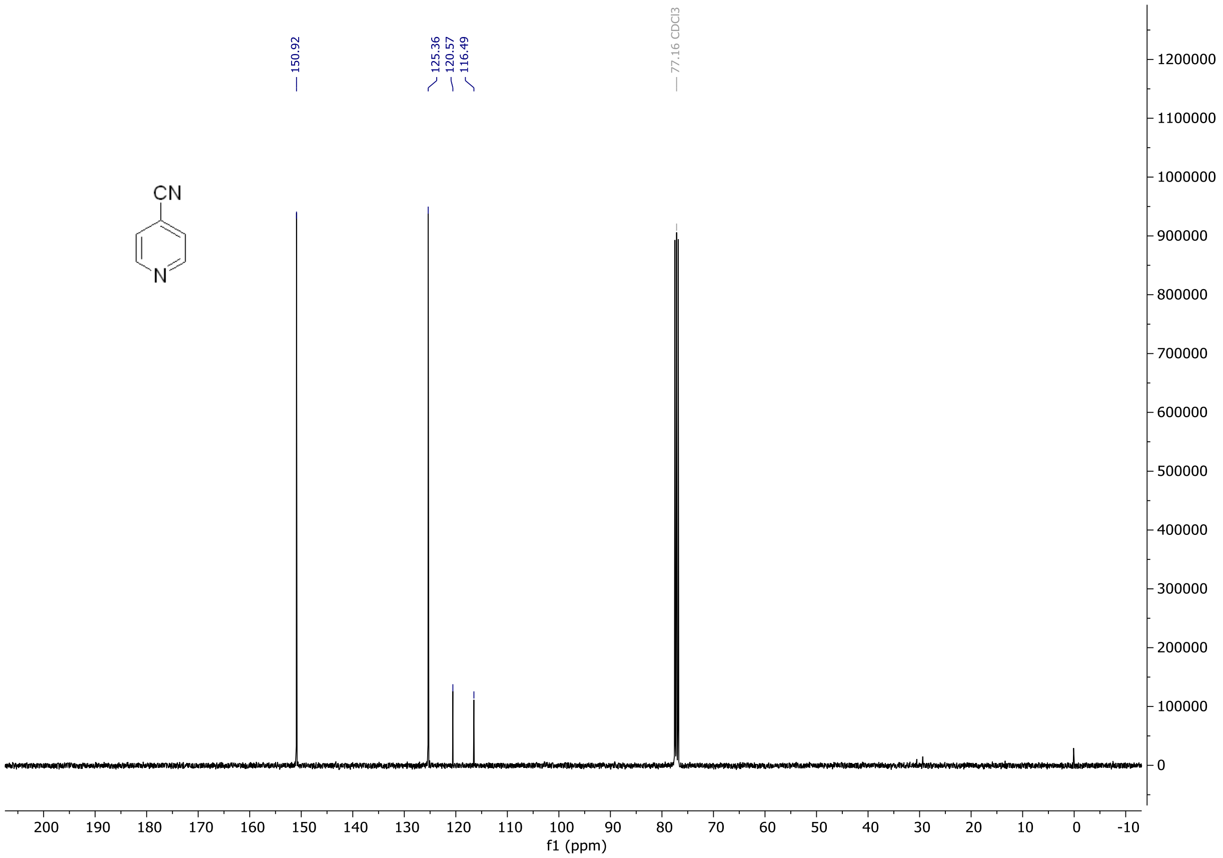

^{1}H NMR (400 MHz, $CDCl_3$) of **2d**:

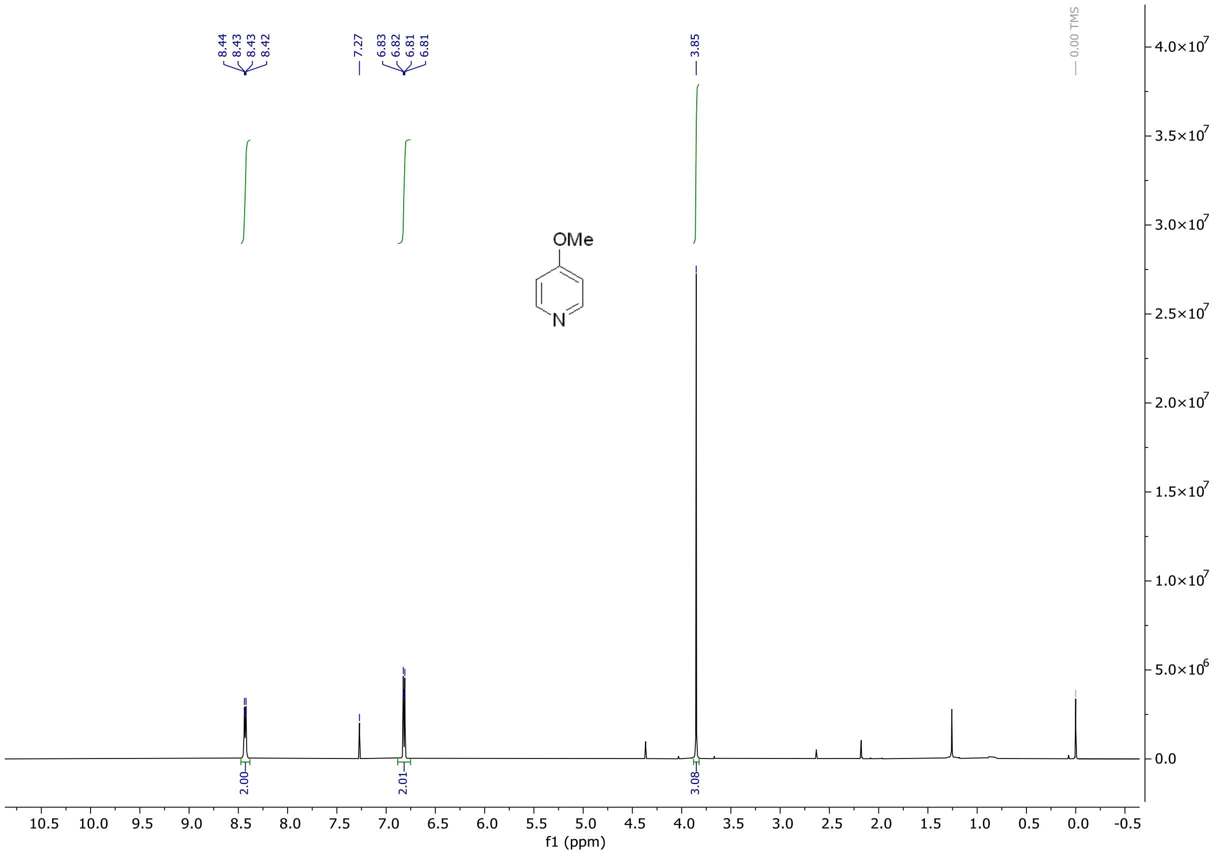

^{13}C NMR (101 MHz, $CDCl_3$) of **2d**:

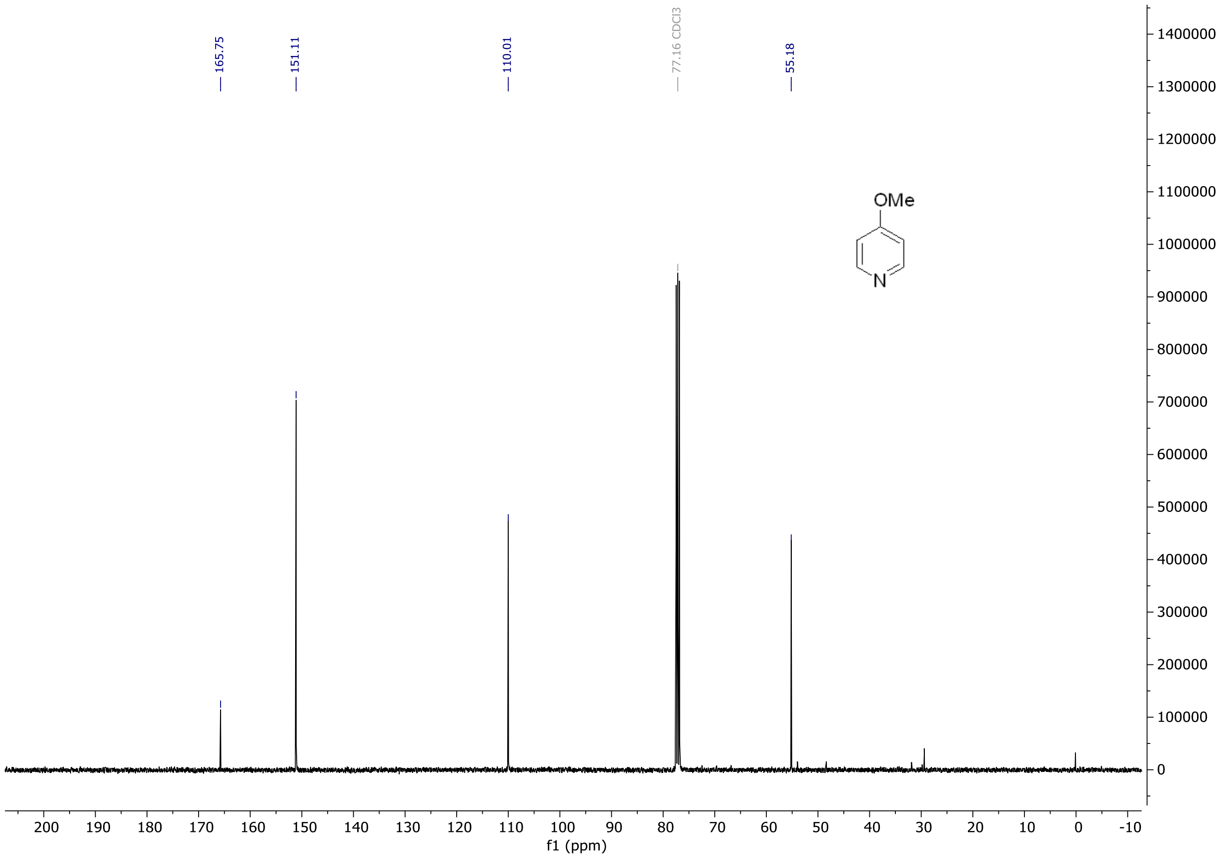

^{1}H NMR (400 MHz, $CDCl_3$) of **2e**:

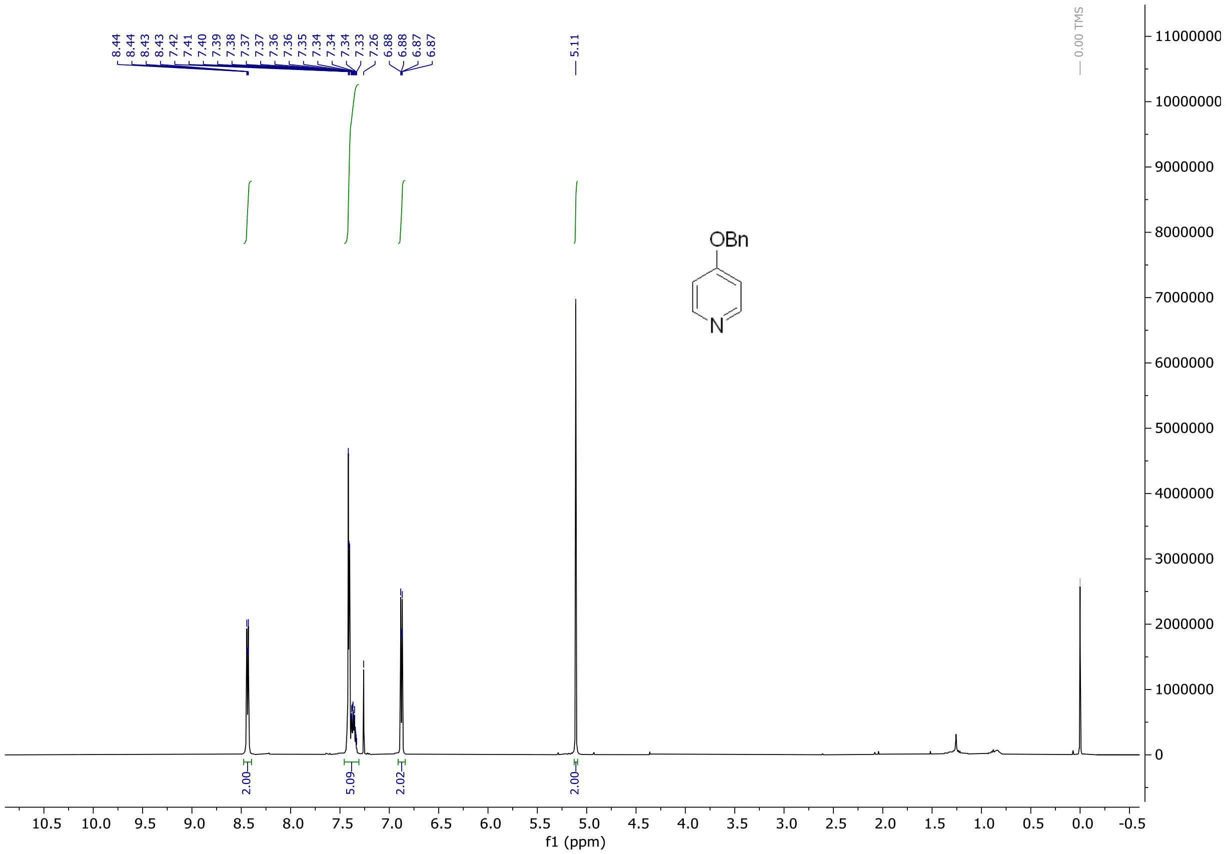

^{13}C NMR (101 MHz, $CDCl_3$) of **2e**:

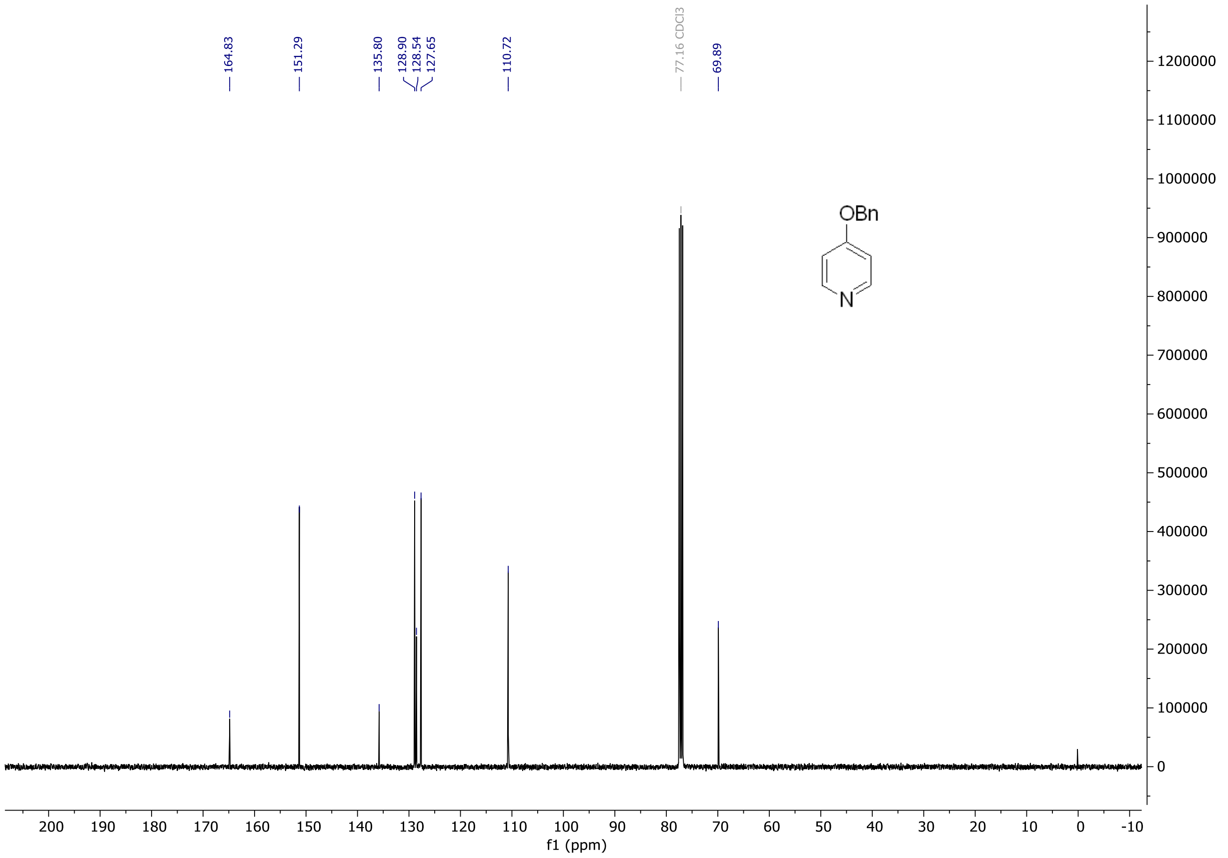

^{1}H NMR (400 MHz, $CDCl_3$) of **2f**:

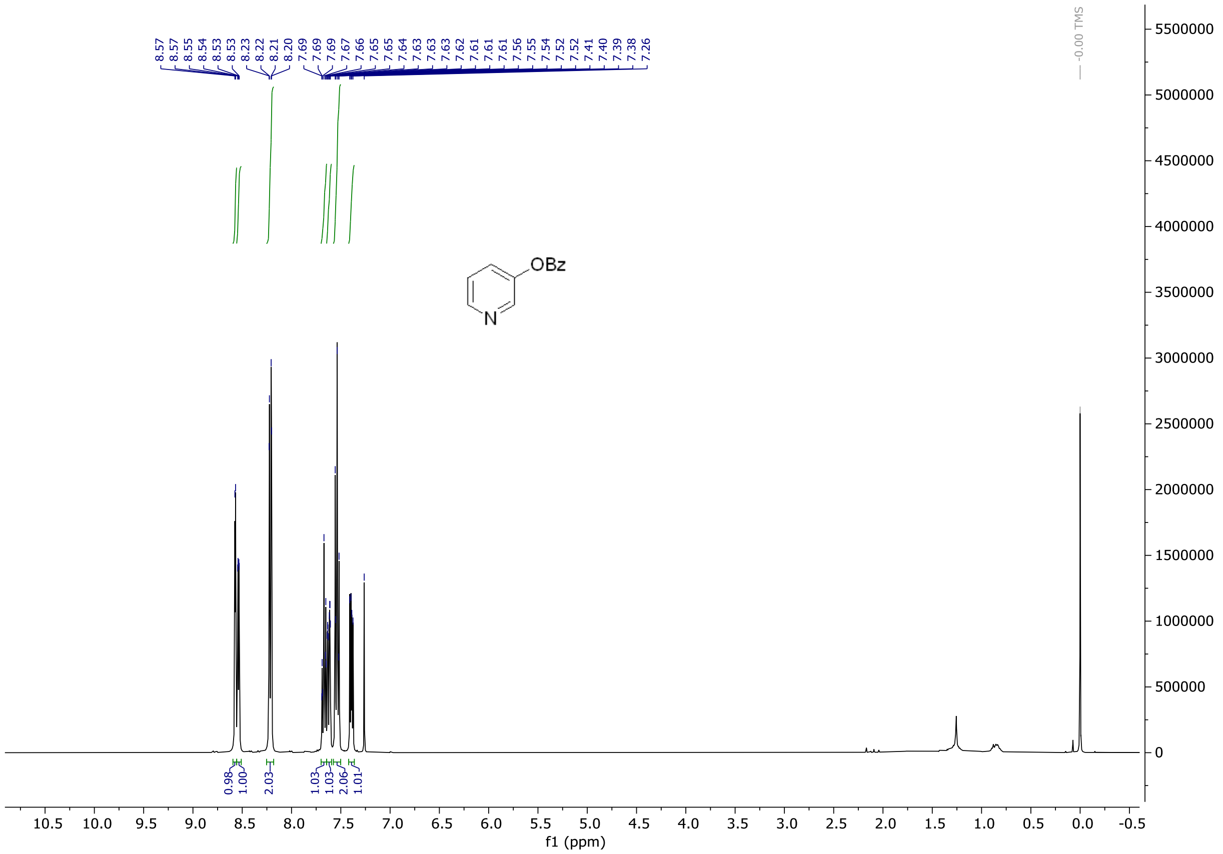

^{13}C NMR (101 MHz, $CDCl_3$) of **2f**:

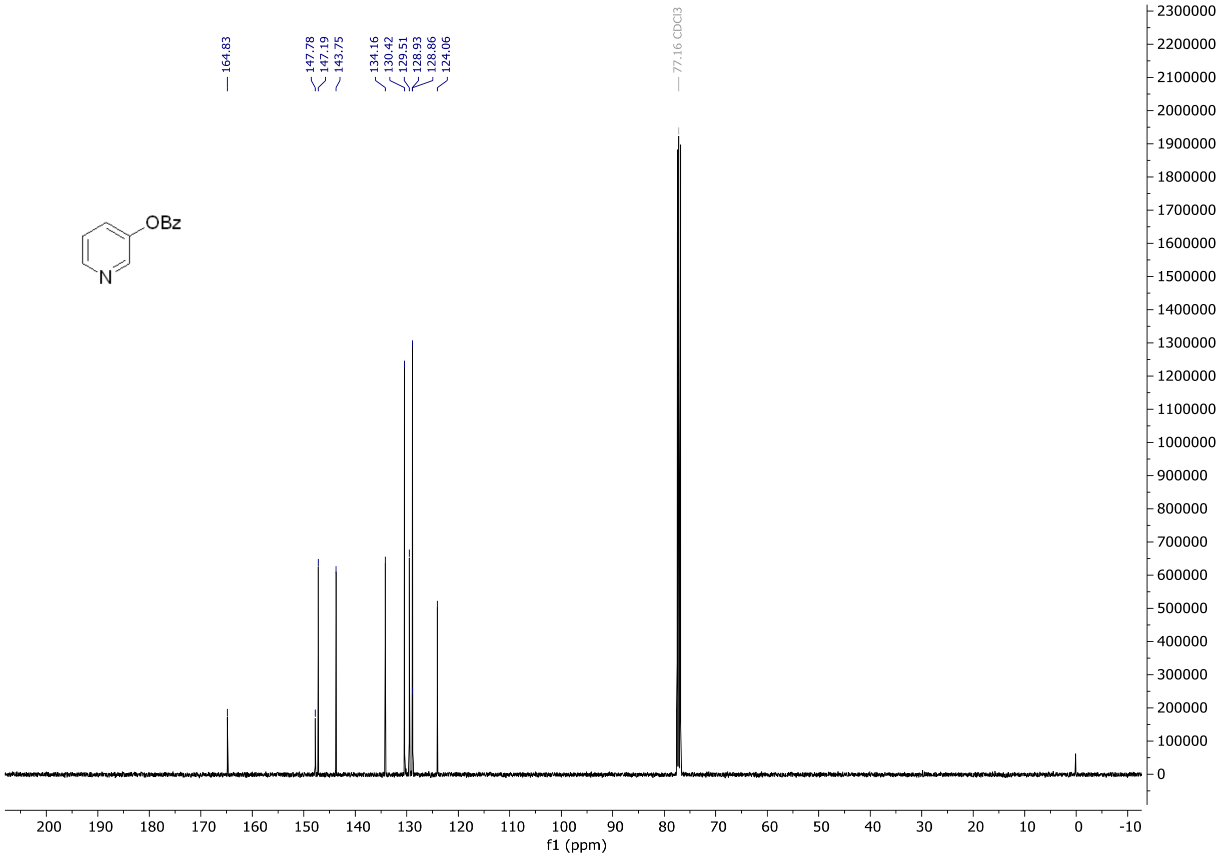

^{1}H NMR (400 MHz, $CDCl_3$) of **2g**:

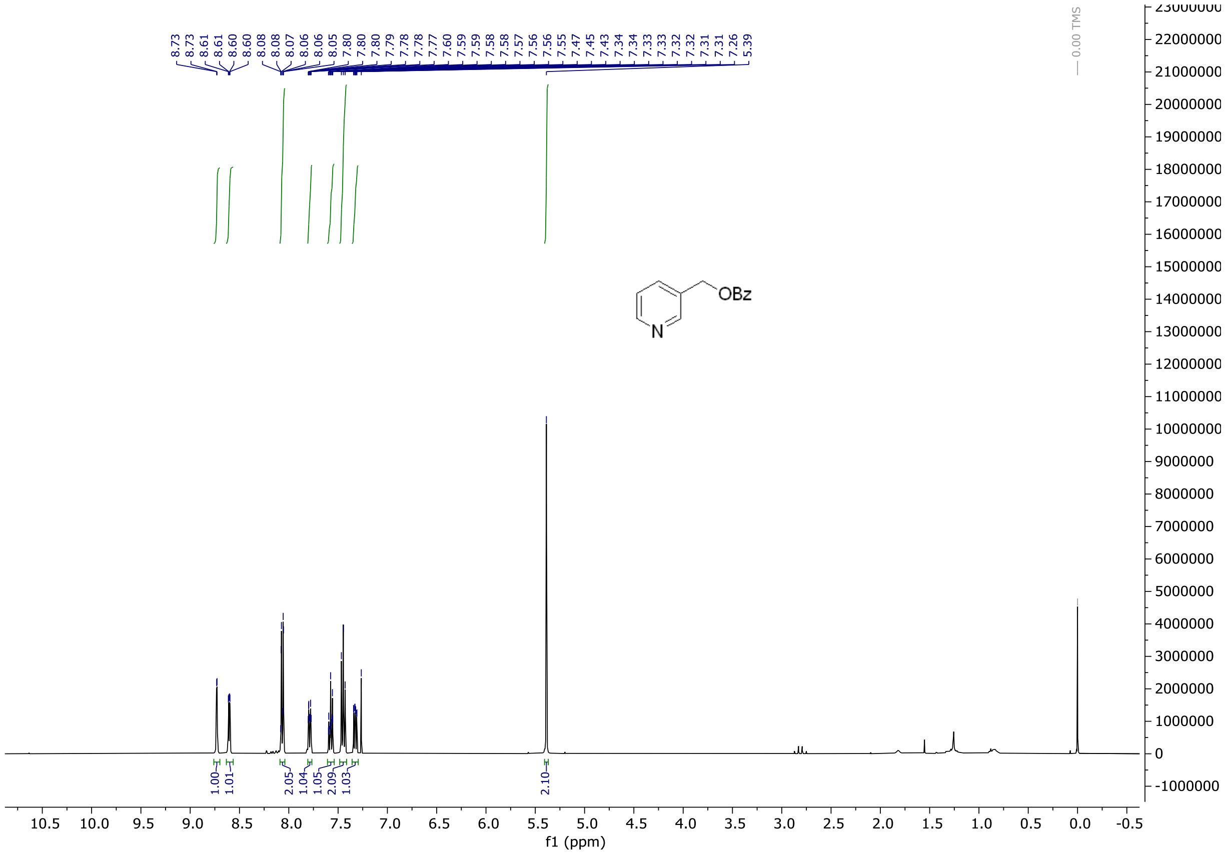

^{13}C NMR (101 MHz, $CDCl_3$) of **2g**:

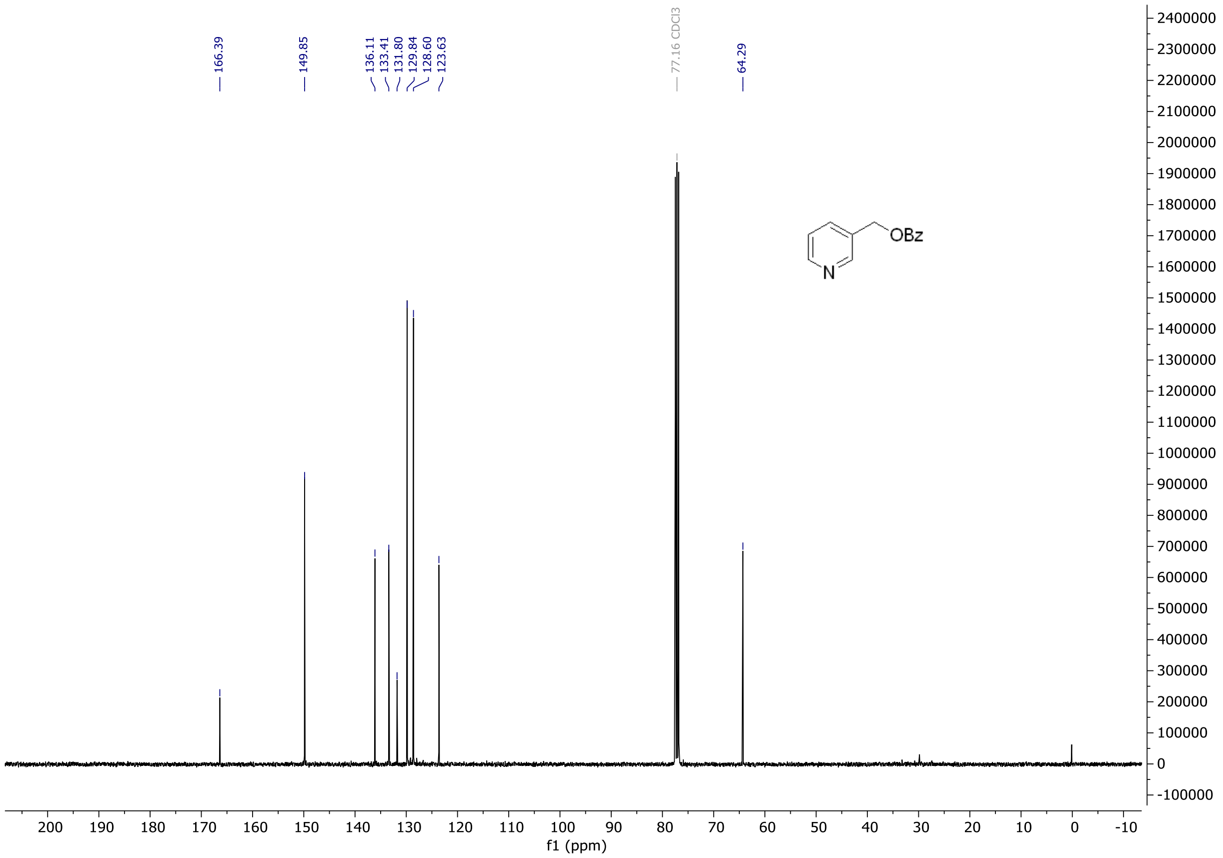

^{1}H NMR (400 MHz, $CDCl_3$) of **2h**:

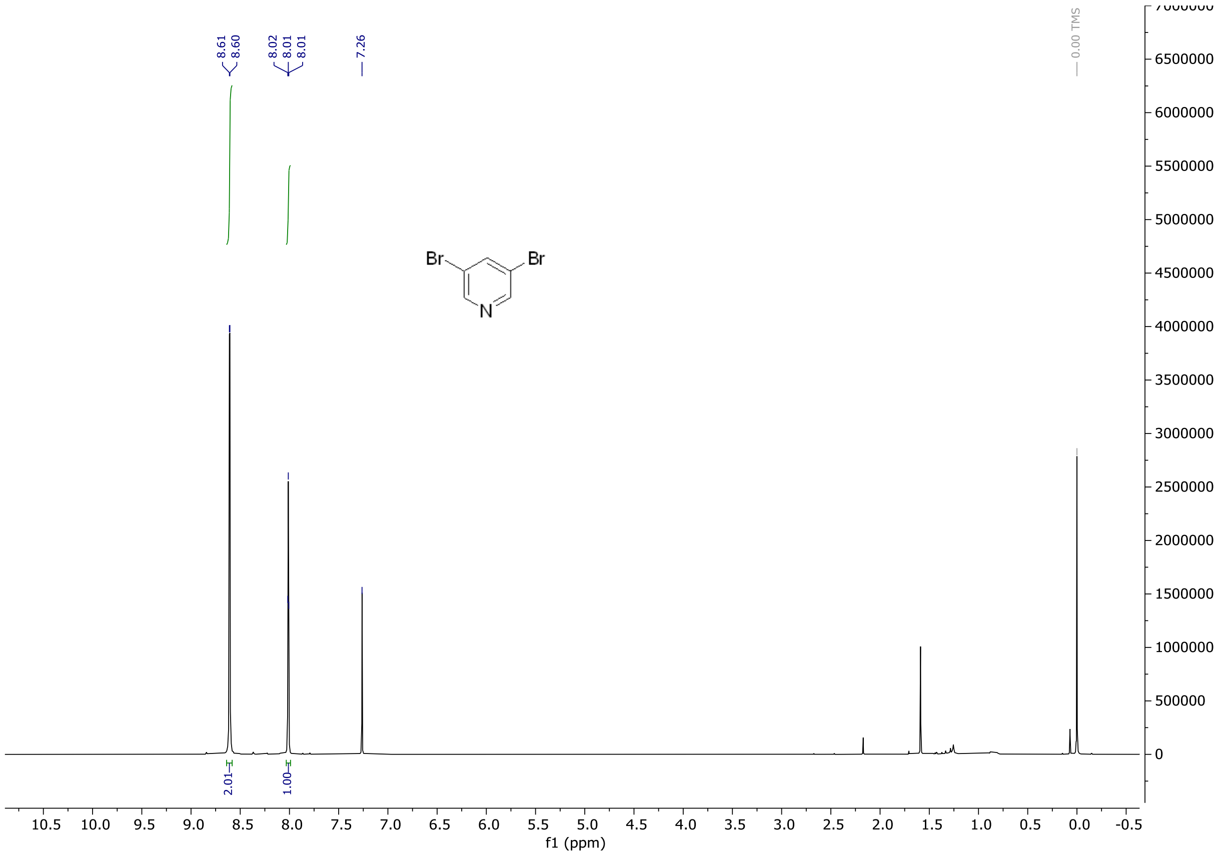

^{13}C NMR (101 MHz, $CDCl_3$) of **2h**:

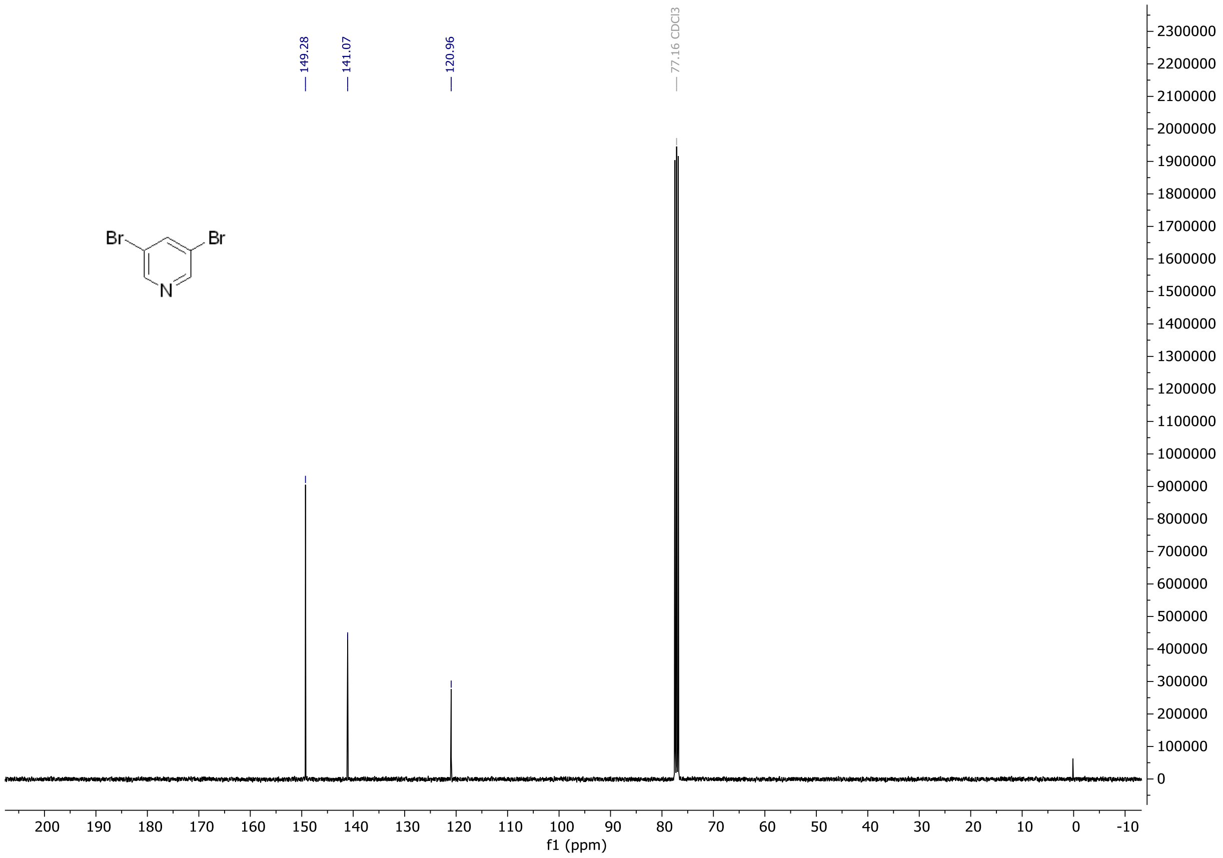

^{1}H NMR (400 MHz, $CDCl_3$) of **2i**:

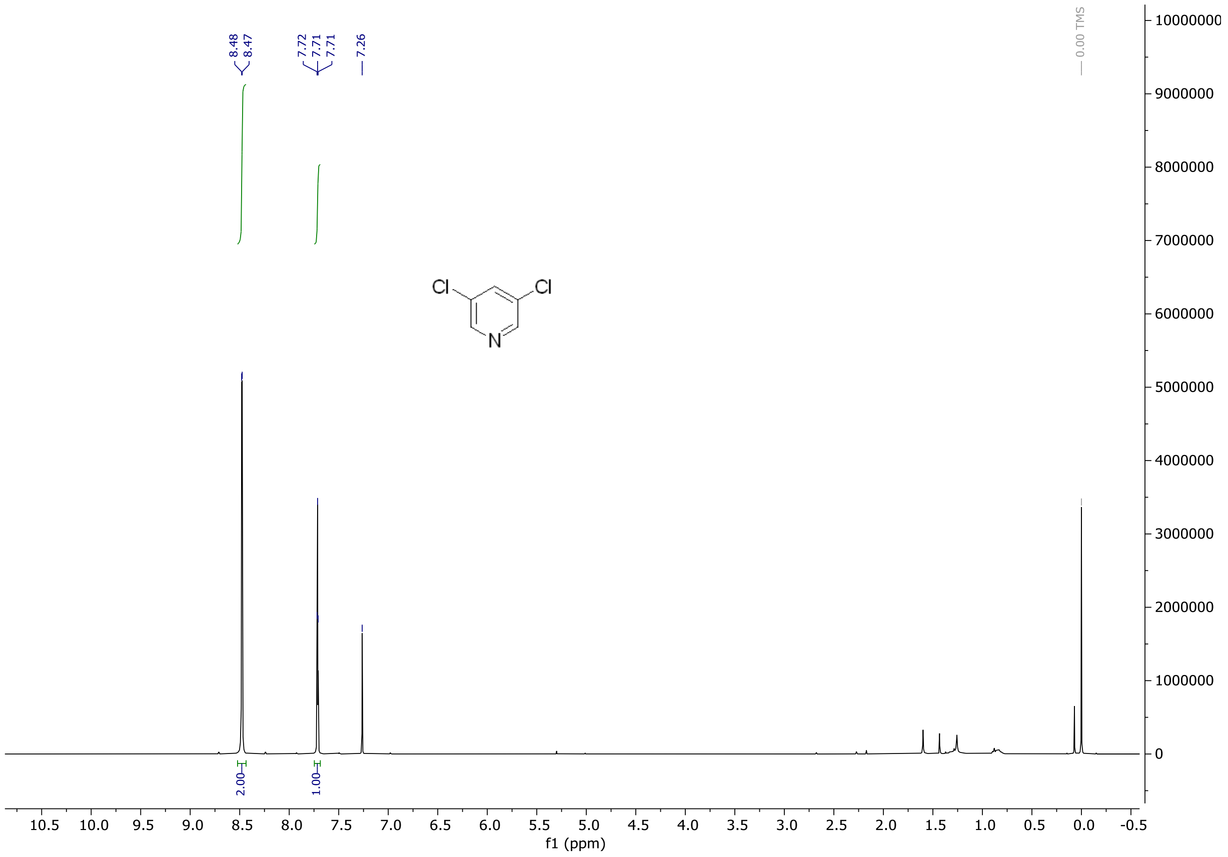

^{13}C NMR (101 MHz, $CDCl_3$) of **2i**:

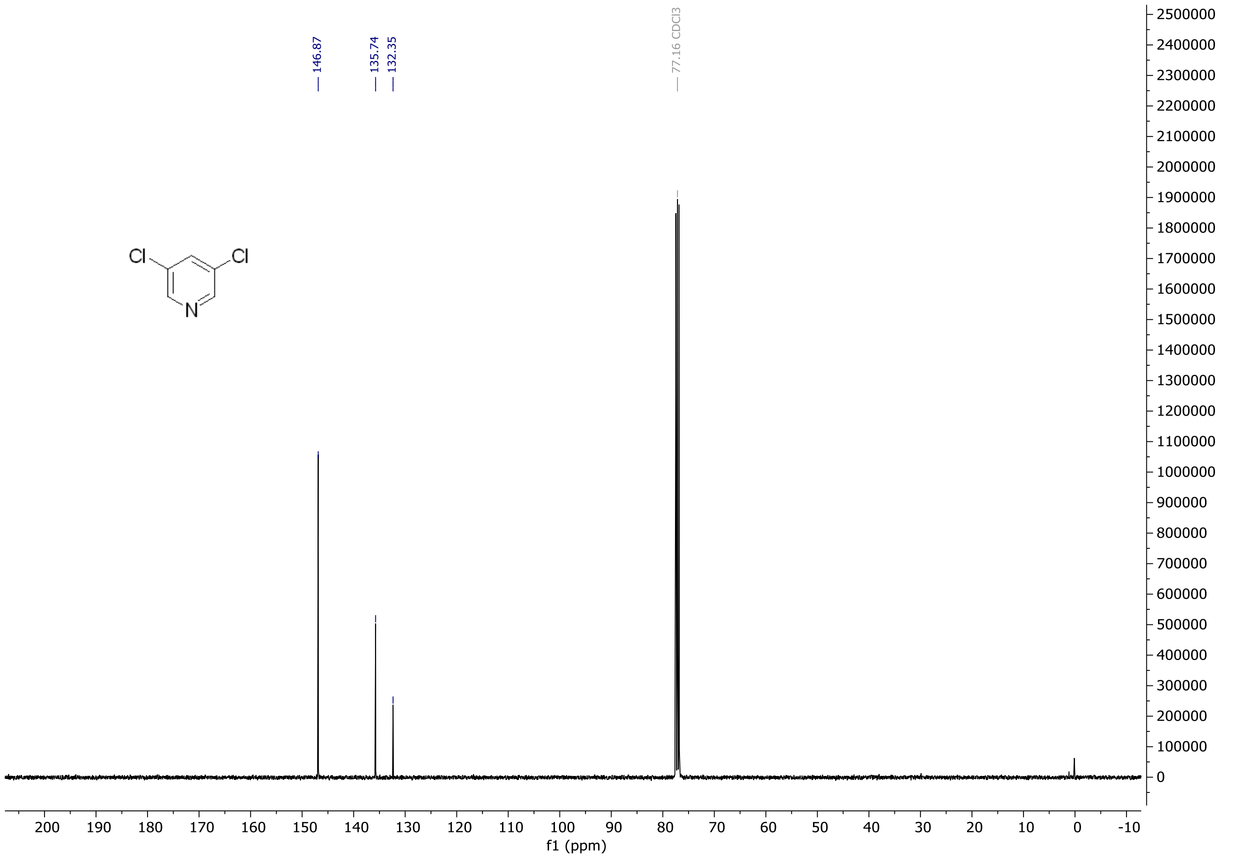

^{1}H NMR (400 MHz, $CDCl_3$) of **2j**:

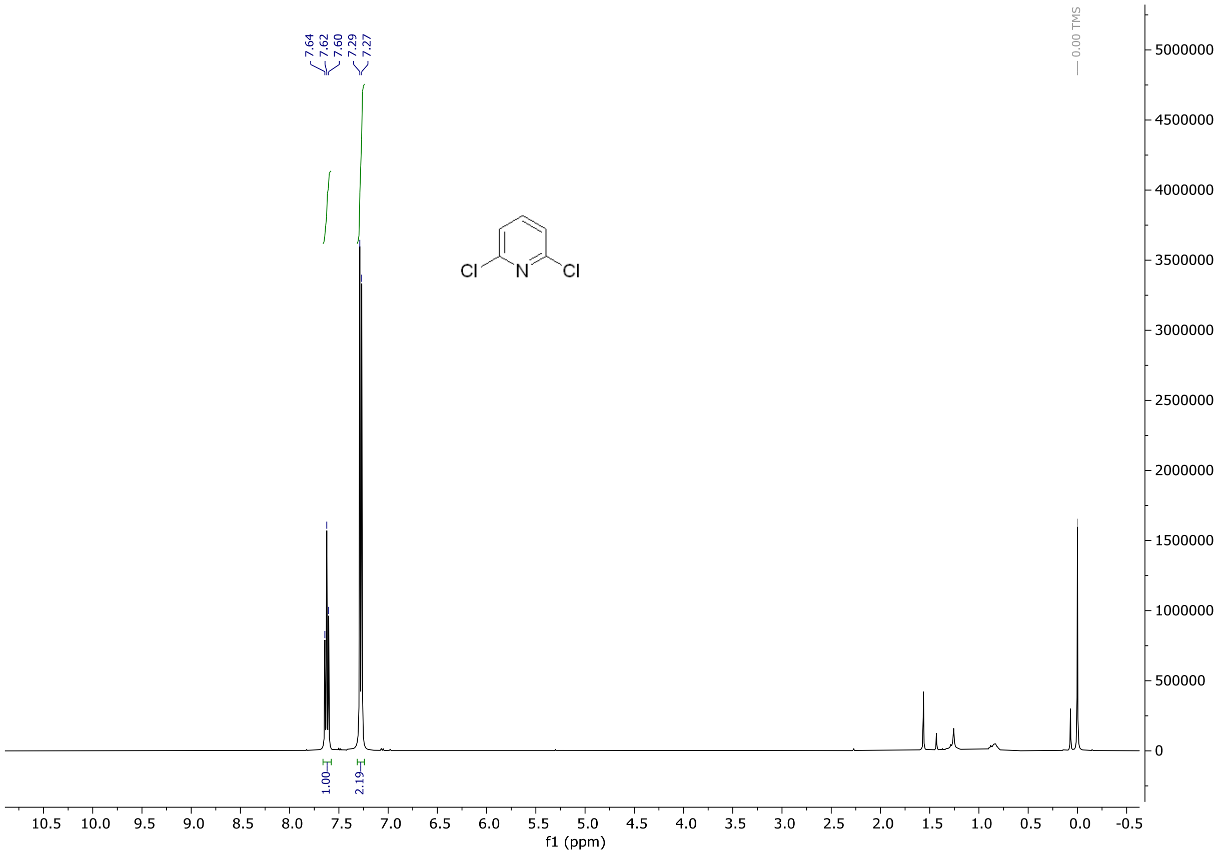

^{13}C NMR (101 MHz, $CDCl_3$) of **2j**:

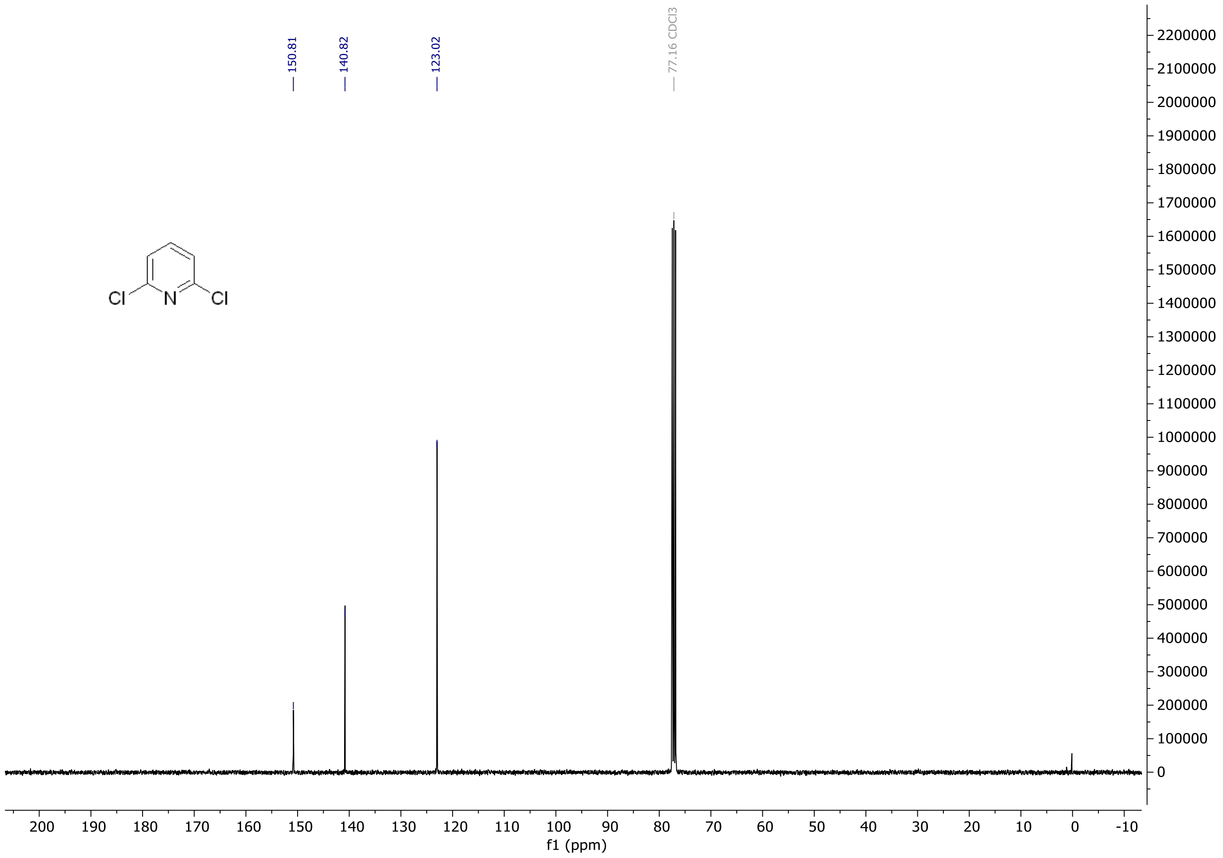

^{1}H NMR (400 MHz, $CDCl_3$) of **2k**:

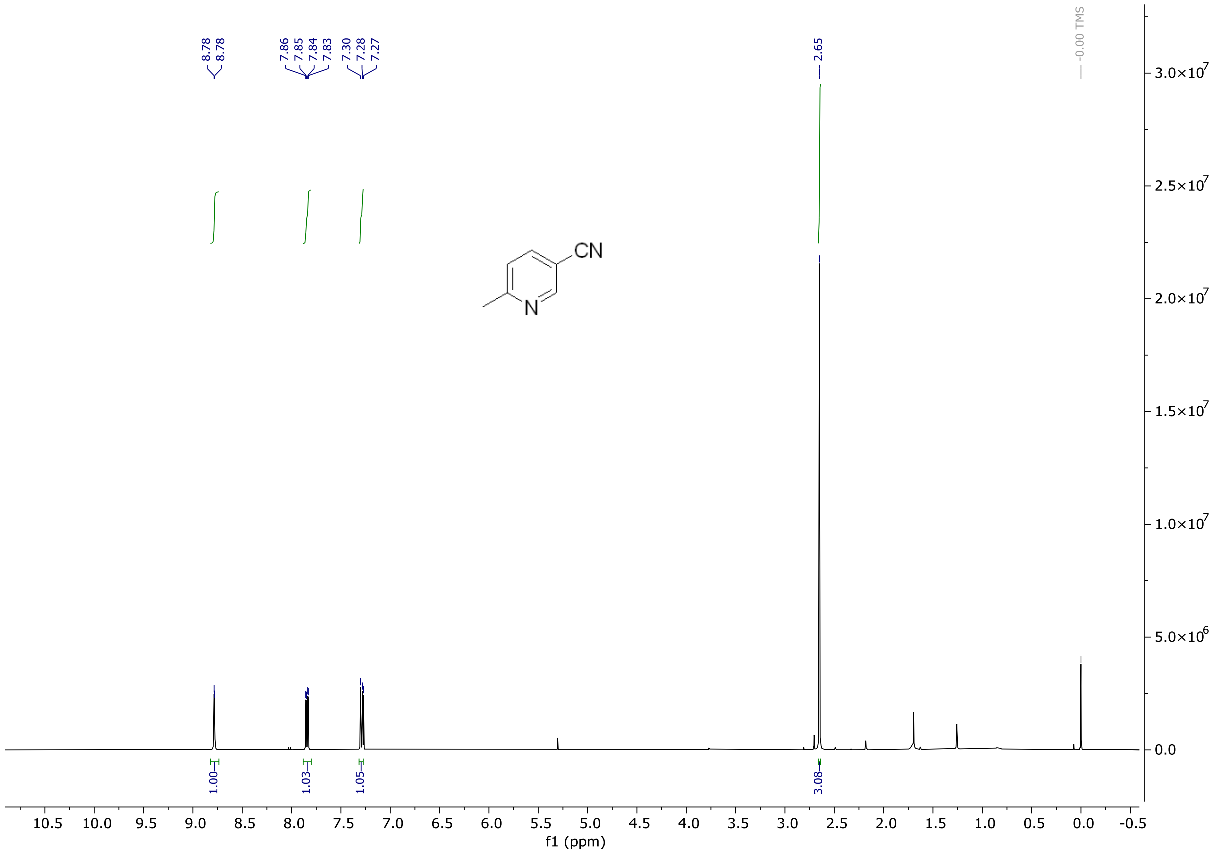

^{13}C NMR (101 MHz, $CDCl_3$) of **2k**:

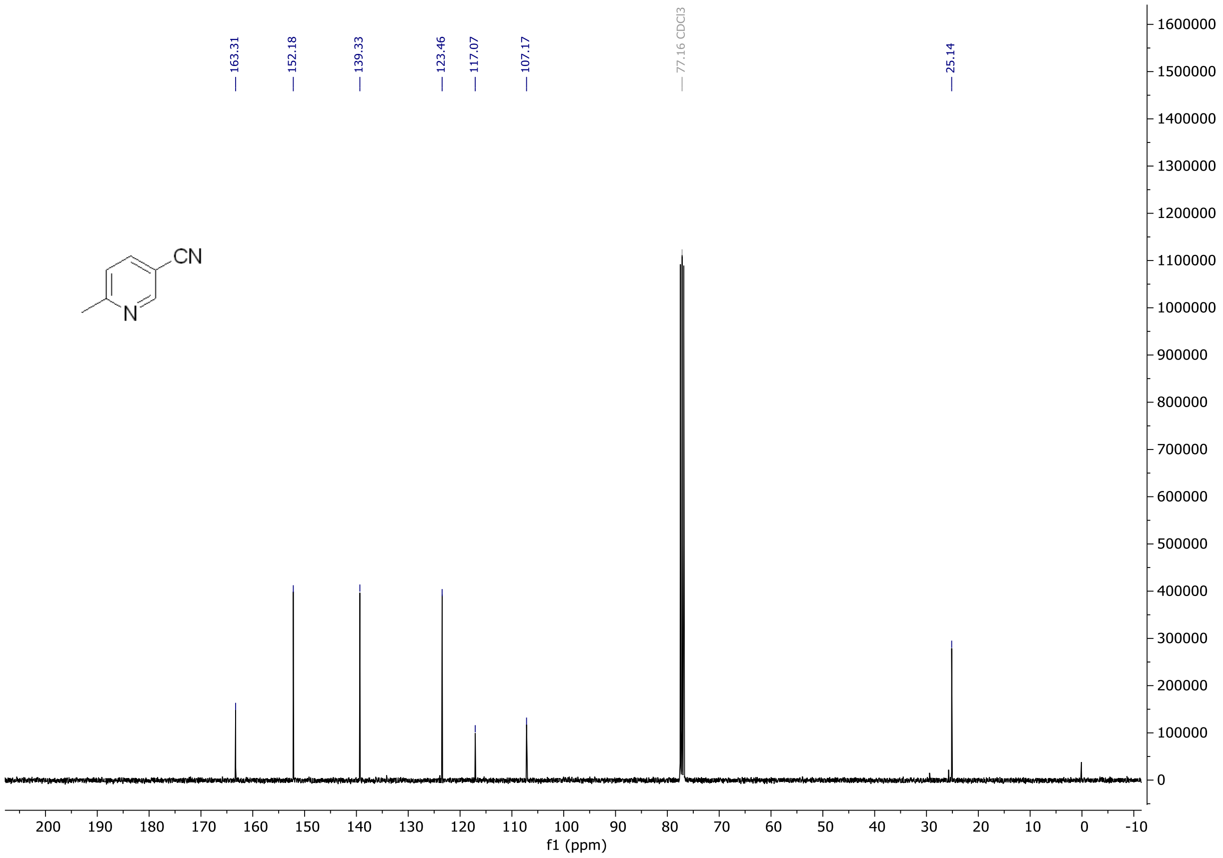

^{1}H NMR (400 MHz, $CDCl_3$) of **4a**:

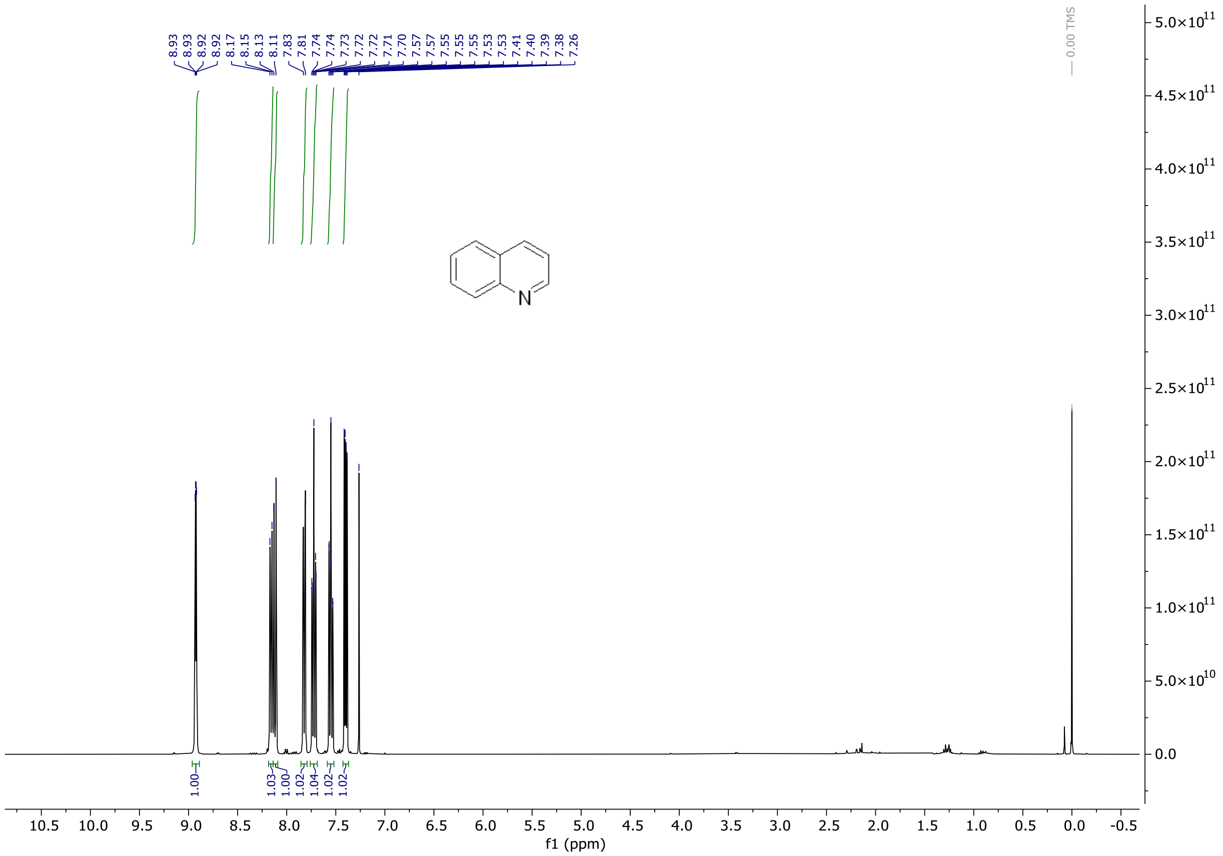

^{13}C NMR (101 MHz, $CDCl_3$) of **4a**:

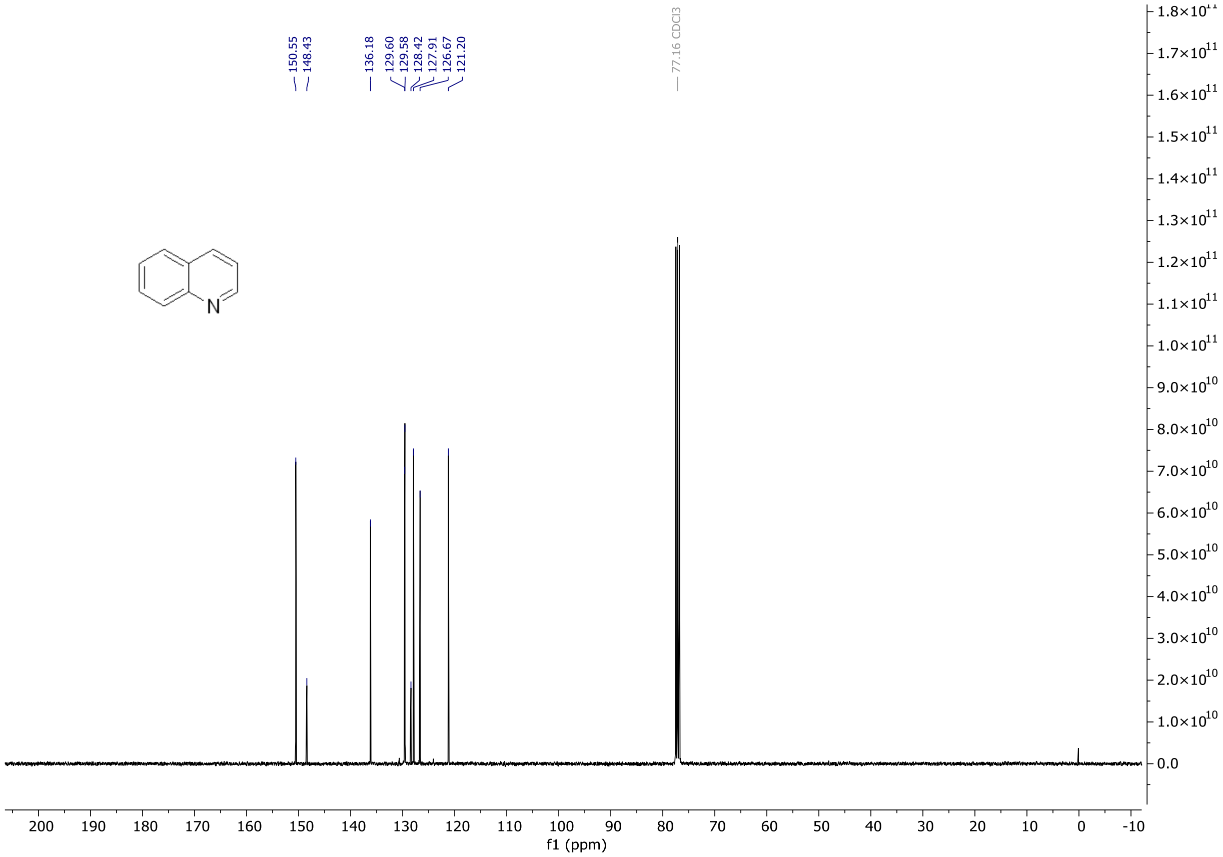

^1H NMR (400 MHz, $CDCl_3$) of **4b**:

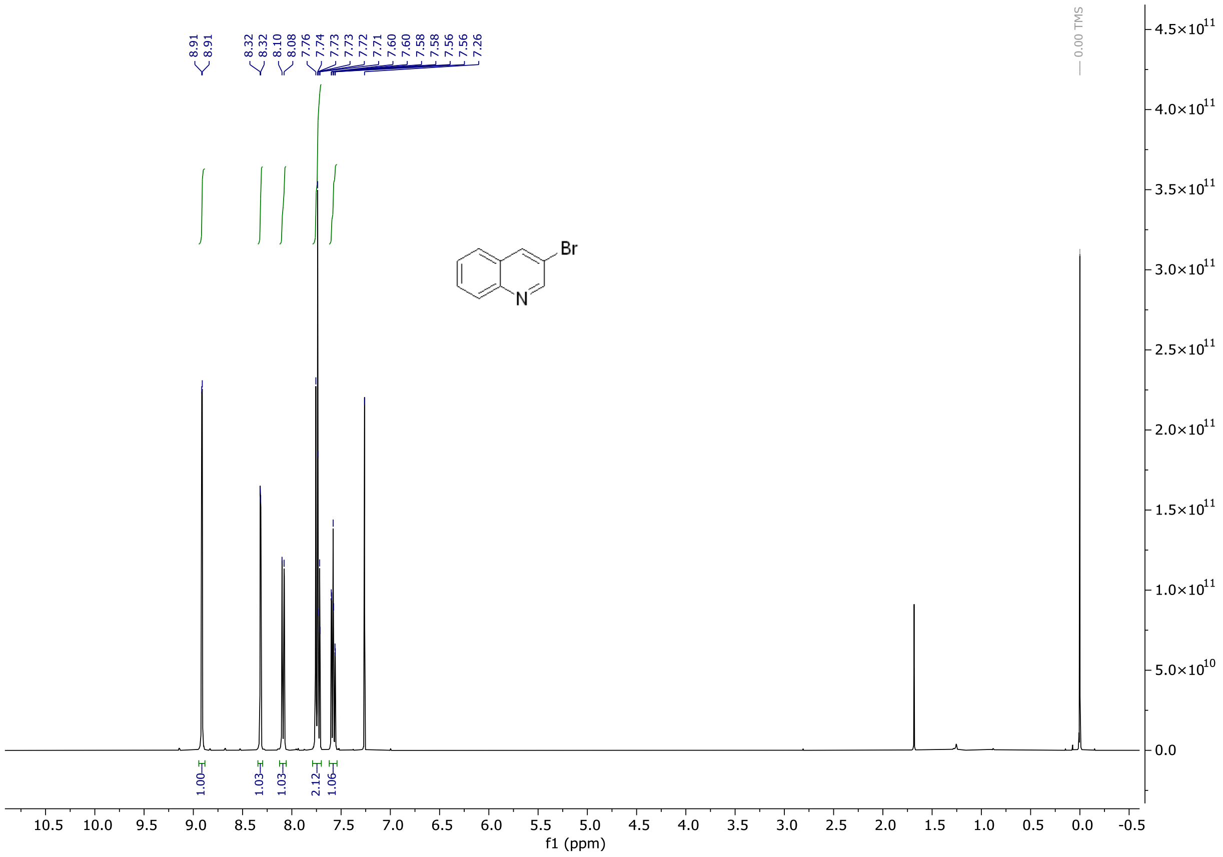

^{13}C NMR (101 MHz, $CDCl_3$) of **4b**:

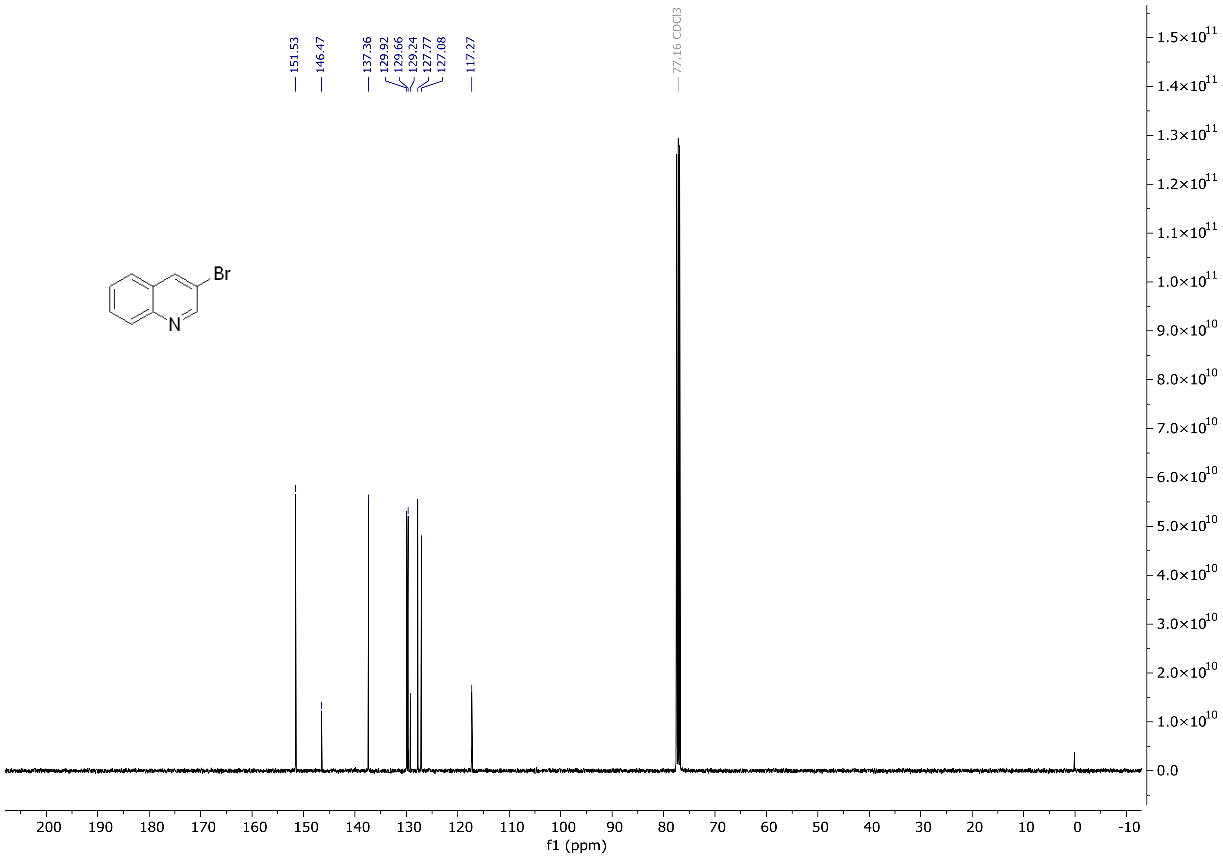

^{1}H NMR (400 MHz, $CDCl_3$) of **4c**:

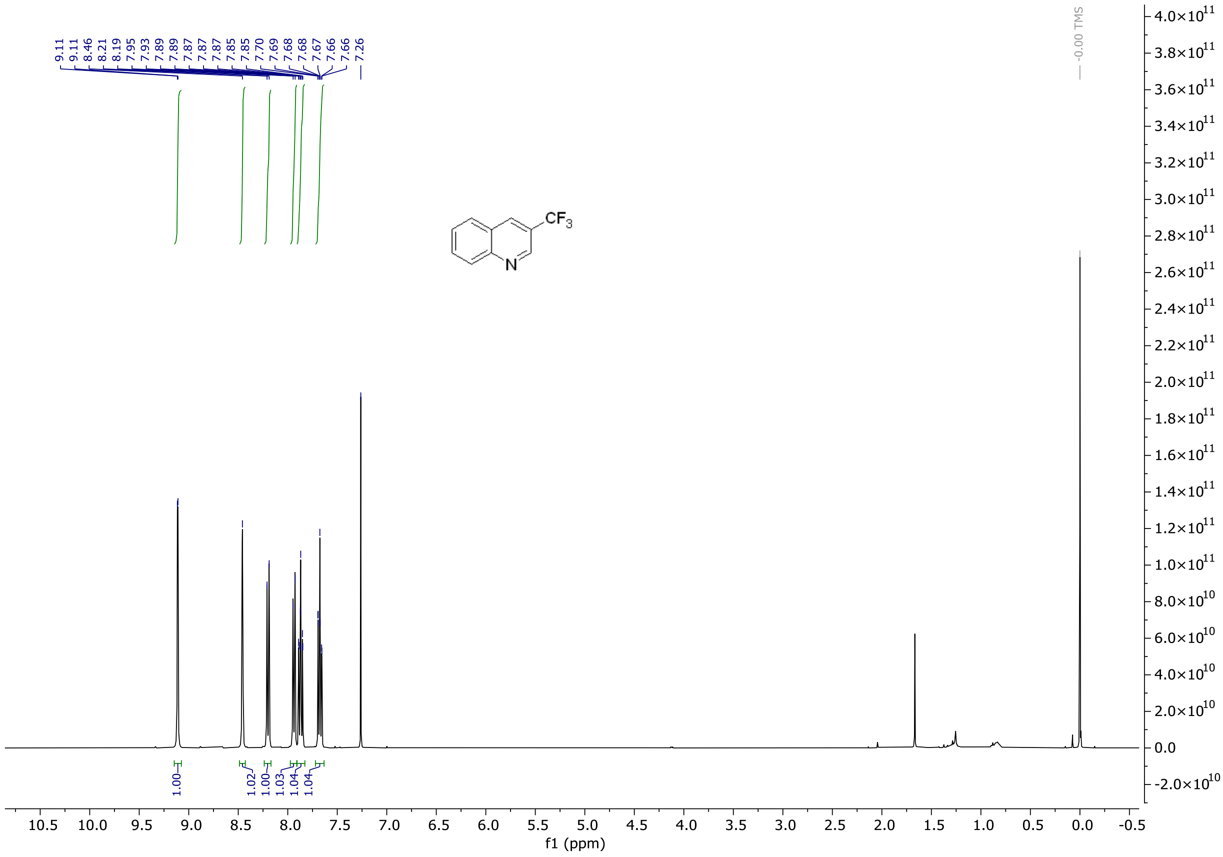

^{13}C NMR (101 MHz, $CDCl_3$) of **4c**:

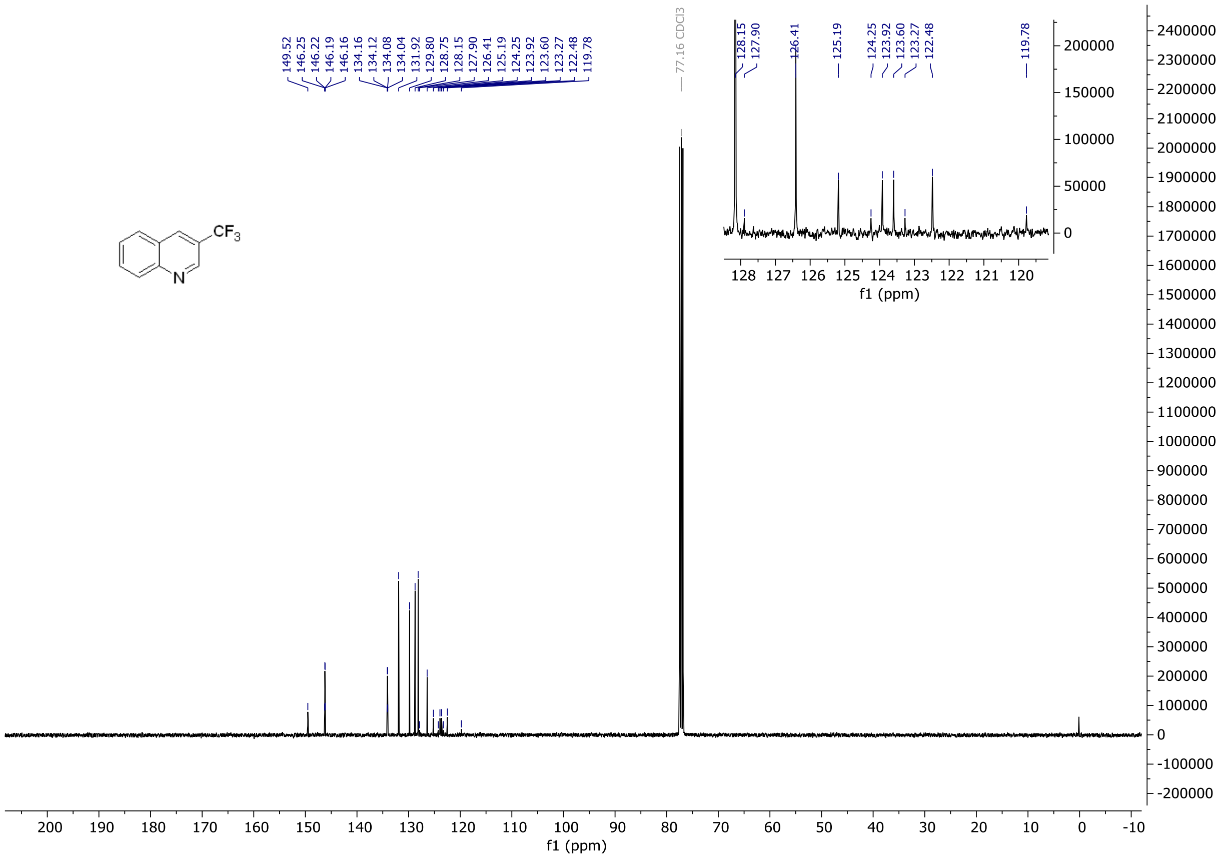

^1H NMR (400 MHz, $CDCl_3$) of **4d**:

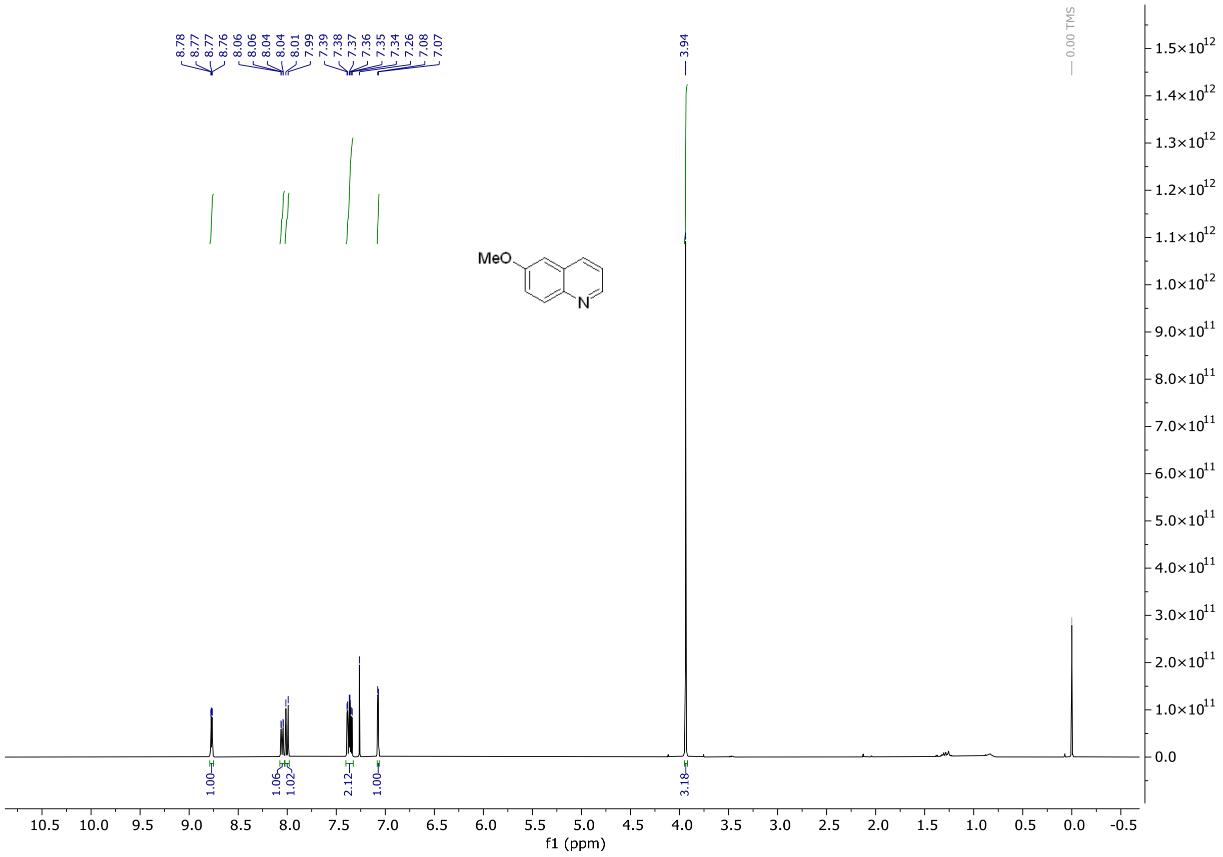

^{13}C NMR (101 MHz, $CDCl_3$) of **4d**:

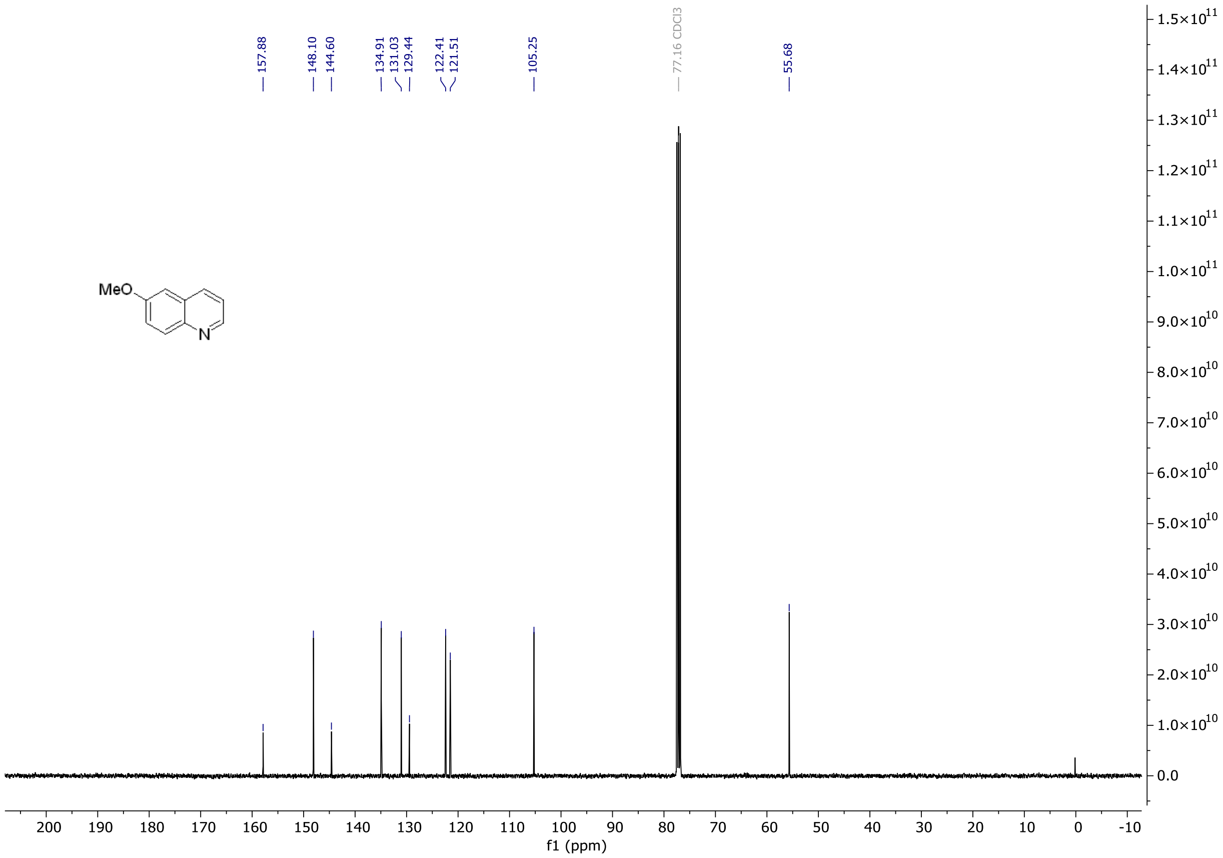

^{1}H NMR (400 MHz, $CDCl_3$) of **4e**:

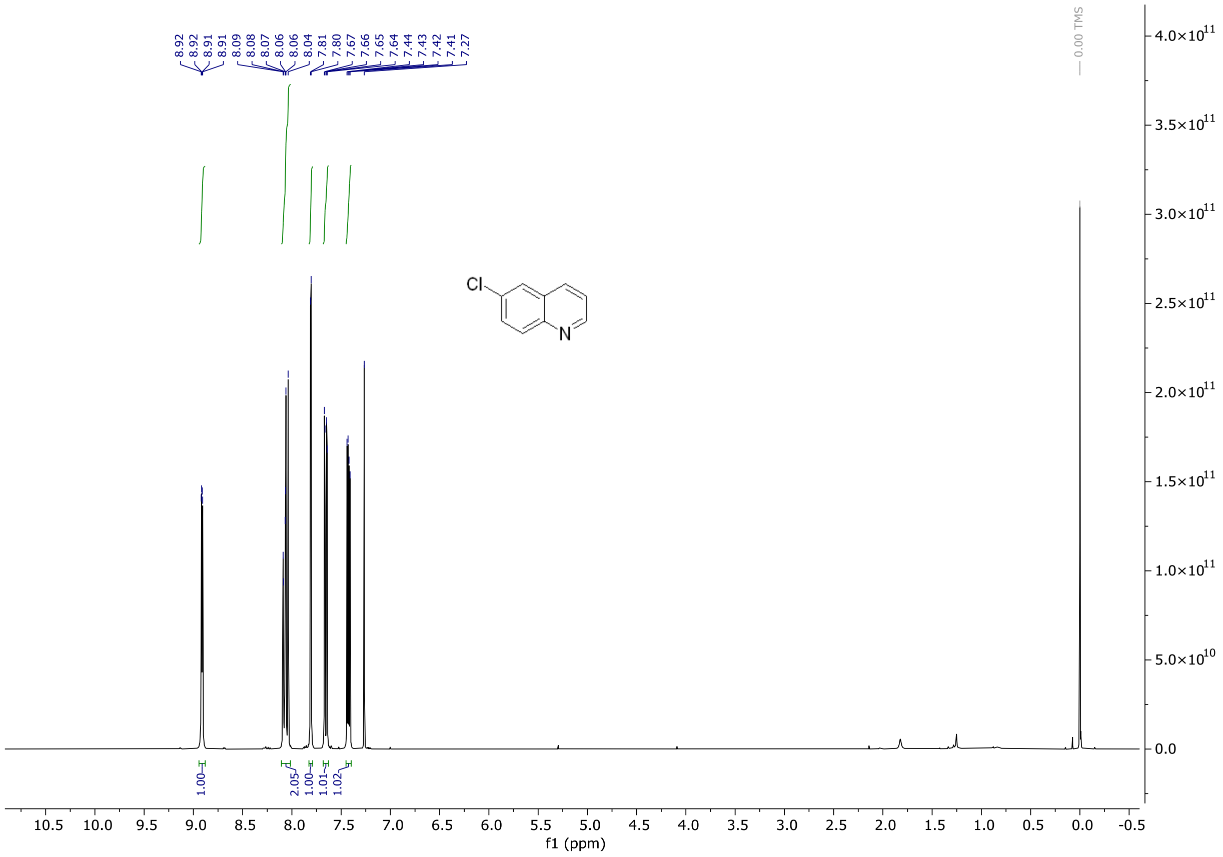

^{13}C NMR (101 MHz, $CDCl_3$) of **4e**:

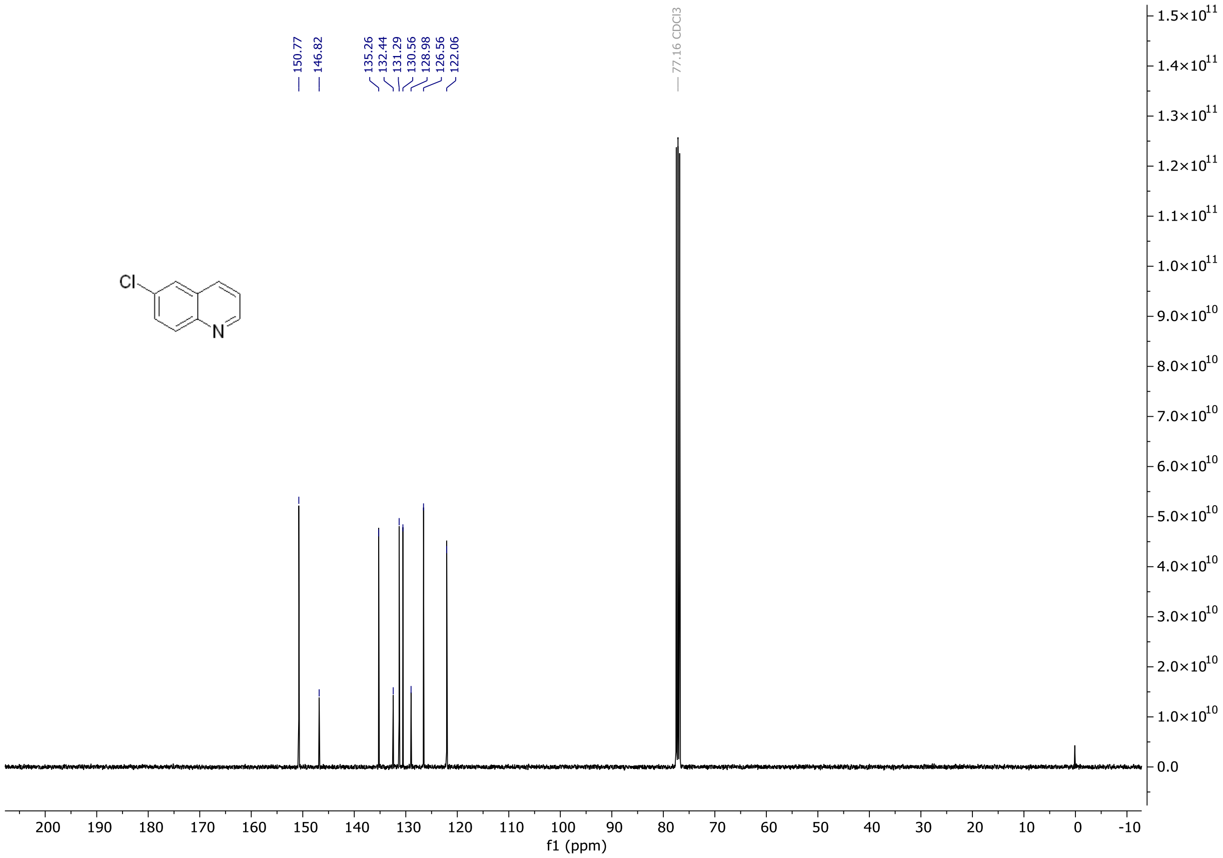

^1H NMR (400 MHz, $CDCl_3$) of **4f**:

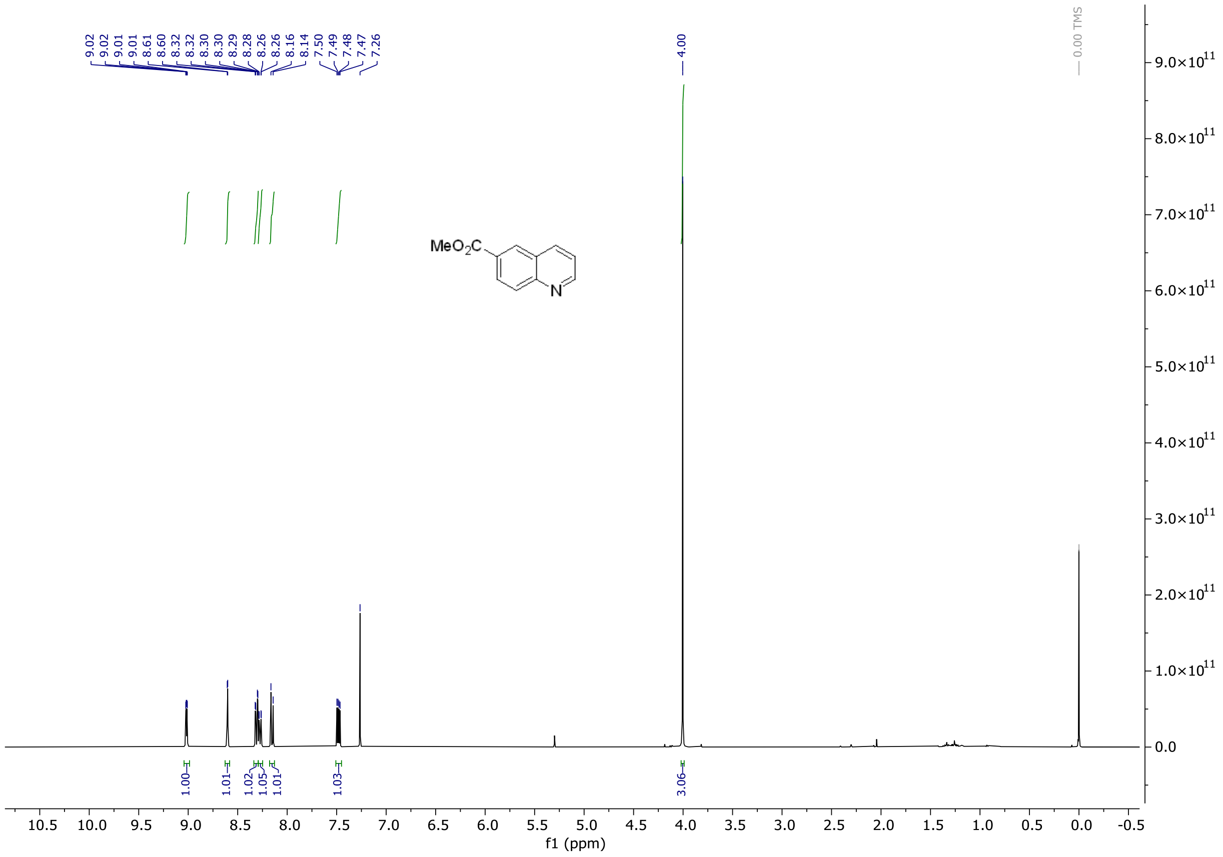

^{13}C NMR (101 MHz, $CDCl_3$) of **4f**:

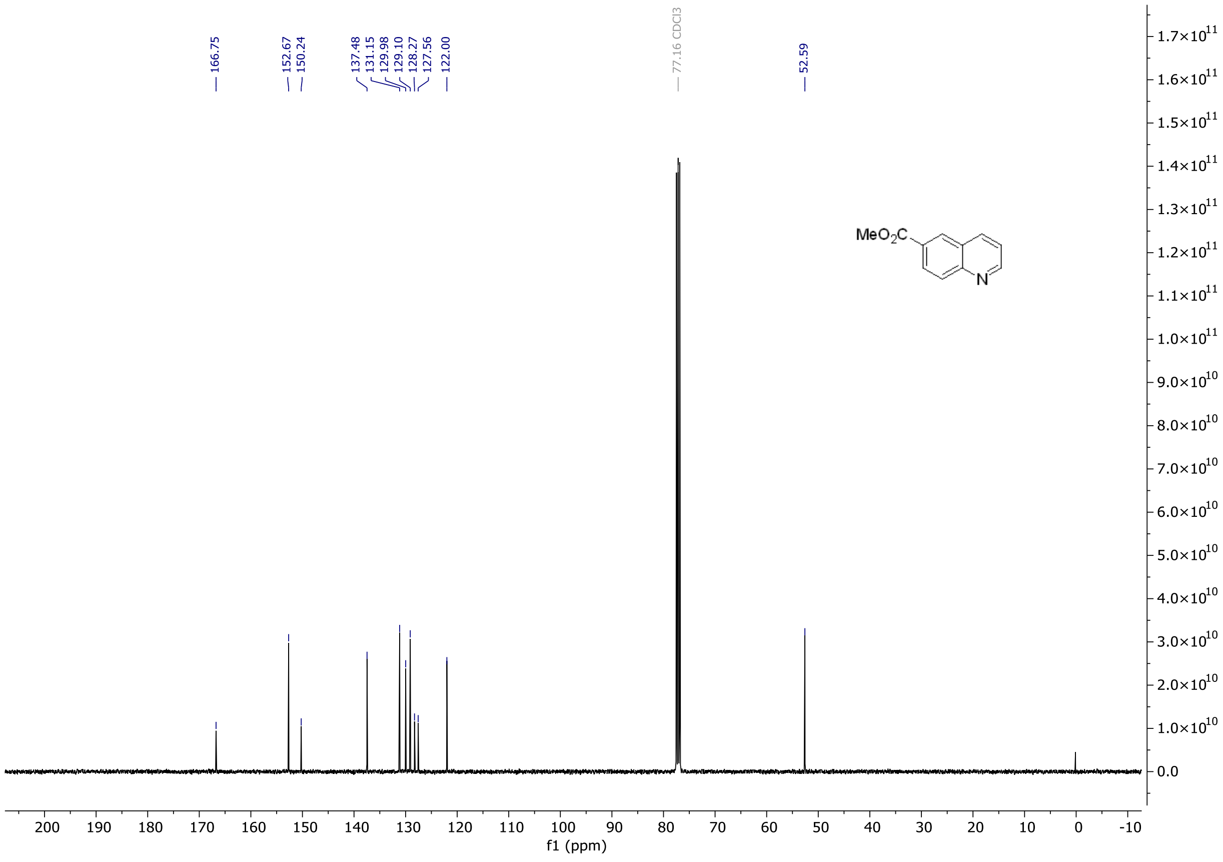

^{1}H NMR (400 MHz, $CDCl_3$) of **4g**:

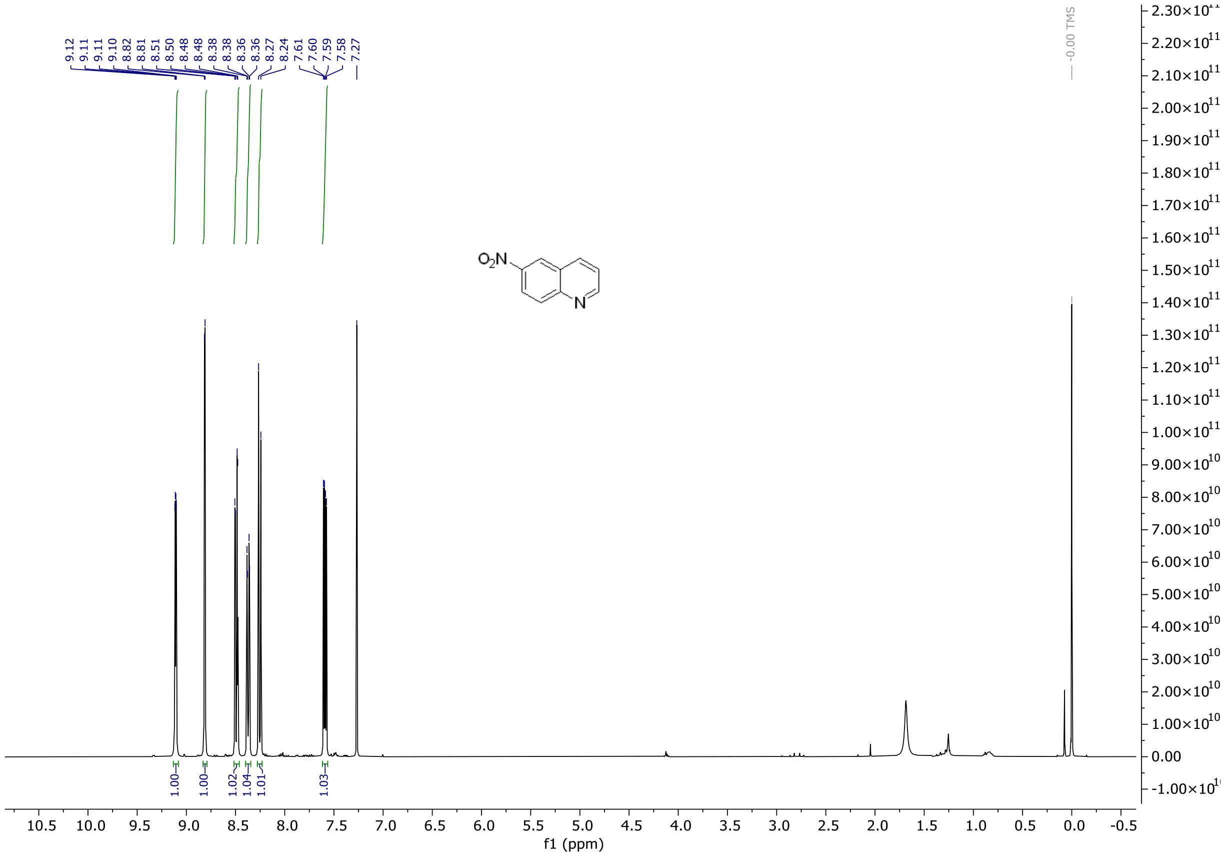

^{13}C NMR (101 MHz, $CDCl_3$) of **4g**:

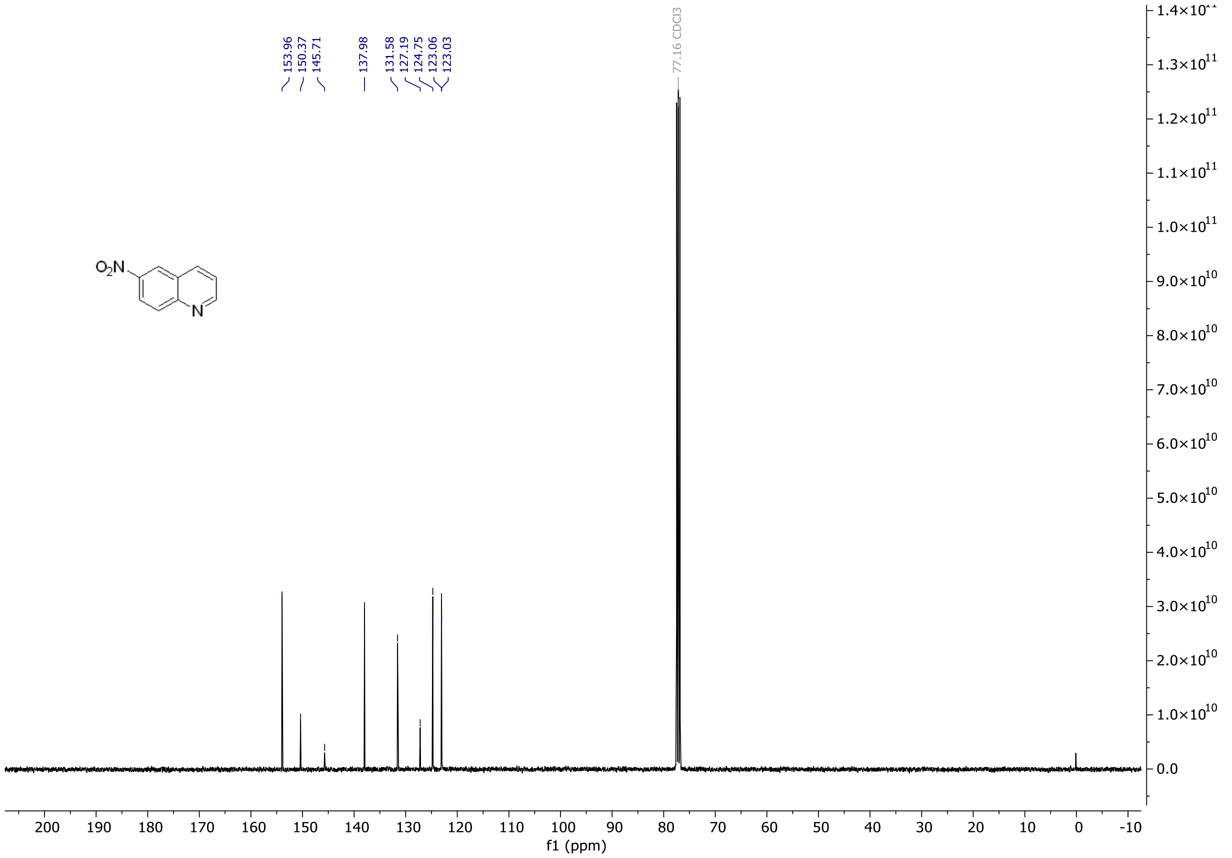

^{1}H NMR (400 MHz, $CDCl_3$) of **4h**:

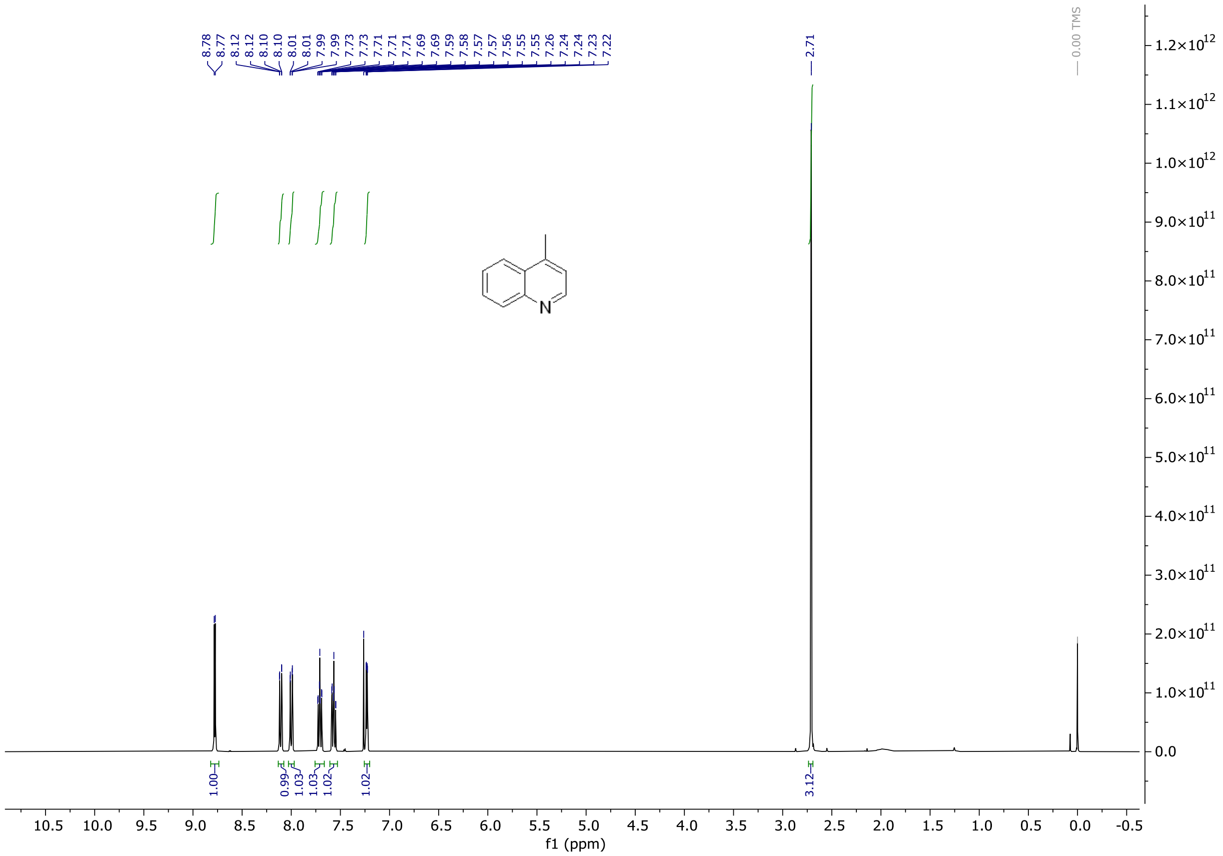

^{13}C NMR (101 MHz, $CDCl_3$) of **4h**:

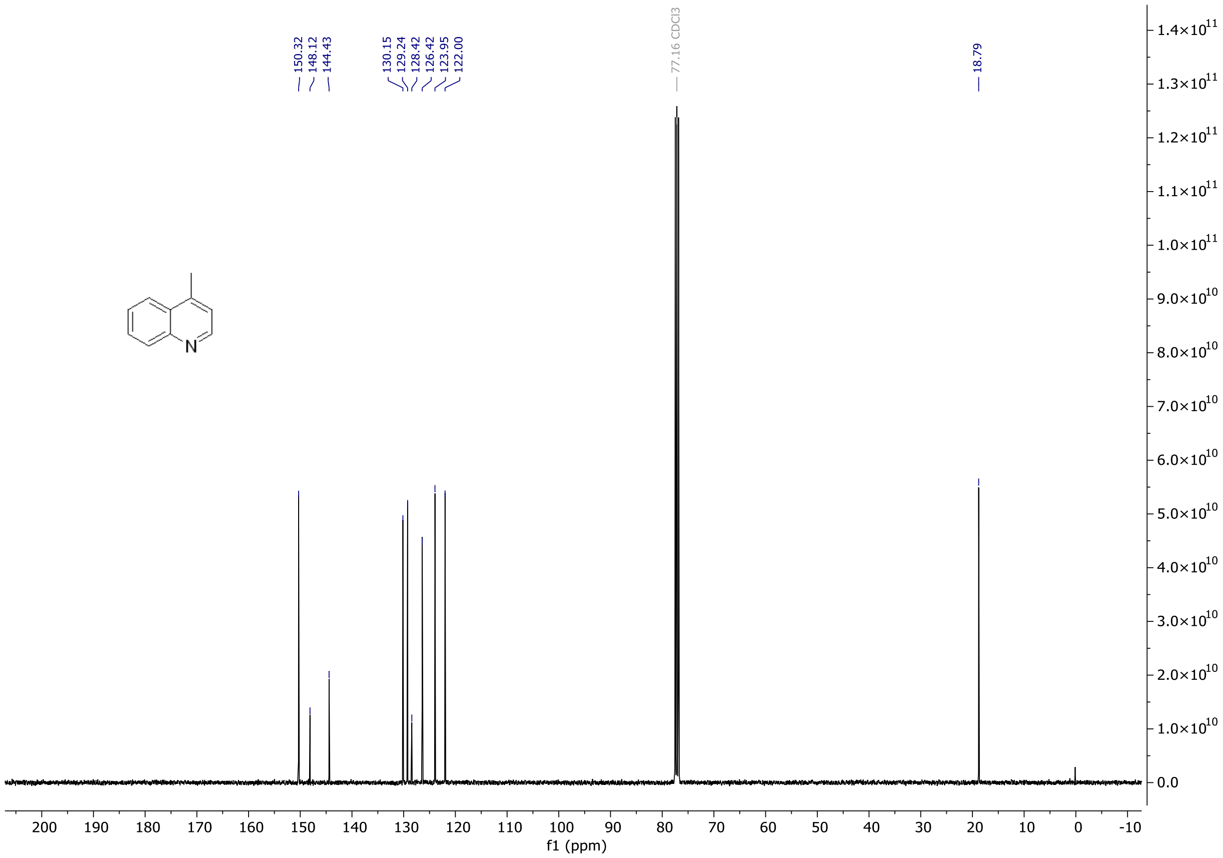

^{1}H NMR (400 MHz, $CDCl_3$) of **4i**:

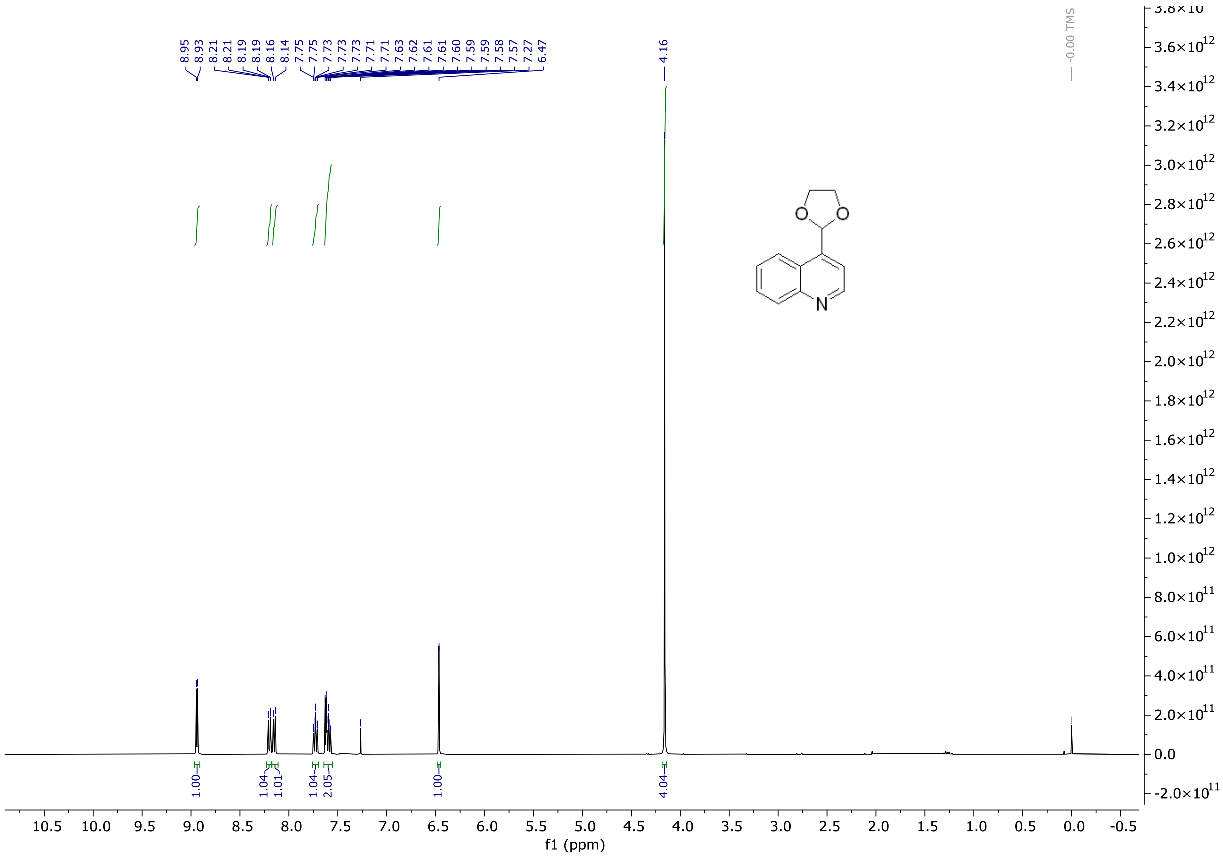

^{13}C NMR (101 MHz, $CDCl_3$) of **4i**:

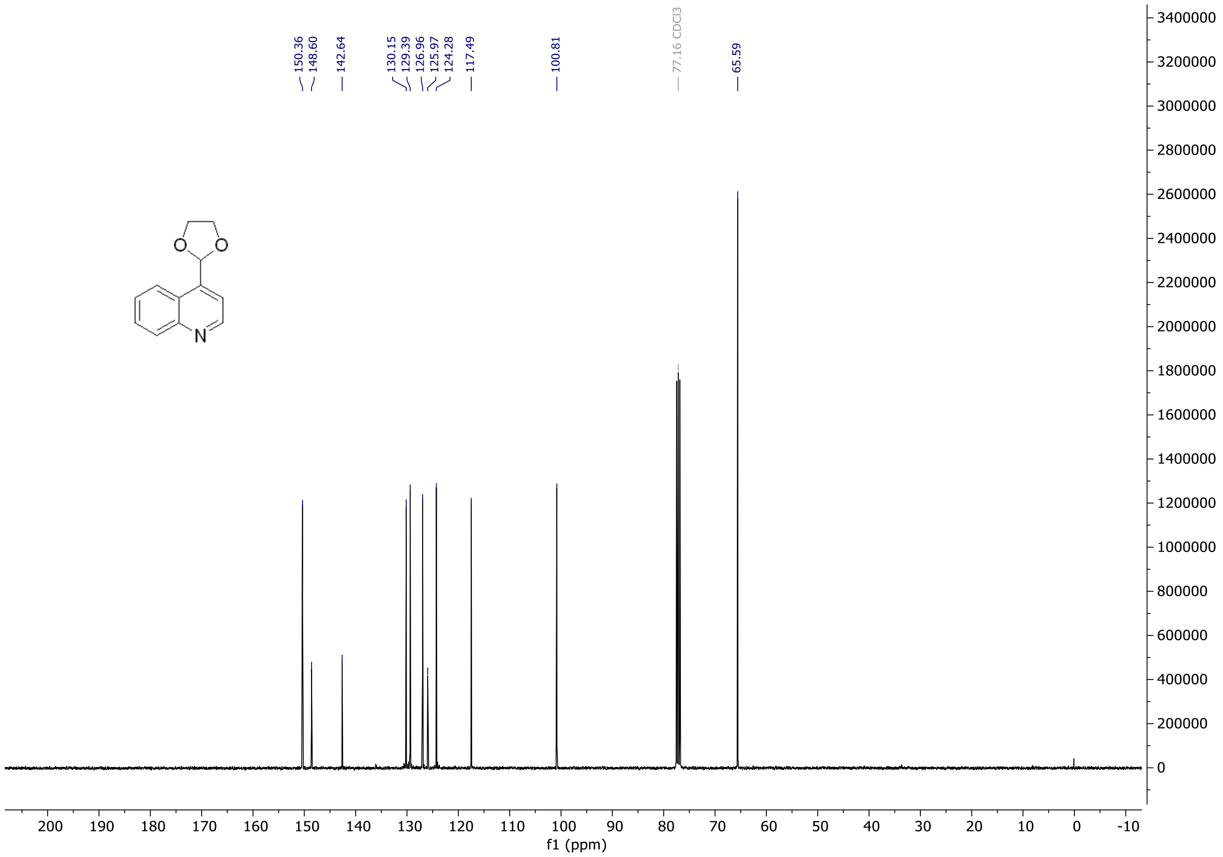

^{1}H NMR (400 MHz, $CDCl_3$) of **4j**:

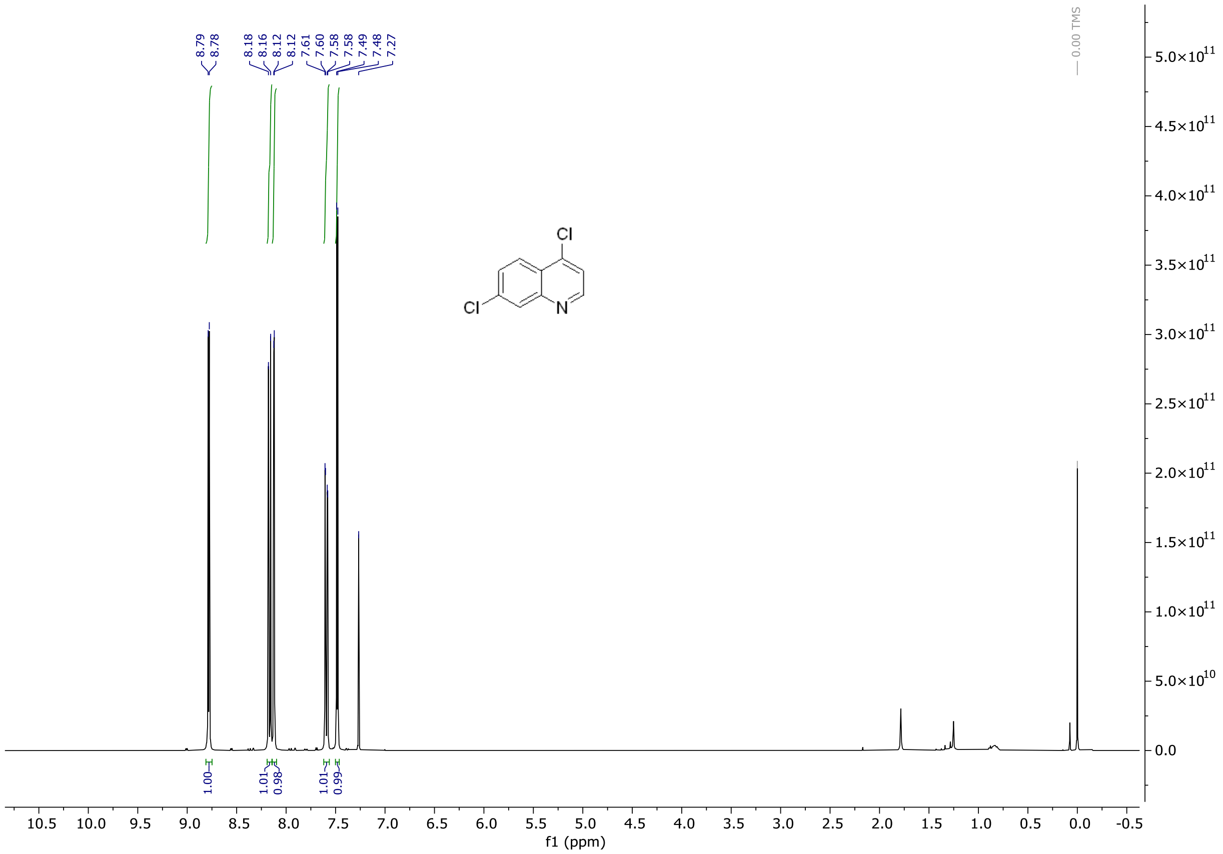

^{13}C NMR (101 MHz, $CDCl_3$) of **4j**:

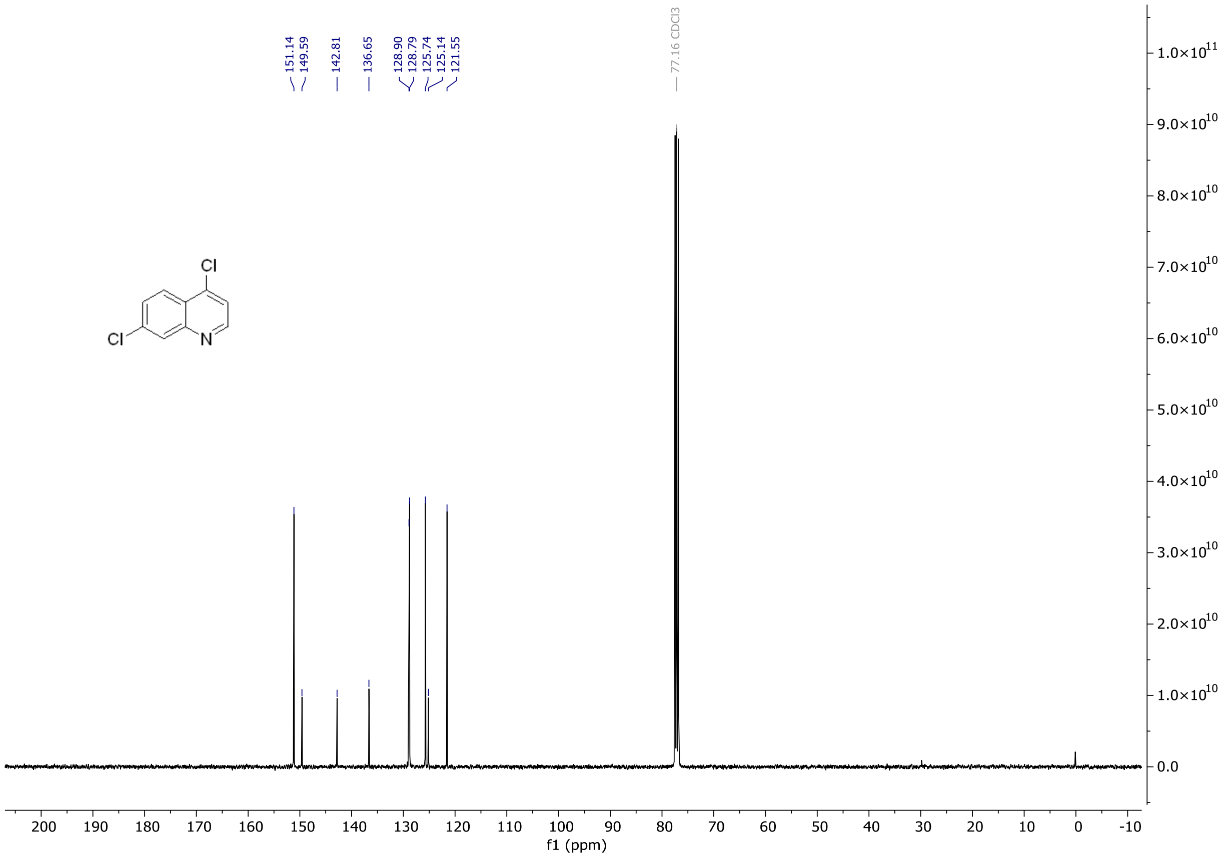

^{1}H NMR (400 MHz, $CDCl_3$) of **4k**:

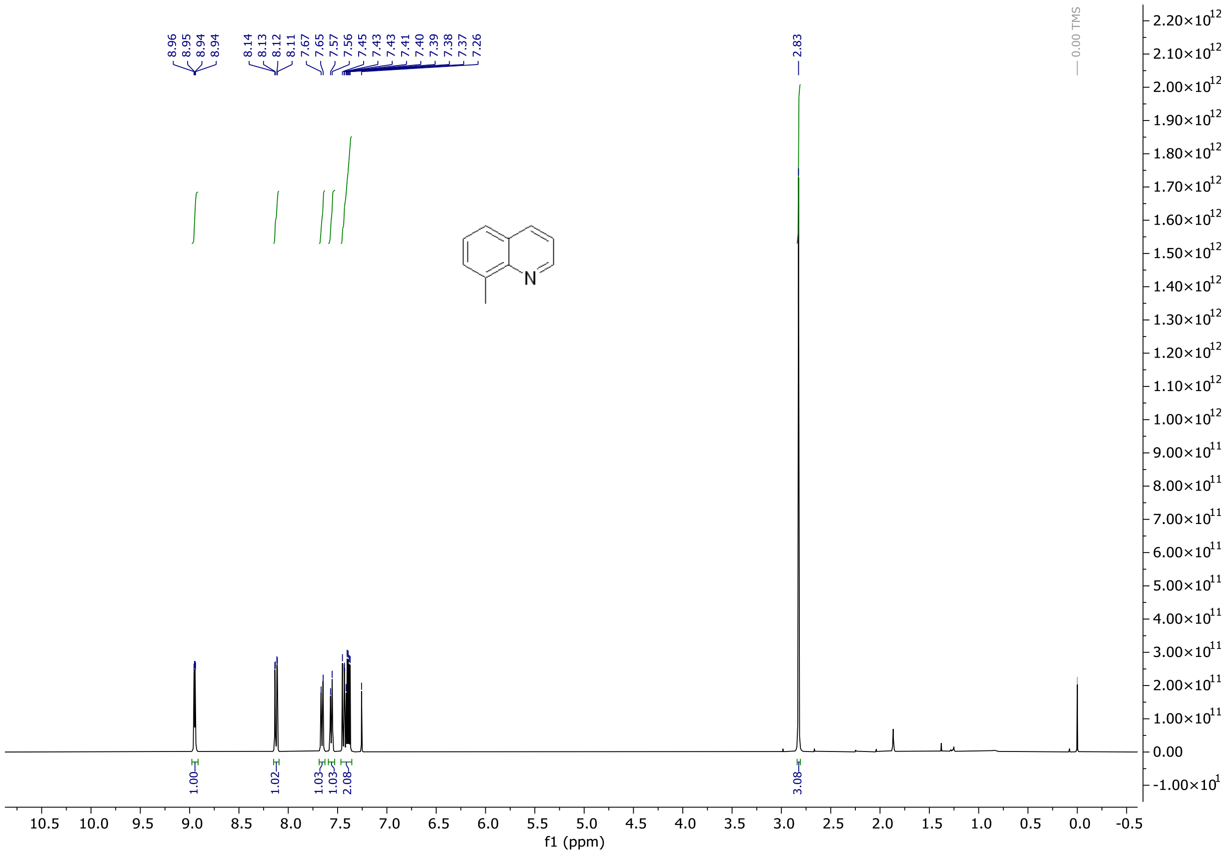

^{13}C NMR (101 MHz, $CDCl_3$) of **4k**:

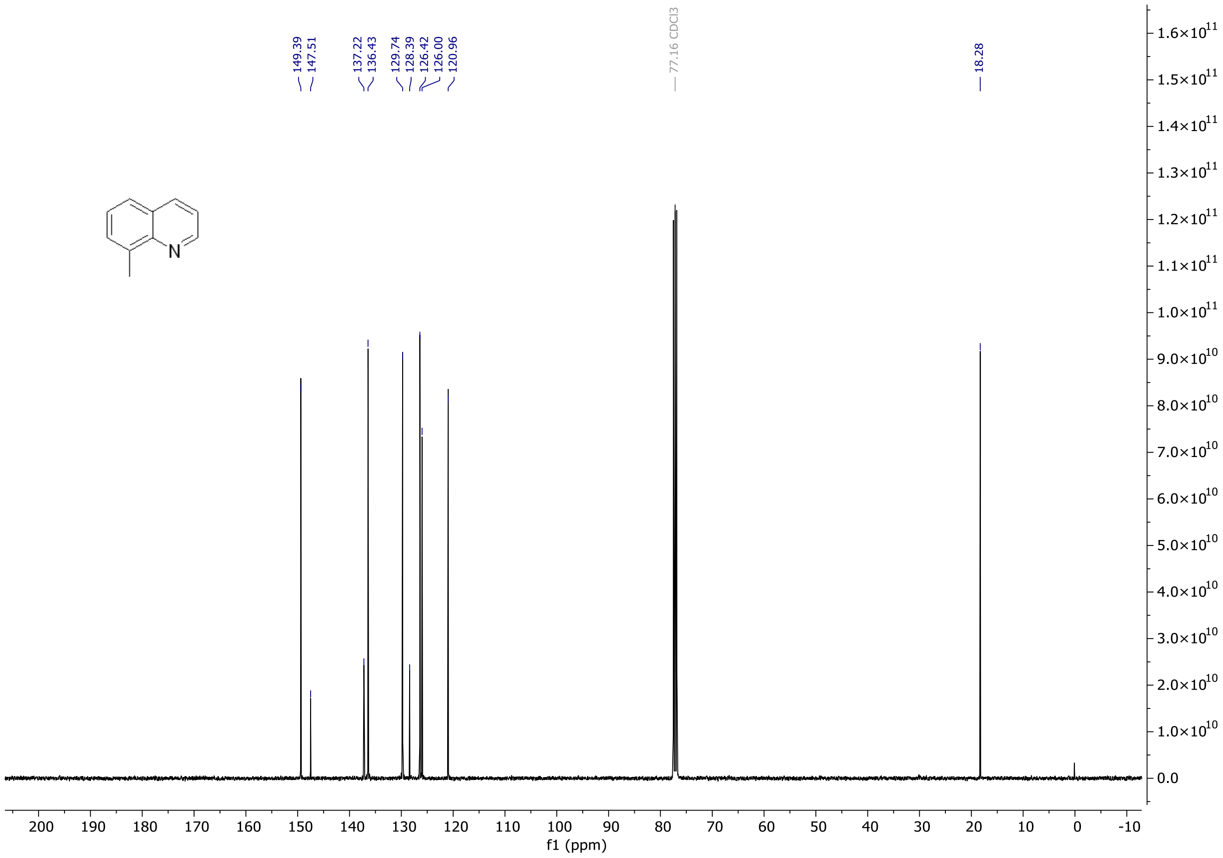

^{1}H NMR (400 MHz, $CDCl_3$) of **4l**:

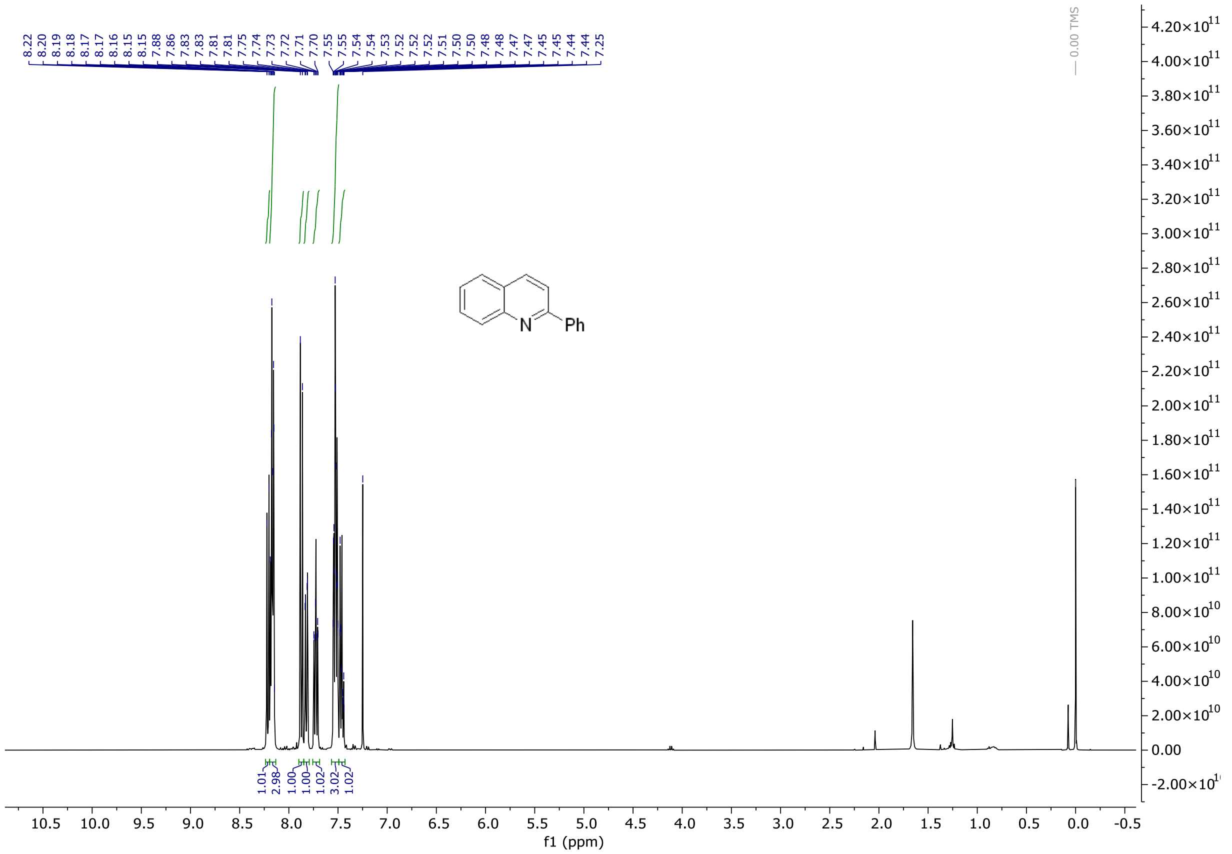

^{13}C NMR (101 MHz, $CDCl_3$) of **4l**:

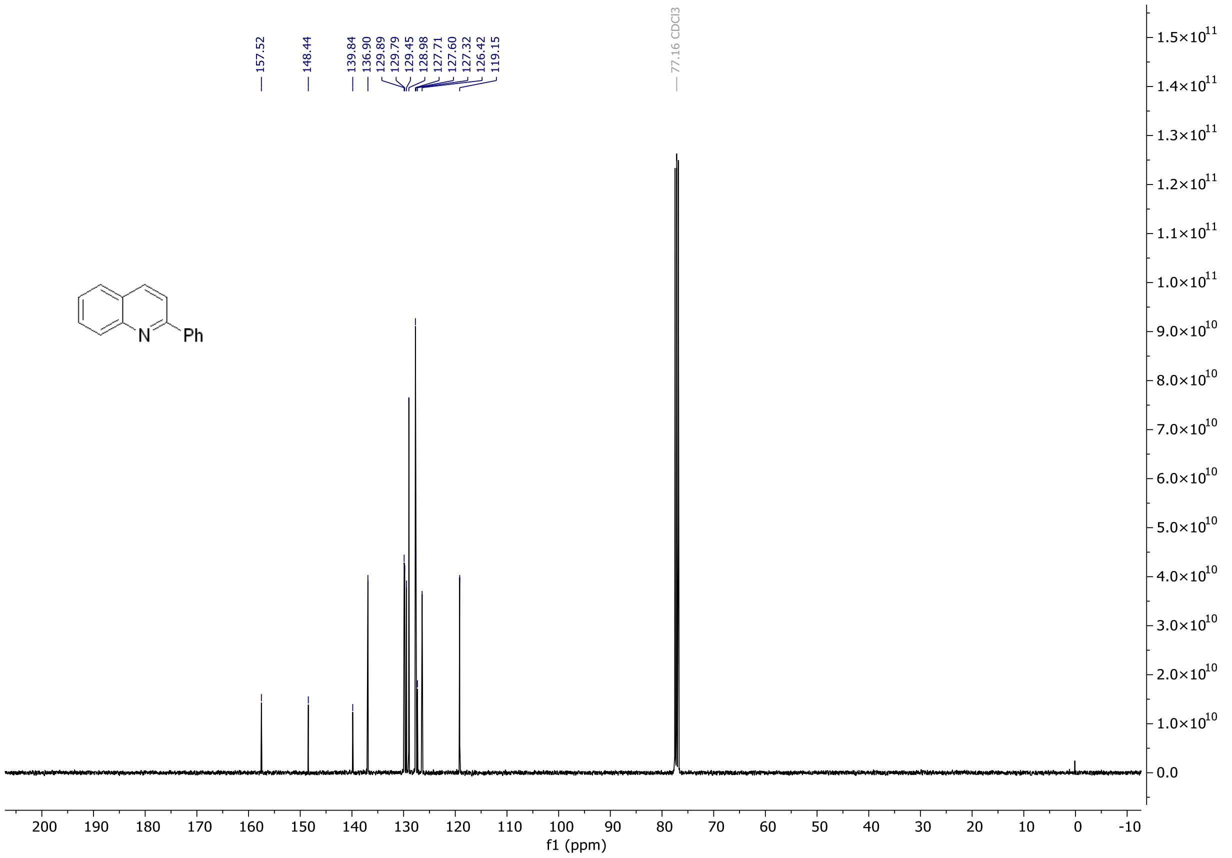

^{1}H NMR (400 MHz, $CDCl_3$) of **4m**:

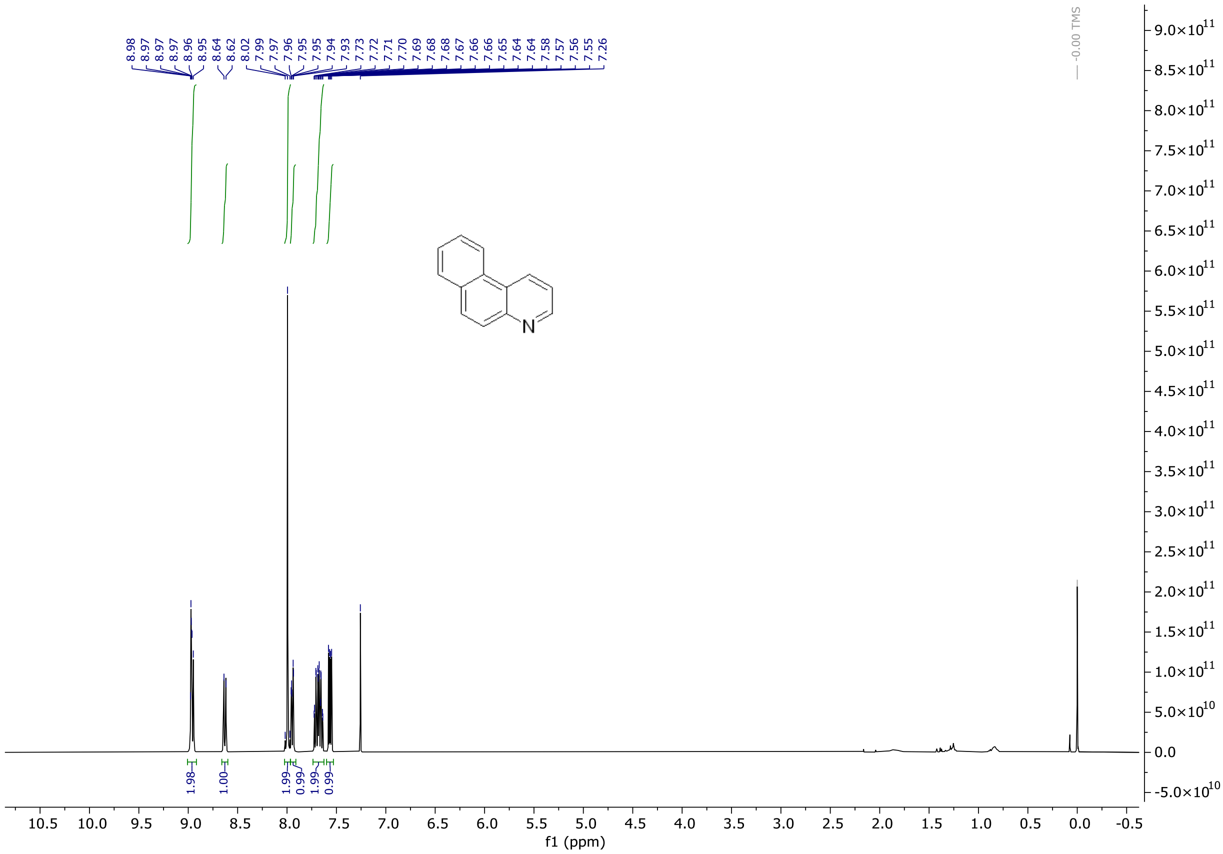

^{13}C NMR (101 MHz, $CDCl_3$) of **4m**:

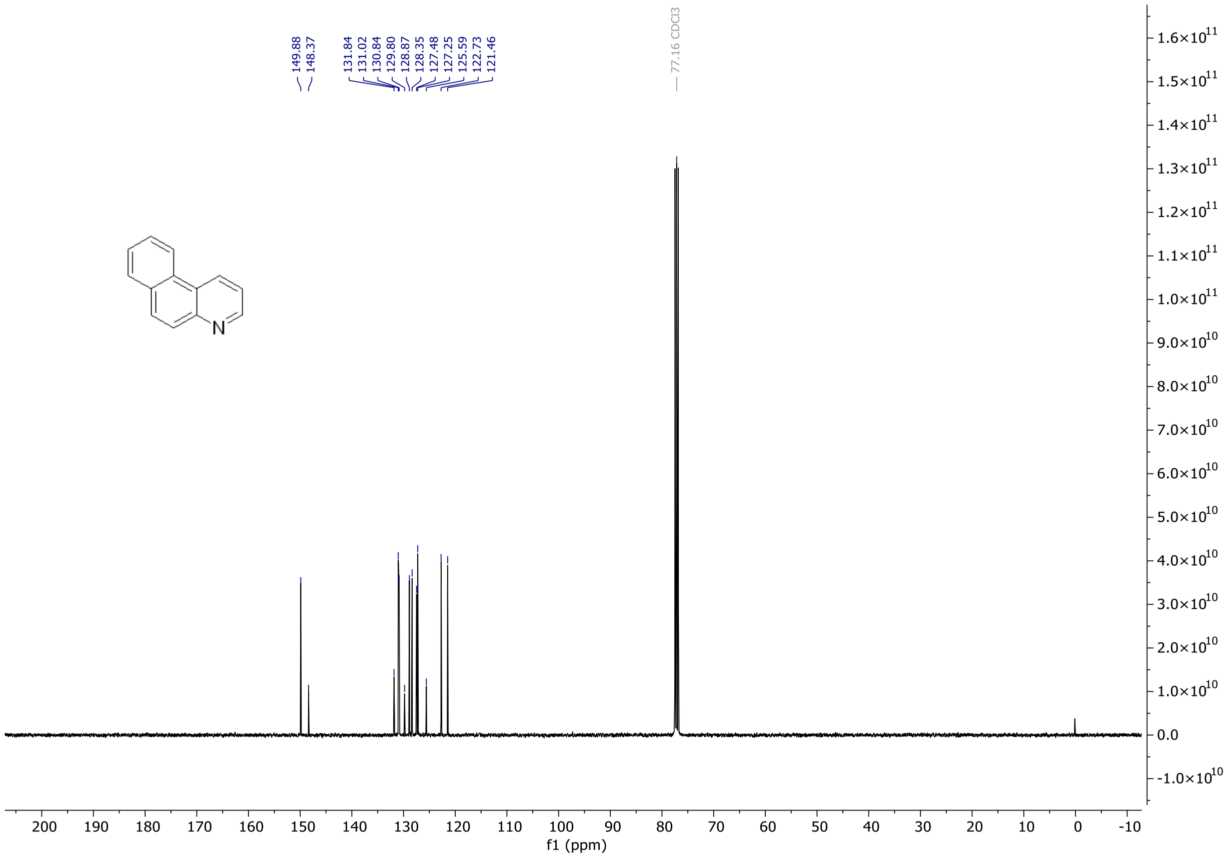

^1H NMR (400 MHz, $CDCl_3$) of **4n**:

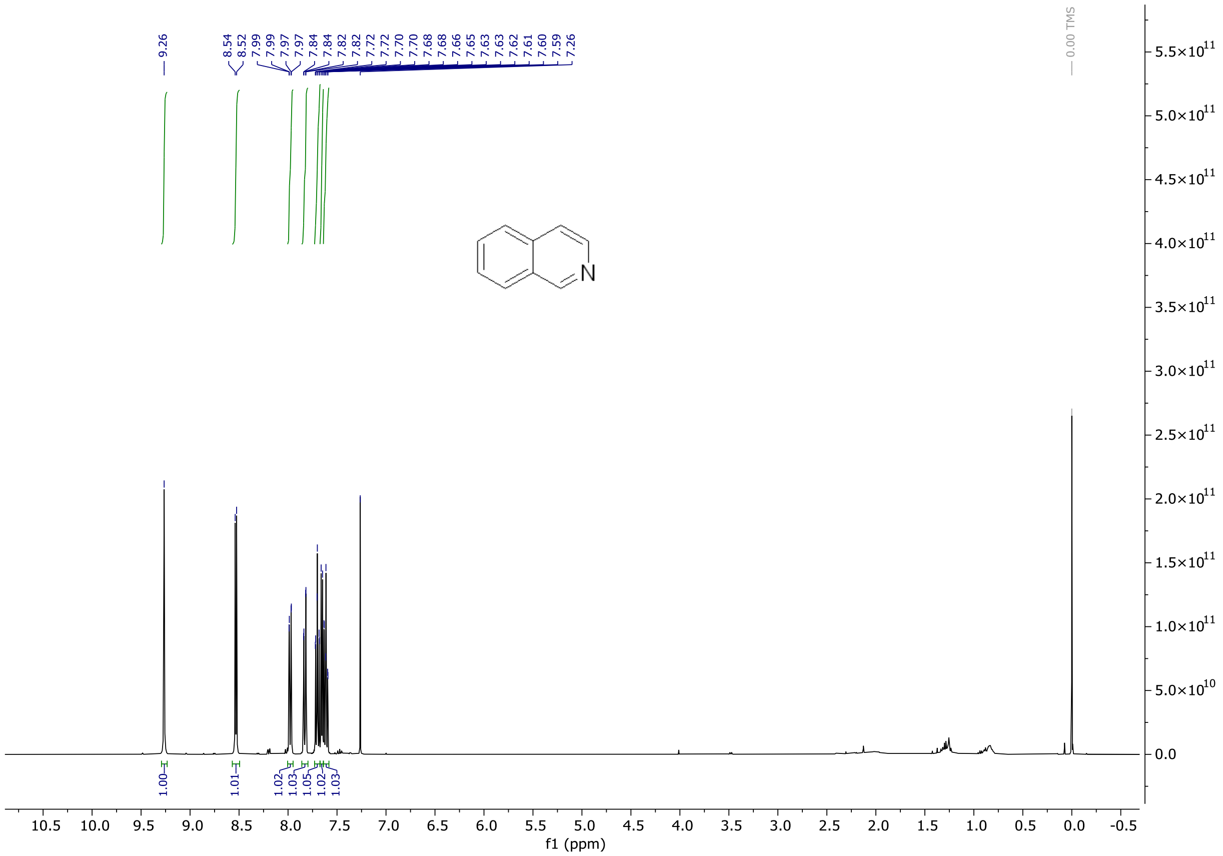

^{13}C NMR (101 MHz, $CDCl_3$) of **4n**:

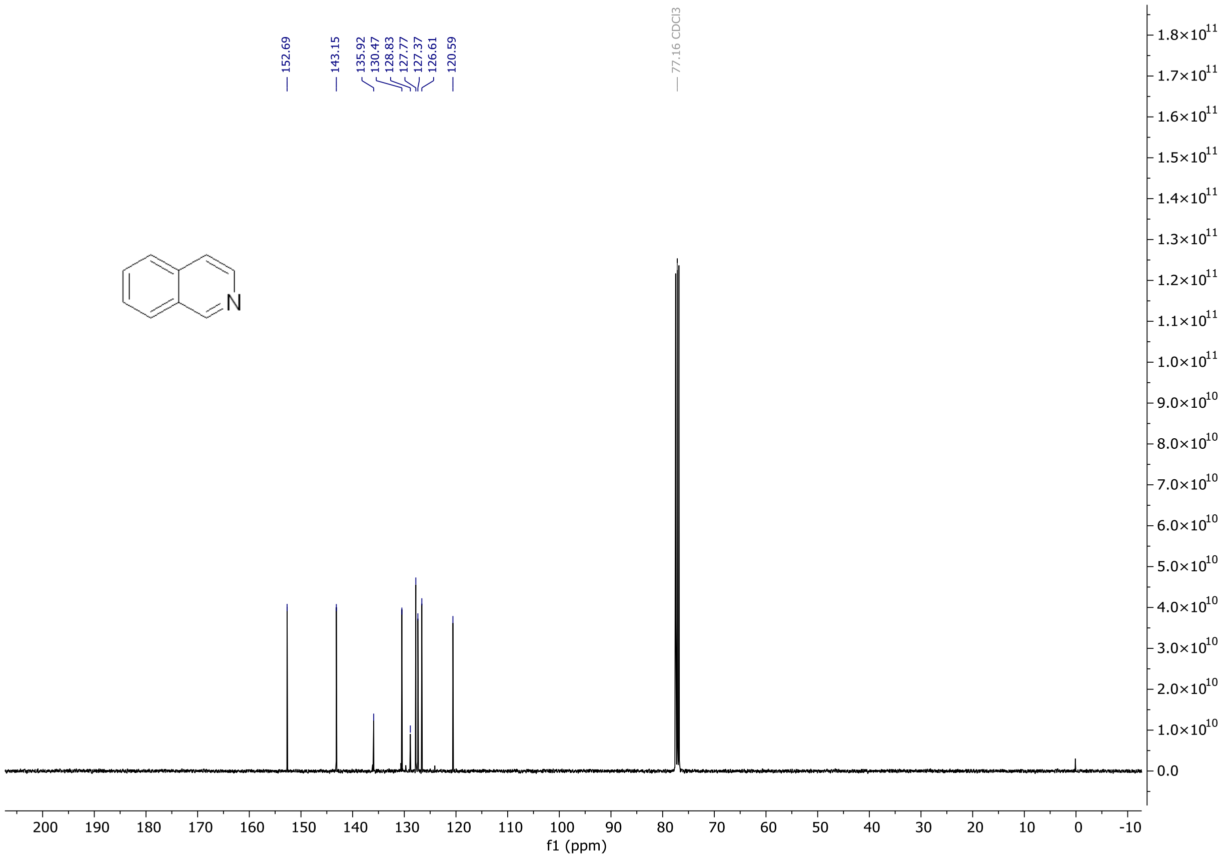

^{1}H NMR (400 MHz, $CDCl_3$) of **4o**:

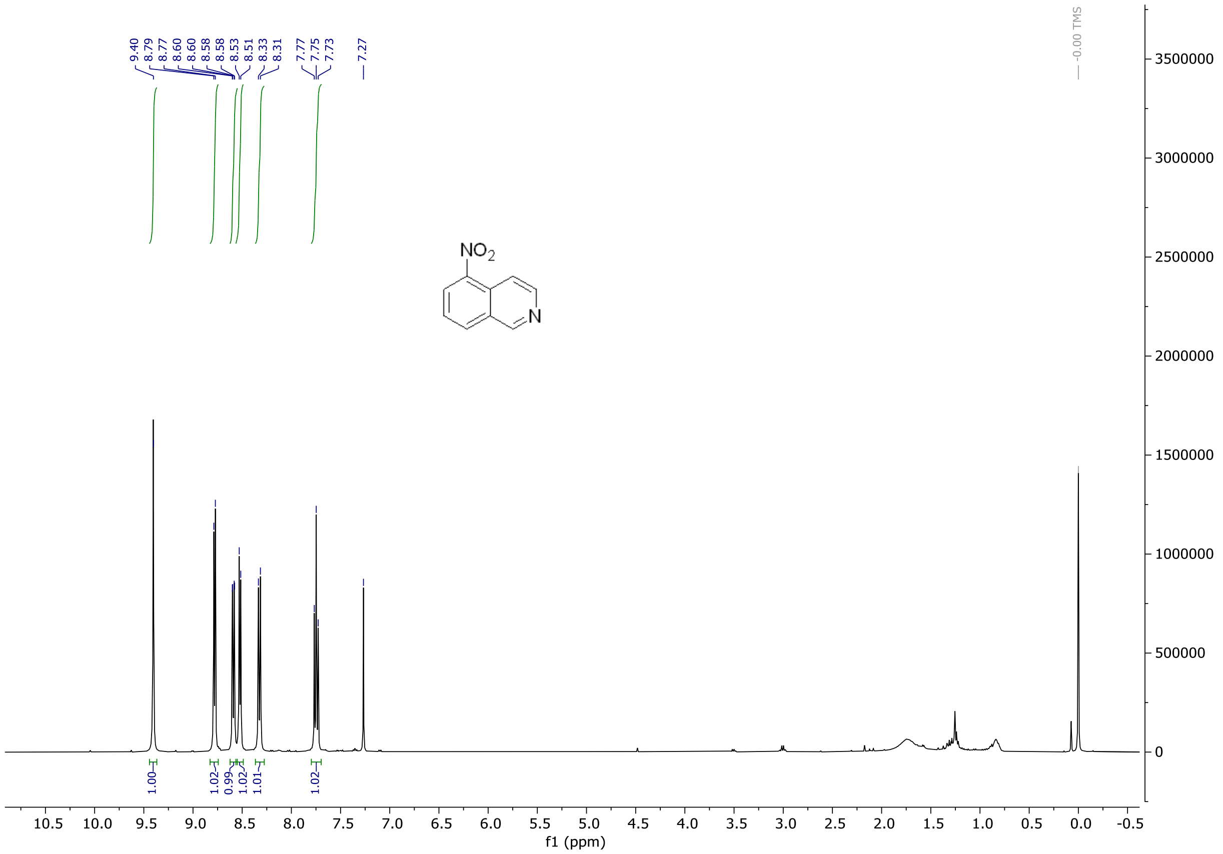

^{13}C NMR (101 MHz, $CDCl_3$) of **4o**:

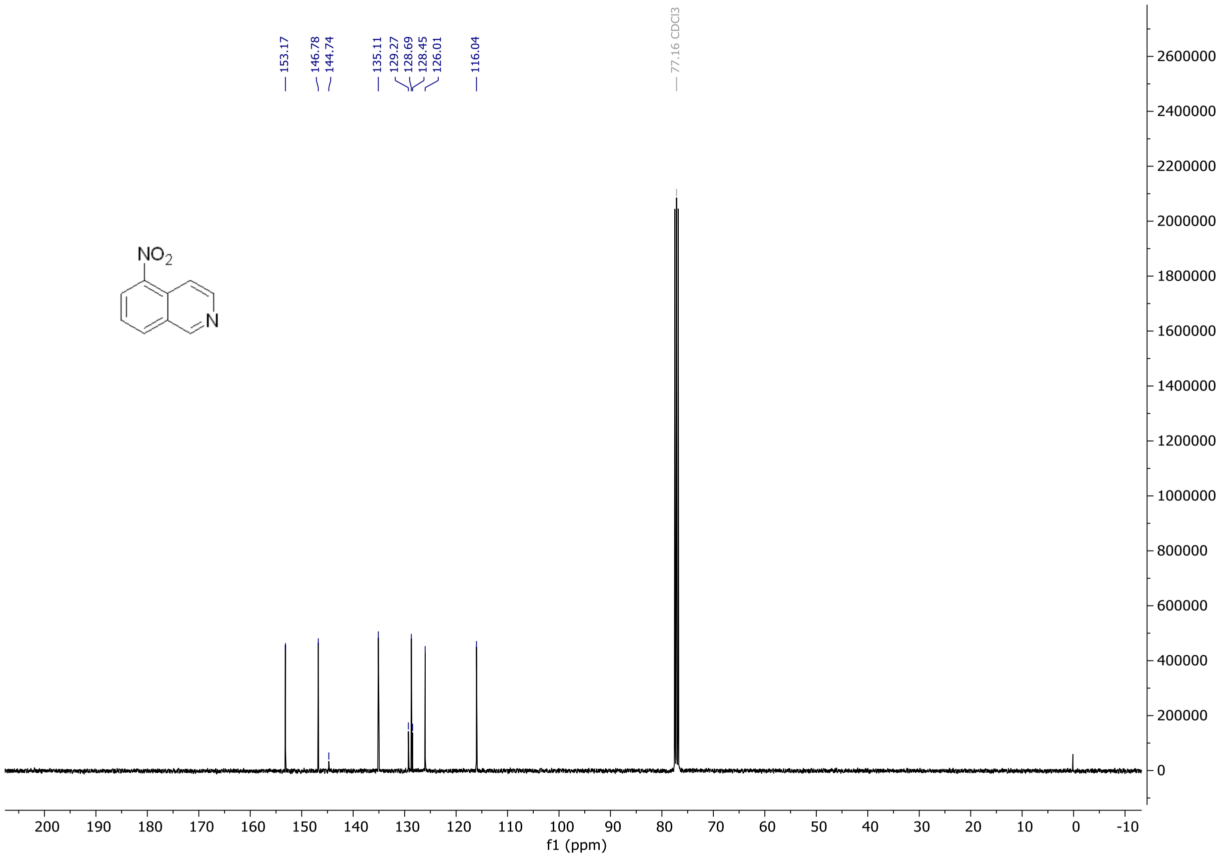

^{1}H NMR (400 MHz, $CDCl_3$) of **4p**:

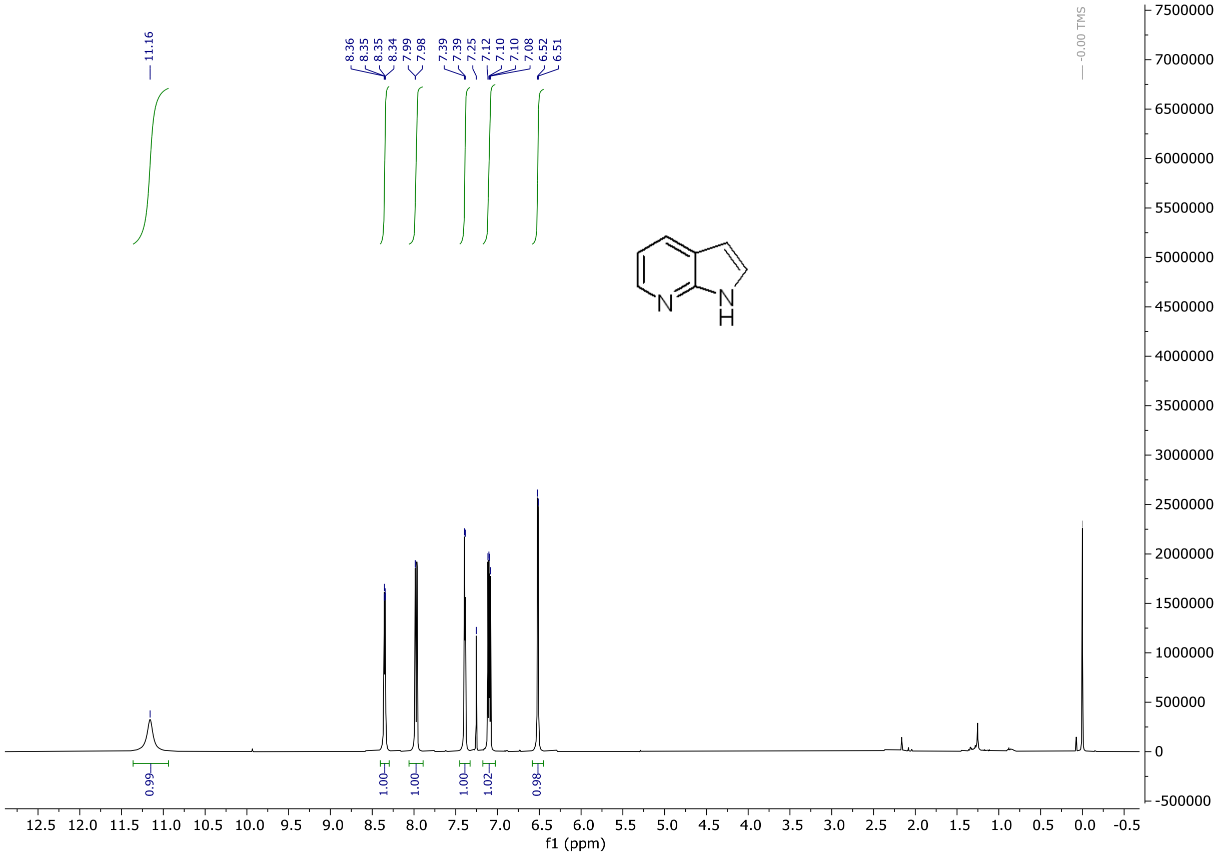

^{13}C NMR (101 MHz, $CDCl_3$) of **4p**:

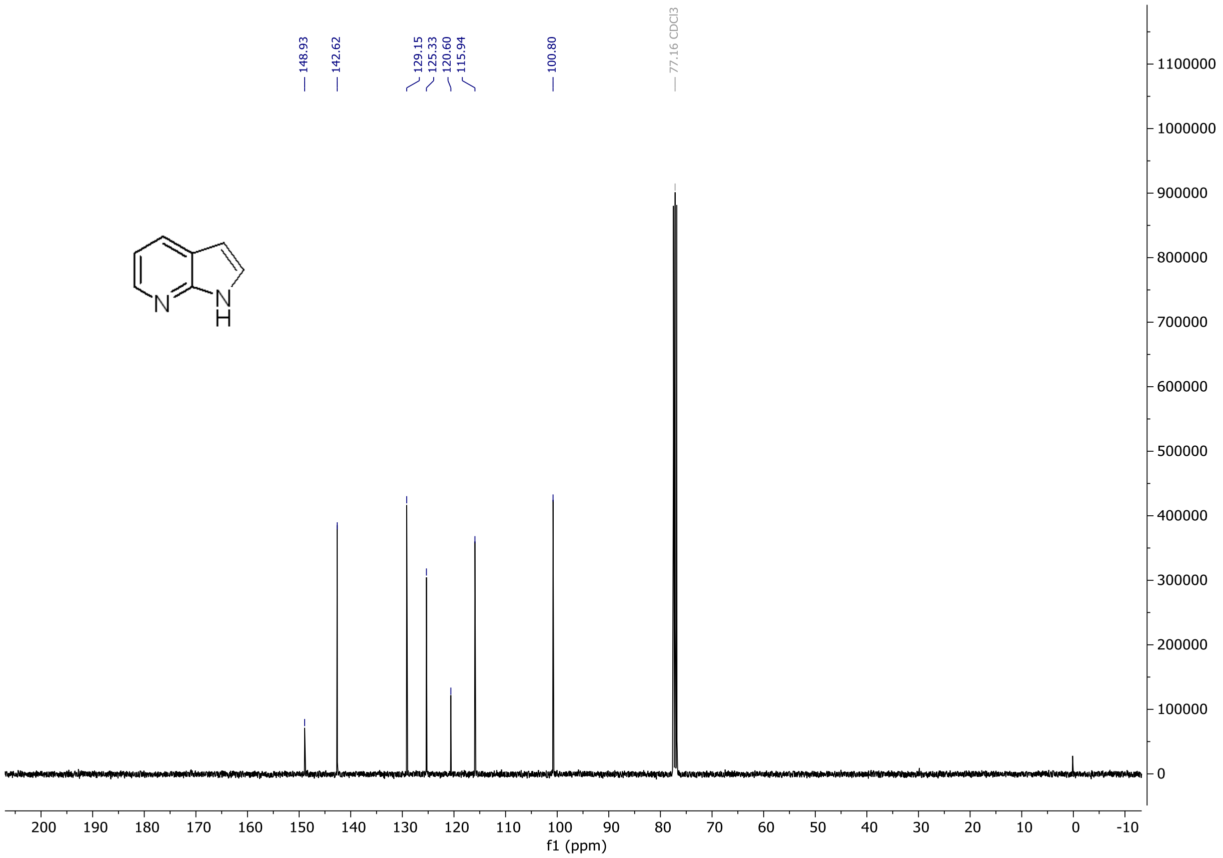

^{1}H NMR (400 MHz, $CDCl_3$) of **4q**:

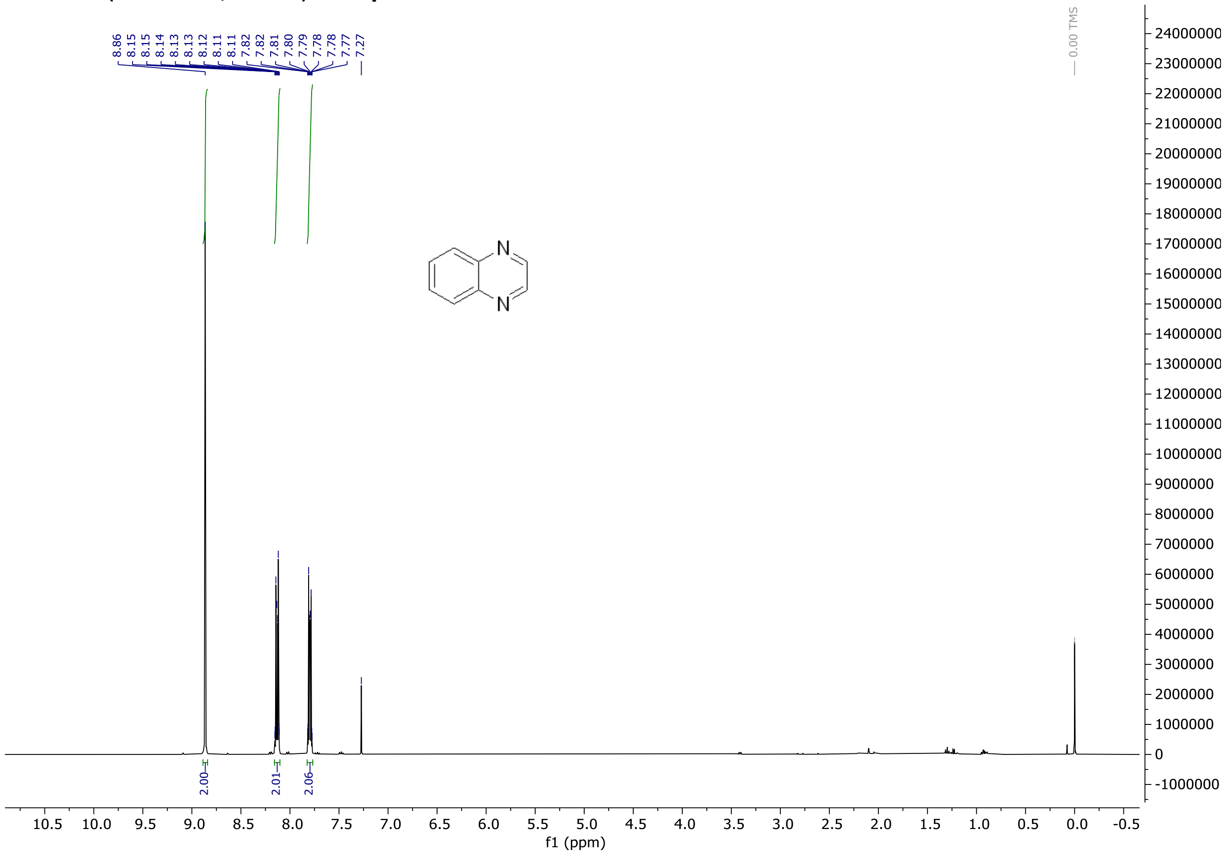

^{13}C NMR (101 MHz, $CDCl_3$) of **4q**:

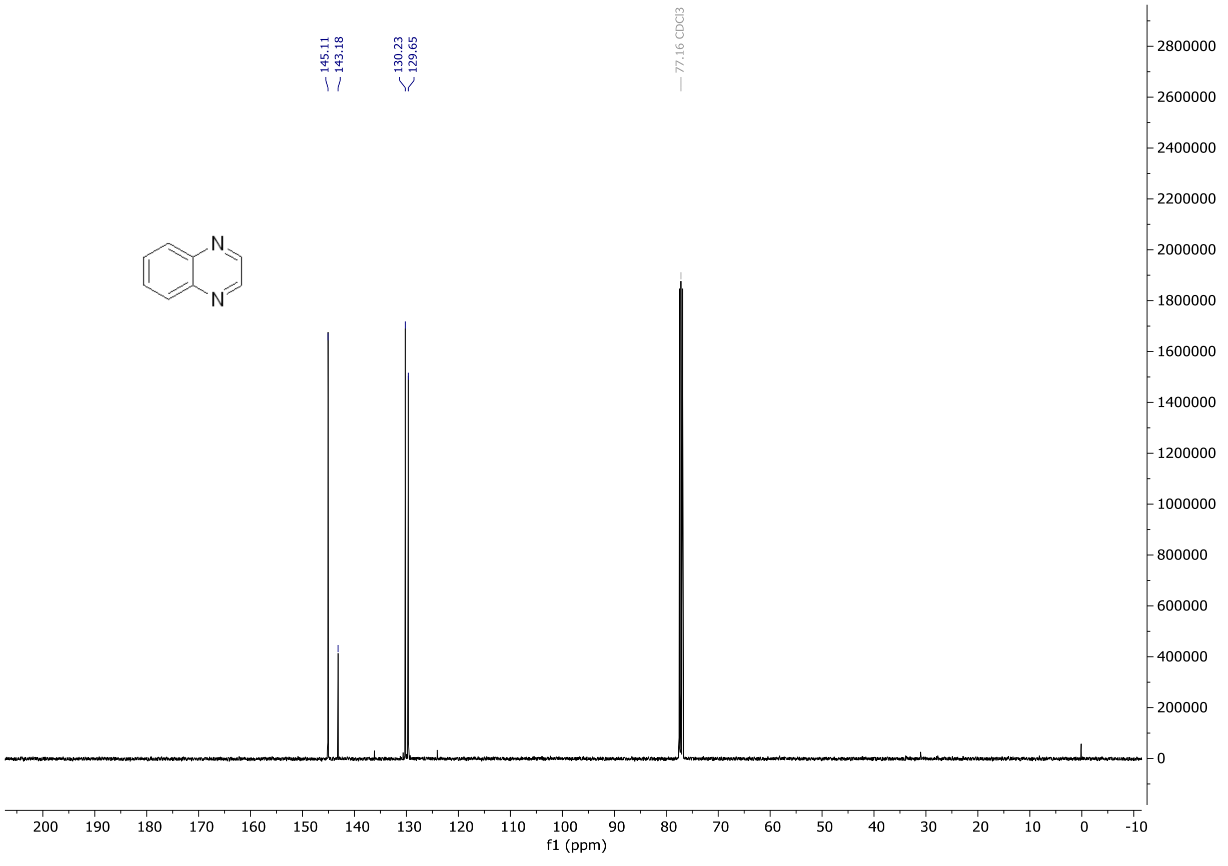

References

(1) Talvitie, J.; Alanko, I.; Lenarda, A.; Durandin, N.; Tkachenko, N.; Nieger, M.; Helaja, J. Electron-Deficient Phenanthrenequinone Derivative for Photoactivated Hydrogen Atom Transfer Mediated Oxidation of Secondary Alcohols. *ChemPhotoChem* **2023**, *7* (10), e202300107. https://doi.org/10.1002/cptc.202300107.

(2) Xia, Q.; Zhao, X.; Zhang, J.; Wang, J.; Song, G. A Novel and Efficient Method for the Direct Synthesis of Pyrrolyl or Indolyl Substituted 9,10-Dihydrophenanthren-9-Ol Analogues. *Tetrahedron Lett.* **2020**, *61* (7), 151500. https://doi.org/10.1016/j.tetlet.2019.151500.

(3) Li, G.; Jia, C.; Sun, K. Copper-Catalyzed Intermolecular Dehydrogenative Amidation/Amination of Quinoline *N*-Oxides with Lactams/Cyclamines. *Org. Lett.* **2013**, *15* (20), 5198–5201. https://doi.org/10.1021/ol402324v.

(4) Kim, K. D.; Lee, J. H. Visible-Light Photocatalyzed Deoxygenation of N-Heterocyclic *N*-Oxides. *Org. Lett.* **2018**, *20* (23), 7712–7716. https://doi.org/10.1021/acs.orglett.8b03446.

(5) Li, D.; Liang, C.; Jiang, Z.; Zhang, J.; Zhuo, W.-T.; Zou, F.-Y.; Wang, W.-P.; Gao, G.-L.; Song, J. Visible-Light-Promoted C2 Selective Arylation of Quinoline and Pyridine *N*-Oxides with Diaryliodonium Tetrafluoroborate. *J. Org. Chem.* **2020**, *85* (4), 2733–2742. https://doi.org/10.1021/acs.joc.9b02933.

(6) Vegi, S. R.; Boovanahalli, S. K.; Sharma, A. P.; Mukkanti, K. The First Total Synthesis of Novel Human Chymase Inhibitor SPF32629A. *Tetrahedron Lett.* **2008**, *49* (44), 6297–6299. https://doi.org/10.1016/j.tetlet.2008.08.048.

(7) Kaithal, A.; Chatterjee, B.; Gunanathan, C. Ruthenium-Catalyzed Regioselective 1,4-Hydroboration of Pyridines. *Org. Lett.* **2016**, *18* (14), 3402–3405. https://doi.org/10.1021/acs.orglett.6b01564.

(8) Stewart, G. W.; Maligres, P. E.; Baxter, C. A.; Junker, E. M.; Krska, S. W.; Scott, J. P. An Approach to Heterodiarylmethanes via sp^2-sp^3 Suzuki-Miyaura Cross-Coupling. *Tetrahedron* **2016**, *72* (26), 3701–3706. https://doi.org/10.1016/j.tet.2016.02.030.

(9) Campeau, L.-C.; Schipper, D. J.; Fagnou, K. Site-Selective sp^2 and Benzylic sp^3 Palladium-Catalyzed Direct Arylation. *J. Am. Chem. Soc.* **2008**, *130* (11), 3266–3267. https://doi.org/10.1021/ja710451s.

(10) Farrell, R. P.; Elipe, M. V. S.; Bartberger, M. D.; Tedrow, J. S.; Vounatsos, F. An Efficient, Regioselective Amination of 3,5-Disubstituted Pyridine *N*-Oxides Using Saccharin as an Ammonium Surrogate. *Org. Lett.* **2013**, *15* (1), 168–171. https://doi.org/10.1021/ol303218p.

(11) Kozikowski, A. P.; Araldi, G. L.; Ball, R. G. Dipolar Cycloaddition Route to Diverse Analogues of Cocaine: The 6- and 7-Substituted 3-Phenyltropanes. *J. Org. Chem.* **1997**, *62* (3), 503–509. https://doi.org/10.1021/jo961957g.

(12) Radix, S.; Hallé, F.; Mahiout, Z.; Teissonnière, A.; Bouchez, G.; Auberger, L.; Barret, R.; Lomberget, T. A Journey through Hemetsberger–Knittel, Leimgruber–Batcho and Bartoli Reactions: Access to Several Hydroxy 5- and 6-Azaindoles. *Helv. Chim. Acta* **2022**, *105* (3), e202100211. https://doi.org/10.1002/hlca.202100211.

(13) An, J. H.; Kim, K. D.; Lee, J. H. Highly Chemoselective Deoxygenation of N-Heterocyclic *N*-Oxides Using Hantzsch Esters as Mild Reducing Agents. *J. Org. Chem.* **2021**, *86* (3), 2876–2894. https://doi.org/10.1021/acs.joc.0c02805.

(14) Nanjappa, C.; Hanumanthappa, S. K. T.; Nagendrappa, G.; Ganapathy, P. S. S.; Shruthi, S. D.; More, S. S.; Jose, G.; Sowmya, H. B. V.; Kulkarni, R. S. Synthesis, ABTS-Radical Scavenging Activity, and Antiproliferative and Molecular Docking Studies of Novel Pyrrolo[1,2-*a*]Quinoline Derivatives. *Synth. Commun.* **2015**, *45*, 2529–2545. https://doi.org/10.1080/00397911.2015.1085572.

(15) Bugaenko, D. I.; Tikhanova, O. A.; Karchava, A. V. Synthesis of Quinoline-2-Thiones by Selective Deoxygenative C-H/C-S Functionalization of Quinoline *N*-Oxides with Thiourea. *J. Org. Chem.* **2023**, *88* (2), 1018–1023. https://doi.org/10.1021/acs.joc.2c02433.

(16) Kim, D.; Ghosh, P.; Kwon, N. Y.; Han, S. H.; Han, S.; Mishra, N. K.; Kim, S.; Kim, I. S. Deoxygenative Amination of Azine-*N*-Oxides with Acyl Azides via [3 + 2] Cycloaddition. *J. Org. Chem.* **2020**, *85* (4), 2476–2485. https://doi.org/10.1021/acs.joc.9b03173.

(17) Çelikoğlu, M. H.; Uçar, S.; Nişancı, B. Deoxygenation of N-Heterocyclic *N*-Oxides with Selectfluor and Disulfane. *J. Heterocycl. Chem.* **2024**, *61* (7), 1029–1034. https://doi.org/10.1002/jhet.4818.

(18) Lu, M.; Liu, Y. Gold-Catalyzed Regio- and Stereoselective Alkenylation of Quinoline *N*-Oxides with Allenamides. *Org. Lett.* **2024**, *26*, 5493–5499. https://doi.org/10.1021/acs.orglett.4c01796.

(19) Torelli, A.; Choi, E. S.; Dupeux, A.; Perner, M. N.; Lautens, M. Stereoselective Kinugasa/Aldol Cyclization: Synthesis of Enantioenriched Spirocyclic β-Lactams. *Org. Lett.* **2023**, *25* (47), 8520–8525. https://doi.org/10.1021/acs.orglett.3c03534.

(20) Hoving, M.; Haaksma, J.-J.; Stoppel, A.; Chronc, L.; Hoffmann, J.; Beil, S. B. Triplet Energy Transfer Mechanism in Copper Photocatalytic *N*- and *O*-Methylation. *Chem. Eur. J.* **2024**, *30* (22), e202400560. https://doi.org/10.1002/chem.202400560.

(21) Xue, G.; Xie, F.; Liang, H.; Chen, G.; Dai, W. Copper-Catalyzed Oxidative C-C Bond Cleavage of Alkyl-(Hetero)Arenes Enabling Direct Access to Nitriles. *Org. Lett.* **2022**, *24* (30), 5590–5595. https://doi.org/10.1021/acs.orglett.2c02238.

(22) Wang, J.-R.; Song, Z.-Q.; Li, C.; Wang, D.-H. Copper-Catalyzed Methoxylation of Aryl Bromides with 9-BBN-OMe. *Org. Lett.* **2021**, *23* (21), 8450–8454. https://doi.org/10.1021/acs.orglett.1c03172.

(23) Todorov, A. R.; Wirtanen, T.; Helaja, J. Photoreductive Removal of *O*-Benzyl Groups from Oxyarene N-Heterocycles Assisted by *O*-Pyridine–pyridone Tautomerism. *J. Org. Chem.* **2017**, *82*, 13756–13767. https://doi.org/10.1021/acs.joc.7b02775.

(24) Beardmore, L. N. D.; Cobb, S. L.; Brittain, W. D. G. One-Pot Ester and Thioester Formation Mediated by Pentafluoropyridine (PFP). *Org. Biomol. Chem.* **2022**, *20*, 8059–8064. https://doi.org/10.1039/d2ob01268e.

(25) Karthik, S.; Muthuvel, K.; Gandhi, T. Base-Promoted Amidation and Esterification of Imidazolium Salts via Acyl C-C Bond Cleavage: Access to Aromatic Amides and Esters. *J. Org. Chem.* **2019**, *84* (2), 738–751. https://doi.org/10.1021/acs.joc.8b02567.

(26) Konev, M. O.; Cardinale, L.; Jacobi von Wangelin, A. Catalyst-Free *N*-Deoxygenation by Photoexcitation of Hantzsch Ester. *Org. Lett.* **2020**, *22*, 1316–1320. https://doi.org/10.1021/acs.orglett.9b04632.

(27) Rubio-Presa, R.; Fernández-Rodríguez, M. A.; Pedrosa, M. R.; Arnáiz, F. J.; Sanz, R. Molybdenum-Catalyzed Deoxygenation of Heteroaromatic *N*-Oxides and Hydroxides Using Pinacol as Reducing Agent. *Adv. Synth. Catal.* **2017**, *359*, 1752–1757. https://doi.org/10.1002/adsc.201700071.

(28) Wang, Y.; Genoux, A.; Ghorai, S.; Chen, H.; Todd, R.; Zhang, L. Direct Conversion of Internal Alkynes into α-Iodoenones: One-Step Collaborative Iodination and Oxidation. *Adv. Synth. Catal.* **2016**, *358* (9), 1417–1420. https://doi.org/10.1002/adsc.201600027.

(29) Mkrtchyan, S.; Shkoor, M.; Sarfaraz, S.; Ayub, K.; Iaroshenko, V. O. Mechanochemical Arylative Detrifluoromethylation of Trifluoromethylarenes. *Org. Biomol. Chem.* **2023**, *21*, 6549–6555. https://doi.org/10.1039/d3ob00787a.

(30) Sivendran, N.; Belitz, F.; Sowa Prendes, D.; Manu Martínez, Á.; Schmid, R.; Gooßen, L. J. Photochemical Sandmeyer-Type Halogenation of Arenediazonium Salts. *Chem. Eur. J.* **2022**, *28* (9), e202103669. https://doi.org/10.1002/chem.202103669.

(31) Muta, R.; Torigoe, T.; Kuninobu, Y. 3-Position-Selective C-H Trifluoromethylation of Pyridine Rings Based on Nucleophilic Activation. *Org. Lett.* **2022**, *24* (44), 8218–8222. https://doi.org/10.1021/acs.orglett.2c03327.

(32) He, K.-H.; Tan, F.-F.; Zhou, C.-Z.; Zhou, G.-J.; Yang, X.-L.; Li, Y. Acceptorless Dehydrogenation of N-Heterocycles by Merging Visible-Light Photoredox Catalysis and Cobalt Catalysis. *Angew. Chem. Int. Ed.* **2017**, *56* (11), 3080–3084. https://doi.org/10.1002/anie.201612486.

(33) Nicolaou, K. C.; Gross, J. L.; Kerr, M. A. Synthesis of Novel Hetereocycles Related to the Dynemicin A Ring Skeleton. *J. Heterocycl. Chem.* **1996**, *33*, 735–746. https://doi.org/10.1002/jhet.5570330336.

(34) Chouhan, N. K.; Ananthabhat, S. K.; Vaidya, S.; Srihari, P. A Scalable Process for the Synthesis of Key Intermediates Novoldiamine & Hydroxynovaldiamine and Their Utility in Chloroquine, Hydroxychloroquine and Mepacrine Synthesis. *Synth. Commun.* **2022**, *52* (7), 1004–1011. https://doi.org/10.1080/00397911.2022.2061358.

(35) Noh, J.; Cho, J.-Y.; Park, M.; Park, B. Y. Visible-Light-Mediated TiO_2-Catalyzed Aerobic Dehydrogenation of N-Heterocycles in Batch and Flow. *J. Org. Chem.* **2023**, *88*, 10682–10692. https://doi.org/10.1021/acs.joc.3c00743.

(36) Gerosa, G. G.; Schwengers, S. A.; Maji, R.; De, C. K.; List, B. Homologation of the Fischer Indolization: A Quinoline Synthesis via Homo-Diaza-Cope Rearrangement. *Angew. Chem. Int. Ed.* **2020**, *59* (46), 20485–20488. https://doi.org/10.1002/anie.202005798.

(37) Tiwari, V. K.; Kamal, N.; Kapur, M. One Substrate, Two Modes of C-H Functionalization: A Metal-Controlled Site-Selectivity Switch in C-H Arylation Reactions. *Org. Lett.* **2017**, *19* (1), 262–265. https://doi.org/10.1021/acs.orglett.6b03558.

(38) Shen, Y.; Chen, F.; Du, Z.; Zhang, H.; Liu, J.; Liu, N. Cu(I) Complexes Catalyzed the Dehydrogenation of N-Heterocycles. *J. Org. Chem.* **2024**, *89* (7), 4530–4537. https://doi.org/10.1021/acs.joc.3c02768.

(39) Espinoza, E.; Calrk, J. A.; Soliman, J.; Derr, J. B.; Morales, M.; Vullev, V. I. Practical Aspects of Cyclic Voltammetry: How to Estimate Reduction Potentials When Irreversibility Prevails, *J. Electrochem. Soc.* **2019**, *166* (5), H3175–H3187. https://doi.org/10.1149/2.0241905jes.

(40) Filippini, G.; Dosso, J.; Prato, M. Phenols as Novel Photocatalytic Platforms for Organic Synthesis. *Helv Chim Acta* **2023**, *106* (7), e202300059. https://doi.org/10.1002/HLCA.202300059.

(41) Zhao, Y.; Truhlar, D. G. The M06 Suite of Density Functionals for Main Group Thermochemistry, Thermochemical Kinetics, Noncovalent Interactions, Excited States, and Transition Elements: Two New Functionals and Systematic Testing of Four M06-Class Functionals and 12 Other Functionals and Inorganometallic Chemistry and for Noncovalent Interactions. *Theor. Chem. Acc.* **2008**, *120*, 215–241. https://doi.org/10.1007/s00214-007-0310-x.

(42) Hehre, W. J.; Ditchfield, R.; Pople, J. A. Self—Consistent Molecular Orbital Methods. XII. Further Extensions of Gaussian—Type Basis Sets for Use in Molecular Orbital Studies of Organic Molecules. *J. Chem. Phys.* **1972**, *56* (5), 2257–2261. https://doi.org/10.1063/1.1677527.

(43) Hariharan, P. C.; Pople, J. A. The Influence of Polarization Functions on Molecular Orbital Hydrogenation Energies. *Theor. Chim. Acta* **1973**, *28* (3), 213–222. https://doi.org/10.1007/BF00533485.

(44) Krishnan, R.; Binkley, J. S.; Seeger, R.; Pople, J. A. Self-consistent Molecular Orbital Methods. XX. A Basis Set for Correlated Wave Functions. *J. Chem. Phys.* **1980**, *72* (1), 650–654. https://doi.org/10.1063/1.438955.

(45) McLean, A. D.; Chandler, G. S. Contracted Gaussian Basis Sets for Molecular Calculations. I. Second Row Atoms, *Z*=11–18. *J. Chem. Phys.* **1980**, *72* (10), 5639–5648. https://doi.org/10.1063/1.438980.

(46) Francl, M. M.; Pietro, W. J.; Hehre, W. J.; Binkley, J. S.; Gordon, M. S.; DeFrees, D. J.; Pople, J. A. Self-consistent Molecular Orbital Methods. XXIII. A Polarization-type Basis Set for Second-row Elements. *J. Chem. Phys.* **1982**, *77* (7), 3654–3665. https://doi.org/10.1063/1.444267.

(47) Rassolov, V. A.; Ratner, M. A.; Pople, J. A.; Redfern, P. C.; Curtiss, L. A. 6-31G* Basis Set for Third-Row Atoms. *J. Comput. Chem.* **2001**, *22* (9), 976–984. https://doi.org/10.1002/jcc.1058.

(48) Weigend, F.; Ahlrichs, R. Balanced Basis Sets of Split Valence, Triple Zeta Valence and Quadruple Zeta Valence Quality for H to Rn: Design and Assessment of Accuracy. *Phys. Chem. Chem. Phys.* **2005**, *7* (18), 3297–3305. https://doi.org/10.1039/B508541A.

(49) Weigend, F. Accurate Coulomb-Fitting Basis Sets for H to Rn. *Phys. Chem. Chem. Phys.* **2006**, *8* (9), 1057–1065. https://doi.org/10.1039/B515623H.

(50) Luchini, G.; Alegre-Requena, J. V.; Funes-Ardoiz, I.; Paton, R. S. GoodVibes: Automated Thermochemistry for Heterogeneous Computational Chemistry Data. *F1000Res* **2020**, *9*, 291. https://doi.org/10.12688/f1000research.22758.1.

(51) Cancès, E.; Mennucci, B.; Tomasi, J. A New Integral Equation Formalism for the Polarizable Continuum Model: Theoretical Background and Applications to Isotropic and Anisotropic Dielectrics. *J. Chem. Phys.* **1997**, *107* (8), 3032–3041. https://doi.org/10.1063/1.474659.

(52) Mennucci, B.; Cancès, E.; Tomasi, J. Evaluation of Solvent Effects in Isotropic and Anisotropic Dielectrics and in Ionic Solutions with a Unified Integral Equation Method: Theoretical Bases, Computational Implementation, and Numerical Applications. *J. Phys. Chem. B* **1997**, *101* (49), 10506–10517. https://doi.org/10.1021/jp971959k.

(53) Mennucci, B.; Tomasi, J. Continuum Solvation Models: A New Approach to the Problem of Solute's Charge Distribution and Cavity Boundaries. *J. Chem. Phys.* **1997**, *106* (12), 5151–5158. https://doi.org/10.1063/1.473558.

(54) Tomasi, J.; Mennucci, B.; Cancès, E. The IEF Version of the PCM Solvation Method: An Overview of a New Method Addressed to Study Molecular Solutes at the QM Ab Initio Level. *J. Mol. Struct.:THEOCHEM* **1999**, *464* (1–3), 211–226. https://doi.org/10.1016/S0166-1280(98)00553-3.

(55) Scalmani, G.; Frisch, M. J. Continuous Surface Charge Polarizable Continuum Models of Solvation. I. General Formalism. *J. Chem. Phys.* **2010**, *132* (11), 114110. https://doi.org/10.1063/1.3359469.

(56) Marenich, A. V.; Cramer, C. J.; Truhlar, D. G. Universal Solvation Model Based on Solute Electron Density and on a Continuum Model of the Solvent Defined by the Bulk Dielectric Constant and Atomic Surface Tensions. *J. Phys. Chem. B* **2009**, *113* (18), 6378–6396. https://doi.org/10.1021/jp810292n.

(57) Frisch, M. J.; Trucks, G. W.; Schlegel, H. B.; Scuseria, G. E.; Robb, M. A.; Cheeseman, J. R.; Scalmani, G.; Barone, V.; Petersson, G. A.; Nakatsuji, H.; Li, X.; Caricato, M.; Marenich, J.; Bloino, A.; Janesko, B. G.; Gomperts, R.; Mennucci, B.; Hratchian, H. P.; Ortiz, J. V; Izmaylov, A. F.; Sonnenberg, J. L.; Williams-Young, D.; Ding, F.; Lipparini, F.; Egidi, F.; Goings, J.; Peng, B.; Petrone, A.; Henderson, T.; Ranasinghe, D.; Zakrzewski, V. G.; Gao, J.; Rega, N.; Zheng, G.; Liang, W.; Hada, M.; Ehara, M.; Toyota, K.; Fukuda, R.; Hasegawa, J.; Ishida, M.; Nakajima, T.; Honda, Y.; Kitao, O.; Nakai, H.; Vreven, T.; Throssell, K.; Montgomery Jr., J. A.; Peralta, J. E.; Ogliaro, F.; Bearpark, M.; Heyd, J. J.; Brothers, E.; Kudin, K. N.; Staroverov, V. N.; Keith, T.; Kobayashi, R.; Normand, J.; Raghavachari, K.; Rendell, A.; Burant, J. C.; Iyengar, S. S.; Tomasi, J.; Cossi, M.; Millam, J. M.; Klene, M.; Adamo, C.; Cammi, R.; Ochterski, J. W.; Martin, R. L.; Morokuma, K.; Farkas, O.; Foresman, J. B.; Fox, D. J. Gaussian 16, Revision B.01. Gaussian Inc., Wallingford CT 2016.

(58) The PyMOL Molecular Graphics System v. 2.0.7. Schrödinger, LLC.

(59) https://gist.github.com/bobbypaton (accessed 13 April 2018).

(60) Alegre-Requena, J. V.; Sowndarya S. V., S.; Pérez-Soto, R.; Alturaifi, T. M.; Paton, R. S. AQME: Automated Quantum Mechanical Environments for Researchers and Educators. *Wiley Interdiscip. Rev.:Comput. Mol. Sci.* **2023**, *13* (5), e1663. https://doi.org/10.1002/wcms.1663.

(61) Grimme, S. Supramolecular Binding Thermodynamics by Dispersion-Corrected Density Functional Theory. *Chem. Eur. J.* **2012**, *18* (32), 9955–9964. https://doi.org/10.1002/chem.201200497.

(62) Bailey, W. F.; Monahan, A. S. Statistical Effects and the Evaluation of Entropy Differences in Equilibrium Processes: Symmetry Corrections and Entropy of Mixing. *J. Chem. Educ.* **1978**, *55* (8), 489–493. https://doi.org/10.1021/ed055p489.

(63) Plata, R. E.; Singleton, D. A. A Case Study of the Mechanism of Alcohol-Mediated Morita Baylis-Hillman Reactions. The Importance of Experimental Observations. *J. Am. Chem. Soc.* **2015**, *137* (11), 3811–3826. https://doi.org/10.1021/ja5111392.

(64) Brute Force Symmetry Analyzer (1996, 2003).
